# Increasing the Chemical Space of L-SIGN Specific Glycomimetics

**DOI:** 10.1021/acs.jmedchem.5c01448

**Published:** 2025-10-18

**Authors:** Gianluca Cavazzoli, Clara Delaunay, Sara Pollastri, Andrea Panzeri, Sara Sattin, Michel Thépaut, Laura Belvisi, Franck Fieschi, Anna Bernardi

**Affiliations:** † Dipartimento di Chimica, 9304 Università degli Studi di Milano, via Golgi 19, Milano 20133, Italy; ‡ Institut de Biologie Structurale, Univ. Grenoble Alpes, CNRS, CEA, Grenoble 38000, France; § Institut Universitaire de France (IUF), Paris 75231, France

## Abstract

Selective ligands for the C-type lectin receptor L-SIGN offer promising avenues in antiviral therapies and for tissue-specific delivery. We recently reported that a guanidine-bearing modified mannose glycomimetic, called **Man84**, binds to L-SIGN with micromolar affinity and high-selectivity against the homologue lectin DC-SIGN. Here we describe a series of **Man84** isosteres (ligands **2–11**) that maintain or improve on this selectivity. The affinity of the ligands for L-SIGN, as well as their selectivity against DC-SIGN, were evaluated by Surface Plasmon Resonance inhibition assays using immobilized SARS-CoV-2 Spike protein. Compounds **4**, **5** and **9** were found to bind to L-SIGN with low micromolar affinity and 50–94-fold selectivity, thus matching or exceeding the performance of **Man84**. The crystal structure of the L-SIGN CRD/**4** complex was solved and highlighted the critical role of a bidentate H-bond interaction of the ligands with the side chain of E370 in L-SIGN.

## Graphical abstract

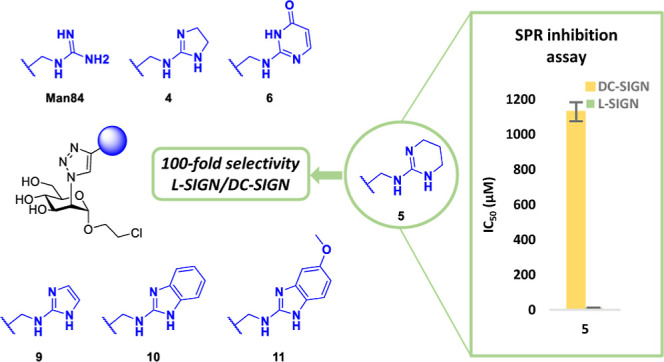

## Introduction

The recognition of glycans by carbohydrate-binding proteins, called lectins, regulates many physiological and pathological processes in living systems. Carbohydrate-lectin interactions participate in the activation of the innate immune system and in other cellular communication events involved in cancer, inflammation, infection. Specific lectin-glycan recognition pairs have been exploited for precision delivery of therapeutic and diagnostic agents to selected organs or cell types. The potential to manage a variety of conditions by specifically targeting lectins with selective antagonists is vast and yet this strategy is still largely untapped. By and large, this depends on the intrinsic difficulty of designing lectin ligands. Lectins have large and shallow binding sites, exposed to the solvent, which bind their native glycan ligands with low (millimolar to micromolar) affinity. They are considered challenging targets with low druggability. Glycomimetics have been employed to antagonize natural carbohydrates in lectins’ binding sites, and some designing principles have been put forward to generate high-affinity functional mimics, often taking advantage of multivalent presentations. Even when the affinity challenge can be overcome, a selectivity problem arises, since many lectins share a common specificity and recognize the same monosaccharide as minimal binding motif. Yet, selectivity is key to avoid off-target effects for drugs and targeting agents alike.^1^

A few notable examples of selective glycomimetic ligands of similar lectins have been reported.^2–15^ We have recently described a glycomimetic ligand, **Man84** (Figure 1),^16^ which affords 50-fold selectivity between two C-type lectin receptors (CLRs), L-SIGN and DC-SIGN, that share 82% amino acid sequence in their carbohydrate recognition domain (CRD). C-type lectin receptors are a class of pathogen recognition receptors (PRRs) that specialize in the recognition of carbohydrate-based motifs and bind them thanks to a Ca^2+^ ion embedded in the CRD. Glycoconjugates exposed on the surface of viruses and other pathogens are sensed and bound by CLRs in the native immune system. These recognition events normally induce a protective immune response for the host. However, several deadly viruses—such as HIV, Ebola, Dengue, hepatitis C viruses and SARS-CoV-1 and 2—have developed strategies to exploit CLRs in order to escape antiviral immunity and promote infection.^17–22^ During the coronavirus pandemic we^23^ and others^24,25^ reported that DC-SIGN and L-SIGN (also called CD209 and CD209L or DC-SIGNR, respectively) act as viral coreceptors: they capture SARS-CoV-2 by recognizing the glycan shield of its spike protein, but then promote trans-infection of competent cells that express SARS-CoV-2 internalization receptor, ACE2. We described several glycomimetic ligands able to antagonize the interaction of SARS-CoV-2 Spike with DC-SIGN and L-SIGN,^26^ and showed that a known polyvalent ligand of DC-SIGN, **PM26**,^27^ inhibits DC-SIGN mediated SARS-CoV-2 trans-infection in a cellular model.^23^

**Figure 1:**
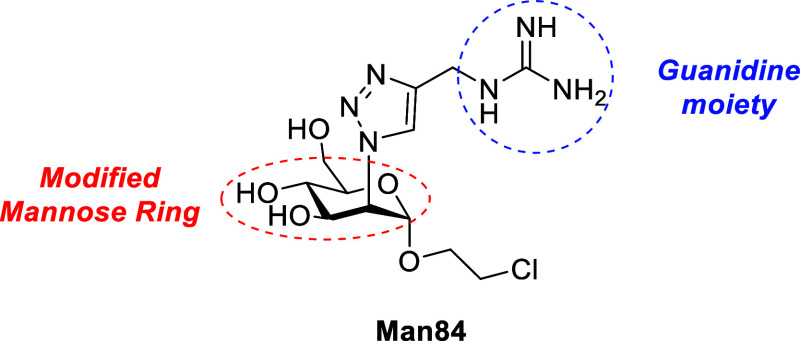
Structure of **Man84**.

However, in order to develop a host-directed therapeutic approach^28^ against corona virus infections, L-SIGN is a better target than DC-SIGN. Indeed, L-SIGN is coexpressed with ACE2 in the respiratory tract^29^ and, as opposed to DC-SIGN, does not have the potential of hyper-activating inflammatory pathways, which may reinforce some of the deadly characteristics of SARS-CoV-2 infections.^30^ Additionally, because of its narrow tissue distribution, selective targeting of L-SIGN is also attractive for tissue-selective delivery of drugs, particularly in the liver.^31^ This motivated us to pursue the search of selective L-SIGN ligands that ultimately led to the discovery of **Man84**.^16^

**Man84** (Figure 1) is a C2-modified mannose ring bearing a triazole and a pending guanidinium group,^16^ which binds L-SIGN with micromolar affinity and shows an impressive 50-fold selectivity over DC-SIGN. The selectivity of **Man84** could be traced to a single amino acid difference between the two lectins’ binding sites (N385 in L-SIGN vs K373 in DC-SIGN) and to a slight reorientation of a conserved phenylalanine side-chain (F325 and F313 in L-SIGN and DC-SIGN, respectively) (Figure 2A,B). To expand this initial hit, we here report a new set of mannose-based glycomimetics carrying a guanidinium isosteric group. We describe the synthesis of these molecules and the determination of their activity/selectivity for L-SIGN, as obtained by surface plasmon resonance (SPR experiments). The structure activity relationship analysis of the series is supported by a new X-ray structure obtained for the L-SIGN complex of one of the most active ligands.

**Figure 2:**
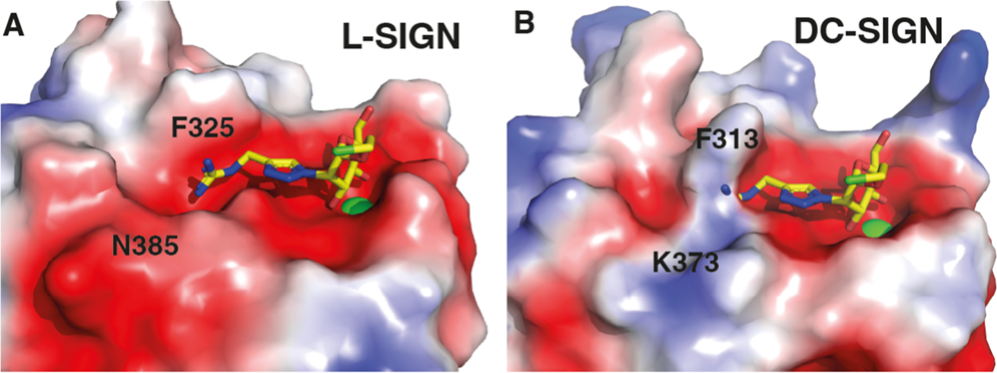
(A) X-ray structure of **Man84** in L-SIGN (PDB 8RCY); (B) Rigid docking of **Man84** in DC-SIGN: the K373 side chain results in an electrostatic repulsive effect with the guanidinium moiety and in a strong steric hindrance assisted by the orientation of the F313 moiety (modified from ref 16).

## Results and Discussion

### Synthesis

Ligands **2–11** (Scheme 1) were prepared from the 2-azido-mannoside intermediate **1**,^4^ through a Copper-catalyzed azide–alkyne cycloaddition (CuAAC) reaction with the appropriate set of functionalized alkynes, **12–21** (Figure 3), followed by removal of protective groups. This approach proved to be more successful than an earlier trial to produce the same compounds by late-stage modification of a methyleneamino triazole intermediate.^26^

**Scheme 1:**
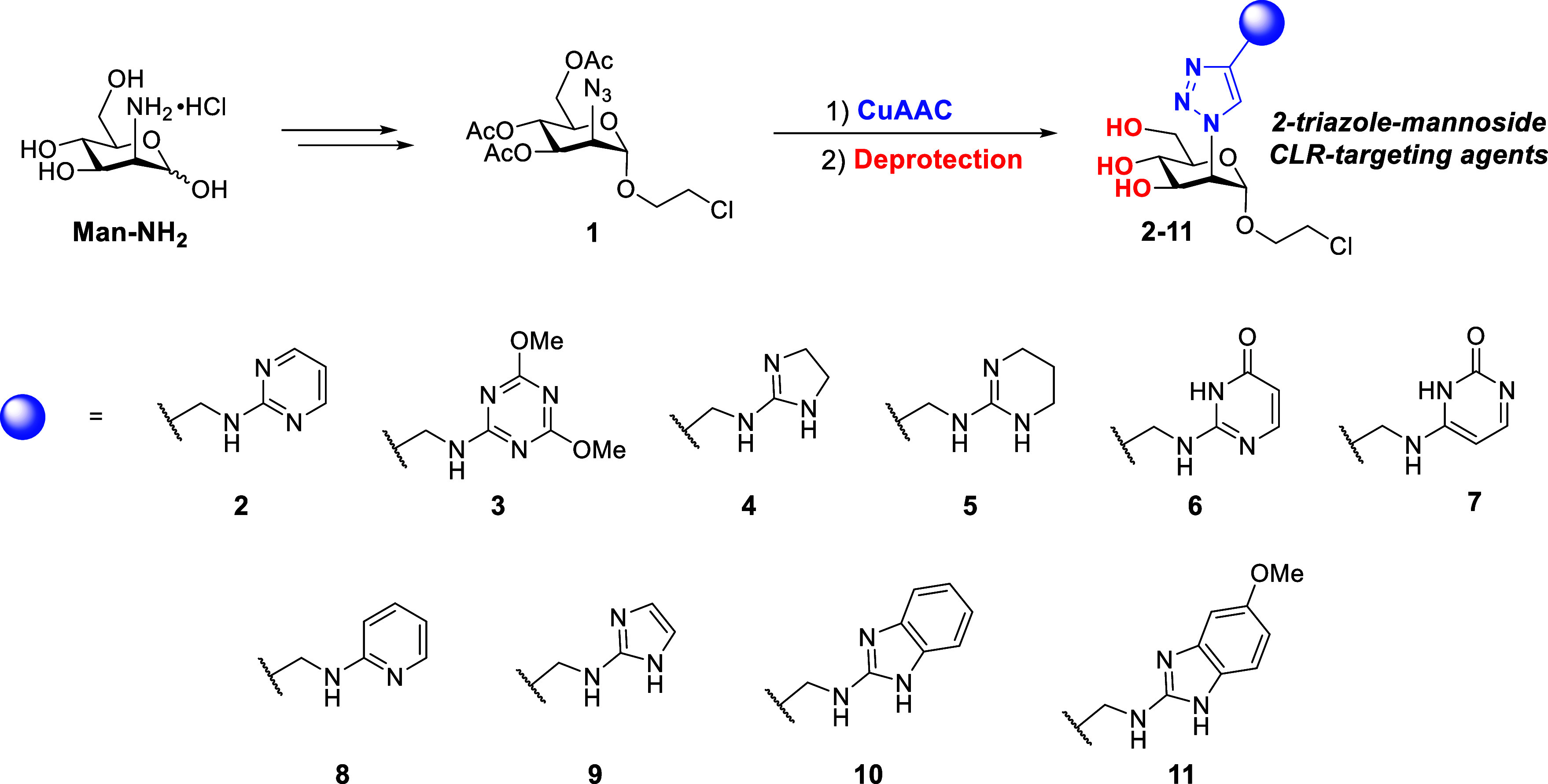
General Synthetic Pathway for the Proposed Set of Mannose-Based Glycomimetics

**Figure 3:**
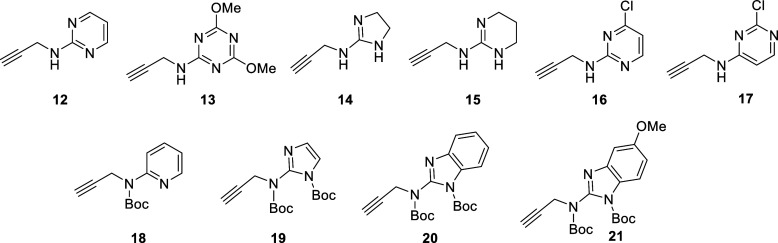
Chemical structures of alkynes **12–21**.

Two different strategies were adopted to synthesize the required alkynes, depending on their structure, i.e. displacement of a leaving group (chloride/thiomethyl) on the guanidine isosteric moiety by propargylamine (alkynes **12–17**, Figure 3 and Scheme 2), or alkylation of a carbamate by propargyl bromide (alkynes **18–21**, Figure 3 and Scheme 3).

**Scheme 2:**
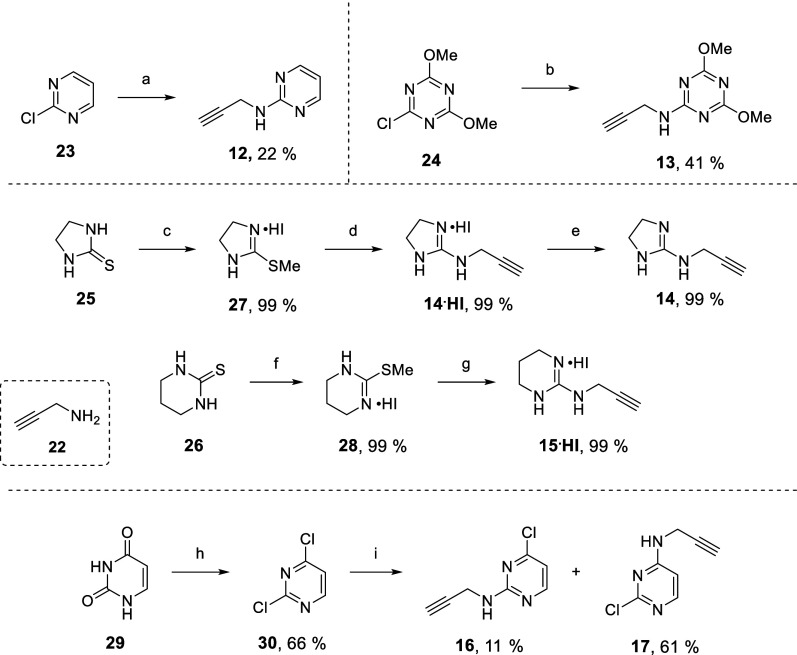
Synthesis of the Alkynes **12-17** by Nucleophilic Substitution with Propargylamine **22** ^a^

a Reagents and conditions: (a) **22**, DIPEA, CH_3_CN, reflux, 16 h; (b) **22**, DIPEA, THF, room temperature, 1 h; (c) CH_3_I, EtOH, room temperature, 12 h; (d) **22**, THF, 40 °C, 24 h; (e) NaOH (40% *aq*), CH_2_Cl_2_, room temperature, 15 min; (f) CH_3_I, EtOH, room temperature, 12 h; (g) **22**, THF, 55 °C, 120 h; (h) POCl_3_, 120 °C, 2 h; (i) **22**, DIPEA, CH_3_CN, room temperature, 24 h.

**Scheme 3:**
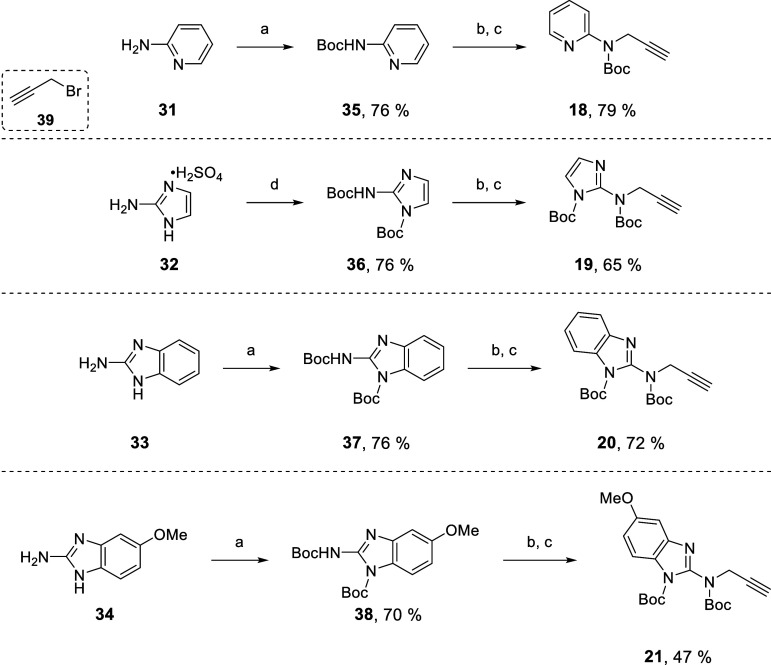
Synthesis of Alkynes **18-21** by *N*-Alkylation with Propargyl Bromide **39** ^a^

a Reagents and conditions: (a) Boc_2_O, Et_3_N, CH_2_Cl_2_, room temperature, 16 h; (b) NaH, DMF, 0 °C, 30 min; (c) **39**, room temperature, 4 h; (d) Boc_2_O, NaOH (2 M *aq*), CH_2_Cl_2_, room temperature, 18 h.

### Synthesis of the Alkynes **12–21**

Alkynes **12** and **13** were obtained by reaction of propargylamine **22** with 2-chloropyrimidine **23** or 2-chloro-4,6-dimethoxytriazine **24**, respectively, in moderate to low yields (Scheme 2, top).

The synthesis of alkynes **14** and **15** (Scheme 2, middle) started from the commercially available thiones **25** and **26,** that were treated with MeI, leading to iodide salts **27** and **28,** respectively. Both salts reacted with propargylamine **22** in good to excellent yields, affording the *N*-alkylation products as the HI salts **14**·**HI** and **15**·**HI**, respectively. The free base **14** was obtained from **14**·**HI** upon treatment with 40% NaOH and extraction in CH_2_Cl_2_. The salt **15**·**HI** was used directly in the subsequent CuAAC reaction (see below).

Alkynes **16** and **17** were synthesized from uracil **29** (Scheme 2, bottom). Reaction with POCl_3_ (neat, reflux) afforded dichloropyrimidine **30**, which underwent a nucleophilic substitution reaction with propargylamine, leading to **17** as the major product (61%), as judged by NOESY experiments (see Supporting Information). The regioisomer **16** was recovered as a byproduct (11%) by flash column chromatography.

Alkynes **18–21** could not be obtained with the same approach and an alternative strategy was developed based on propargyl bromide (Scheme 3). The required heteroaromatic amines **31–34** were treated with Boc_2_O, then the resulting carbamates **35–38** were activated with NaH and alkylated with propargyl bromide **39**, leading in high yields to alkynes **18–21**, which were used directly in the subsequent CuAAC.

### Synthesis of Ligands **2–11**

CuAAC reaction (CuSO_4_·5H_2_O and Na-ascorbate in a H_2_O/THF mixture) of 2-azidomannoside **1** with the appropriate alkynes (Scheme 4) afforded triazoles **40**, **41** and **44**–**49** in good yields, after solvent evaporation and chromatography (Scheme 4A). Due to the high p*K*_a_ of imidazolinium salts (see Table 1), compound **42** was not isolated as such from the CuAAC crude. Rather, the crude was suspended in AcOEt and washed with NaOH. This step removed the ascorbic acid and deprotected the sugar. Treatment of the residue with HCl allowed to isolate **4**, directly, as the HCl salt (50% yield, Scheme 4B). Similarly, the *O*-acetyl triazole **43** could not be obtained, because the HI salt **15**·**HI** (Scheme 2, middle) could not be neutralized to afford **15** and all our attempts led to decomposition. Instead, **15**·**HI** was installed directly on the unprotected 2-azidomannoside **50** by CuAAC (Scheme 4C), to give the final, unprotected mannoside **5**. Probably due to the formation of unfavorable redox couples related to the presence of I_2_ traces, this product could only be recovered in low yield (ca. 10%).

**Scheme 4:**
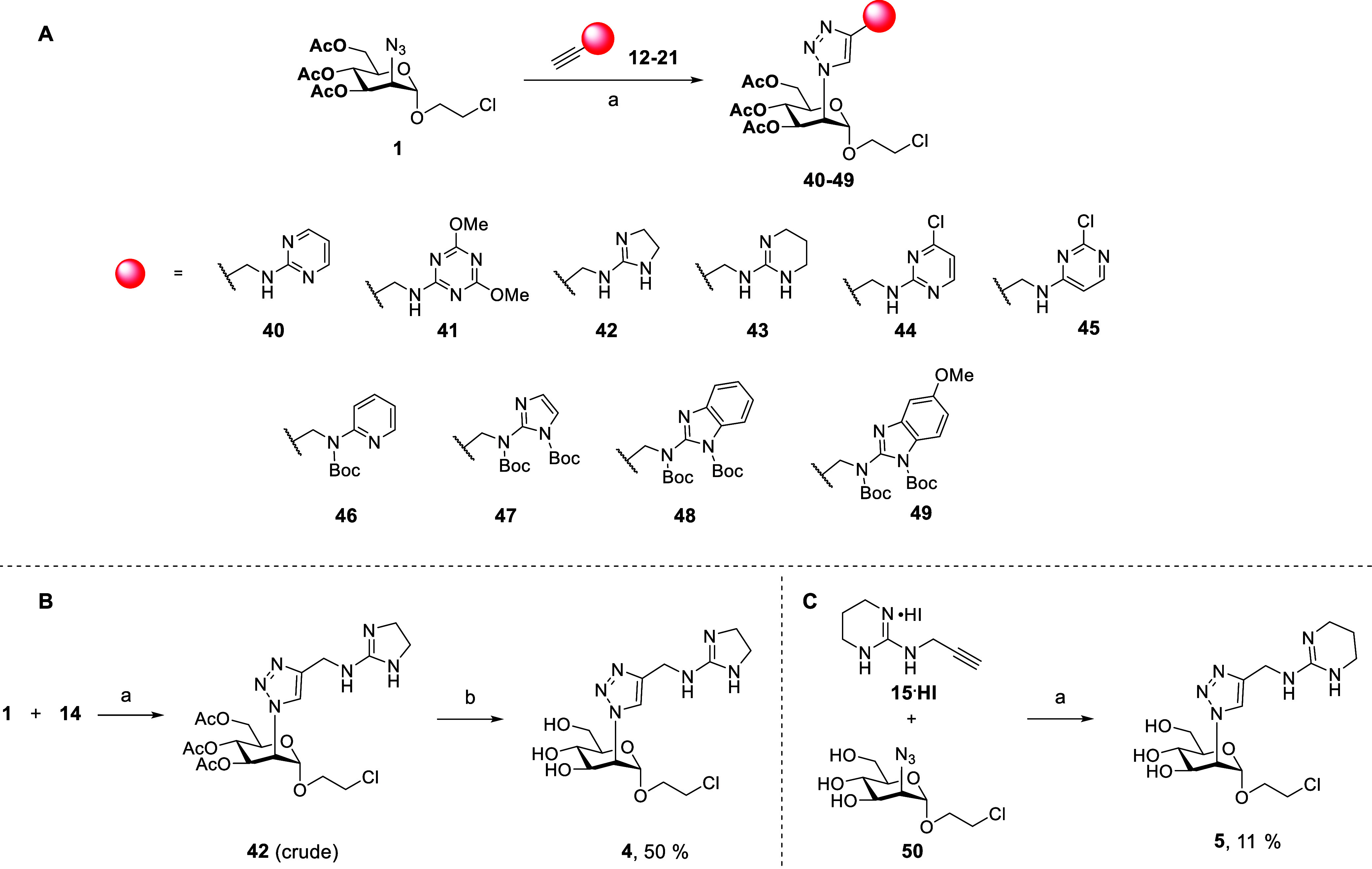
Synthesis of the Triazoles **40-49** ^a^

a Reagents and conditions: (a) CuSO_4_·5H_2_O, Na-ascorbate,H_2_O/THF, room temperature; (b) NaOH (40% *aq*), AcOEt then HCl (1M).

**Table 1:** IC_50_ Values (μM) and Resulting Selectivity^a^

ligand	L-SIGN IC_50_ (μM)	DC-SIGN IC_50_ (μM)	Sel^a^	p*K*_a_ ^b^
51 [Man79]	278 ± 7	318 ± 1	1	7.98
Man84	19.00 ± 0.02	1162 ± 25	61	10.56
2	602.0 ± 0.5	798 ± 16	1	3.50
3	453.0 ± 0.4	555 ± 14	1	0.44
4	15.00 ± 0.01	1133 ± 26	76	11.42
5	12.00 ± 0.01	1129 ± 55	94	12.86
6	30.0 ± 0.2	750 ± 29	25	3.46
7	130 ± 1	692 ± 8	5	3.98
8	101.0 ± 0.2	822 ± 16	8	6.90
9	18.0 ± 0.1	895 ± 8	50	8.40
10	48.0 ± 0.4	779 ± 24	16	6.79
11	51.6 ± 0.7	602 ± 12	12	6.95

a Selectivity = (IC_50_ [DC-SIGN]/IC_50_ [L-SIGN]).

b Calculated for the conjugated acid of the heterocyclic fragment by Epik, vers. 4.3011 (Schrodinger 2018).

In the final steps of the syntheses, protecting groups were removed (Scheme 5). The acetyl groups of compounds **40** and **41** were solvolyzed under Zemplèn conditions (cat. MeONa in MeOH), affording, in excellent yields, ligands **2** and **3**, respectively. Acid-catalyzed transesterification (HCl, EtOH in CHCl_3_) of the acetyl groups in **44** and **45** also hydrolyzed the aryl chlorides, affording the isocytosine derivative **6** and the cytosine isomer **7**, both in excellent yields. Finally, Zemplèn methanolysis of **46–49**, followed by removal of the Boc carbamates (TFA in CH_2_Cl_2_) afforded **8–11** as their TFA salts (Scheme 5).

**Scheme 5:**
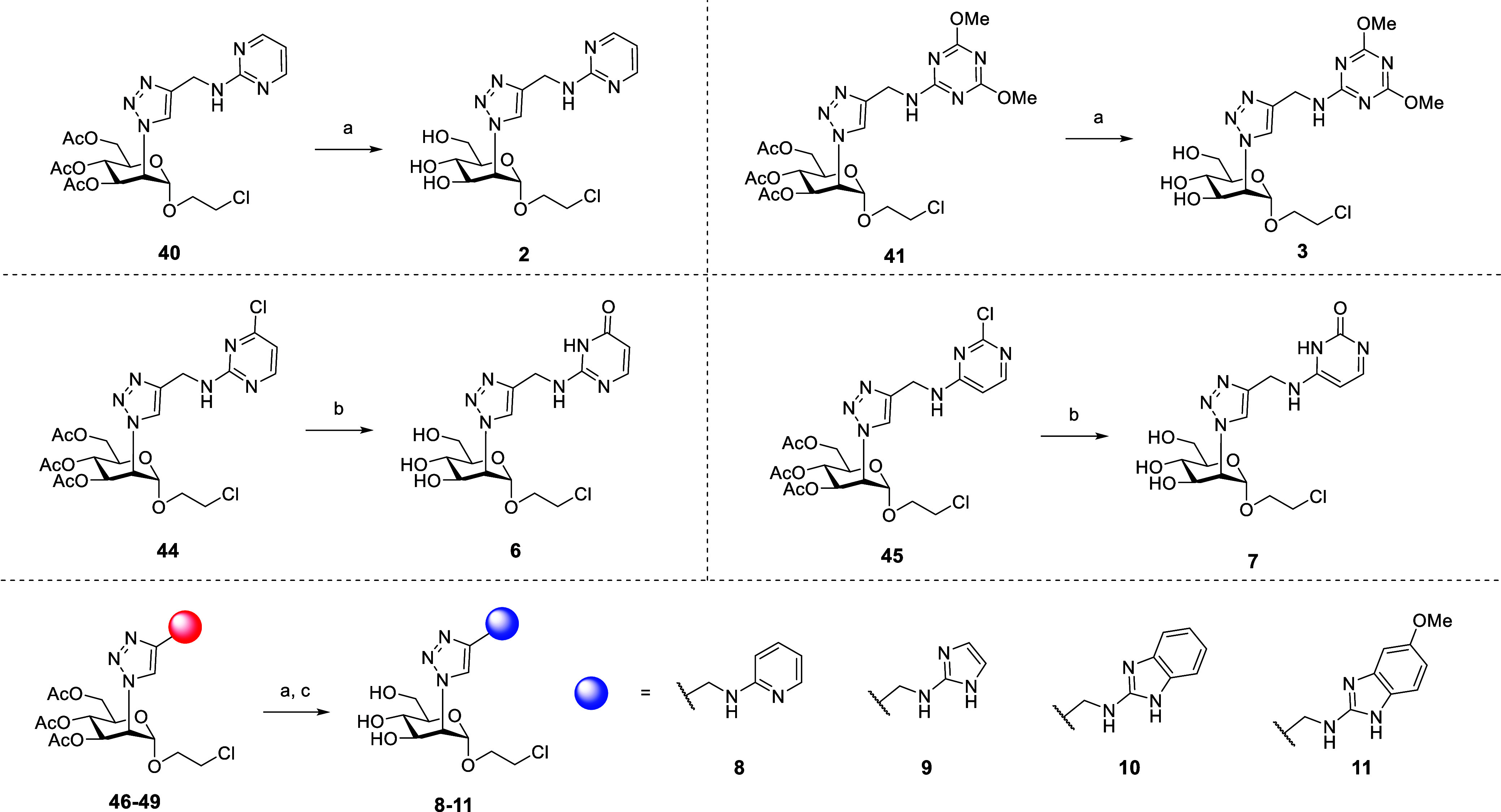
Global Deprotection of Triazoles **40**, **41**, **44-49** ^a^

a Reagents and conditions: (a) NaOMe in MeOH, room temperature, 1 h; (b) HCl, EtOH, CHCl_3_, 40 °C, 16 h; (c) trifluoroacetic acid:CH_2_Cl_2_ (1:4), room temperature, 3h.

### Binding Inhibition Assays on SARS-CoV-2 Spike Surface

We determined the activity of compounds **2–11** toward L-SIGN and DC-SIGN through a surface plasmon resonance (SPR) inhibition experiment^26^ using the fully glycosylated stabilized-SARS-CoV-2 Spike protein as interaction reporter.^32^ The methyleneamino derivative **51** [**Man79**][Bibr ref16] was employed as an unselective control.

As an example, the sensorgrams and corresponding inhibition curve for compound **4** are shown in Figure 4. The same data for all other compounds are collected in the Supporting Information (Sensorgrams section).

**Figure 4:**
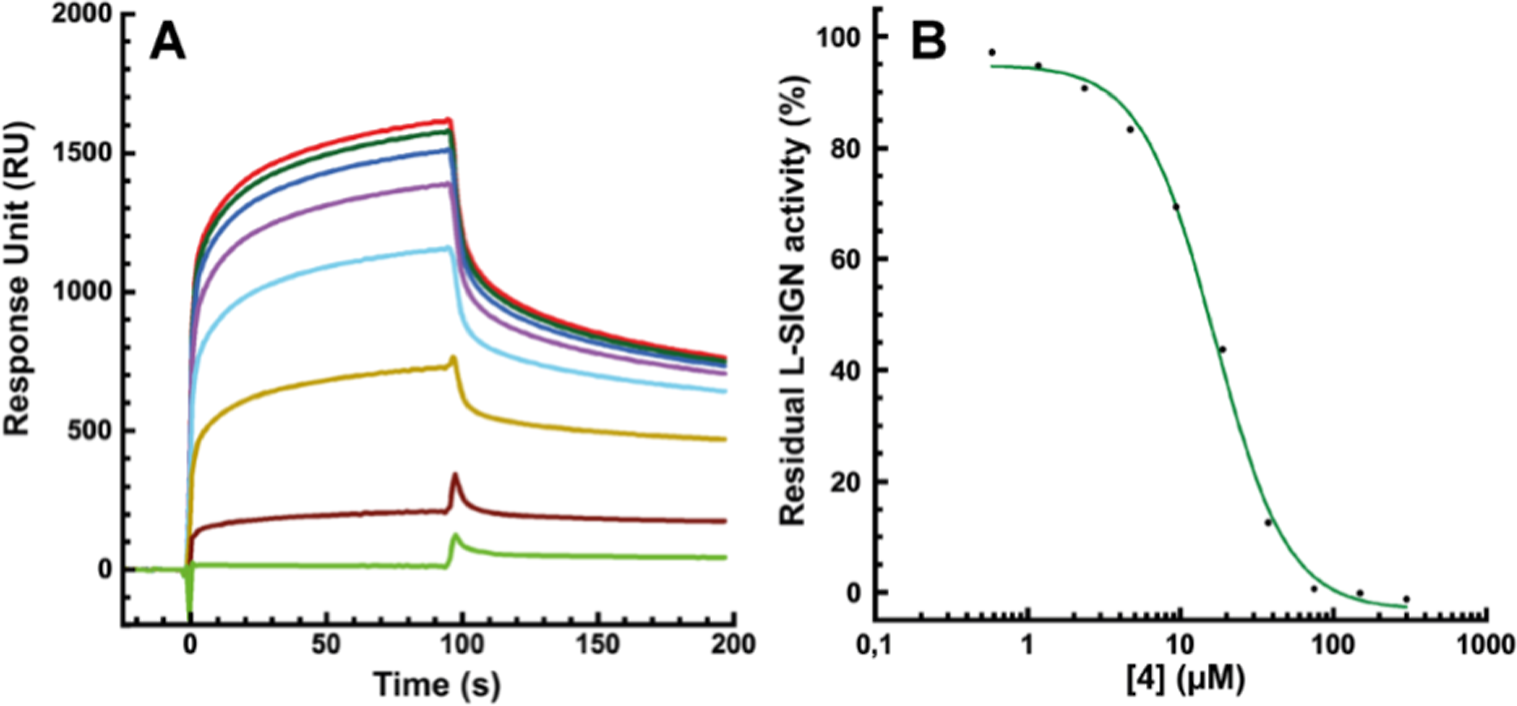
SPR inhibition experiments: (A) Sensorgrams of L-SIGN binding inhibition by **4**. The range of ligand concentration goes from 300 μM to 0.6 μM by serial dilution by a factor of 2. (B) SPR inhibition curve for **4** (see ESI† for all sensorgrams).

As shown in Figure 5 and Table 1, **Man84** confirmed its selectivity and high affinity for L-SIGN, in stark contrast with the corresponding amino derivative **51**, that binds both lectins with IC_50_ ca. 300 μM.^16^ Low affinity and no selectivity were also observed for the aminopyrimidine derivative **2** and the triazine **3.** The cytosine derivative **7** and the 2-aminopyridine **8** showed a modest increase in affinity for L-SIGN, with moderate selectivity (5-fold and 8-fold, respectively). On the contrary, ligands **4–6** and **9–10** showed high affinity and a strong selectivity (1- to 2-orders of magnitude) for L-SIGN. In particular, the 2-aminoimidazoline **4**, the 2-aminotetrahydropyrimidine **5** and the 2-aminoimidazole **9**, together with the guanidine **Man84** showed the lowest IC_50_ values in the series (IC_50_ 15.00 ± 0.01 μM, 12.00 ± 0.01 μM, 18.00 ± 0.10 μM and 19.00 ± 0.02 μM, respectively).

**Figure 5:**
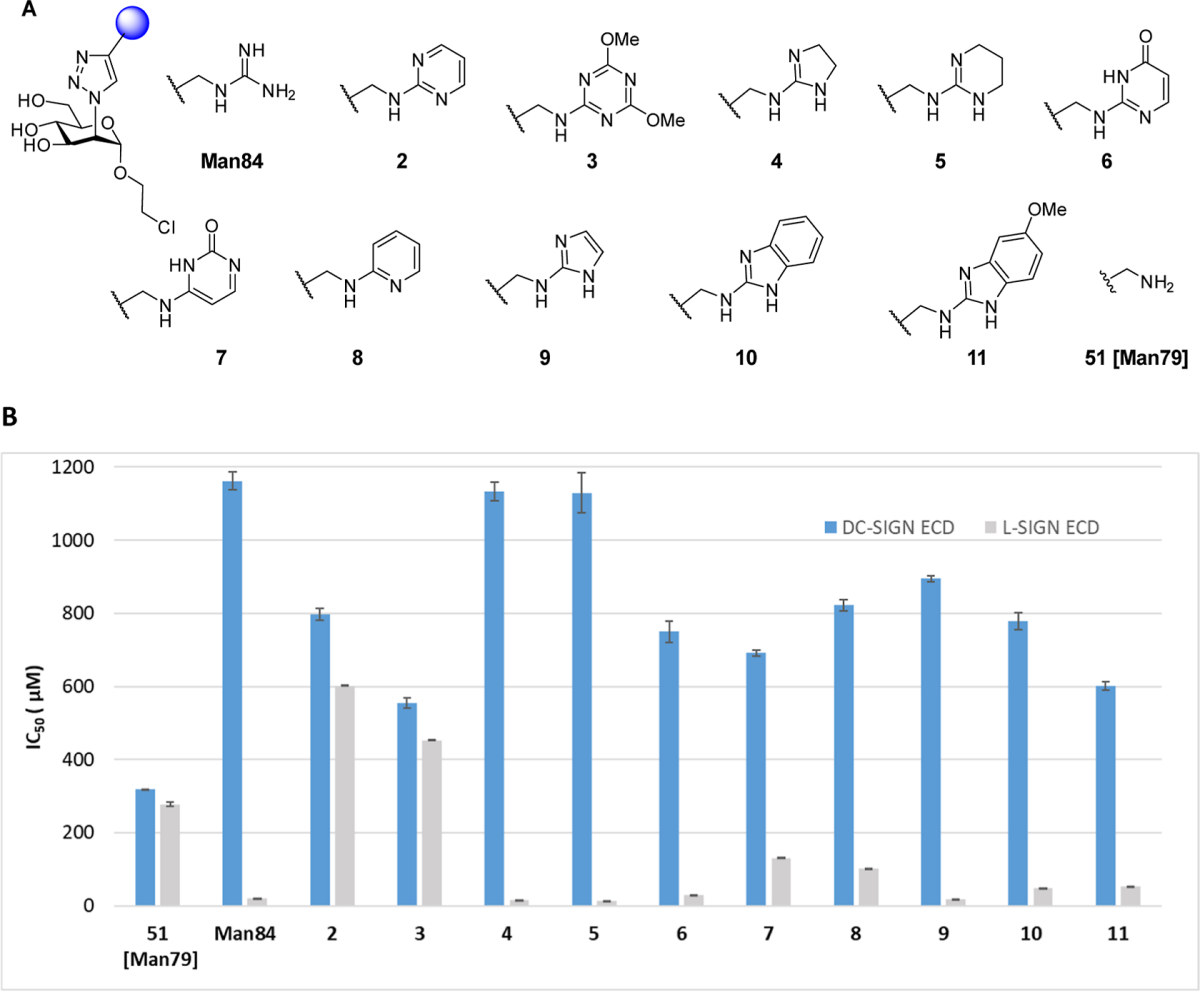
Binding inhibition assay of **2–11, Man84** and **51** (SPR with Spike functionalized chip, duplicates, pH 8). L-SIGN or DC-SIGN (20 μM) and ligands at increasing concentrations were coinjected. (A) List of compounds; (B) graphical representation of the data.

### X-ray Structure of the L-Sign CRD/**4** Complex

To interpret these affinity data, we solved the structure of the complex formed with L-SIGN CRD by compound **4**, one of the best ligands in the series (Figure 6C), and compared it to the crystal structure already available for the L-SIGN CRD/**Man84** complex (Figure 6A).^16^ The latter structure showed that the guanidium fragment of **Man84** forms a dense array of interactions in the binding site that includes: an ion pair and a bidentate H-bonding interaction with the side chain of E370, a stacking (cation−π) interaction with the side chain of F325, a H-bonding interaction with the side-chain of N385 and a water mediated H-bond interaction with the side-chain of Q286 (Figure 6A). A similar recognition pattern is now observed in the L-SIGN/**4** complex (Figure 6C), with a few site adaptations. The ion pair/bidentate binding of E370 is confirmed, as well as the stacking with F325 and the H-bond interaction with N385. The water mediated contact with Q286 is lost in the complex of **4** and replaced by H-bond interactions, also mediated by water molecules, between the triazole ring of the ligand and N379. Other small differences are observed in the ligand pose and in the position of F325 and N385 (Figure 6D), both of which move closer to the imidazoline ring of **4** than to the guanidine of **Man84** (see superimposition of the two structures in Figure 6D). Thus, the bidentate binding of E370, the stacking interaction with F325 and the H-bonding interaction with N385 are confirmed as the central features of this class of selective L-SIGN ligands. The plasticity of the protein and the extensive and adaptive first solvation shell of the complex also play subtle roles in complex stabilization. As we previously reported, N385 is replaced by K373 in DC-SIGN and this residue disrupts the guanidium binding site, as supported by NMR data that we discussed in ref 16 (see Figure 2).^16^ Additional discussions concerning the predicted binding mode of the unselective ligand **51** (**Man79**) in both lectins are reported as Supporting Information (Figure SI6).

**Figure 6:**
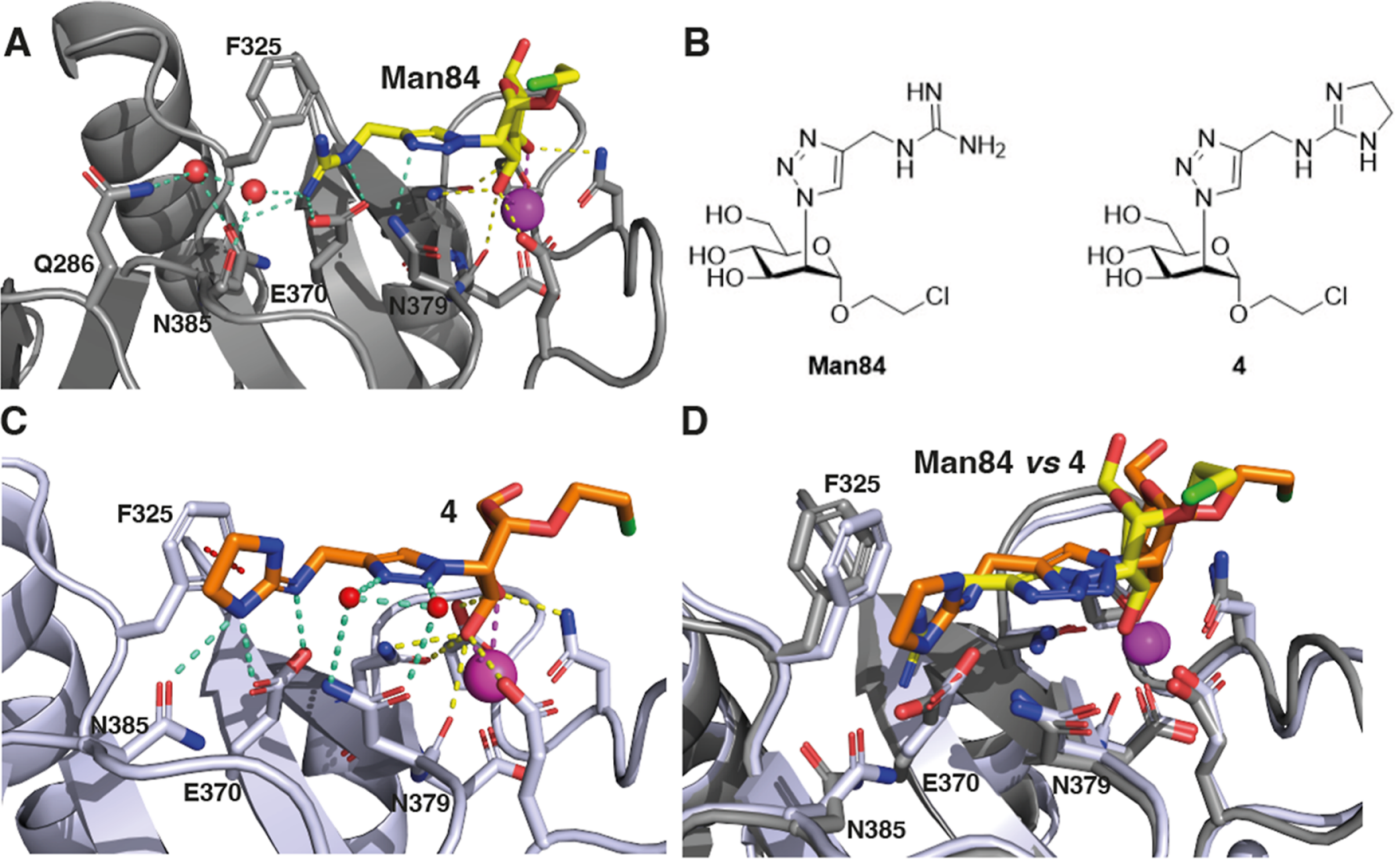
Structure of the L-SIGN CRD complex with **Man84** (yellow) and isostere **4** (orange). The ligands are shown as sticks; the L-SIGN CRD is represented as a cartoon, the side chain of residues involved in binding are shown as sticks. In (A,C), H-bond interactions are represented as dashed lines (cyan-blue for the aglycone part, yellow for the conserved mannose ring), Ca^2+^ coordination bonds with the mannose ring are represented as magenta dashed line and cation–π interactions by red dashed lines. Canonical Ca^2+^ coordination bonds with L-SIGN CRD residues are not shown for clarity. Water molecules are represented as red spheres. (A) Crystallographic structure of L-SIGN/**Man84** (PDB 8RCY) adapted from ref 16. (B) Chemical structure of **Man84** and **4**. (C). Crystallographic structure of L-SIGN/**4** (PDB: 9G6W). (D) Structural alignment of L-SIGN CRD in complex with **Man84** and **4** (PDB: 8RCY and 9G6W). The L-SIGN CRD backbone and relevant side-chains are shown in gray for 8RCY (**Man84**) and white-blue for 9G6W (**4**). See Table SI2 for data collection and structure refinement statistics).

### Structure Activity Relationship of **2–11**

The two crystal structures that we have solved facilitate an atomic level analysis of the structure activity relationship for ligands **2–11**. The essential interactions which are conserved for both **Man84** and **4** in the L-SIGN CRD are a bidentate H-bonding interaction and an ion pair electrostatic interaction with the side chain of E370, a stacking (cation−π) interaction with the side chain of F325 and a H-bonding interaction with the side-chain of N385. In order to reproduce this interaction mode, the isosteres need to deploy a minimum of 2 N–H groups and, possibly, a positive charge at the experimental pH (pH 8). Thus, the p*K*_a_ values of the isosteric moiety (values estimated by Epik are collected in Table 1) are clearly a contributing factor to ligands’ affinity. This is visible comparing compounds **2** and **8**: despite the very similar structure, their affinity differs by a factor of 5, which can be related to the different estimated p*K*_a_ values (3.5 for **2** and 6.9 for **8**). More generally, the trend is clearly shown by plotting p*K*_a_ vs L-SIGN IC_50_ (see correlation graph in Figure SI1). In fact, the most basic ligands **Man84** (*pK*_*a*_ (conj. acid) = 10.56, IC_50_ = 19 μM), **4** (*pK*_*a*_ (conj. acid) = 11.42, IC_50_ = 15 μM) and **5** (*pK*_*a*_ (conj. acid) = 12.86, IC_50_ = 12 μM) are among the strongest L-SIGN binders analyzed. At the other end of the p*K*_a_ range, ligands **2** and **3**, neutral at pH = 8 and lacking a second NH in the neutral form, cannot develop the required interactions with E370 and are the weakest L-SIGN binders in the series (Table 1). Ligands **8** (*pK*_*a*_ (conj. acid) = 6.90, IC_50_ = 101 μM), **10** (*pK*_*a*_ (conj. acid) = 6.79, IC_50_ = 48 μM) and **11** (*pK*_*a*_ (conj. acid) = 6.95, IC_50_ = 51.6 μM), with p*K*_a_ values around 7, will be only partially protonated at pH 8, leading to moderate affinity.

However, p*K*_a_ is not the only factor that determines L-SIGN affinity, as clearly seen by comparing the aminoimidazole derivative **9** and the amine **51 (Man79)**. The calculated p*K*_a_ of the two functional groups is close (8.40 for **9** and 7.98 for **51**) and yet their IC_50_ values for L-SIGN differ by 15-fold (Table 1 and Figure SI2). This reveals the influence of the bidentate H-bond interaction with E370 carboxylate: the 2-aminoimidazole **9**, only partially protonated at pH 8, can establish a bidentate H-bond interaction even in its neutral form and its affinity (IC_50_ = 18 μM) reaches the same range as the more basic counterparts **Man84, 4** and **5.** The amine **51**, with a similar p*K*_a_, can generate the same kind of electrostatic interactions with the protein, but cannot form a bidentate H-bond with E370: its affinity drops and the IC_50_ is 278 μM. Similarly, ligands **10** and **11** can engage E370 in a bidentate interaction even in their neutral form, and their affinity is 2-fold higher than the affinity of **8**, which can generate this type of interaction only in the protonated form.

Finally, ligands **6** and **7** appear as outliers in this SAR. Both molecules, with p*K*_a_ of 3.46 and 3.98, respectively, are not charged at pH = 8, but both possess two N–H functionalities in the neutral form. Therefore, like **10** and **11**, they are expected to fall in a middle range of affinity/selectivity. The affinity difference between **6** (IC_50_ = 30 μM) and **7** (IC_50_ = 130 μM) can be understood on the basis of their tautomeric equilibria (Figure 7) and of their docking poses in L-SIGN (Figure SI5). In the free state, tautomer ***A*** is favored by both ligands, but tautomer ***B*** provides the best poses in the L-SIGN complexes. Indeed, the steric hindrance exerted by the carbonyl group when **6** tautomer ***A*** (Figure 7) forms the bidentate H-bond interaction with E370 can be relieved using tautomer **B**, which provides optimal docking poses in L-SIGN and shows all the key interactions available to a neutral ligand (Figure SI5, top). The best poses of ligand **7** are also provided by its tautomer ***B***, but the hindrance generated by the carbonyl group disrupts the bidentate interaction, which is replaced by a single H-bond contact with E370 (Figure SI5, bottom). Hence the IC_50_ measured for this ligand (130 μM) is similar to that of the 2-aminopyridine **8**.

**Figure 7:**
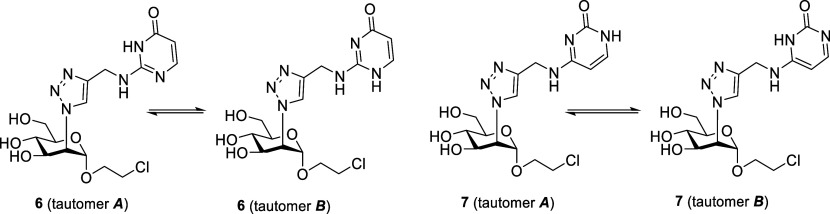
Tautomeric equilibria for ligands **6** and **7**.

Concerning L-SIGN vs DC-SIGN selectivity, it can be noted from Table 1 that this is mostly dependent on the L-SIGN IC_50_ values, since the affinity for DC-SIGN of **Man84** and all its isosteres is uniformly poor. Three clusters can be identified in Figure SI3, which shows the L-SIGN IC_50_ vs selectivity correlation: the poor L-SIGN ligands **2, 3** and **51** (**Man79**) are totally unselective; the middle range affinity ligands **6–8**, **10** and **11** also display a midrange selectivity (selectivity factors between 5 and 25); the high-affinity ligands **4**, **5**, **9** and **Man84** are strongly selective, with selectivity factors varying from 50 to 94. In this group, ligand **9** (calculated p*K*_a_: 8.4) is the only molecule which is not expected to carry a permanent charge at physiological pH. As such, it probably represents the best candidate to be developed for further studies.

## Conclusions

Selective ligands of the C-type lectin receptor L-SIGN can have important applications in anti-Covid therapies, since this lectin was found to act as a coreceptor of SARS-CoV-2.^23^ Targeting L-SIGN with selective agents is also of interest for tissue-selective delivery of therapeutic agents and diagnostics, particularly in liver sinusoidal endothelial cells, where the lectin is highly expressed. To expand the chemical space of selective L-SIGN ligands, we have synthesized ten new 2-triazolyl-mannosides **2–11** (Scheme 1) that carry a guanidine isosteric group on the triazole moiety and have compared them with the guanidine-carrying parent structure **Man84**. The affinity of the ligands for L-SIGN and their selectivity vs DC-SIGN have been established by surface plasmon resonance binding inhibition studies, using immobilized SARS-CoV-2 Spike as a reporter (Table 1).^32^ Three of the new ligands, compounds **4**, **5** and **9** were found to bind L-SIGN with low micromolar affinity and 50–94-fold selectivity, which are comparable to, or better than, those previously obtained for **Man84**. The crystal structure of the complex formed by **4** with L-SIGN CRD was solved, and together with the already available structure of the same complex for **Man84**, allowed to understand at the atomic level the structure activity relationship for ligands **2–11.** Three interactions established by the guanidium moiety were found to be conserved in both structures: a bidentate H-bonding interaction and an ion pair electrostatic interaction with the side chain of E370, a stacking (cation−π) interaction with the side chain of F325 and a H-bonding interaction with the side-chain of N385. Together, these features explain the variability of the affinity in the series **2–11**, its partial dependence on the molecules’ p*K*_a_, as well as the selectivity against DC-SIGN, which is determined solely by the L-SIGN affinity (Figure SI3). Ligand **9**, which carries a 2-aminoimidazole group as the guanidine isostere, binds L-SIGN with an IC_50_ 18.0 ± 0.1 μM and shows a 50-fold selectivity against DC-SIGN. Overall, this is probably the most interesting candidate to develop, because it has the same affinity of a guanidine for L-SIGN, but the basicity of an amine (calc. p*K*_a_ 8.4). Finally, we point out that the synthesis of **2–11** was achieved by CuAAC reaction of a 2-azido-mannoside **1** with the appropriate alkynes **12–21** (Figure 3). These alkynes are likely to find applications in a number of other situations where a guanidine-like moiety is called for.

## Experimental Section

### General

All commercial reagents (Abcr, Carbosynth and Merck) were used without further purification, unless otherwise indicated. When anhydrous conditions were required, the reactions were performed under a N_2_ atmosphere. Anhydrous solvents were purchased from Merck with water content ≤0.0005%. Triethylamine, *N*,*N*-diisopropylethylamine (DIPEA), CH_2_Cl_2_, CH_3_CN and MeOH were dried over calcium hydride, THF was dried over sodium/benzophenone and freshly distilled. All solvents were of reagent grade or HPLC grade. Reactions were monitored by analytical thin-layer chromatography (TLC) performed on Silica Gel 60 F254 plates (Merck) or Silica Gel 60 RP-18F254s plates (Merck) with UV detection (254 and 366 nm) and/or staining with ceric ammonium molybdate reagent, potassium permanganate, ninhydrin or iron trichloride. Flash chromatography was performed according to Still’s procedure^33^ using Silica gel Macherey-Nagel 60 (40–63 μm, 230–400 mesh). Automated chromatography was performed on a Biotage Isolera Prime system with double UV detection; Biotage SFÄR cartridges were employed. UPLC purifications were performed with a Dionex Ultimate 3000 equipped with a Dionex RS Variable Wavelength Detector (column: Atlantis Prep T3 OBDTM 5 μm 19 × 100 mm, flow 15 mL min^–1^ unless otherwise stated) and was used at a flow rate of 10.0 mL/min. After lyophilization, the final compounds were isolated either as a salt or as neutral molecules (see Table SI1). Both a Jasco LC-4000 equipped with a C18 cartridge (Phenomenex Luna, 100 Å, 5 μm, 4.6 mm × 150 mm; flow rate 1 mL/min) and a Waters (515 HPLC Pump and 996 PDA) equipped with a C18 cartridge (Atlantis T3, 5 μm, 4.6 mm × 100 mm; flow rate 1 mL/min) were employed for analytical HPLC. The purity of all tested compounds (**2–11**) was assessed by HPLC and found to be ≥95%. NMR experiments were recorded on a Bruker Avance 400 MHz instrument at 298 K (unless otherwise stated). Chemical shifts (δ) are expressed in ppm and are referred to internal standards (TMS). The δ (ppm) axis has been calibrated on the solvent residual signal for which every spectrum was recorded. The signal shapes (^1^H NMR) are abbreviated as s (singlet), d (doublet), t (triplet), q (quartet), qui (quintet), sex (sextet), m (multiplet), dd (doublet of doublets), dt (doublet of triplets). COSY and HSQC experiments were used to assist the ^1^H and ^13^C resonance assignments. Mass spectra were recorded on a ThermoFisherLCQ apparatus (ESI ionization); high-resolution mass spectra (HRMS) were acquired on a Waters SYNAPT G2 Si ESI QTof instrument. **Man84** and **Man79** were obtained as described in ref 16.

### Synthesis of Alkynes **12–21**

#### *N*-(Prop-2-yn-1-yl)­pyrimidin-2-amine **(12)**

Following a procedure from Veltri et al.,^34^ 2-chloropyrimidine **23** (90 mg, 0.78 mmol, 1.0 equiv) was suspended in dry CH_3_CN (1.56 mL), then propargylamine **22** (100 μL, 1.56 mmol, 2.0 equiv) and DIPEA (407 μL, 2.34 mmol, 3.0 equiv) were added. The reaction was stirred at reflux for 16 h. Then, the solvent was evaporated. The crude was purified by automated flash chromatography (Hex/AcOEt gradient from 0% to 60% AcOEt) leading to **12** as a white solid (20 mg, 22%). Analytical data were found to be in agreement with the reported ones.^34^ *R*_f_ (**12**): 0.26 in Hex/AcOEt (6:4). ^1^H NMR (400 MHz, CDCl_3_): δ­(ppm) = 8.33 (d, 2H, *J* = 4.8 Hz, **H**_**Ar**_-4,6), 6.61 (t, 1H, *J* = 4.8 Hz, **H**_**Ar**_-5), 5.37 (br s, 1H, N**H**), 4.23 (dd, 2H, *J* = 5.8 Hz, *J* = 2.5 Hz, -C**H**_**2**_-C≡CH), 2.22 (t, 1H, *J* = 2.5 Hz, ≡C**H**). ^13^C NMR (400 MHz, CDCl_3_): δ­(ppm) extrapolated from HSQC = 158.1 (**C**_**Ar**_-4,6), 111.0 (**C**_**Ar**_-5), 71.1 (≡**C**H), 30.9 (-**C**H_2_–C≡CH). MS (ESI): *m*/*z* calculated for [C_7_H_8_N_3_]^+^: 134.07 [M + H]^+^, found: 134.08.

#### 4,6-Dimethoxy-*N*-(prop-2-yn-1-yl)-1,3,5-triazin-2-amine **(13)**

2-chloro-4,6-dimethoxytriazine **24** (200 mg, 1.14 mmol) and DIPEA (229 μL, 1.37 mmol, 1.2 equiv) were suspended in THF (3.8 mL) and stirred for 10 min at room temperature under N_2_ atmosphere. Propargylamine **22** (87 μL, 1.37 mmol, 1.2 equiv) was added dropwise to the suspension and the resulting mixture stirred at the same temperature. After 1h, the solvent was removed under vacuum. The crude was purified by automated flash chromatography (Hex/AcOEt gradient from 30% to 100% AcOEt) giving **13** as a white solid (90 mg, 0.46 mmol, 41%). *R*_f_ (**13**): 0.45 in Hex/AcOEt (1:1). ^1^H NMR (400 MHz, CDCl_3_): δ­(ppm) = 5.92 (br s, 1H, N**H**), 4.25 (dd, 2H, *J* = 5.7 Hz, *J* = 2.4 Hz, -C**H**_**2**_-C≡CH), 4.00 (s, 3H, O**Me**), 3.96 (s, 3H, O**Me**), 2.25 (t, 1H, *J* = 2.4 Hz, ≡C**H**). ^13^C NMR (400 MHz, CDCl_3_): δ­(ppm) extrapolated from HSQC = 72.4 (≡**C**H), 55.2 (O**Me**), 30.6 (-**C**H_2_–C≡CH). MS (ESI): *m*/*z* calculated for [C_8_H_11_N_4_O_2_]^+^: 195.08 [M + H]^+^, found, 195.13.

#### *N*-(Prop-2-yn-1-yl)-4,5-dihydro-1H-imidazol-2-amine **(14)**

Following a procedure from Yoshida et al.,^35^ CH_3_I (0.585 mL, 9.40 mmol, 1.2 equiv) was added dropwise during 5 min to a stirring suspension of ethylenthiourea **25** (800 mg, 7.83 mmol) in dry EtOH (15.7 mL) at room temperature. After stirring the reaction mixture for 6 h (^1^H NMR monitoring), the solvent and the excess of CH_3_I were removed under vacuum. The residue was washed with Et_2_O and the resulting solid was dried in vacuo for 12 h at 40 °C, to give **27** as a white solid (1.90 g, 7.78 mmol, 99%). Analytical data were found to be in agreement with the reported ones.^35^ ^1^H NMR (400 MHz, DMSO): δ­(ppm) = 9.97 (br s, 2H, N**H**), 3.86 (s, 4H, N–C**H**_**2**_–C**H**_**2**_–N), 2.62 (s, 3H, S**Me**). MS (ESI): *m*/*z* calculated for [C_4_H_9_N_2_S]^+^: 117.04 [M + H]^+^, found: 116.83. Propargylamine **22** (0.173 mL, 2.71 mmol, 1.1 equiv) was added to a stirring suspension of **27** (600 mg, 2.46 mmol) in dry THF (2.46 mL) at room temperature. The reaction mixture was stirred at 40 °C for 24 h (^1^H NMR monitoring) and then evaporated under reduced pressure. The residue was washed with Et_2_O and the resulting solid was dried in vacuo for 12 h at 40 °C, leading to **14**·**HI** as a light-orange solid (614 mg, 2.44 mmol, 99%). ^1^H NMR (400 MHz, D_2_O): δ­(ppm) = 4.06 (s, 2H, -C**H**_**2**_-C≡CH), 3.75 (s, 4H, N–C**H**_**2**_–C**H**_**2**_–N), 2.77 (s, 1H, ≡C**H**). MS (ESI): *m*/*z* calculated for [C_6_H_10_N_3_]^+^: 124.08 [M + H]^+^, found: 123.87. **14**·**HI** (300 mg, 1.20 mmol) was added to a NaOH solution (40% *aq*, 0.480 mL). CH_2_Cl_2_ (0.960 mL) was added to the mixture, which was vigorously stirred at room temperature for 15 min. The layers were separated and the aqueous phase was extracted with CH_2_Cl_2_. The combined organic layers were dried over Na_2_SO_4_, filtered and evaporated under reduced pressure. The obtained residue was dried in vacuo for 12 h at 40 °C, giving **14** as a yellow oil (146 mg, 1.18 mmol, 99%). ^1^H NMR (400 MHz, CDCl_3_): δ­(ppm) = 3.90 (d, 2H, *J* = 2.5 Hz, -C**H**_**2**_ **-**C≡CH), 3.53 (s, 4H, N–C**H**_**2**_–C**H**_**2**_–N), 2.24 (t, 1H, *J* = 2.5 Hz, ≡C**H**). ^13^C NMR (400 MHz, CDCl_3_): δ­(ppm) extrapolated from HSQC = 70.9 (≡**C**H), 45.1 (*N*–**C**H_2_–**C**H_2_–N), 34.0 (-**C**H_2_–C≡CH). MS (ESI): *m*/*z* calculated for [C_6_H_10_N_3_]^+^: 124.08 [M + H]^+^, found, 123.90.

#### *N*-(Prop-2-yn-1-yl)-1,4,5,6-tetrahydropyrimidin-2-amine Hydroiodide **(15**·**HI)**

Following a procedure from Aoyagi et al.,^36^ CH_3_I (0.321 mL, 5.16 mmol, 1.2 equiv) was added dropwise over 5 min to a stirring suspension of trimethylenthiourea **26** (500 mg, 4.30 mmol) in dry EtOH (4.30 mL) at room temperature. After stirring the reaction mixture for 12 h (^1^H NMR monitoring), the solvent and the excess of CH_3_I were removed under vacuum. The residue was washed with Et_2_O and dried in vacuo for 12 h at 40 °C, to give **28** as a white solid (1.09 g, 4.29 mmol, 99%). Analytical data were found to be in agreement with the reported ones.^36^ ^1^H NMR (400 MHz, DMSO): δ­(ppm) = 9.55 (br s, 2H, N**H**), 3.38 (t, 4H, *J* = 5.8 Hz, N–C**H**_**2**_–CH_2_–C**H**_**2**_–N), 2.58 (s, 3H, S**Me**), 1.90 (qui, 2H, *J* = 5.8 Hz, N–CH_2_–C**H**_**2**_–CH_2_–N). MS (ESI): *m*/*z* calculated for [C_5_H_11_N_2_S]^+^: 131.06 [M + H]^+^, found: 131.12. Propargylamine **22** (0.149 mL, 2.33 mmol, 1.2 equiv) was added to a stirring suspension of **28** (500 mg, 1.94 mmol) in dry THF (1.94 mL) at room temperature The reaction mixture was stirred at 60 °C for 120 h and monitored by ^1^H NMR analysis. After total consumption of the starting material (^1^H NMR monitoring), the mixture was evaporated under reduced pressure. The residue was washed with Et_2_O and the resulting solid was dried in vacuo for 12 h at 40 °C, leading to **15**·**HI** as a light-orange solid (459 mg, 1.73 mmol, 89%). Analytical data were found to be in agreement with the reported ones.^36 1^H NMR (400 MHz, CD_3_OD): δ­(ppm) = 3.98 (d, 2H, *J* = 2.5 Hz, -C**H**_**2**_-C≡CH), 3.38 (t, 4H, *J* = 5.7 Hz, N–C**H**_**2**_–CH_2_–C**H**_**2**_–N), 2.83 (t, 1H, *J* = 2.5 Hz, ≡C**H**), 1.97 (qui, 2H, *J* = 5.7 Hz, N–CH_2_–C**H**_**2**_–CH_2_–N). ^13^C NMR (400 MHz, CD_3_OD): δ­(ppm) extrapolated from HSQC = 73.1 (≡**C**H), 38.1 (*N*–**C**H_2_–CH_2_–**C**H_2_–N), 29.2 (-**C**H_2_–C≡CH), 20.0 (N–CH_2_–**C**H_2_–CH_2_–N). MS (ESI): *m*/*z* calculated for [C_7_H_12_N_3_]^+^: 138.10 [M + H]^+^, found: 138.07.

#### 4-Chloro-*N*-(prop-2-yn-1-yl)­pyrimidin-2-amine (16) and 2-chloro-*N*-(prop-2-yn-1-yl)­pyrimidin-4-amine **(17)**

Uracil **29** (400 mg, 3.57 mmol) in POCl_3_ (8.34 mL, 89.25 mmol, 25 equiv) was heated at 120 °C for 2 h. The reaction was stopped, cooled to room temperature and excess POCl_3_ was removed under vacuum. before adding iced water. The reaction mixture was extracted twice with AcOEt and the combined organic extracts were washed with brine. The organic phase was dried over Na_2_SO_4_ and the solvent was removed under reduced pressure. The crude was purified by automated flash chromatography (Hex/AcOEt gradient from 10% to 100%) to obtain **30** as a white solid (349 mg, 2.34 mmol, 66%). Analytical data were found to be in agreement with the reported ones.^37^ *R*_f_ (**30**): 0.36 in Hex/AcOEt (8:2). ^1^H NMR (400 MHz, CDCl_3_): δ­(ppm) = 8.53 (d, 1H, *J* = 5.4 Hz, **H**_**Ar**_-6), 7.33 (d, 1H, *J* = 5.4 Hz, **H**_**Ar**_-5). MS (ESI): *m*/*z* calculated for [C_4_H_3_Cl_2_N_2_]^+^: 148.96 [M + H]^+^, found: 149.01. Following a procedure by Wright et al.,^38^ 2,4-dichloropyrimidine **30** (300 mg, 2.01 mmol) was dissolved in CH_3_CN (8.04 mL). Propargylamine **22** (0.134 mL, 2.11 mmol, 1.05 equiv) and DIPEA (1.4 mL, 8.04 mmol, 4 equiv) were added dropwise to the solution at 0 °C. The reaction mixture was allowed to warm to room temperature and stirred for 23 h. The solvent was evaporated under vacuum and the crude was purified by flash chromatography (Hex/AcOEt gradient from 15% to 60%), giving **17** (203 mg, 1.21 mmol, 60%) and **16** (36 mg, 0.21 mmol, 11%), both as foamy white solids. **16**: *R*_f_ (**16**): 0.33 in Hex/AcOEt (8:2). ^1^H NMR (400 MHz, CDCl_3_): δ­(ppm) = 8.20 (d, 1H, *J* = 5.1 Hz, **H**_**Ar**_-6), 6.63 (d, 1H, *J* = 5.1 Hz, **H**_**Ar**_-5), 5.86 (br s, 1H, N**H**), 4.23 (dd, 2H, *J* = 5.8 Hz, *J* = 2.5 Hz, -C**H**_**2**_-C≡CH), 2.23 (t, 1H, *J* = 2.5 Hz, ≡C**H**). ^13^C NMR (400 MHz, CDCl_3_): δ­(ppm) extrapolated from HSQC = 159.5 (**C**_**Ar**_-6), 111.2 (**C**_**Ar**_-5), 71.3 (≡**C**H), 31.7 (-**C**H_2_–C≡CH). MS (ESI): *m*/*z* calculated for [C_7_H_7_ClN_3_]^+^: 168.03 [M + H]^+^, found: 168.30. **17:** *R*_f_ (**17**): 0.09 in Hex/AcOEt (8:2). ^1^H NMR (400 MHz, CDCl_3_): δ­(ppm) = 8.09 (d, 1H, *J* = 5.6 Hz, **H**_**Ar**_-6), 6.37 (d, 1H, *J* = 5.6 Hz, **H**_**Ar**_-5), 5.81 (br s, 1H, N**H**), 4.23 (s, 2H, -C**H**_**2**_-C≡CH), 2.28 (t, 1H, *J* = 2.4 Hz, ≡C**H**). ^13^C NMR (400 MHz, CDCl_3_): δ­(ppm) extrapolated from HSQC = 156.8 (**C**_**Ar**_-6), 102.6 (**C**_**Ar**_-5), 72.3 (≡**C**H), 30.9 (-**C**H_2_–C≡CH). MS (ESI): *m*/*z* calculated for [C_7_H_7_ClN_3_]^+^: 168.03 [M + H]^+^, found: 168.24.

#### *tert*-Butyl prop-2-yn-1-yl­(pyridin-2-yl)­carbamate **(18)**

Following a procedure from Jeong et al.,^39^ 2-aminopyridine **31** (300 mg, 3.2 mmol, freshly crystallized from CHCl_3_/petroleum ether), Et_3_N (1.16 mL, 8.32 mmol, 2.6 equiv), and Boc_2_O (1.04 g, 4.8 mmol, 1.5 equiv) were dissolved in CH_2_Cl_2_ (10.7 mL) and stirred at room temperature and under N_2_ atmosphere. After 14 h, Boc_2_O (698 mg, 3.2 mmol, 1 equiv) and Et_3_N (0.89 mL, 6.4 mmol, 2 equiv) were added and the reaction mixture was stirred for additional 24 h. Upon completion (TLC monitoring), the mixture was washed with saturated aqueous NaHCO_3_ and brine, and the phases were separated. The organic layer was dried over Na_2_SO_4_, filtered and evaporated under vacuum. The crude was purified by flash chromatography (Hex/AcOEt gradient from 0% to 15%), leading to **35** as a white solid (473 mg, 2.44 mmol, 76%). Analytical data were found to be in agreement with the reported ones.^39^ *R*_f_ (**35**): 0.47 in Hex/AcOEt (9:1). ^1^H NMR (400 MHz, CDCl_3_): δ­(ppm) = 8.26 (ddd, 1H, *J* = 5.0 Hz, *J* = 2.1 Hz, *J* = 1.0 Hz, **H**_**Ar**_-6), 7.98 (br s, 1H, N**H**), 7.94 (d, *J* = 8.6 Hz, **H**_**Ar**_-3), 7.65 (ddd, 1H, *J* = 8.6 Hz, *J* = 7.3 Hz, *J* = 2.1 Hz, **H**_**Ar**_-4), 6.94 (ddd, 1H, *J* = 7.3, *J* = 5.0 Hz, *J* = 1.0 Hz, **H**_**Ar**_-5), 1.53 (s, 9H, *t* **Bu**). MS (ESI): *m*/*z* calculated for [C_10_H_15_N_2_O_2_]^+^: 195.11 [M + H]^+^, found: 195.35. NaH (90%, 50 mg, 2.06 mmol, 2 equiv) was suspended in anhydrous DMF (2.0 mL) under N_2_ atmosphere, and cooled to 0 °C. Then, a solution of **35** (200 mg, 1.03 mmol) in DMF (4.9 mL) was added and the reaction mixture was vigorously stirred for 30 min at the same temperature. Propargyl bromide **39** (80% solution in toluene, 141 μL, 1.85 mmol, 1.8 equiv) was added dropwise, the mixture was warmed to room temperature, and stirred for 16 h. Upon completion (TLC monitoring), the reaction was quenched with saturated aqueous NaHCO_3_ and extracted three times with AcOEt. The combined organic layers were dried over anhydrous Na_2_SO_4_, filtered and concentrated under vacuum. The crude was purified by automated flash column chromatography (Hex/AcOEt gradient from 5% to 6%), leading to **18** as a white solid (188 mg, 0.81 mmol, 79%). Analytical data were found to be in agreement with the reported ones.^39^ *R*_f_ (**18**): 0.41 in Hex/AcoEt (9:1). ^1^H NMR (400 MHz, CDCl_3_): δ­(ppm) = 8.41 (ddd, 1H, *J* = 5.0 Hz, *J* = 2.1 Hz, *J* = 0.9 Hz, **H**_**Ar**_-6), 7.70 (d, *J* = 8.3 Hz, **H**_**Ar**_-3), 7.64 (ddd, 1H, *J* = 8.3 Hz, *J* = 7.0 Hz, *J* = 1.9 Hz, **H**_**Ar**_-4), 7.02 (ddd, 1H, *J* = 7.0, *J* = 5.0 Hz, *J* = 1.2 Hz, **H**_**Ar**_-5), 4.75 (d, 2H, *J* = 2.4 Hz, -C**H**_**2**_-C≡CH), 2.15 (t, 1H, *J* = 2.4 Hz, ≡C**H**), 1.54 (s, 9H, *t* **Bu**). ^13^C NMR (400 MHz, CDCl_3_): δ­(ppm) extrapolated from HSQC = 147.5 (**C**_**Ar**_-6), 137.0 (**C**_**Ar**_-4), 119.7 (**C**_**Ar**_-5), 118.9 (**C**_**Ar**_-3), 70.7 (≡**C**H), 36.6 (-**C**H_2_–C≡CH), 28.3 (*t* **Bu**). MS (ESI): *m*/*z* calculated for [C_13_H_16_N_2_O_2_Na]^+^: 255.12 [M + Na]^+^, found: 255.32.

#### *tert*-Butyl 2-((*tert*-butoxycarbonyl)­(prop-2-yn-1-yl)­amino)-1*H*-imidazole-1-carboxylate **(19)**

Following a procedure from Jeong et al.,^39^ 2-aminoimidazole **32** (400 mg, 3.0 mmol) was dissolved in NaOH (2 M *aq*, 4.5 mL, 9.1 mmol, 3 equiv) and stirred at room temperature for 10 min. Then, a solution of Boc_2_O (1.06 g, 4.9 mmol, 1.6 equiv) in anhydrous CH_2_Cl_2_ (6.6 mL) was added and the reaction mixture was stirred at the same temperature. After 24 h, Boc_2_O (659 mg, 3.0 mmol, 1 equiv) and NaOH (2 M *aq*, 3.1 mL, 6.1 mmol, 2 equiv) were added and the reaction mixture was warmed to 30 °C. Upon completion of the reaction (TLC monitoring after 24 h), the two phases were separated. The organic layer was washed with water, dried over anhydrous Na_2_SO_4_, filtered and concentrated under vacuum. The crude was purified by automated flash chromatography (Hex/AcOEt gradient from 10% to 100%), leading to **36** as a white solid (691 mg, 2.4 mmol, 76%). Analytical data were found to be in agreement with the reported ones.^39^ *R*_f_ (**36**): 0.43 in Hex/AcOEt (1:1). ^1^H NMR (400 MHz, CDCl_3_): δ­(ppm) = 9.04 (s, 1H, N**H**), 6.97 (d, 1H, *J* = 1.8 Hz, **H**_**Ar**_-5), 6.77 (d, 1H, *J* = 1.8 Hz, **H**_**Ar**_-4), 1.60 (s, 9H, *t* **Bu**), 1.52 (s, 9H, *t* **Bu**). MS (ESI): *m*/*z* calculated for [C_26_H_42_N_6_O_8_Na]^+^: 589.28 [2M + Na]^+^, found: 588.75. NaH (90%, 27 mg, 1.11 mmol) was suspended in anhydrous DMF (1.1 mL) under N_2_ atmosphere, and cooled to 0 °C. Then, a solution of **36** (150 mg, 0.52 mmol) in DMF (2.6 mL) was added and the reaction mixture was vigorously stirred for 30 min at the same temperature. Propargyl bromide **39** (80% in toluene, 94 μL, 0.99 mmol, 1.8 equiv) was added dropwise, warmed to room temperature and stirred for 16 h. Upon completion (TLC monitoring), the reaction was quenched with saturated aqueous NaHCO_3_ and extracted three times with AcOEt. The combined organic layers were dried over anhydrous Na_2_SO_4_, filtered and concentrated under vacuum. The obtained crude was purified by automated flash column chromatography (Hex/AcOEt gradient from 0% to 100%), leading to the product as a white solid (108 mg, 0.34 mmol, 65%). *R*_f_ (**19**): 0.42 in Hex/AcOEt (1:1). Analytical data were found to be in agreement with the reported ones.^39^ ^1^H NMR (400 MHz, CDCl_3_): δ­(ppm) = 7.31 (s, 1H, **H**_**Ar**_-5), 6.90 (s, 1H, **H**_**Ar**_-4), 4.46 (m, 2H, -C**H**_**2**_-C≡CH), 2.19 (s, 1H, ≡C**H**), 1.60 (s, 9H, *t* **Bu**), 1.36 (s, 9H, *t* **Bu**). ^13^C NMR (400 MHz, CDCl_3_): δ­(ppm) extrapolated from HSQC = 126.5 (**C**_**Ar**_-5), 117.7 (**C**_**Ar**_-4), 72.6 (≡**C**H), 38.8 (-**C**H_2_–C≡CH), 28.4 (*t* **Bu**). MS (ESI): *m*/*z* calculated for [C_16_H_24_N_3_O_4_]^+^: 322.17 [M + H]^+^, found: 322.30.

#### *tert*-Butyl-2-((*tert*-butoxycarbonyl)­(prop-2-yn-1-yl)­amino)-1*H*-benzo­[*d*]­imidazole-1-carboxylate **(20)**

Following a procedure from Jeong et al.,^39^ 2-Aminobenzimidazole **33** (400 mg, 3 mmol), Et_3_N (1.54 mL, 11 mmol, 3.7 equiv), and Boc_2_O (1.31 g, 6 mmol, 2 equiv) were dissolved in CH_2_Cl_2_ (10 mL) and stirred at room temperature, under N_2_ atmosphere. After 16 h, Boc_2_O (1.31 g, 6 mmol, 2 equiv) and ET_3_N (1.54 mL, 11 mmol, 3.7 equiv) were added and the reaction mixture was stirred for 24 h more. The reaction mixture was washed with saturated NaHCO_3_ and brine, and the phases were separated. The organic layer was dried over Na_2_SO_4_, filtered and evaporated under vacuum to afford **37** as a white solid (759 mg, 2.28 mmol, 76% yield). Analytical data were found to be in agreement with the reported ones.^39^ *R*_f_ (**37**): 0.47 in Hex/AcoEt (7:3). ^1^H NMR (400 MHz, CDCl_3_): δ­(ppm) = 9.81 (br s, 1H, N**H**), 7.68 (d, 1H, *J* = 4.2 Hz, **H**_**Ar**_-7), 7.66 (d, 1H, *J* = 4.2 Hz, **H**_**Ar**_-4), 7.27 (t, 1H, *J* = 7.5 Hz, **H**_**Ar**_-6), 7.19 (t, 1H, *J* = 7.5 Hz, **H**_**Ar**_-5), 1.73 (s, 9H, *t* **Bu**), 1.55 (s, 9H, *t* **Bu**). MS (ESI): *m*/*z* calculated for [C_17_H_24_N_3_O_4_]^+^: 334.17 [M + H]^+^, found: 333.94. NaH (90%, 29 mg, 1.2 mmol) was suspended in anhydrous DMF (1.1 mL) under N_2_ atmosphere, and cooled to 0 °C. A solution of **37** (200 mg, 0.60 mmol) in DMF (2.9 mL) was added, and the reaction mixture was stirred vigorously for 30 min at the same temperature. Propargyl bromide **39** (80% in toluene, 103 μL, 1.08 mmol) was added dropwise, the solution was warmed to room temperature, and stirred for 16 h. Upon completion (TLC monitoring), the reaction was quenched with saturated aqueous NaHCO_3_ and extracted three times with AcOEt. The combined organic layers were dried over anhydrous Na_2_SO_4_, filtered, and concentrated under vacuum. The obtained crude was purified by automated flash column chromatography (Hex/AcOEt gradient from 0% to 60%), leading to **20** as a white solid (170 mg, 0.46 mmol, 72%). Analytical data were found to be in agreement with the reported ones.^39^ *R*_f_ (**20**): 0.67 in Hex/AcOEt (7:3). ^1^H NMR (400 MHz, CD_3_OD): δ (ppm) = 7.99 (s, 1H, **H**_**Ar**_-7), 7.66 (d, 1H, *J* = 7.6 Hz, **H**_**Ar**_-4), 7.41 (q, 2H, *J* = 9.3 Hz, *J* = 7.6 Hz, **H**_**Ar**_-5,6), 4.60–4.47 (m, 2H, -C**H**_**2**_-C≡CH), 2.64 (s, 1H, ≡C**H**), 1.70 (s, 9H, *t* **Bu**), 1.36 (s, 9H, *t* **Bu**). ^13^C NMR (400 MHz, CD_3_OD): δ­(ppm) extrapolated from HSQC = 124.8 (**C**_**Ar**_-5,6), 119.4 (**C**_**Ar**_-4), 114.8 (**C**_**Ar**_-7), 73.4 (≡**C**H), 37.1 (-**C**H_2_–C≡CH), 26.1 (*t* **Bu**). MS (ESI): *m*/*z* calculated for [C_20_H_26_N_3_O_4_]^+^: 371.18 [M + H]^+^, found: 371.73.

#### *tert*-Butyl-2-((*tert*-butoxycarbonyl)­(prop-2-yn-1-yl)­amino)-5-methoxy-1H-benzo­[d]­imidazole-1-carboxylate **(21)**

Following a procedure from Jeong et al.,^39^ 5-methoxy-2-aminobenzimidazole **34** (108 mg, 0.41 mmol), Et_3_N (0.212 mL, 1.52 mmol, 3.7 equiv), and Boc_2_O (225 mg, 1.03 mmol, 2.5 equiv) were dissolved in CH_2_Cl_2_ (1.4 mL) and stirred at room temperature and under N_2_ atmosphere for 16 h. The reaction mixture was washed with saturated NaHCO_3_ and brine, and the phases were separated. The organic layer was dried over Na_2_SO_4_, filtered and evaporated under vacuum. The obtained crude was purified by flash chromatography (Hex/AcOEt (8:2) to remove apolar impurities and CH_2_Cl_2_ with Et_2_O gradient from 10 to 20% to recover the product), leading to **38** (mixture of two regioisomers) as a white solid (103 mg, 0.28 mmol, 70%). *R*_f_ (**38**): 0.23 in Hex/AcoEt (7:3), 0.65 in CH_2_Cl_2_:Et_2_O (9:1). ^1^H NMR (400 MHz, CDCl_3_, major regioisomer): δ­(ppm) = 9.78 (br s, 1H, N**H**), 7.52 (d, 1H, *J* = 8.9 Hz, **H**_**Ar**_-7), 7.22 (d, 1H, *J* = 2.5 Hz, **H**_**Ar**_-4), 6.77 (dd, 1H, *J* = 8.9 Hz, *J* = 2.5 Hz, **H**_**Ar**_-6), 3.82 (s, 3H, O**Me**), 1.72 (s, 9H, *t* **Bu**), 1.56 (s, 9H, *t* **Bu**). ^1^H NMR (400 MHz, CDCl_3_, minor regioisomer): δ­(ppm) = 9.69 (br s, 1H, N**H**), 7.55 (d, 1H, *J* = 8.9 Hz, **H**_**Ar**_-7), 7.26 (m, 1H, **H**_**Ar**_-4), 6.88 (dd, 1H, *J* = 8.9 Hz, *J* = 2.5 Hz, **H**_**Ar**_-6), 3.83 (s, 3H, O**Me**), 1.74 (s, 9H, *t* **Bu**), 1.55 (s, 9H, *t* **Bu**). MS (ESI): *m*/*z* calculated for [C_18_H_25_N_3_O_5_]^+^: 363.18 [M + H]^+^, found: 363.77. NaH (90%, 13 mg, 0.55 mmol, 2 equiv) was suspended in anhydrous DMF (0.5 mL) under N_2_ atmosphere, and cooled to 0 °C. Then, a solution of **38** (100 mg, 0.28 mmol, 1 equiv) in DMF (1.4 mL) was added, and the reaction mixture was vigorously stirred for 30 min at the same temperature. Propargyl bromide **39** (80% in toluene, 48 μL, 0.50 mmol, 1.8 equiv) was added dropwise, the solution was warmed to room temperature, and stirred for 16 h. Upon completion (TLC monitoring), the reaction was quenched with saturated aqueous NaHCO_3_ and extracted three times with AcOEt. The combined organic layers were dried over anhydrous Na_2_SO_4_, filtered, and concentrated under vacuum. The obtained crude was purified by flash chromatography (CH_2_Cl_2_/Et_2_O gradient from 2 to 10%), leading to **21** (mixture of two regioisomers) as a white solid (52 mg, 0.13 mmol, 47%). *R*_f_ (**21**): 0.51 in CH_2_Cl_2_:Et_2_O (95:5). ^1^H NMR (400 MHz, CDCl_3_, major regioisomer) δ (ppm) = 7.81 (s, 1H, **H**_**Ar**_-7), 7.21 (d, 1H, *J* = 2.0 Hz, **H**_**Ar**_-4), 6.97 (m, 1H, **H**_**Ar**_-6), 4.66–4.50 (m, 2H, -C**H**_**2**_-C≡CH), 3.86 (s, 3H, O**Me**), 2.18 (s, 1H, ≡C**H**), 1.67 (s, 9H, *t* **Bu**), 1.38 (s, 9H, *t* **Bu**). ^13^C NMR (400 MHz, CDCl_3_, major regioisomer): δ­(ppm) extrapolated from HSQC = 116.0 (**C**_**Ar**_-7), 114.1 (**C**_**Ar**_-6), 102.8 (**C**_**Ar**_-4), 72.6 (≡**C**H), 55.5 (O**Me**), 38.1 (-**C**H_2_–C≡CH), 28.4 (*t* **Bu**). ^1^H NMR (400 MHz, CDCl_3_, minor regioisomer) δ (ppm) = 7.60 (d, 1H, *J*_3–4_ = 9.2 Hz, **H**_**Ar**_-7), 7.55–7.49 (m, 1H, **H**_**Ar**_-4), 6.97 (m, 1H, **H**_**Ar**_-6), 4.66–4.50 (m, 2H, -C**H**_**2**_-C≡CH), 3.88 (s, 3H, O**Me**), 2.18 (s, 1H, ≡C**H**), 1.67 (s, 9H, *t* **Bu**), 1.38 (s, 9H, *t* **Bu**). ^13^C NMR (400 MHz, CDCl_3_, minor regioisomer): δ­(ppm) extrapolated from HSQC = 120.2 (**C**_**Ar**_-7), 114.1 (**C**_**Ar**_-6), 98.9 (**C**_**Ar**_-4), 72.6 (≡**C**H), 55.5 (O**Me**), 38.1 (-**C**H_2_–C≡CH), 28.4 (*t* **Bu**). MS (ESI): *m*/*z* calculated for [C_21_H_27_N_3_O_5_]^+^: 401.20 [M + H]^+^, found: 401.87.

### General Procedure for the Synthesis of **40**, **41**, **44–49** (CuAAC reaction)

A 0.4 M solution of the alkyne and a 0.4 M solution of the azide **1** were prepared in deoxygenated HPLC-grade THF. A 0.04 M CuSO_4_·5H_2_O solution and a 0.16 M Na-ascorbate solution were prepared in deoxygenated HPLC-grade water. To the alkyne solution (1 equiv) were added: CuSO_4_·5H_2_O solution (0.1 equiv), Na-ascorbate solution (0.4 equiv) and azide solution (1 equiv). The reaction was stirred at room temperature and protected from light. Upon completion (TLC analysis), the solvents were removed under vacuum and the crude was purified by flash chromatography.

#### 2-Chloroethyl 3,4,6-Tri-*O*-acetyl-2-deoxy-2-(4-pyrimidin-2-ylamino)­methyl)-1,2,3-triazol-1-yl)-α-*D*-mannopyranoside **(40)**

**40** was synthesized from **1** (59 mg, 0.15 mmol, 1 equiv) and **12** (20 mg, 0.15 mmol, 1 equiv) according to the general procedure for CuAAC reaction and purified by flash chromatography (Hex/AcOEt gradient from 50% to 90%; white foam, 52 mg, 0.10 mmol, 67%). *R*_f_ (**40**): 0.23 in Hex/AcOEt (3:7). ^1^H NMR (400 MHz, CDCl_3_): δ­(ppm) = 8.29 (d, 2H, *J* = 4.8 Hz, **H**_**Ar**_-4,6), 8.00 (s, 1H, **H**_**TrCH**_), 6.57 (t, 1H, *J* = 4.8 Hz, **H**_**Ar**_-5), 5.71 (t, 1H, *J* = 6.5 Hz, N**H**), 5.50 (dd, 1H, *J* = 9.8 Hz, *J* = 5.2 Hz, **H**-3), 5.41 (dd, 1H, *J* = 5.2 Hz, *J*_1,2_ = 1.1 Hz, **H**-2), 5.26 (t, 1H, *J*_3,4_ = *J*_4,5_ = 9.8 Hz, **H**-4), 5.11 (br s, 1H, **H**-1), 4.77 (ddd, 2H, *J*_*gem*_ = 14.7 Hz, *J* = 6.5 Hz, -C**H**_**2**_-N), 4.30–4.17 (m, 3H, **H**-5,6), 4.00–3.93 (m, 1H, -OC**H**_**2**_-CH_2_Cl), 3.89–3.82 (m, 1H, -OC**H**_**2**_-CH_2_Cl), 3.71 (t, 2H, *J* = 5.1 Hz, –OCH_2_–C**H**_**2**_Cl), 2.13 (s, 3H, O**Ac**), 2.04 (s, 3H, O**Ac**), 1.92 (s, 3H, O**Ac**). ^13^C NMR (400 MHz, CDCl_3_): δ­(ppm) extrapolated from HSQC = 158.1 (**C**_**Ar**_-4,6), 121.9 (**C**_**TrCH**_), 111.2 (**C**_**Ar**_-5), 97.8 (**C**-1), 69.5 (**C**-5), 68.8 (-O**C**H_**2**_-CH_2_Cl), 68.1 (**C**-3), 64.6 (**C**-4), 61.6 (**C**-6), 60.1 (**C**-2), 42.3 (−OCH_**2**_–**C**H_2_Cl), 37.0 (-**C**H_**2**_-N), 20.9 (O**Ac**), 20.8 (O**Ac**), 20.7 (O**Ac**). MS (ESI): *m*/*z* calculated for [C_21_H_28_ClN_6_O_8_]^+^: 527.17 [M + H]^+^, found: 527.17.

#### 2-Chloroethyl 3,4,6-Tri-*O*-acetyl-2-deoxy-2-(4-(((4,6-dimethoxy-1,3,5-triazin-2-yl)­amino)­methyl)-1,2,3-triazol-1-yl)-α-D-mannopyranoside **(41)**

**41** was synthesized from **1** (181 mg, 0.46 mmol, 1 equiv) and **13** (90 mg, 0.46 mmol, 1 equiv) according to general procedure for CuAAC reaction and purified via flash chromatography (Hex/AcOEt gradient from 50% to 90%; white foam, 229 mg, 0.39 mmol, 85%). *R*_f_ (**41**): 0.51 in AcOEt. ^1^H NMR (400 MHz, CD_3_OD): δ­(ppm) = 8.14 (s, 1H, **H**_**TrCH**_), 5.48 (dd, 1H, *J* = 10.1 Hz, *J* = 5.2 Hz, **H**-3), 5.40 (dd, 1H, *J* = 5.2 Hz, *J* = 1.1 Hz, **H**-2), 5.29 (t, 1H, *J* = 10.1 Hz, **H**-4), 5.27 (br s, 1H, **H**-1), 4.72 (dd, 2H, *J* = 15.8 Hz, *J* = 2.7 Hz, -C**H**_**2**_-N), 4.39–4.30 (m, 2H, **H**-5,6a), 4.20 (d, 1H, *J* = 10.2 Hz, **H**-6b), 4.06–3.98 (m, 1H, -OC**H**_**2**_-CH_2_Cl), 3.97–3.87 (m, 7H, -OC**H**_**2**_-CH_2_Cl, 2xO**Me**), 3.79 (*t*, 2H, *J* = 5.2 Hz, –OCH_**2**_-C**H**_**2**_Cl), 2.08 (s, 3H, O**Ac**), 2.03 (s, 3H, O**Ac**), 1.87 (s, 3H, O**Ac**). ^13^C NMR (400 MHz, CDCl_3_): δ­(ppm) extrapolated from HSQC = 123.5 (**C**_**TrCH**_), 98.4 (**C**-1), 70.0 (**C**-5), 69.6 (-O**C**H_**2**_-CH_2_Cl), 69.5 (**C**-3), 65.5 (**C**-4), 62.6 (**C**-6), 61.2 (**C**-2), 54.8 (O**Me**), 43.3 (−OCH_**2**_–**C**H_2_Cl), 37.2 (-**C**H_**2**_-N), 20.4 (O**Ac**), 20.0 (O**Ac**), 19.7 (O**Ac**). MS (ESI): *m*/*z* calculated for [C_22_H_31_ClN_7_O_10_]^+^: 588.18 [M + Na]^+^, found: 588.03.

#### 2-Chloroethyl 3,4,6-Tri-*O*-acetyl-2-deoxy-2-(4-(((4-chloropyrimidin-2-yl)­amino)­methyl)-1,2,3-triazol-1-yl)-α-D-mannopyranoside **(44)**

**44** was synthesized from **1** (83 mg, 0.21 mmol, 1 equiv) and **16** (36 mg, 0.21 mmol, 1 equiv) according to the general procedure for CuAAC reaction and purified via flash chromatography (Hex/AcOEt gradient from 40% to 60%; white foam, 101 mg, 0.18 mmol, 86%). *R*_f_ (**44**): 0.49 in Hex/AcOEt (3:7). ^1^H NMR (400 MHz, CD_3_OD): δ­(ppm) = 8.18 (d, 1H, *J* = 5.2 Hz, **H**_**Ar**_-6), 8.16 (s, 1H, **H**_**TrCH**_), 6.67 (d, 1H, *J* = 5.2 Hz, **H**_**Ar**_-5), 5.48 (dd, 1H, *J* = 10.1 Hz, *J* = 5.1 Hz, **H**-3), 5.40 (dd, 1H, *J* = 5.1 Hz, *J* = 1.1 Hz, **H**-2), 5.31 (t, 1H, *J* = 10.1 Hz, **H**-4), 5.28 (d, 1H, *J* = 1.1 Hz, **H**-1), 4.75–4.64 (dd, 2H, *J* = 15.7 Hz, *J* = 2.3 Hz, -C**H**_**2**_-N), 4.36–4.30 (m, 2H, **H**-5,6a), 4.22 (dd, 1H, *J* = 12.6 Hz, *J* = 2.0 Hz, **H**-6b), 4.04–3.99 (m, 1H, -OC**H**_**2**_-CH_**2**_Cl), 3.92–3.87 (m, 1H, -OC**H**_**2**_-CH_**2**_Cl), 3.79 (t, 2H, *J* = 5.1 Hz, –OCH_2_–C**H**_**2**_Cl), 2.10 (s, 3H, O**Ac**), 2.03 (s, 3H, O**Ac**), 1.87 (s, 3H, O**Ac**). ^13^C NMR (400 MHz, CD_3_OD): δ­(ppm) extrapolated from HSQC = 160.3 (**C**_**Ar**_-6), 123.5 (**C**_**TrCH**_), 110.2 (**C**_**Ar**_-5), 98.7 (**C**-1), 70.0 (**C**-5), 69.9 (**C**-3), 69.4 (-O**C**H_2_–CH_2_Cl), 65.7 (**C**-4), 62.6 (**C**-6), 61.1 (**C**-2), 43.3 (−OCH_2_–**C**H_2_Cl), 37.4 (-**C**H_**2**_-N), 20.4 (O**Ac**), 20.1 (O**Ac**), 19.9 (O**Ac**). MS (ESI): *m*/*z* calculated for [C_21_H_27_Cl_2_N_6_O_8_]^+^: 561.13 [M + H]^+^, found: 561.23; *m*/*z* calculated for [C_21_H_26_Cl_2_N_6_NaO_8_]^+^: 583.11 [M + Na]^+^, found: 583.22.

#### 2-Chloroethyl 3,4,6-Tri-*O*-acetyl-2-deoxy-2-(4-(((2-chloropyrimidin-4-yl)­amino)­methyl)-1,2,3-triazol-1-yl)-α-D-mannopyranoside **(45)**

**45** was synthesized from **1** (236 mg, 0.60 mmol, 1 equiv) and **17** (100 mg, 0.60 mmol, 1 equiv) according to the general procedure for CuAAC reaction and purified via flash chromatography (Hex/AcOEt gradient from 70 to 100%; white foam, 237 mg, 0.42 mmol, 70%). *R*_f_ (**45**): 0.49 in AcOEt. ^1^H NMR (400 MHz, CD_3_OD): δ (ppm) = 8.22 (s, 1H, **H**_**TrCH**_), 7.89 (s, 1H, **H**_**Ar**_-6), 6.37 (d, 1H, *J* = 5.2 Hz, **H**_**Ar**_-5), 5.48 (dd, 1H, *J* = 10.1 Hz, *J* = 5.3 Hz, **H**-3), 5.40 (dd, 1H, *J* = 5.3 Hz, *J* = 1.3 Hz, **H**-2), 5.31 (t, 1H, *J* = 10.1 Hz, **H**-4), 5.28 (br s, 1H, **H**-1), 4.75–4.64 (m, 2H, -C**H**_**2**_-N), 4.37–4.31 (m, 2H, **H**-5,6a), 4.22 (dd, 1H, *J* = 11.8 Hz, *J* = 1.9 Hz, **H**-6b), 4.05–3.99 (m, 1H, -OC**H**_**2**_-CH_**2**_Cl), 3.92–3.88 (m, 1H, -OC**H**_**2**_-CH_**2**_Cl), 3.79 (t, 2H, *J* = 5.2 Hz, –OCH_2_–C**H**_**2**_Cl), 2.09 (s, 3H, O**Ac**), 2.03 (s, 3H, O**Ac**), 1.88 (s, 3H, O**Ac**). ^13^C NMR (400 MHz, CD_3_OD): δ­(ppm) extrapolated from HSQC = 155.7 (**C**_**Ar**_-6), 124.5 (**C**_**TrCH**_), 106.0 (**C**_**Ar**_-5), 98.8 (**C**-1), 70.0 (**C**-5), 69.8 (**C**-3), 69.5 (-O**C**H_2_–CH_**2**_Cl), 66.0 (**C**-4), 62.8 (**C**-6), 61.3 (**C**-2), 43.3 (−OCH_2_–**C**H_**2**_Cl), 36.1 (-**C**H_**2**_-N), 20.5 (O**Ac**), 20.1 (O**Ac**), 19.9 (O**Ac**). MS (ESI): *m*/*z* calculated for [C_21_H_27_Cl_2_N_6_O_8_]^+^: 561.13 [M + H]^+^, found: 561.17; *m*/*z* calculated for [C_21_H_26_Cl_2_N_6_NaO_8_]^+^: 583.11 [M + Na]^+^, found: 583.21.

#### 2-Chloroethyl 3,4,6-Tri-*O*-acetyl-2-deoxy-2-(4-(((*tert*-butoxycarbonyl)­(pyridin-2-yl)­amino)­methyl)-1,2,3-triazol-1-yl)-α-D-mannopyranoside **(46)**

**46** was synthesized from **1** (169 mg, 0.43 mmol, 1 equiv) and **18** (100 mg, 0.43 mmol, 1 equiv) according to the general procedure for CuAAC reaction and purified via flash chromatography (Hex/AcOEt gradient from 40 to 80%; white foam, 199 mg, 0.32 mmol, 74%). *R*_f_ (**46**): 0.43 in Hex/AcOEt (3:7). ^1^H NMR (400 MHz, CD_3_OD): δ­(ppm) = 8.41 (ddd, 1H, *J* = 5.0 Hz, *J* = 2.1 Hz, *J* = 1.2 Hz, **H**_**Ar**_-6), 8.12 (s, 1H, **H**_**TrCH**_), 7.75 (ddd, 1H, *J* = 8.3 Hz, *J* = 7.9 Hz, *J* = 1.9 Hz, **H**_**Ar**_-4), 7.54 (d, *J* = 8.3 Hz, **H**_**Ar**_-3), 7.16 (ddd, 1H, *J* = 7.9, *J* = 5.0 Hz, *J* = 0.9 Hz, **H**_**Ar**_-5), 5.45 (dd, 1H, *J* = 10.1 Hz, *J* = 5.3 Hz, **H**-3), 5.39 (dd, 1H, *J* = 5.3 Hz, *J* = 1.4 Hz, **H**-2), 5.28–5.21 (m, 2H, **H**-1,4), 5.18 (s, 2H, -C**H**_**2**_-N), 4.36–4.28 (m, 2H, **H**-5,6a), 4.16 (dd, 1H, *J* = 12.0 Hz, *J* = 2.2 Hz, **H**-6b), 4.03–3.98 (m, 1H, -OC**H**_**2**_-CH_**2**_Cl), 3.92–3.87 (m, 1H, -OC**H**_**2**_-CH_**2**_Cl), 3.79 (t, 2H, *J* = 5.1 Hz, –OCH_2_–C**H**_**2**_Cl), 2.02 (s, 3H, O**Ac**), 1.94 (s, 3H, O**Ac**), 1.85 (s, 3H, O**Ac**), 1.45 (s, 9H, *t* **Bu**). ^13^C NMR (400 MHz, CD_3_OD): δ­(ppm) extrapolated from HSQC = 147.5 (**C**_**Ar**_-6), 137.6 (**C**_**Ar**_-4), 122.9 (**C**_**TrCH**_), 120.4 (**C**_**Ar**_-3), 120.2 (**C**_**Ar**_-5), 98.3 (**C**-1), 70.0 (**C**-5), 68.9 (**C**-3), 68.5 (-O**C**H_2_–CH_**2**_Cl), 64.4 (**C**-4), 61.4 (**C**-6), 60.4 (**C**-2), 42.1 (−OCH_2_–**C**H_**2**_Cl), 41.9 (-**C**H_**2**_-N), 27.7 (*t* **Bu**), 19.1 (O**Ac**), 19.0 (O**Ac**), 18.9 (O**Ac**). MS (ESI): *m*/*z* calculated for [C_27_H_37_ClN_5_O_10_]^+^: 625.22 [M + H]^+^, found: 625.03.

#### 2-Chloroethyl 3,4,6-Tri-*O*-acetyl-2-deoxy-2-(4-(((*tert*-butoxycarbonyl)­(1-(*tert*-butoxycarbonyl)-1*H*-imidazol-2-yl)­amino)­methyl)-1,2,3-triazol-1-yl)-α-D-mannopyranoside **(47)**

**47** was synthesized from **1** (134 mg, 0.34 mmol, 1 equiv) and **19** (108 mg, 0.34 mmol, 1 equiv) according to the general procedure for CuAAC reaction. Purified via flash chromatography (CH_2_Cl_2_/MeOH gradient from 0 to 6%; white foam, 164 mg, 0.23 mmol, 68%). *R*_f_ (**47**): 0.46 in CH_2_Cl_2_:MeOH (95:5). ^1^H NMR (400 MHz, CD_3_OD): δ­(ppm) = 8.24 (m, 1H, **H**_**TrCH**_), 7.45 (m, 1H, **H**_**Ar**_-5), 6.89 (m, 1H, **H**_**Ar**_-4), 5.46 (m, 1H, **H**-3), 5.39 (m, 1H, **H**-2), 5.29 (m, 1H, **H**-4), 5.25 (m, 1H, **H**-1), 4.95–4.72 (m, 2H, -C**H**_**2**_-N), 4.31 (m, 3H, **H**-5,6), 4.05–3.91 (m, 2H, -OC**H**_**2**_-CH_**2**_Cl), 3.82 (t, 2H, *J* = 6.6 Hz, –OCH_2_–C**H**_**2**_Cl), 2.19 (s, 3H, O**Ac**), 2.05 (s, 3H, O**Ac**), 1.90 (s, 3H, O**Ac**), 1.60 (s, 9H, *t* **Bu**), 1.38 (s, 9H, *t* **Bu**). ^13^C NMR (400 MHz, CD_3_OD): δ­(ppm) extrapolated from HSQC = 125.6 (**C**_**Ar**_-4), 123.6 (**C**_**TrCH**_), 118.8 (**C**_**Ar**_-5), 97.8 (**C**-1), 68.8 (**C**-5), 68.5 (**C**-3), 68.4 (-O**C**H_2_–CH_**2**_Cl), 65.1 (**C**-4), 61.7 (**C**-6), 60.2 (**C**-2), 43.9 (-**C**H_**2**_-N), 42.0 (−OCH_2_–**C**H_**2**_Cl), 27.2 (*t* **Bu**), 26.7 (*t* **Bu**) 20.1 (O**Ac**), 19.3 (O**Ac**), 19.2 (O**Ac**). MS (ESI): *m*/*z* calculated for [C_30_H_44_ClN_6_O_12_]^+^: 715.27 [M + H]^+^, found: 715.30.

#### 2-Chloroethyl 3,4,6-Tri-*O*-acetyl-2-deoxy-2-(4-(((*tert*-butoxycarbonyl)­(1-(*tert*-butoxycarbonyl)-1H-benzo­[d]­imidazol-2-yl)­amino)­methyl)-1,2,3-triazol-1-yl)-α-D-mannopyranoside **(48)**

**48** was synthesized from **1** (126 mg, 0.32 mmol, 1 equiv) and **20** (120 mg, 0.32 mmol, 1 equiv) according to the general procedure for CuAAC reaction and purified via flash chromatography (Hex/AcOEt gradient from 15 to 100%; white foam, 146 mg, 0.19 mmol, 60%). *R*_f_ (**48**): 0.20 in Hex/AcOEt (1:1). ^1^H NMR (400 MHz, CD_3_OD): δ­(ppm) = 8.28 (m, 1H, **H**_**TrCH**_), 7.94 (m, 1H, **H**_**Ar**_-4), 7.64 (d, 1H, *J* = 7.1 Hz, **H**_**Ar**_-7), 7.37 (m, 2H, **H**_**Ar**_-5,6), 5.46 (m, 1H, **H**-3), 5.39 (m, 1H, **H**-2), 5.29 (m, 1H, **H**-4), 5.18 (m, 1H, **H**-1), 5.11 (m, 2H, -C**H**_**2**_-N), 4.31 (m, 2H, **H**-6), 4.20 (m, 1H, **H**-5), 4.05–3.96 (m, 1H, -OC**H**_**2**_-CH_**2**_Cl), 3.92–3.84 (m, 1H, -OC**H**_**2**_-CH_**2**_Cl), 3.79 (t, 2H, *J* = 6.6 Hz, –OCH_2_–C**H**_**2**_Cl), 2.03 (s, 3H, O**Ac**), 1.85 (s, 3H, O**Ac**), 1.50 (s, 3H, O**Ac**), 1.71 (s, 9H, *t* **Bu**), 1.40 (s, 9H, *t* **Bu**). ^13^C NMR (400 MHz, CD_3_OD): δ­(ppm) extrapolated from HSQC = 126.1 (**C**_**Ar**_-5,6) 124.3 (**C**_**TrCH**_), 120.3 (**C**_**Ar**_-7), 115.8 (**C**_**Ar**_-4), 98.7 (**C**-1), 69.8 (**C**-5), 69.5 (-O**C**H_2_–CH_**2**_Cl), 69.4 (**C**-3), 66.1 (**C**-4), 62.5 (**C**-6), 61.5 (**C**-2), 44.8 (-**C**H_**2**_-N), 42.3 (−OCH_2_–**C**H_**2**_Cl), 27.7 (*t* **Bu**), 27.1 (*t* **Bu**), 21.1 (O**Ac**), 20.9 (O**Ac**), 20.8 (O**Ac**). MS (ESI): *m*/*z* calculated for [C_34_H_46_ClN_6_O_12_]^+^: 765.29 [M + H]^+^, found: 764.80.

#### 2-Chloroethyl 3,4,6-Tri-*O*-acetyl-2-deoxy-2-(4-(((*tert*-butoxycarbonyl)­(1-(*tert*-butoxycarbonyl)-5-methoxy-1H-benzo­[d]­imidazol-2-yl)­amino)­methyl)-1,2,3-triazol-1-yl)-α-D-mannopyranoside **(49)**

**49** was synthesized from **1** (51 mg, 0.13 mmol, 1 equiv) and **21** (52 mg, 0.13 mmol, 1 equiv) according to the general procedure for CuAAC reaction and purified via flash chromatography (Hex/AcOEt gradient from 40 to 55%; white foam, 55 mg, 0.07 mmol, 53%). *R*_f_ (**49**): 0.27 in Hex/AcOEt (1:1). ^1^H NMR (400 MHz, CD_3_OD): δ­(ppm) = 8.37–8.17 (m, 1H, **H**_**TrCH**_), 7.87–7.43 (m, 1H, **H**_**Ar**_-7), 7.23–7.07 (m, 1H, **H**_**Ar**_-4), 7.06–6.94 (m, 1H, **H**_**Ar**_-6), 5.49–5.23 (m, 3H, **H**-2,3,4), 5.19 (br s, 1H, **H**-1), 5.16–4.96 (m, 2H, -C**H**_**2**_-N), 4.37–4.15 (m, 3H, **H**-5,6), 4.06–3.94 (m, 1H, -OC**H**_**2**_-CH_**2**_Cl), 3.93–3.82 (m, 3H, -OC**H**_**2**_-CH_**2**_Cl, O**Me**), 3.79 (t, 2H, *J* = 5.1 Hz, –OCH_2_–C**H**_**2**_Cl), 2.17–1.29 (m, 30H, O**Ac**, *t* **Bu**). ^13^C NMR (400 MHz, CD_3_OD): δ­(ppm) extrapolated from HSQC = 124.0 (**C**_**TrCH**_), 115.5 (**C**_**Ar**_-7), 113.6 (**C**_**Ar**_-6), 102.1 (**C**_**Ar**_-4), 97.8 (**C**-1), 69.8 (**C**-5), 69.5 (-O**C**H_2_–CH_**2**_Cl), 69.4 (**C**-3), 64.6 (**C**-4), 61.5 (**C**-6), 60.0 (**C**-2), 54.8 (O**Me**), 43.0 (-**C**H_**2**_-N), 41.7 (−OCH_2_–**C**H_**2**_Cl), 27.4 (*t* **Bu**), 27.2 (*t* **Bu**), 20.0 (O**Ac**), 19.9 (O**Ac**), 19.1 (O**Ac**). MS (ESI): *m*/*z* calculated for [C_35_H_48_ClN_6_O_13_]^+^: 795.30 [M + H]^+^, found: 795.27.

#### 2-Chloroethyl 2-Deoxy-2-(4-imidazolin-2-ylamino)­methyl)-1,2,3-triazol-1-yl)-α-D-mannopyranoside **(4)**

A 0.4 M solution of the alkyne **14** (80 mg, 0.65 mmol, 1 equiv) and a 0.4 M solution of the azide **1** (256 mg, 0.65 mmol, 1 equiv) were prepared in deoxygenated HPLC-grade THF. A 0.04 M solution of CuSO_4_·5H_2_O (16 mg, 0.065 mmol, 0.1 equiv) and a 0.16 M solution of Na-ascorbate (52 mg, 0.26 mmol, 0.4 equiv) were prepared in deoxygenated HPLC-grade water. To the alkyne solution (1.62 mL) were added: CuSO_4_·5H_2_O solution (1.62 mL), Na-ascorbate solution (1.62 mL) and azide solution (1.62 mL). The reaction was stirred at room temperature and protected from light. Upon consumption of the starting materials (^1^H NMR monitoring, 24 h), the solvents were removed. The resulting crude material was dissolved in AcOEt and washed twice with 40% NaOH (40% *aq*). The organic phase was dried over Na_2_SO_4_, filtered and concentrated under vacuum. The resulting crude was dissolved in HCl (1 M *aq*, 700 μL), stirred for 1h and reconcentrated in vacuo. The resulting mixture of salts was purified via UPLC (flow: 10 mL/min; UV channels: 210 nm; 254 nm; A:H_2_O + 0.1% HCOOH, B: CH_3_CN. Gradient: 0–5 min: 0% B, 5–20 min: 0–50% B, 20–20.5 min: 50–100%; *t*_r_ (**4**) = 11.50 min) to obtain **4** as the formate salt. ^1^H NMR (400 MHz, CD_3_OD): δ­(ppm) = 8.54 (br s, 1H, **H**_**HCOOH**_), 8.21 (s, 1H, **H**_**CHTr**_), 5.12 (s, 2H, **H**-1,2), 4.50 (s, 2H, -C**H**_**2**_-N), 4.22 (dd, 1H, *J* = 9.6 Hz, *J* = 3.8 Hz, **H**-3), 4.01 (m, 1H, -OC**H**_**2**_-CH_2_Cl), 3.88 (d, 2H, *J* = 4.0 Hz, **H**-6), 3.86–3.71 (m, 9H, **H**-4,5, -OC**H**_**2**_-C**H**_**2**_Cl, -C**H**_**2**_-C**H**_**2**_-). ^13^C NMR (400 MHz, CD_3_OD): δ­(ppm) = 161.7 (**C**_**q**_), 144.5 (**C**_**Trq**_), 125.0 (**C**_**TrCH**_), 99.8 (**C**-1), 75.4 (**C**-5), 70.8 (**C**-3), 69.7 (-O**C**H_**2**_-CH_2_Cl), 67.9 (**C**-4), 66.2 (**C**-2), 62.2 (**C**-6), 44.9–43.9 (−OCH_**2**_–**C**H_2_Cl, -**C**H_2_-**C**H_2_-), 39.0 (-**C**H_**2**_-N). HRMS (ESI): *m*/*z* calculated for [C_14_H_24_ClN_6_O_5_]^+^: 391.1497 [M + H]^+^, found: 391.1494; *m*/*z* calculated for [C_14_H_23_ClN_6_NaO_5_]^+^: 413.1311 [M + Na]^+^, found: 413.1313.

#### 2-Chloroethyl 2-Deoxy-2-(4-(((1,4,5,6-tetrahydropyrimidin-2-yl)­amino)­methyl)-1,2,3-triazol-1-yl)-α-D-mannopyranoside **(5)**

The azide **1** (55 mg, 0.14 mmol, 1 equiv) was deacetylated in dry MeOH (2.8 mL) at room temperature under N_2_ atmosphere. A freshly prepared 1 M NaOMe solution in MeOH was added dropwise to 0.015 M final concentration of MeONa. The reaction was stirred at room temperature, under N_2_ atmosphere for 1.5 h to afford **50** (35 mg, 0.13 mmol, 94%). *R*_f_ (**50**): 0.29 in toluene/AcOEt (1:1). ^1^H NMR (400 MHz, CD_3_OD): δ­(ppm) = 4.86 (br s, 1H, **H**-1), 3.99–3.91 (m, 2H, **H**-3, -OC**H**_**2**_-CH_2_Cl), 3.87 (dd, 1H, *J* = 4.05 Hz, *J* = 1.3 Hz, **H**-2), 3.82 (d, 1H, *J* = 11.8 Hz, **H**-6a), 3.78–3.63 (m, 4H, -OC**H**_**2**_-C**H**_**2**_Cl, **H**-6b), 3.62–3.53 (m, 2H, **H**-4,5). ^13^C NMR (400 MHz, CD_3_OD): δ­(ppm) = 99.8 (**C**-1), 75.0 (**C**-5), 72.4 (**C**-3), 69.1 (-O**C**H_**2**_-CH_2_Cl), 68.6 (**C**-4), 65.6 (**C**-2), 62.7 (**C**-6), 43.8 (−OCH_**2**_–**C**H_2_Cl). **50** (65 mg, 0.24 mmol, 1 equiv) and **15**·**HI** (64 mg, 0.24 mmol, 1 equiv) were treated according to the general procedure for CuAAC reaction to afford **5**, which was purified via UPLC (Flow: 10 mL/min; UV channels: 210 nm; 254 nm; A:H_2_O + 0.1% HCOOH, B: CH_3_CN. Gradient: 0–1 min: 0% B, 1–20 min: 0–60% B, 20–23 min: 60–100%; *t*_r_ (**5**) = 10.31 min) (formate salt, yellowish foam, 11 mg, 0.026 mmol, 11%). ^1^H NMR (400 MHz, CD_3_OD): δ­(ppm) = 8.54 (br s, 1H, **H**_**HCOOH**_), 8.20 (s, 1H, **H**_**CHTr**_), 5.11 (m, 2H, **H**-1,2), 4.44 (s, 2H, -C**H**_**2**_-N), 4.21 (dd, 1H, *J* = 9.1 Hz, *J* = 5.0 Hz, **H**-3), 4.04–3.98 (m, 1H, -OC**H**_**2**_-CH_**2**_Cl), 3.88 (d, 2H, *J* = 3.9 Hz, **H**-6), 3.86–3.70 (m, 5H, **H**-4,5, -OC**H**_**2**_-C**H**_**2**_Cl), 3.38 (t, 4H, *J* = 5.7 Hz, N–C**H**_**2**_–CH_2_–C**H**_**2**_–N), 1.97 (qui, 2H, *J* = 5.7 Hz, N–CH_2_–C**H**_**2**_–CH_2_–N). ^13^C NMR (400 MHz, CD_3_OD): δ­(ppm) = 170.3 (H**C**OOH), 154.8 (**C**_**q**_), 144.3 (**C**_**Trq**_), 124.7 (**C**_**TrCH**_), 99.5 (**C-**1), 75.1 (**C-**5), 70.2 (**C-**3), 69.4 (-O**C**H_2_–CH_2_Cl), 67.6 (**C-**4), 65.5 (**C-**2), 61.8 (**C-**6), 43.8 (−OCH_2_–**C**H_2_Cl), 39.8 (N–CH_2_–**C**H_2_–CH_2_–N), 37.2 (-**C**H_**2**_-N), 21.1 (*N*–**C**H_2_–CH_2_–**C**H_2_–N). HRMS (ESI): *m*/*z* calculated for [C_15_H_26_ClN_6_O_5_]^+^: 405.1648 [M + H]^+^, found: 405.1654.

### General Procedure for the Synthesis of **2**, **3**, **8–11** (Zemplén Deacetylation)

The acetylated compound (1 equiv) was dissolved in dry MeOH ([Substrate] = 0.05 M) at room temperature under N_2_ atmosphere. A freshly prepared 1 M NaOMe solution in MeOH was added dropwise to 0.015 M final concentration of MeONa. The reaction was stirred at room temperature, under N_2_ atmosphere. Upon completion (TLC analysis), the reaction was neutralized with Amberlite IR120 ion-exchange resin (hydrogen form), filtered and concentrated under vacuum to give the product.

#### 2-Chloroethyl 2-Deoxy-2-(4-pyrimidin-2-ylamino)­methyl)-1,2,3-triazol-1-yl)-α-D-mannopyranoside **(2)**

**2** was obtained as a white foam from **40** (52 mg, 0.10 mmol) according to general procedure for Zemplén deacetylation (37 mg, 0.09 mmol, 94%). *R*_f_ (**2**): 0.35 in CHCl_3_:MeOH (85:15). ^1^H NMR (400 MHz, CD_3_OD): δ­(ppm) = 8.27 (d, 2H, *J* = 4.7 Hz, **H**_**Ar**_-4,6), 8.14 (s, 1H, **H**_**TrCH**_), 6.62 (t, 1H, *J* = 4.7 Hz, **H**_**Ar**_-5), 5.13–5.06 (m, 2H, **H**-1,2), 4.63 (s, 2H, -C**H**_**2**_-N), 4.19 (dd, 1H, *J* = 9.4 Hz, *J* = 5.2 Hz, **H**-3), 4.02–3.94 (m, 1H, -OC**H**_**2**_-CH_2_Cl), 3.89–3.76 (m, 4H, **H**-5,6,-OC**H**_**2**_-CH_2_Cl), 3.75–3.68 (m, 3H, **H**-4,-OCH_2_–C**H**_**2**_Cl). ^13^C NMR (400 MHz, CD_3_OD): δ­(ppm) = 163.3 (**C**_**Ar**_-4), 159.4 (**C**_**Ar**_-6), 146.9 (**C**_**Trq**_), 124.8 (**C**_**TrCH**_), 111.9 (**C**_**Ar**_-5), 99.6 (**C**-1), 75.1 (**C**-5), 70.3 (**C**-3), 69.4 (-O**C**H_**2**_-CH_2_Cl), 67.8 (**C**-4), 65.4 (**C**-2), 62.0 (**C**-6), 43.8 (−OCH_**2**_–**C**H_2_Cl), 37.6 (-**C**H_**2**_-N). HRMS (ESI): *m*/*z* calculated for [C_15_H_22_ClN_6_O_5_]^+^: 401.1335 [M + H]^+^, found: 401.1339; *m*/*z* calculated for [C_15_H_21_ClN_6_NaO_5_]+: 423.1154 [M + H]^+^, found: 423.1160.

#### 2-Chloroethyl 2-Deoxy-2-(4-(((4,6-dimethoxy-1,3,5-triazin-2-yl)­amino)­methyl)-1,2,3-triazol-1-yl)-α-D-mannopyranoside **(3)**

**3** was obtained as a white foam from **41** (292 mg, 0.50 mmol, 1 equiv) according to general procedure for Zemplén deacetylation and purified via UPLC (Flow: 10 mL/min; UV channels: 210 nm; 254 nm; A:H_2_O + 0.1% HCOOH, B: CH_3_CN. Gradient: 0–1 min: 0% B, 1–20 min: 0–60% B, 20–23 min: 60–100%; t_r_ (**3**) = 15.39 min) (217 mg, 0.47 mmol, 94%). *R*_f_ (**3**): 0.28 in CHCl_3_:MeOH (9:1). ^1^H NMR (400 MHz, CD_3_OD): δ­(ppm) = 8.15 (s, 1H, **H**_**TrCH**_), 5.12 (br s, 1H, **H**-1), 5.09 (d, 1H, *J* = 5.2 Hz, **H**-2) 4.67 (s, 2H, -C**H**_**2**_-N), 4.19 (dd, 1H, *J* = 9.4 Hz, *J* = 5.2 Hz, **H**-3), 4.02–3.92 (m, 4H, -OC**H**_**2**_-CH_2_Cl, O**Me**), 3.91–3.67 (m, 10H, **H**-4,5,6, -OC**H**_**2**_-C**H**_**2**_Cl, O**Me**). ^13^C NMR (400 MHz, CD_3_OD): δ­(ppm) = 173.7 (**C**–OMe), 173.3 (**C**–OMe), 169.6 (**C**_**q**_), 146.2 (**C**_**Trq**_), 124.8 (**C**_**TrCH**_), 99.7 (**C**-1), 75.2 (**C**-5), 70.4 (**C**-3), 69.2 (-O**C**H_**2**_-CH_2_Cl), 67.9 (**C**-4), 65.2 (**C**-2), 62.0 (**C**-6), 55.3 (O**Me**), 55.1 (O**Me**), 43.9 (−OCH_**2**_–**C**H_2_Cl), 37.1 (-**C**H_**2**_-N). HRMS (ESI): *m*/*z* calculated for [C_16_H_24_ClN_7_NaO_7_]^+^: 484.1318 [M + Na]^+^, found: 484.1326.

#### 2-Chloroethyl 2-Deoxy-2-((4-((pyridin-2-ylamino)­methyl))-1,2,3-triazol-1-yl)-α-D-mannopyranoside **(8)**

Compound **46** (109 mg, 0.18 mmol, 1 equiv) was treated according to the general procedure for Zemplén deacetylation, to afford the deacetylated intermediate as a white foam (67 mg, 0.14 mmol, 77%). *R*_f_: 0.51 in CHCl_3_:MeOH (9:1). ^1^H NMR (400 MHz, CD_3_OD): δ­(ppm) = 8.39 (ddd, 1H, *J* = 5.0 Hz, *J* = 2.0 Hz, *J* = 1.2 Hz, **H**_**Ar**_-6), 8.06 (s, 1H, **H**_**TrCH**_), 7.75 (ddd, 1H, *J* = 8.8 Hz, *J* = 7.8 Hz, *J* = 2.0 Hz, **H**_**Ar**_-4), 7.54 (d, *J* = 8.8 Hz, **H**_**Ar**_-3), 7.16 (ddd, 1H, *J* = 7.8 Hz, *J* = 5.0 Hz, *J* = 1.0 Hz, **H**_**Ar**_-5), 5.15 (s, 2H, -C**H**_**2**_-N), 5.08 (d, 1H, *J* = 1.2 Hz, **H**-1), 5.04 (dd, 1H, *J* = 5.1 Hz, *J* = 1.2 Hz, **H**-2), 4.16 (dd, 1H, *J* = 9.3 Hz, *J* = 5.1 Hz, **H**-3), 4.02–3.96 (m, 1H, -OC**H**_**2**_-CH_**2**_Cl), 3.87 (dd, 1H, *J* = 13.8 Hz, *J* = 3.7 Hz, **H**-6a), 3.83–3.76 (m, 3H, **H**-5.6b, -OC**H**_**2**_-CH_**2**_Cl), 3.73 (t, 2H, *J* = 5.1 Hz, –OCH_2_–C**H**_**2**_Cl), 3.65 (t, 1H, *J* = 9.3 Hz, **H**-4), 1.47 (s, 9H, *t* **Bu**). ^13^C NMR (400 MHz, CD_3_OD): δ­(ppm) extrapolated from HSQC = 148.1 (**C**_**Ar**_-6), 138.6 (**C**_**Ar**_-4), 124.3 (**C**_**TrCH**_), 121.9 (**C**_**Ar**_-3), 121.6 (**C**_**Ar**_-5), 99.5 (**C**-1), 74.9 (**C**-5), 70.1 (**C**-3), 69.3 (-O**C**H_2_–CH_**2**_Cl), 67.9 (**C**-4), 65.4 (**C**-2), 62.1 (**C**-6), 43.8 (−OCH_2_–**C**H_**2**_Cl), 43.3 (-**C**H_**2**_-N), 28.4 (*t* **Bu**). MS (ESI): *m*/*z* calculated for [C_21_H_21_ClN_5_O_7_]^+^: 501.20 [M + H]^+^, found: 501.04. The crude (67 mg, 0.13 mmol, 1 equiv) was not purified but directly dissolved in a 4:1 mixture of dry CH_2_Cl_2_ and trifluoroacetic acid (2.6 mL) and the reaction was stirred at room temperature, under N_2_ atmosphere. After 4 h, the mixture was concentrated under vacuum and coevaporated with toluene three times. The resulting crude was purified via UPLC (flow: 10 mL/min; UV channels: 210 nm; 254 nm; A:H_2_O + 0.1% HCOOH, B: CH_3_CN. Gradient: 0–5 min: 0% B, 5–20 min: 0–50% B, 20–20.5 min: 50–100%; *t*_r_ (**8**) = 13.99 min) leading to **8** as the trifluoroacetate salt (white foam, 66 mg, 0.12 mmol, 99%). *R*_f_ (**8**): 0.74 in CH_2_Cl_2_:MeOH (8:2). ^1^H NMR (400 MHz, CD_3_OD): δ­(ppm) = 8.28 (s, 1H, **H**_**TrCH**_), 7.96 (t, 1H, *J* = 9.0 Hz, **H**_**Ar**_-4), 7.90 (d, 1H, *J* = 6.2 Hz, **H**_**Ar**_-6), 7.15 (d, 1H, *J* = 9.0 Hz, **H**_**Ar**_-3), 6.95 (t, 1H, *J* = 6.2 Hz, **H**_**Ar**_-5), 5.15–5.09 (m, 2H, **H**-1,2), 4.71 (s, 2H, -C**H**_**2**_-N), 4.16 (dd, 1H, *J* = 9.5 Hz, *J* = 4.8 Hz, **H**-3), 4.04–3.97 (m, 1H, -OC**H**_**2**_-CH_**2**_Cl), 3.87 (d, 2H, *J* = 3.0 Hz, **H**-6), 3.83–3.78 (m, 2H, H-5, -OC**H**_**2**_-CH_**2**_Cl), 3.77–3.69 (m, 3H, H-4, –OCH_2_–C**H**_**2**_Cl). ^13^C NMR (400 MHz, CD_3_OD): δ (ppm) = 154.4 (**C**_**q**_), 145.0 (**C**_**Ar**_-4), 143.4 (**C**_**Trq**_), 136.7 (**C**_**Ar**_-6), 125.1 (**C**_**TrCH**_), 114.5 (**C**_**Ar**_-3), 114.1 (**C**_**Ar**_-5), 99.5 (**C**-1), 75.1 (**C**-5), 70.1 (**C**-3), 69.4 (-O**C**H_2_–CH_**2**_Cl), 67.6 (**C**-4), 65.5 (**C**-2), 61.8 (**C**-6), 43.8 (−OCH_2_–**C**H_**2**_Cl), 38.2 (-**C**H_**2**_-N). HRMS (ESI): *m*/*z* calculated for [C_16_H_23_ClN_5_O_5_]^+^: 400.1382 [M + H]^+^, found: 389.1394; *m*/*z* calculated for [C_16_H_22_ClN_5_NaO_5_]^+^: 422.1202 [M + Na]^+^, found: 422.1213.

#### 2-Chloroethyl 2-Deoxy-2-(4-(((1H-imidazol-2-yl)­amino)­methyl)-1,2,3-triazol-1-yl)-α-D-mannopyranoside **(9)**

Compound **47** (146 mg, 0.20 mmol, 1 equiv) was treated according to the general procedure for Zemplén deacetylation, to afford the deacetylated intermediate as a white foam (102 mg, 0.19 mmol, 99%). *R*_f_: 0.49 in CHCl_3_:MeOH (9:1). ^1^H NMR (400 MHz, CDCl_3_): δ­(ppm) = 8.05 (s, 1H, **H**_**TrCH**_), 6.88 (s, 2H, **H**_**Ar**_-5,6), 5.20–4.98 (m, 4H, **H**-1,2, -C**H**_**2**_-N), 4.18 (dd, 1H, *J* = 10.0 Hz, *J* = 4.0 Hz, **H**-3), 4.02 (m, 1H, -OC**H**_**2**_-CH_**2**_Cl), 3.94–3.72 (m, 6H, **H**-5,6, -OC**H**_**2**_-C**H**_**2**_Cl), 3.67 (t, 1H, **H**-4), 1.50 (s, 9H, *t* **Bu**). ^13^C NMR (400 MHz, CD_3_OD): δ­(ppm) extrapolated from HSQC = 123.3 (**C**_**TrCH**_), 100.4 (**C**_**Ar**_-4,5), 98.4 (**C**-1), 74.0 (**C**-5), 69.2 (**C**-3), 68.0 (-O**C**H_2_–CH_**2**_Cl), 66.3 (**C**-4), 63.5 (**C**-2), 60.7 (**C**-6), 42.2 (−OCH_2_–**C**H_**2**_Cl), 43.3 (-**C**H_**2**_-N), 26.1 (*t* **Bu**). MS (ESI): *m*/*z* calculated for [C_19_H_30_ClN_6_O_7_]^+^: 489.19 [M + H]^+^, found: 489.10. The crude (102 mg, 0.19 mmol, 1 equiv) was not purified but directly dissolved in a 4:1 mixture of dry CH_2_Cl_2_ and trifluoroacetic acid (3.8 mL). The reaction was stirred at room temperature, under N_2_ atmosphere. After 4 h, the mixture was concentrated under vacuum and coevaporated with toluene three times. The resulting crude was purified via UPLC (flow: 10 mL/min; UV channels: 210 nm; 254 nm; A:H_2_O + 0.1% HCOOH, B: CH_3_CN. Gradient: 0–5 min: 0% B, 5–20 min: 0–50% B, 20–20.5 min: 50–100%; *t*_r_ (**9**) = 13.69 min) leading to **9** as trifluoroacetate salt (white foam, 105 mg, 0.18 mmol, 99%). *R*_f_ (**9**): 0.39 in CH_2_Cl_2_:MeOH (8:2). ^1^H NMR (400 MHz, CD_3_OD): δ­(ppm) = 8.22 (s, 1H, **H**_**CHTr**_), 6.88 (s, 2H, **H**_**Ar**_-5,6), 5.13–5.10 (m, 2H, **H**-1,2), 4.58 (s, 2H, -C**H**_**2**_-N), 4.21 (dd, *J* = 9.6 Hz, *J* = 4.6 Hz, 1H, **H**-3), 4.01–3.97 (m, 1H, -OC**H**_**2**_-CH_**2**_Cl), 3.90 (d, 2H, *J* = 3.2 Hz, **H**-6), 3.82–3.76 (m, 2H, **H**-5, -OC**H**_**2**_-CH_**2**_Cl), 3.77–3.69 (m, 2H, **H**-4, –OCH_2_–C**H**_**2**_Cl). ^13^C NMR (400 MHz, CD_3_OD): δ­(ppm) = 144.5 (**C**_**q**_), 135.8 (**C**_**Trq**_), 124.7 (**C**_**TrCH**_), 115 (**C**_**Ar**_-4,5) 99.5 (**C**-1), 74.9 (**C**-5), 70.2 (**C**-3), 69.4 (-O**C**H_2_–CH_**2**_Cl), 67.7 (**C**-4), 65.5 (**C**-2), 61.9 (**C**-6), 43.7 (−OCH_2_–**C**H_**2**_Cl), 39.1 (-**C**H_**2**_-N). HRMS (ESI): *m*/*z* calculated for [C_14_H_22_ClN_6_O_5_]^+^: 389.1335 [M + H]^+^, found: 389.1341; *m*/*z* calculated for [C_14_H_21_ClN_6_NaO_5_]^+^: 411.1154 [M + Na]^+^, found: 411.1160.

#### 2-Chloroethyl 2-Deoxy-2-(4-(((1H-benzo­[*d*]­imidazol-2-yl)­amino)­methyl)-1,2,3-triazol-1-yl)-α-D-mannopyranoside **(10)**

Compound **48** (100 mg, 0.13 mmol, 1 equiv) was treated according to the general procedure for Zemplén deacetylation, to afford the deacetylated intermediate as a white foam (66 mg, 0.12 mmol, 94%) that was directly submitted to Boc removal. *R*_f_: 0.61 in CH_2_Cl_2_: MeOH (9:1). ^1^H NMR (400 MHz, CD_3_OD): δ­(ppm) = 8.12 (s, 1H, **H**_**TrCH**_), 7.47 (m, 2H, **H**_**Ar**_-4,7), 7.15 (dd, 1H, *J* = 6.1 Hz, *J* = 2.8 Hz, **H**_**Ar**_-5,6), 5.34 (s, 2H, -C**H**_**2**_-N), 5.08 (br s, 1H, **H**-1), 5.05 (d, *J* = 5.2 Hz, 1H, **H**-2), 4.15 (dd, 1H, *J* = 9.7 Hz, *J* = 4.0 Hz, **H**-3), 4.03–3.95 (m, 1H, -OC**H**_**2**_-CH_**2**_Cl), 3.85–3.70 (m, 6H, **H**-5,6, -OC**H**_**2**_-C**H**_**2**_Cl), 3.64 (t, 1H, **H**-4), 1.54 (s, 9H, *t* **Bu**). ^13^C NMR (400 MHz, CD_3_OD): δ­(ppm) extrapolated from HSQC = 126.5 (**C**_**Ar**_-5,6), 123.6 (**C**_**TrCH**_), 121.1 (**C**_**Ar**_-4,7), 98.1 (**C**-1), 74.0 (**C**-5), 68.9 (**C**-3), 67.8 (-O**C**H_2_–CH_**2**_Cl), 66.6 (**C**-4), 63.8 (**C**-2), 60.7 (**C**-6), 42.5 (−OCH_2_–**C**H_**2**_Cl), 41.1 (-**C**H_**2**_-N), 26.9 (*t* **Bu**). MS (ESI): *m*/*z* calculated for [C_23_H_32_ClN_6_O_7_]^+^: 539.20 [M + H]^+^, found: 539.26. The crude (66 mg, 0.12 mmol, 1 equiv) was not purified but directly dissolved in a 4:1 mixture of dry CH_2_Cl_2_ and trifluoroacetic acid (2.4 mL) and the reaction was stirred at room temperature, under N_2_ atmosphere. After 4 h, the mixture was concentrated under vacuum and coevaporated with toluene three times. The resulting crude was purified via UPLC (flow: 10 mL/min; UV channels: 210 nm; 254 nm; A:H_2_O + 0.1% HCOOH, B: CH_3_CN. Gradient: 0–1 min: 0% B, 1–20 min: 0–60% B, 20–23 min: 60–100%; *t*_r_ (**10**) = 15.12 min) leading to **10** as trifluoroacetate salt (white foam, 66 mg, 0.11 mmol, 99%). *R*_f_ (**10**): 0.49 in CH_2_Cl_2_:MeOH (8:2). ^1^H NMR (400 MHz, CD_3_OD): δ­(ppm) = 8.29 (s, 1H, **H**_**CHTr**_), 7.43–7.36 (m, 2H, **H**_**Ar**_-4,7), 7.34–7.27 (m, 2H, **H**_**Ar**_-5,6), 5.13 (m, 2H, **H**-1,2), 4.74 (s, 2H, -C**H**_**2**_-N), 4.22 (dd, 1H, *J* = 9.3 Hz, *J* = 4.1 Hz, **H**-3), 4.05–3.97 (m, 1H, -OC**H**_**2**_-CH_**2**_Cl), 3.87 (d, 2H, *J* = 2.9 Hz, **H**-6), 3.82–3.78 (m, 2H, **H**-5, -OC**H**_**2**_-CH_**2**_Cl), 3.77–3.70 (m, 3H, **H**-4, –OCH_2_–C**H**_**2**_Cl). ^13^C NMR (400 MHz, CD_3_OD): δ­(ppm) = 151.8 (**C**_**q**_), 144.3 (**C**_**q**_ + **C**_**q**_), 131.2 (**C**_**Trq**_), 125.3 (**C**_**TrCH**_), 125.2 (**C**_**Ar**_-5,6), 112.6 (**C**_**Ar**_-4,7), 99.8 (**C**-1), 75.2 (**C**-5), 70.3 (**C**-3), 69.5 (-O**C**H_2_–CH_**2**_Cl), 67.7 (**C**-4), 65.7 (**C**-2), 61.9 (**C**-6), 44.1 (−OCH_2_–**C**H_**2**_Cl), 39.4 (-**C**H_**2**_-N). HRMS (ESI): *m*/*z* calculated for [C_18_H_24_ClN_6_O_5_]^+^: 439.1491 [M + H]^+^, found: 439.1498; *m*/*z* calculated for [C_18_H_23_ClN_6_NaO_5_]^+^: 461.1311 [M + Na]^+^, found: 461.1314.

#### 2-Chloroethyl 2-Deoxy-2-(4-(((5-methoxy-1H-benzo­[d]­imidazol-2-yl)­amino)­methyl)-1,2,3-triazol-1-yl)-α-D-mannopyranoside (**11**)

Compound **49** (25 mg, 0.03 mmol, 1 equiv) was treated according to the general procedure for Zemplén deacetylation, to afford the deacetylated intermediate as a white foam (14 mg, 0.02 mmol, 82%) that was directly submitted to Boc removal. *R*_f_: 0.48 in CHCl_3_:MeOH (9:1). ^1^H NMR (400 MHz, CD_3_OD): δ­(ppm) = 8.10 (s, 1H, **H**_**TrCH**_), 7.34 (d, 1H, *J* = 8.7 Hz, **H**_**Ar**_-7), 7.02 (d, 1H, *J* = 2.4 Hz, **H**_**Ar**_-4), 6.79 (dd, 1H, *J* = 8.7 Hz, *J* = 2.4 Hz, **H**_**Ar**_-6), 5.29 (s, 2H, -C**H**_**2**_-N), 5.08 (d, 1H, *J* = 1.1 Hz, **H**-1), 5.05 (dd, 1H, *J* = 5.2 Hz, *J* = 1.1 Hz, **H**-2), 4.15 (dd, 1H, *J* = 9.7 Hz, *J* = 5.2 Hz, **H**-3), 4.01–3.94 (m, 1H, -OC**H**_**2**_-CH_**2**_Cl), 3.85–3.70 (m, 9H, **H**-5,6, -OC**H**_**2**_-C**H**_**2**_Cl, O**Me**), 3.64 (t, 1H, *J* = 9.7 Hz, **H**-4), 1.53 (s, 9H, *t* **Bu**). ^13^C NMR (400 MHz, CD_3_OD): δ­(ppm) extrapolated from HSQC = 123.6 (**C**_**TrCH**_), 110.0 (**C**_**Ar**_-6), 102.1 (**C**_**Ar**_-7), 98.1 (**C**-1), 97.0 (**C**_**Ar**_-4), 74.0 (**C**-5), 69.5 (**C**-3), 68.0 (-O**C**H_2_–CH_**2**_Cl), 66.9 (**C**-4), 63.8 (**C**-2), 61.2 (**C**-6), 54.7 (O**Me**), 42.2 (−OCH_2_–**C**H_**2**_Cl), 41.7 (-**C**H_**2**_-N), 26.4 (*t* **Bu**). MS (ESI): *m*/*z* calculated for [C_24_H_34_ClN_6_O_8_]^+^: 570.02 [M + H]^+^, found: 570.09. The crude (14 mg, 0.02 mmol, 1 equiv) was not purified but directly dissolved in a 4:1 mixture of dry CH_2_Cl_2_ and trifluoroacetic acid (0.4 mL) and the reaction was stirred at room temperature, under N_2_ atmosphere. After 4 h, the mixture was concentrated under vacuum and coevaporated with toluene three times. The resulting crude was purified via UPLC (flow: 10 mL/min; UV channels: 210 nm; 254 nm; A:H_2_O + 0.1% HCOOH, B: CH_3_CN. Gradient: 0–5 min: 0% B, 5–20 min: 0–50% B, 20–20.5 min: 50–100%; t_r_ (**11**) = 16.65 min) leading to **11** as trifluoroacetate salt (white foam, 11 mg, 0.02 mmol, 99%). *R*_f_ (**11**): 0.43 in CH_2_Cl_2_:MeOH (8:2). ^1^H NMR (400 MHz, CD_3_OD): δ­(ppm) = 8.28 (s, 1H, **H**_**CHTr**_), 7.27 (d, 1H, *J* = 8.8 Hz, **H**_**Ar**_-7), 6.94 (d, 1H, *J* = 2.2 Hz, **H**_**Ar**_-4), 6.88 (dd, 1H, *J* = 8.8 Hz, *J* = 2.2 Hz, **H**_**Ar**_-6), 5.13–5.11 (m, 2H, **H**-1,2), 4.72 (s, 2H, -C**H**_**2**_-N), 4.21 (dd, 1H, *J* = 9.3 Hz, *J* = 4.8 Hz, **H**-3), 4.03–3.98 (m, 1H, -OC**H**_**2**_-CH_**2**_Cl), 3.87 (d, 2H, *J* = 3.2 Hz, **H**-6), 3.85–3.77 (m, 5H, **H**-5, -OC**H**_**2**_-CH_**2**_Cl, O**Me**), 3.76–3.70 (m, 3H, **H**-4, –OCH_2_–C**H**_**2**_Cl). ^13^C NMR (400 MHz, CD_3_OD): δ­(ppm) = 158.7 (**C**_**q**_), 151.8 (**C**_**q**_), 143.8 (**C**_**q**_ + **C**_**q**_), 132.0 (**C**_**Trq**_), 124.9 (**C**_**TrCH**_), 113.0 (**C**_**Ar**_-7), 112.1 (**C**_**Ar**_-6), 99.5 (**C**-1), 97.8 (**C**_**Ar**_-4), 75.1 (**C**-5), 70.2 (**C**-3), 69.4 (-O**C**H_2_–CH_**2**_Cl), 67.7 (**C**-4), 65.5 (**C**-2), 61.9 (**C**-6), 56.4 (O**Me**), 43.8 (−OCH_2_–**C**H_**2**_Cl), 39.2 (-**C**H_**2**_-N). HRMS (ESI): *m*/*z* calculated for [C_19_H_26_ClN_6_O_6_]^+^: 469.1597 [M + H]^+^, found: 469.1608; *m*/*z* calculated for [C_19_H_25_ClN_6_NaO_6_]^+^: 491.1416 [M + Na]^+^, found: 491.1424.

### General Procedure for the Synthesis of 6, 7 (Transesterification Conditions).^40^

The acetylated substrate (1 equiv) was dissolved in a mixture of EtOH and CHCl_3_ (*V*_EtOH_/*V*_CHCl3_ = 3:1, [Substrate] = 0.075 M) and HCl (37% *aq*, 0.2 equiv) was added to the solution. The reaction mixture was stirred and kept at 40 °C for 23 h. Upon total consumption of the starting material (^1^H NMR monitoring), the solvents were removed in vacuo. The obtained crude was purified by UPLC according to the conditions reported below.

#### 2-Chloroethyl 2-Deoxy-2-(4-(((pyrimidin-4­(3H)-onyl)­amino)­methyl)-1,2,3-triazol-1-yl)-α-D-manno Pyranoside **(6)**

**6** was obtained as a white foam from **44** (40 mg, 0.07 mmol, 1 equiv) according to general procedure for transesterification and purified via UPLC (Flow: 10 mL/min; UV channels: 210 nm; 254 nm; A:H_2_O + 0.1% HCOOH, B: CH_3_CN. Gradient: 0–1 min: 0% B, 1–20 min: 0–60% B, 20–23 min: 60–100%; *t*_r_ (**6**) = 10.35 min) (25 mg, 0.06 mmol, 95%). ^1^H NMR (400 MHz, CD_3_OD): δ­(ppm) = 8.24 (s, 1H, **H**_**TrCH**_), 7.64 (d, 2H, *J* = 6.7 Hz, **H**_**Ar**_-6), 5.75 (d, 1H, *J* = 6.7 Hz, **H**_**Ar**_-5), 5.11 (br s, 1H, **H**-1), 5.08 (dd, 1H, *J* = 5.1 Hz, *J* = 0.9 Hz, **H**-2), 4.61 (s, 2H, -C**H**_**2**_-N), 4.19 (dd, 1H, *J* = 9.2 Hz, *J* = 5.1 Hz, **H**-3), 4.00–3.97 (m, 1H, -OC**H**_**2**_-CH_**2**_Cl), 3.87 (d, 2H, *J* = 3.5 Hz, **H**-6), 3.83–3.71 (m, 5H, **H**-4,5, -OC**H**_**2**_-C**H**_**2**_Cl). ^13^C NMR (400 MHz, CD_3_OD): δ (ppm) = 165.0 (**C**_**Ar**_-6), 125.6 (**C**_**TrCH**_), 104.6 (**C**_**Ar**_-5), 99.2 (**C**-1), 74.9 (**C**-5), 70.3 (**C**-3), 69.5 (-O**C**H_2_–CH_2_Cl), 67.8 (**C**-4), 65.5 (**C**-2), 62.0 (**C**-6), 43.9 (−OCH_2_–**C**H_2_Cl), 37.1 (-**C**H_**2**_-N). HRMS (ESI): *m*/*z* calculated for [C_15_H_21_ClN_6_NaO_6_]^+^: 439.1103 [M + Na]^+^, found: 439.1111.

#### 2-Chloroethyl 2-Deoxy-2-(4-(((pyrimidin-2­(1H)-onyl)­amino)­methyl)-1,2,3-triazol-1-yl)-α-D-manno Pyranoside **(7)**

**7** was obtained as a white foam from **45** (150 mg, 0.27 mmol, 1 equiv) according to general procedure for transesterification and purified via UPLC (Flow: 10 mL/min; UV channels: 210 nm; 254 nm; A:H_2_O + 0.1% HCOOH, B: CH_3_CN. Gradient: 0–1 min: 0% B, 1–20 min: 0–60% B, 20–23 min: 60–100%; *t*_r_ (**7**) = 8.73 min) (104 mg, 0.25 mmol, 93%). ^1^H NMR (400 MHz, CD_3_OD): δ­(ppm) = 8.34 (s, 1H, **H**_**TrCH**_), 7.33 (d, 2H, *J* = 7.3 Hz, **H**_**Ar**_-6), 5.79 (d, 1H, *J* = 7.3 Hz, **H**_**Ar**_-5), 5.10 (br s, 1H, **H**-1), 5.08 (d, 1H, *J* = 5.3 Hz, **H**-2), 4.62 (dd, 2H, *J* = 15.3 Hz, *J* = 6.5 Hz, -C**H**_**2**_-N), 4.19 (dd, 1H, *J* = 8.1 Hz, *J* = 5.6 Hz, **H**-3), 4.02–3.97 (m, 1H, -OC**H**_**2**_-CH_**2**_Cl), 3.87 (d, 2H, *J* = 2.2 Hz, **H**-6), 3.83–3.73 (m, 5H, **H**-4,5, -OC**H**_**2**_-C**H**_**2**_Cl). ^13^C NMR (400 MHz, CD_3_OD): δ (ppm) = 166.5 (**C**_**q**_), 160.5 (**C**_**q**_), 145.3 (**C**_**Trq**_), 142.5 (**C**_**Ar**_-6), 125.6 (**C**_**TrCH**_), 99.5 (**C**-1), 96.3 (**C**_**Ar**_-5), 75.3 (**C**-5), 70.2 (**C**-3), 69.3 (-O**C**H_2_–CH_**2**_Cl), 67.7 (**C**-4), 65.4 (**C**-2), 61.9 (**C**-6), 43.8 (−OCH_2_–**C**H_**2**_Cl), 36.6 (-**C**H_**2**_-N). HRMS (ESI): *m*/*z* calculated for [C_15_H_21_ClN_6_NaO_6_]^+^: 439.1103 [M + Na]^+^, found: 439.1113.

### Sars-Cov-2 Spike Protein, L-Sign and DC-SIGN Constructs for SPR and X-ray Crystallographic Studies

L-SIGN 3G-ECD (amino acids 78 to 399), L-SIGN CRD (amino acids 266 to 399) and DC-SIGN 3G-ECD (amino acids 66 to 404) were produced as previously described.^26,41,42^ Constructs used were supplemented with 3 glycines “3G” residues on the *N*-terminus side. The name “lectin ECD” has been used in the main text rather than “lectin 3G-ECD” to improve clarity and readability. Glycosylated Spike hexaPro, stabilized version of the spike protein thanks to 6 Proline inserted mutations, has been produced and purified as described.^23,32^

### Competition Experiments by Surface Plasmon Resonance

Surface plasmon resonance (SPR) experiments were performed on a Biacore T200 instrument at 25 °C. Two types of competition experiments were conducted using Spike functionalized surfaces prepared using a CM4 sensorchip. Flow cell 1 (Fc1) was used as a reference surface. Fc1 to Fc3 were activated with 60 μL of a mixture of EDC-NHS (according to manufacturer). Fc1 was functionalized with 16.7 μL of BSA at 20 μg/mL in 10 mM NaOAc (pH = 4). Remaining activated groups were neutralized with 60 μL of 1 M ethanolamine (pH = 8.5). After functionalization, all surfaces were washed with 100 μL of 10 mM HCl followed by 100 μL of 50 mM NaOH/1 M NaCl at 100 μL/min. Finally, Fc2 and 3 were functionalized with Spike HexaPro at 20 μg/mL in 100 mM NaOAc (pH = 5), reaching 1700 and 2000 RU respectively. Competition experiments were performed with decreasing concentrations of compounds from 5 mM to 10 μM, using a 1:2 dilution. Compounds were coinjected with 20 μM of DC-SIGN or 20 μM of L-SIGN over Spike surface. Flow was set at 5 μL/min in 25 mM HEPES pH 8, 150 mM NaCl, 4 mM CaCl_2_, supplemented with 0.05% Tween, with an association and dissociation time set to 100 s each. Surface regeneration was done for 10 s at 100 μL/min with a 150 s stabilization period, using 50 mM EDTA for DC-SIGN tests and 50 mM glycine NaOH (pH = 12) 0.15% Triton 25 mM EDTA for L-SIGN.

DC/L-SIGN ECD equilibrium binding responses (Req) for each sample were obtained from the reference surface corrected sensorgrams 95 s after the start of the injection. The obtained Req values were converted to DC-SIGN residual activity values (*y*, %) with respect to Req of DC/L-SIGN alone, which was assigned a 100% activity value. After plotting residual activity against corresponding compound concentration, the 4-parameter logistic model (eq, 1) was fitted to the plots, and finally the IC_50_ values were calculated using eq 2.

1 $${y=R}_{hi}-\frac{R_{hi}-R_{lo}}{{1+\left(\frac{Conc}{A_{1}}\right)}^{A_{2}}}$$

2 $${{IC}_{50}=A_{1}\cdot \left(\frac{R_{hi}-R_{lo}}{R_{hi}-50}\right)}^{1/A_{2}}$$

where *R*_hi_ and *R*_lo_ are maximum and minimum asymptotes, A1 is the inflection point and A2 is a slope of the curve. All the graphs were modeled using Kaleidagraph software and 4 parameter equation. Sensorgrams and inhibition curves are reported in the Supporting Information

### Structure Resolution of L-Sign CRD/4 Complex by X-ray Crystallography

Crystallization experiments were performed thanks to HTX Lab platform at EMBL Grenoble^43^ with a sample of L-SIGN 3G-CRD concentrated at 20.1 mg/mL containing 3.75 mM of **4**. The crystallization experiments were carried out using the sitting-drop vapor-diffusion method with a Mosquito robot (SPTLabtech) with a mix of 0.1 μL of protein solution and 0.05 μL of reservoir equilibrated against 45 μL reservoir solution at 20 °C in 96-well CrystalDirect plates (MiTeGen) and automatically imaged in RockImager robot (Formulatrix). Best crystals grew in condition 1–13 of JCSG-plus screen (molecular dimensions; 0.8 M ammonium sulfate, 0.1 M citrate pH 4). Automated crystal cryo-cooling and harvesting were performed with CrystalDirect Technology.^44–46^

X-ray diffraction data were collected at ID30A-1/MASSIF-1 beamline at ESRF Grenoble.^47^ A data set of 1040 images with 0.05° oscillation, was collected. Data were reduced, integrated, scaled and converted to HKL file using XDS suite.^48^ The structure of L-SIGN 3G-CRD/**4** complex was solved at a 2 Å resolution by molecular replacement using MOLREP and the coordinates of the chain A of 1sl6 structure of L-SIGN CRD^49^ as model. The initial solution was evaluated using COOT^50^ and the final model was obtained by successive rounds of refinement using the programs REFMAC^51^ and manual construction using COOT.^50^ Crystallographic data statistics are presented in Table SI2. The PDB deposition code for the L-SIGN 3G-CRD/**4** complex structure is 9G6W.

### Computational Studies

Docking calculations were performed using the Schrödinger Suite through Maestro graphical interface (Schrödinger Release 2018–1). Docking protocols based on the complex between L-SIGN CRD and **Man84** (PDB code: 8RCY) and on a cocrystal of DC-SIGN CRD (PDB code: 6GHV) were developed using the software Glide and the OPLS3 force field. Ligands were prepared for docking using the LigPrep tool (version 45011) to create energy minimized 3D structures for the most likely charge and tautomeric states. The protonation states were generated at pH 7 ± 2 and then employed in computational studies. Ligand charge and tautomeric states were further controlled by calculating the p*K*_a_ values for the conjugated acid of the heterocyclic fragments by Epik (v. 4.3011). Additional details are reported as Supporting Information

## Supplementary Material







## Notes

Primary data can be found in Supporting Information or can be requested to the authors The Supporting Information is available free of charge at https://pubs.acs.org/doi/10.1021/acs.jmedchem.5c01448.

Includes: sensorgrams and inhibition curves of SPR binding competition assays of DC-SIGN and L-SIGN; aggregation studies; correlation graphs for L-SIGN affinity and selectivity; crystallographic data and statistics of L-SIGN CRD/**4** complex; details of the computational (docking) studies; NMR spectral data of compounds **2–21**; HPLC traces of **2–11** primary data can be found in Supporting Information or can be requested to the authors (PDF)
Coordinates (PDB format) of the docked structures shown in Figures SI-5 and SI-6 (ZIP)
Molecular Formula Strings (CSV)

## Abbreviationused

DIPEA: *N,N*-diisopropylethylamine
HRMS: high resolution mass spectrometry
MS: mass spectrometry
SPR: surface plasmon resonance.
