# Supplementary material for: Increasing the Chemical Space of L-SIGN Specific Glycomimetics
Source: J Med Chem. 2025 Oct 18;68(21):22530–46. doi: 10.1021/acs.jmedchem.5c01448 (PMC12621191; doi:10.1021/acs.jmedchem.5c01448)
Supplement: Supplementary file 1 [file jm5c01448_si_001.pdf]

Supporting Information

Increasing the chemical space of L-SIGN specific glycomimetics

Gianluca Cavazzoli,[a] Clara Delaunay,[b] Sara Pollastri,[a] Andrea Panzeri,[a] Sara Sattin,[a] Michel Thépaut,[b] Laura Belvisi,[a] Franck Fieschi, [b,c]* Anna Bernardi [a]*

a. *Università degli Studi di Milano, Dipartimento di Chimica, via Golgi 19, 20133 Milano*
b. *Univ. Grenoble Alpes, CNRS, CEA, Institut de Biologie Structurale, 38000, Grenoble France.*
c. *Institut Universitaire de France (IUF), 75231, Paris, France*

franck.fieschi@ibs.fr
anna.bernardi@unimi.it

Table of Contents

Table SI-1. Tested compounds. **3**

Sensorgrams and inhibition curves of binding inhibition to immobilized Spike **5**

Binding competition assays with DC-SIGN **5**

DC-SIGN/Man84 5

DC-SIGN/ 2 (Man 89) 6

DC-SIGN/ 3 (Man98) 6

DC-SIGN/ 4 (Man96) 7

DC-SIGN/ 5 (Man102) 8

DC-SIGN/ 6 (Man100) 8

DC-SIGN/ 7 (Man101) 9

DC-SIGN/ 8 (Man99) 10

DC-SIGN/ 9 (Man97) 10

DC-SIGN/ 10 (Man94) 11

DC-SIGN/ 11 (Man103) 12

Binding competition assays with L-SIGN **13**

L-SIGN/Man84 13

L-SIGN/ 2 (Man89) 14

L-SIGN/ 3 (Man98) 15

L-SIGN/ 4 (Man96) 15

L-SIGN/ 5 (Man102) 16

L-SIGN/ 6 (Man100) 17

L-SIGN/ 7 (Man101) 17

L-SIGN/ 8 (Man99) 18

L-SIGN/ 9 (Man97) 19

L-SIGN/ 10 (Man94) 19
L-SIGN/ 11 (Man103) 20
Aggregation studies **21**
Affinity and selectivity correlation graphs **23**
Figure SI-1. 23
Figure SI-2. 23
Figure SI-3. 24
Table SI-2. Crystallographic data and statistics of L-SIGN CRD/4 complex **25**
Computational studies **26**
Ligand preparation **26**
Figure SI- 4. 26
Protein preparation **26**
Docking calculations **26**
Results **27**
Figure SI-5. 28
Figure SI-6. 29
Spectral data for 2-21 .... **30**
HPLC chromatograms of 2-11 .... **51**
References **55**

Table SI-1. Tested compounds.

**Compound**	**Lab name**	**Ligand Structure**<br>SMILE notation	**Tested in SPR as**
**2**	**Man89**	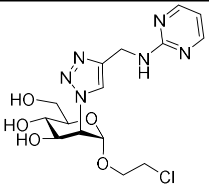<br>OC[C@H]1O[C@H](OCCCl)[C@@H](N2C=C(CNC3=NC=CC=N3)N=N2)[C@@H](O)[C@@H]1O	Neutral
**3**	**Man98**	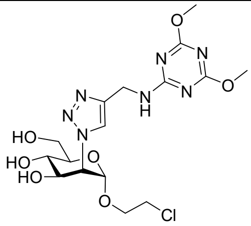<br>OC[C@H]1O[C@H](OCCCl)[C@@H](N2C=C(CNC3=NC(OC)=NC(OC)=N3)N=N2)[C@@H](O)[C@@H]1O	Neutral
**4**	**Man96**	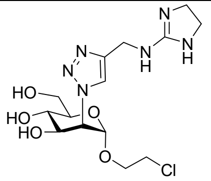<br>OC[C@H]1O[C@H](OCCCl)[C@@H](N2C=C(CNC3=NCCN3)N=N2)[C@@H](O)[C@@H]1O	Formate salt
**5**	**Man102**	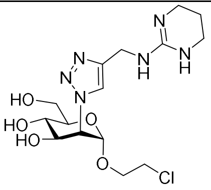<br>OC[C@H]1O[C@H](OCCCl)[C@@H](N2C=C(CNC3=NCCCN3)N=N2)[C@@H](O)[C@@H]1O	Formate salt
**6**	**Man100**	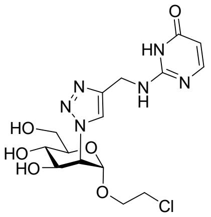<br>OC[C@H]1O[C@H](OCCCl)[C@@H](N2C=C(CNC3=NC=CC(N3)=O)N=N2)[C@@H](O)[C@@H]1O	Neutral
**7**	**Man101**	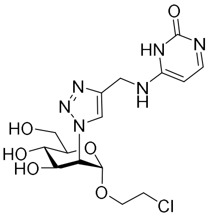<br>OC[C@H]1O[C@H](OCCCl)[C@@H](N2C=C(CNC3=CC=NC(N3)=O)N=N2)[C@@H](O)[C@@H]1O	Neutral

**8**	**Man99**	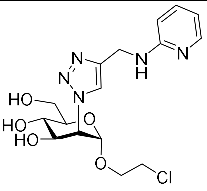<br>OC[C@H]1O[C@H](OCCCl)[C@@H](N2C=C(CNC3=NC=CC=C3)N=N2)[C@@H](O)[C@@H]1O	Trifluoroacetate salt
**9**	**Man97**	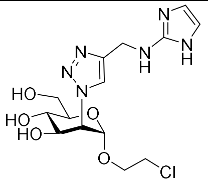<br>OC[C@H]1O[C@H](OCCCl)[C@@H](N2C=C(CNC3=NC=CN3)N=N2)[C@@H](O)[C@@H]1O	Trifluoroacetate salt
**10**	**Man94**	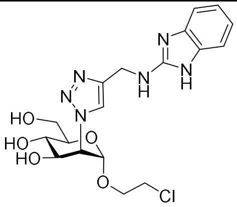<br>OC[C@H]1O[C@H](OCCCl)[C@@H](N2C=C(CNC3=NC4=C(C=CC=C4)N3)N=N2)[C@@H](O)[C@@H]1O	Trifluoroacetate salt
**11**	**Man103**	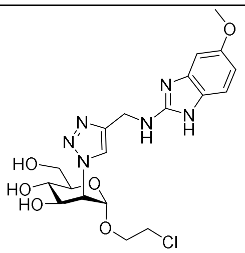<br>OC[C@H]1O[C@H](OCCCl)[C@@H](N2C=C(CNC3=NC4=C(C=CC(OC)=C4)N3)N=N2)[C@@H](O)[C@@H]1O	Trifluoroacetate salt
**51**	**Man79**	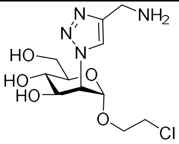<br>OC[C@H]1O[C@H](OCCCl)[C@@H](N2C=C(CN)N=N2)[C@@H](O)[C@@H]1O	Trifluoroacetate salt
**Man84**	**Man84**	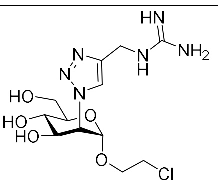<br>OC[C@H]1O[C@H](OCCCl)[C@@H](N2C=C(CNC(N)=N)N=N2)[C@@H](O)[C@@H]1O	Trifluoroacetate salt

Sensorgrams and inhibition curves of binding inhibition to immobilized Spike

The compounds are indicated here by both the **number** used in the main text and (in brackets) by the lab name (assigned during the synthetic work). Numbers and lab names are collected in **Table SI-1**. All experiments were performed in duplicate (on FC2 and FC3, respectively) as described in the main text

Binding competition assays with DC-SIGN

DC-SIGN/Man84

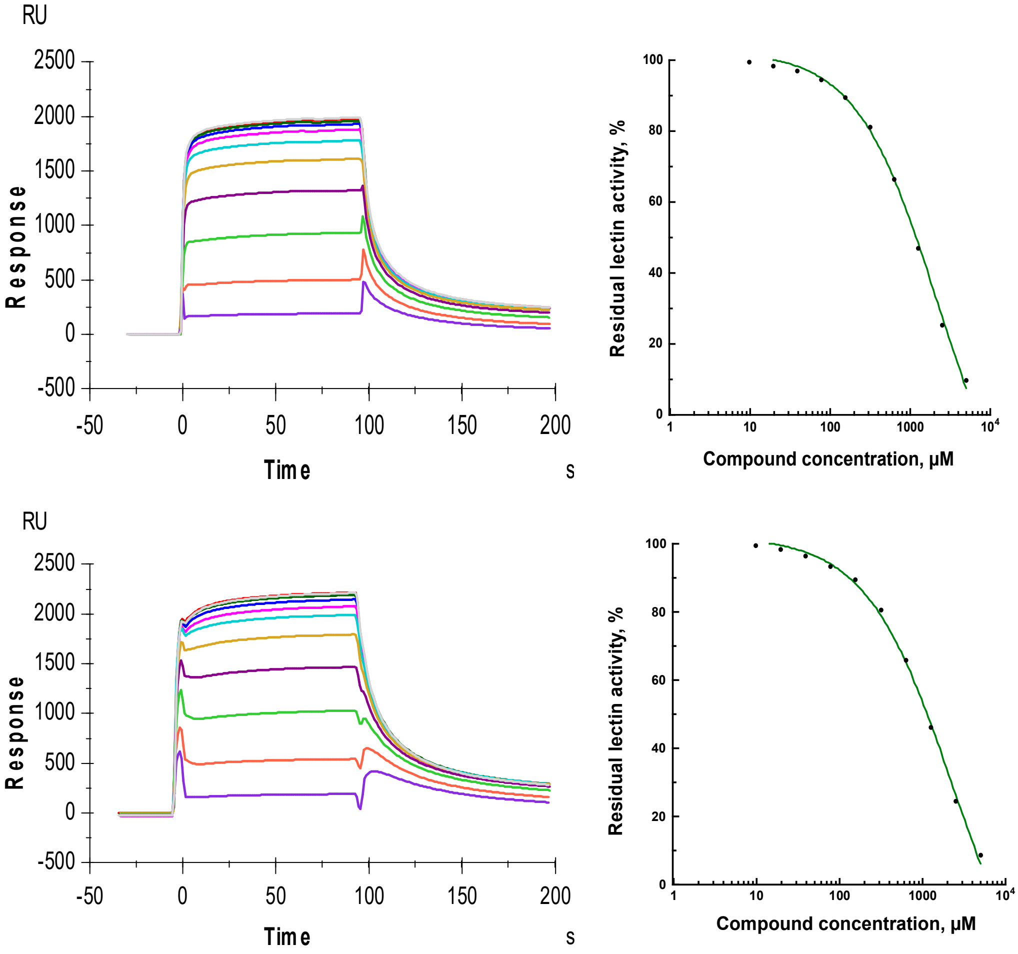

DC-SIGN/ 2 (Man 89)

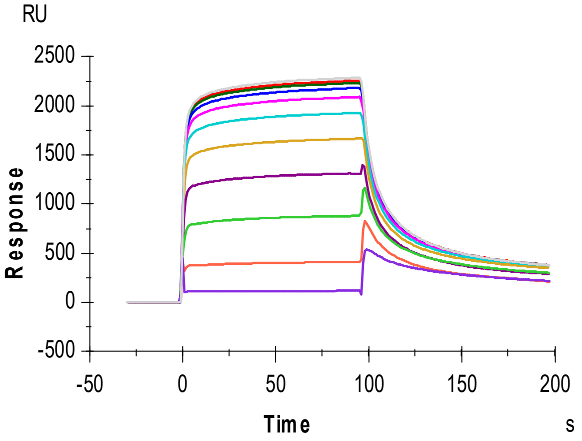

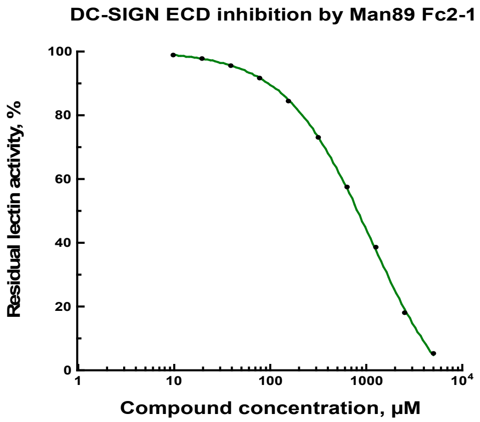

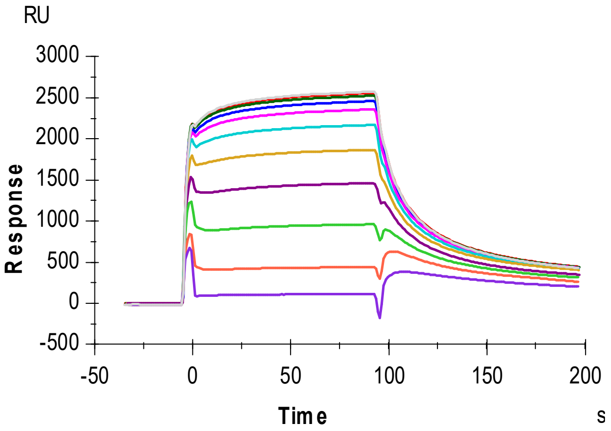

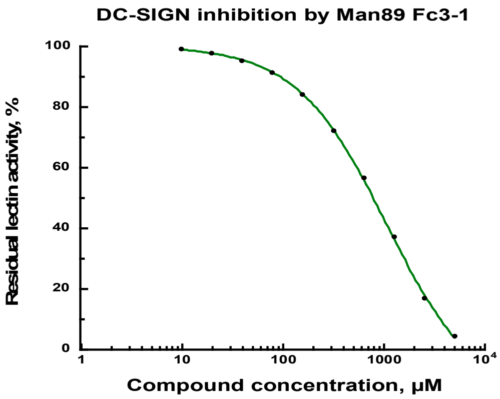

DC-SIGN/ 3 (Man98)

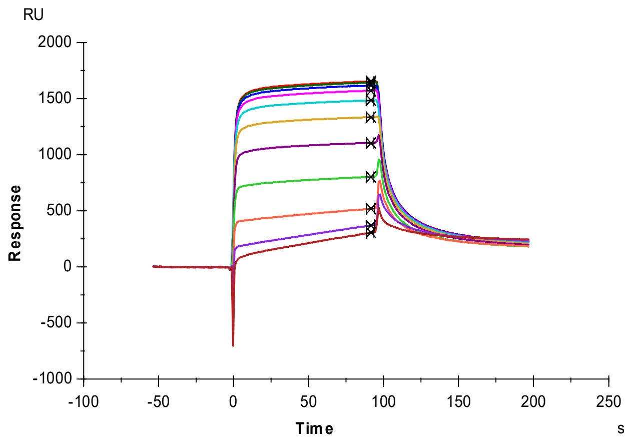

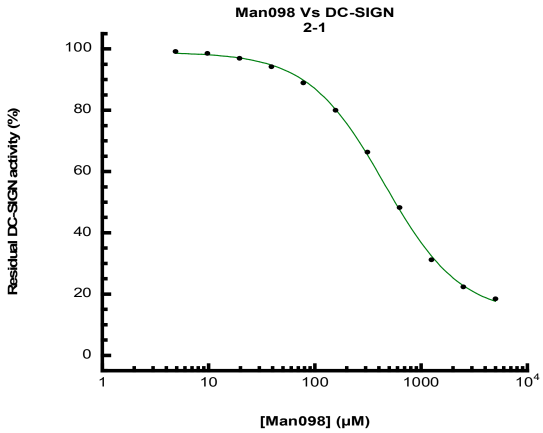

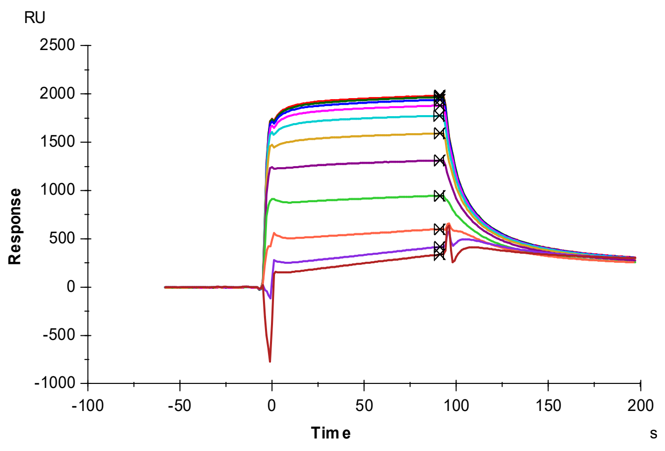

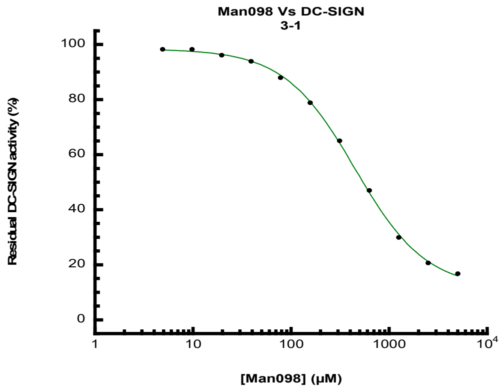

DC-SIGN/ 4 (Man96)

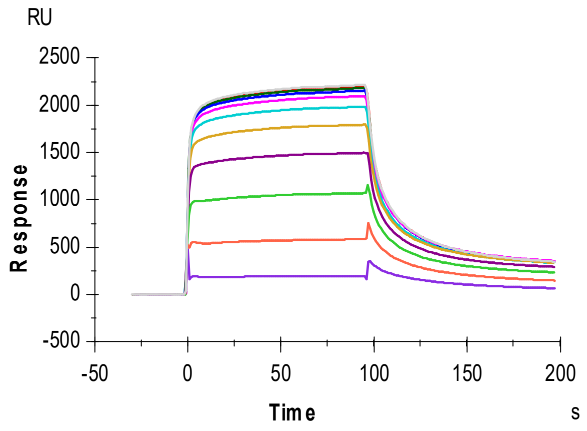

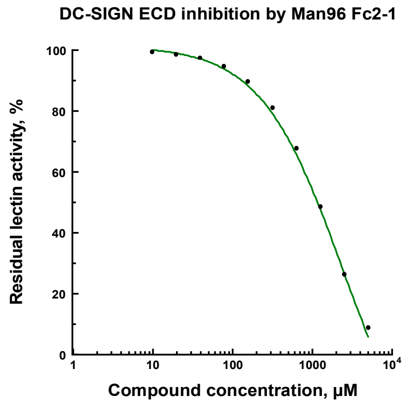

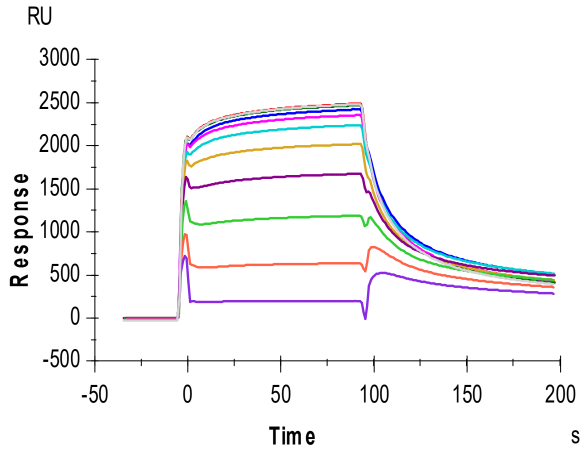

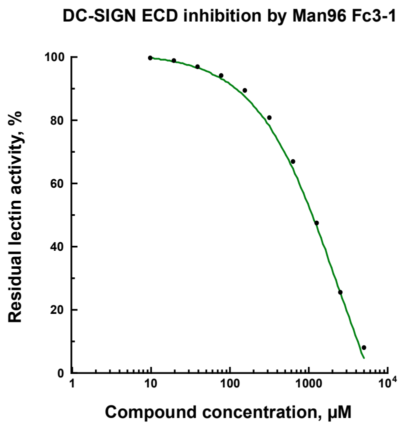

DC-SIGN/ 5 (Man102)

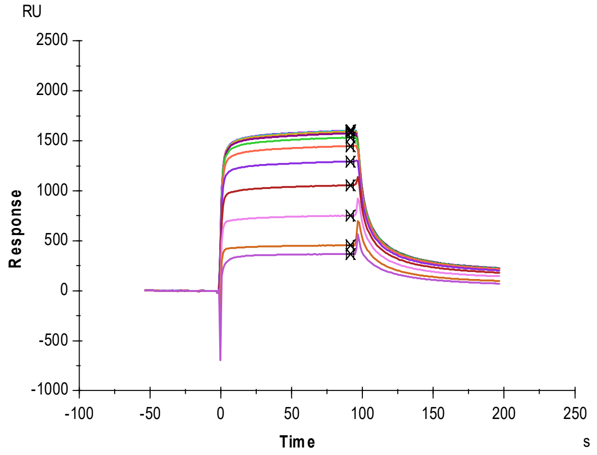

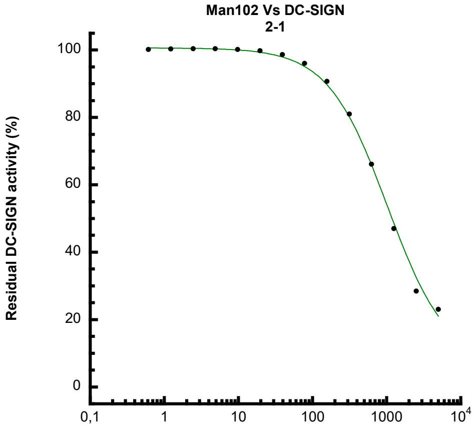

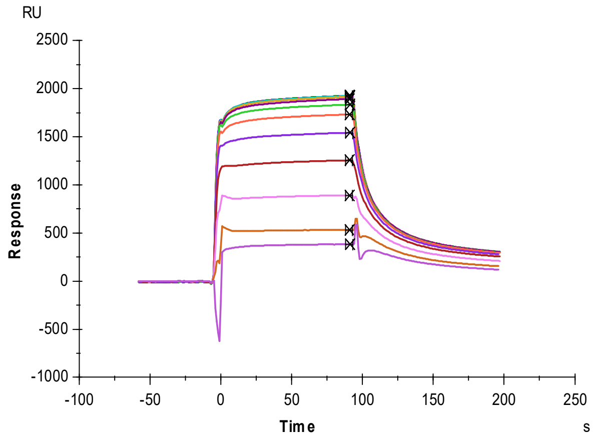

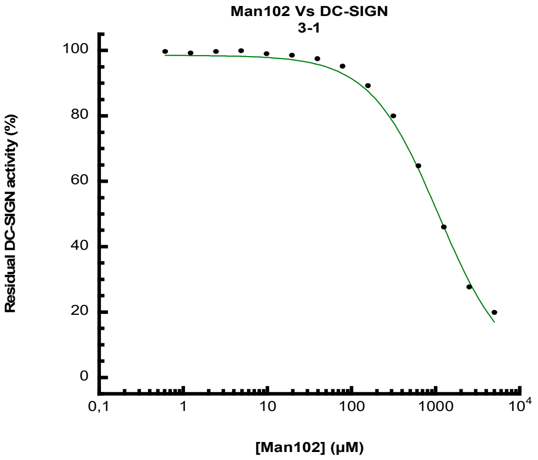

DC-SIGN/ 6 (Man100)

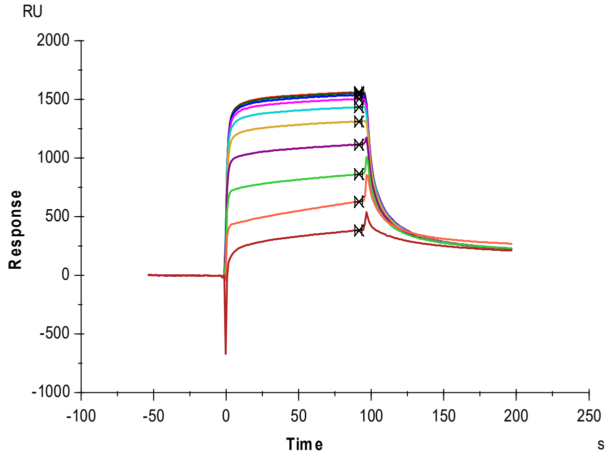

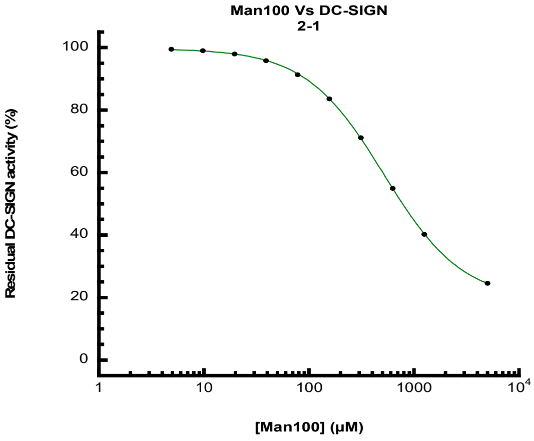

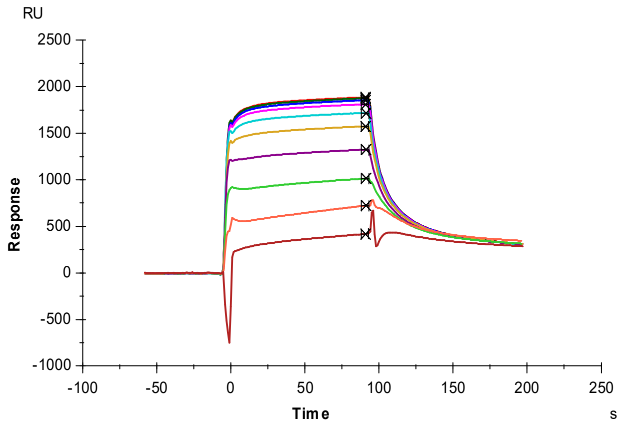

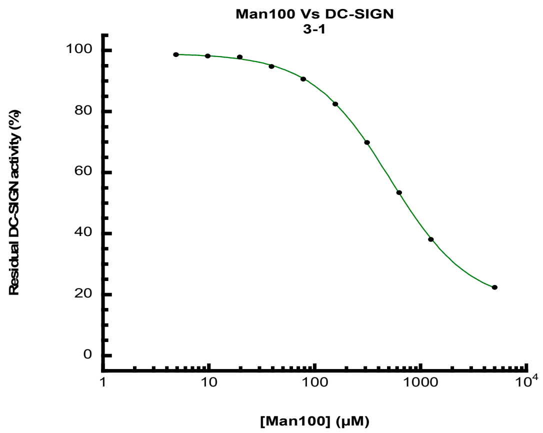

DC-SIGN/ 7 (Man101)

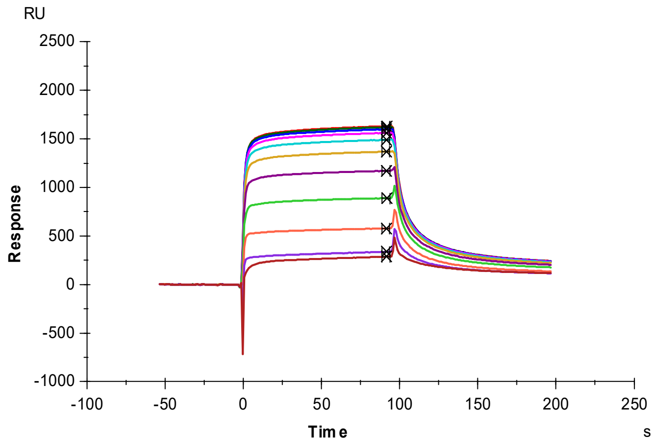

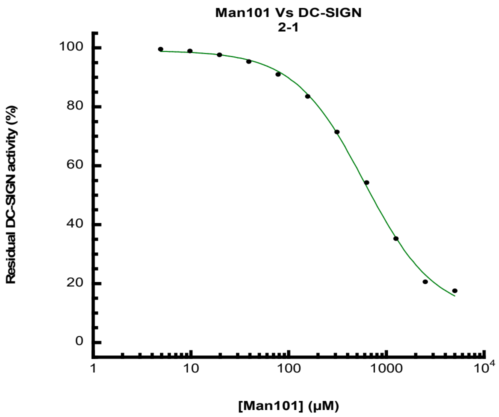

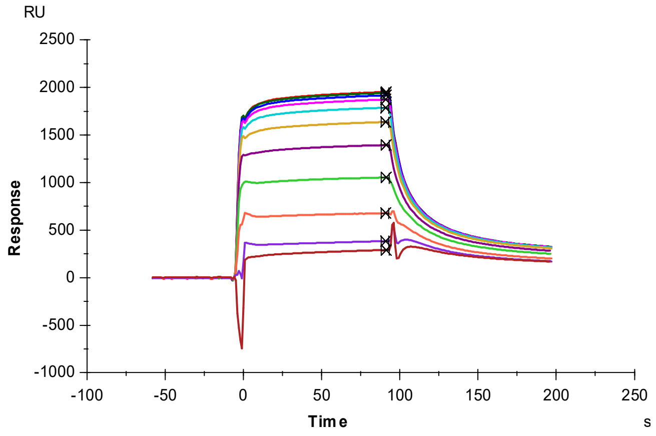

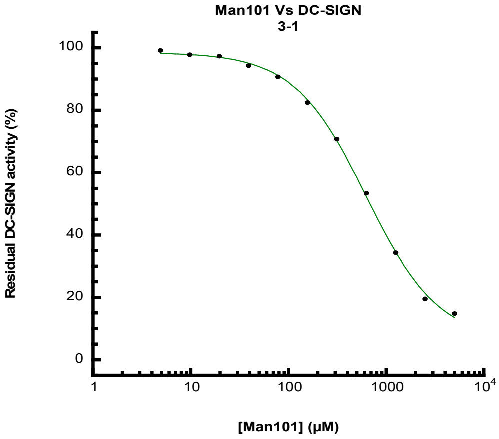

DC-SIGN/ 8 (Man99)

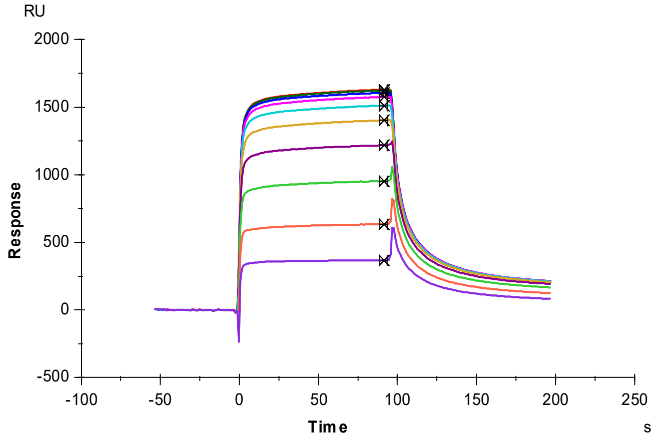

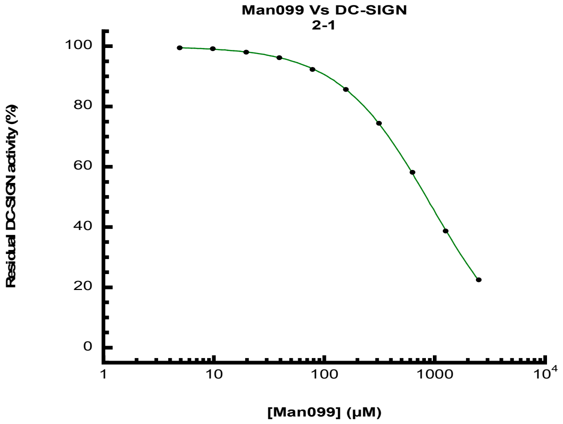

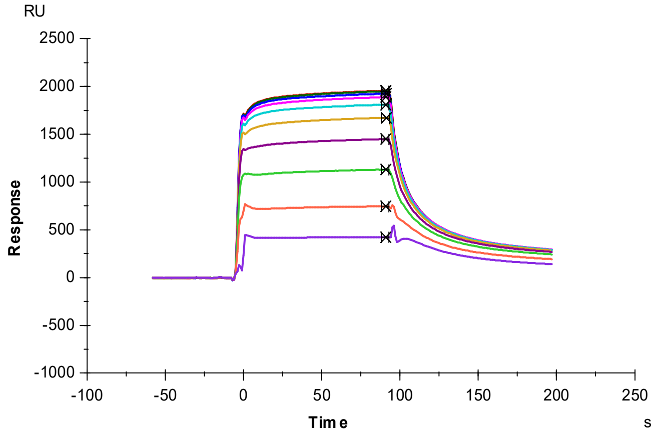

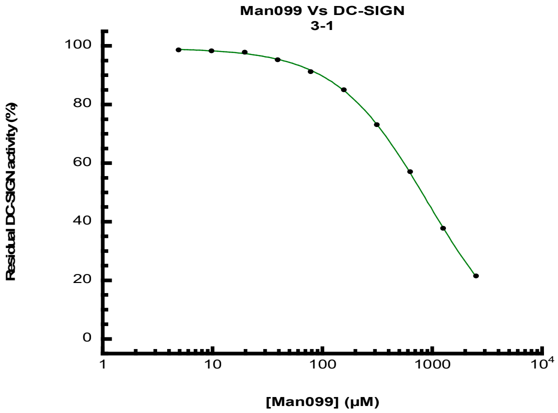

DC-SIGN/ 9 (Man97)

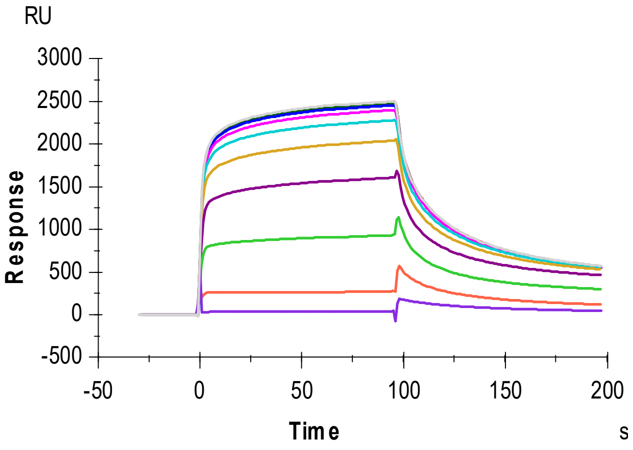

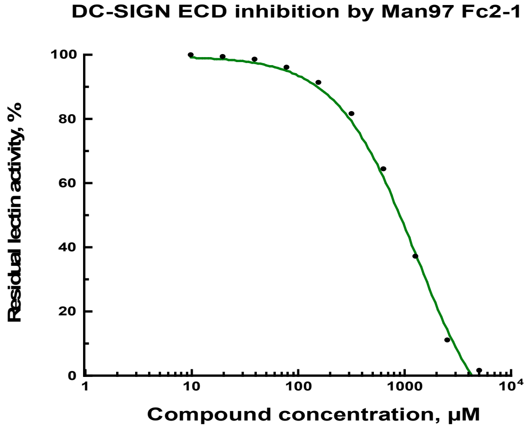

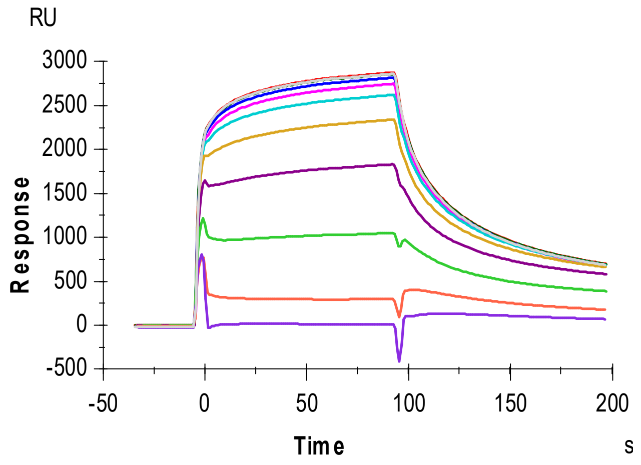

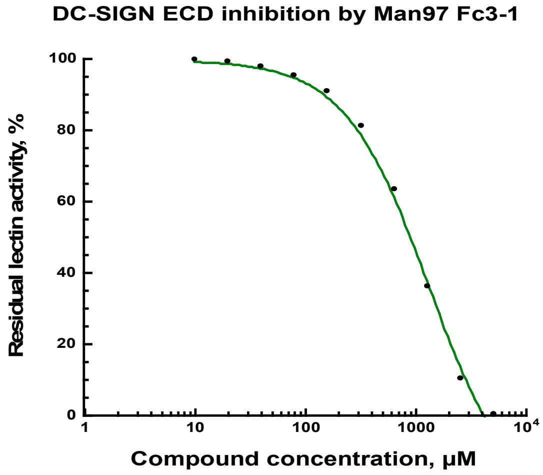

DC-SIGN/ 10 (Man94)

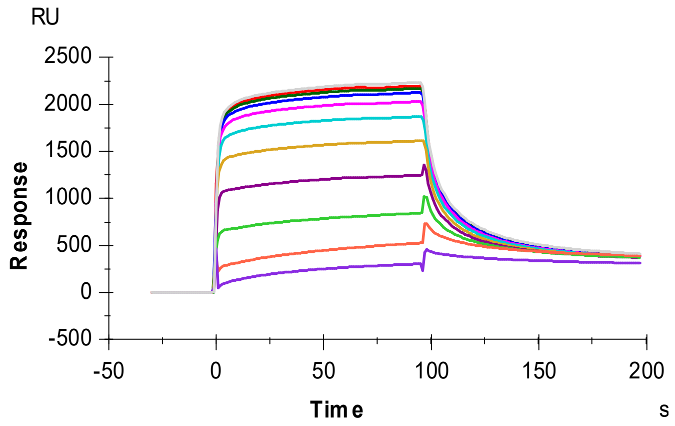

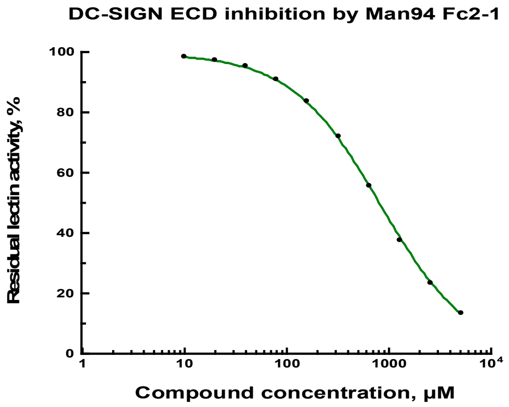

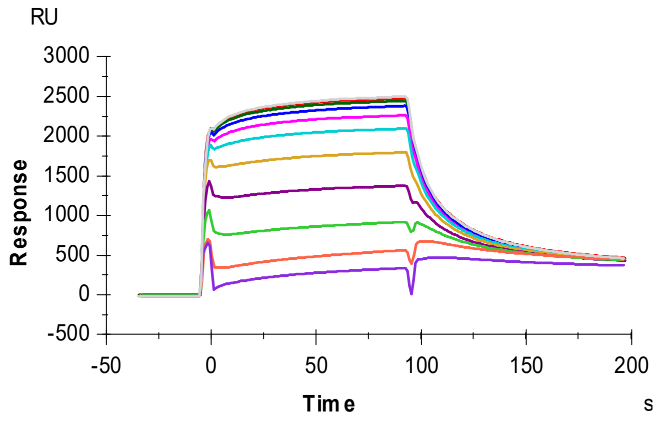

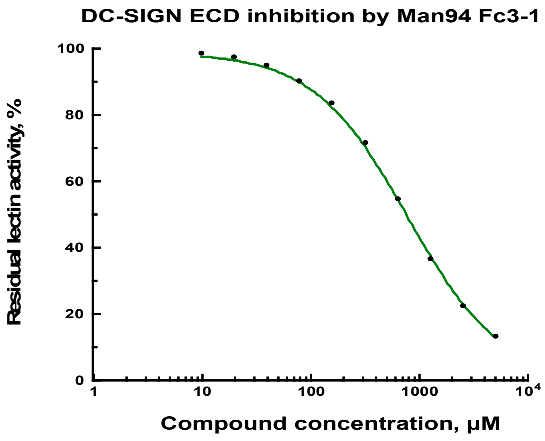

DC-SIGN/ 11 (Man103)

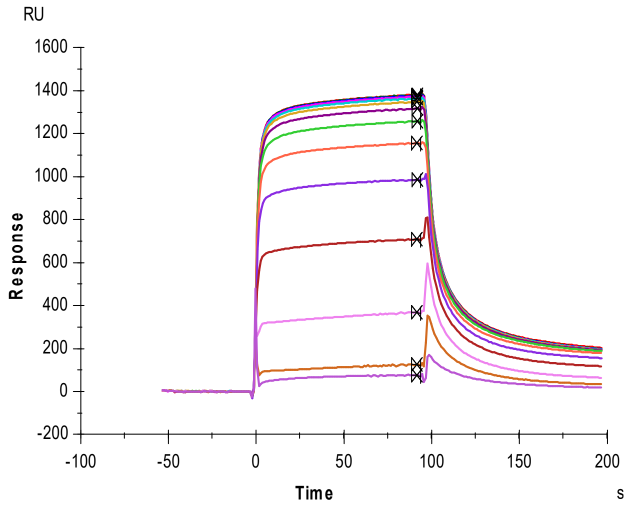

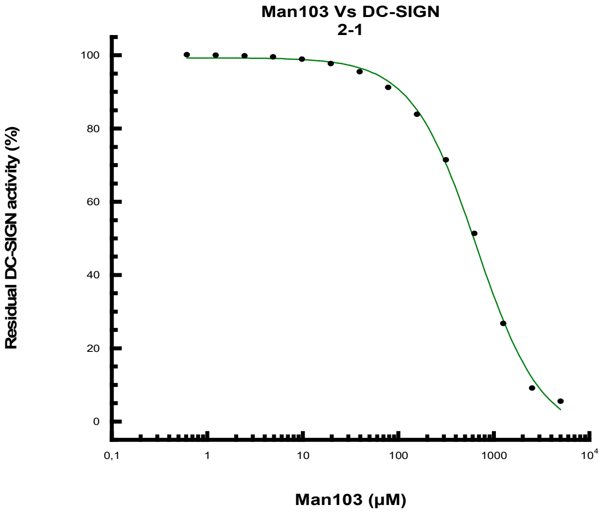

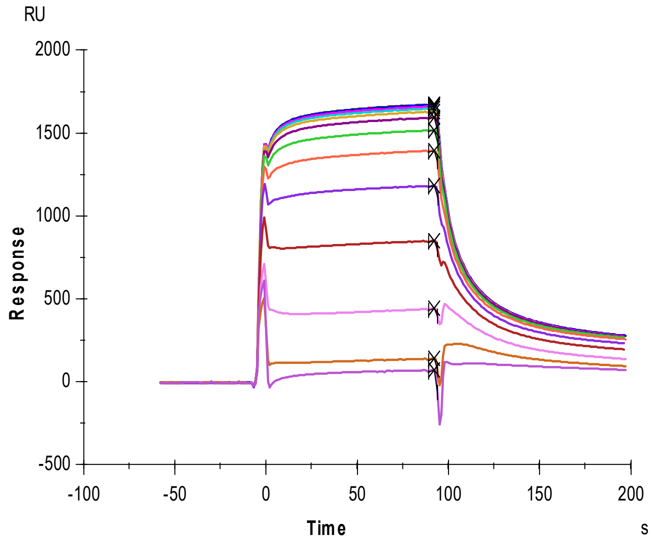

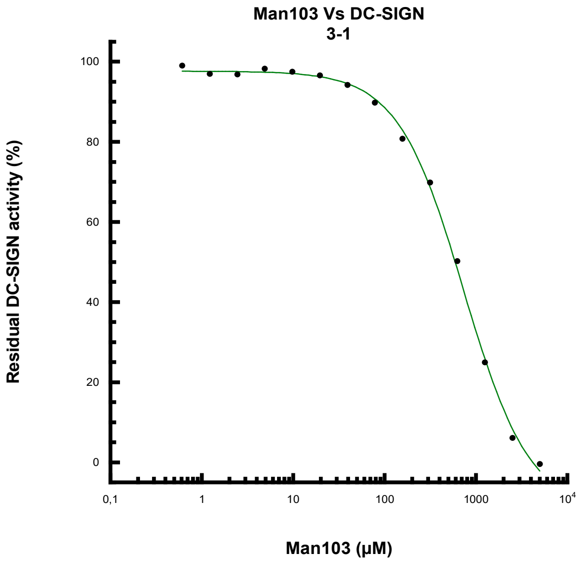

Binding competition assays with L-SIGN

L-SIGN/Man84

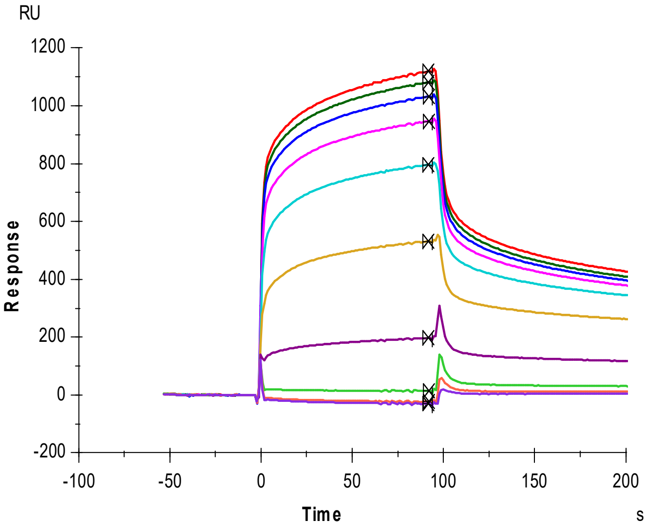

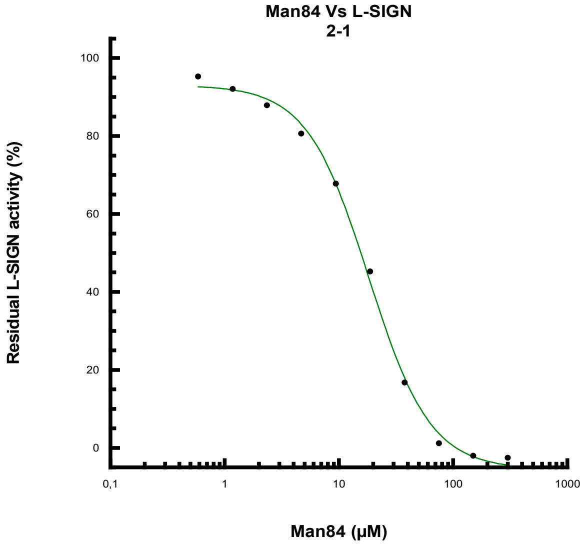

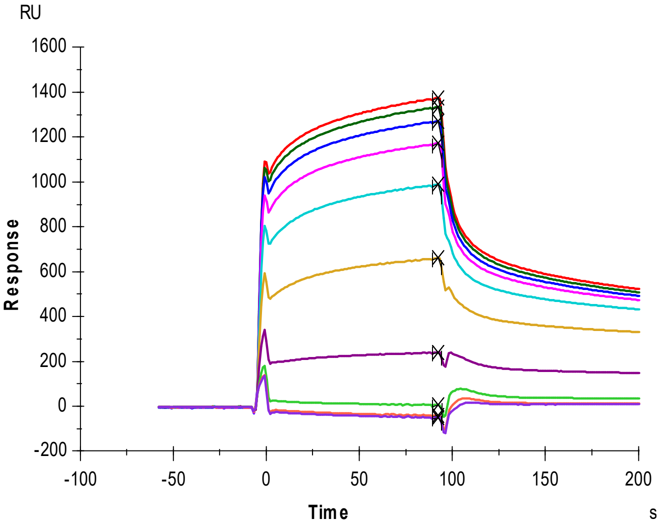

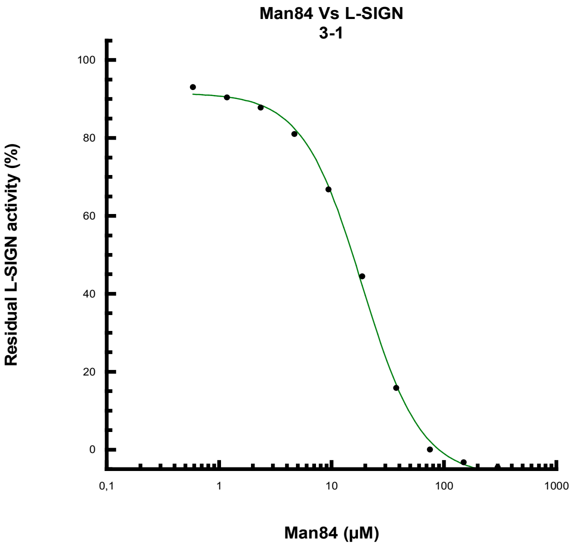

L-SIGN/ 2 (Man89)

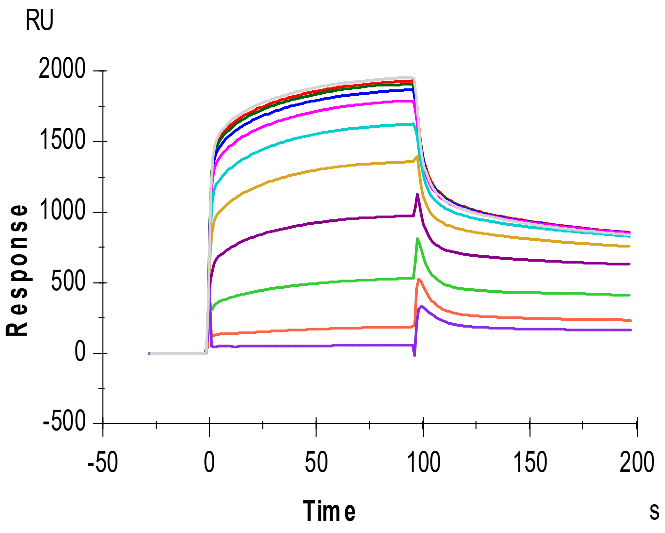

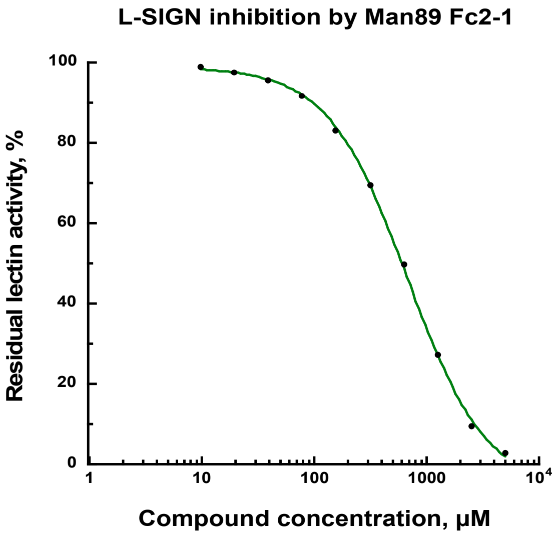

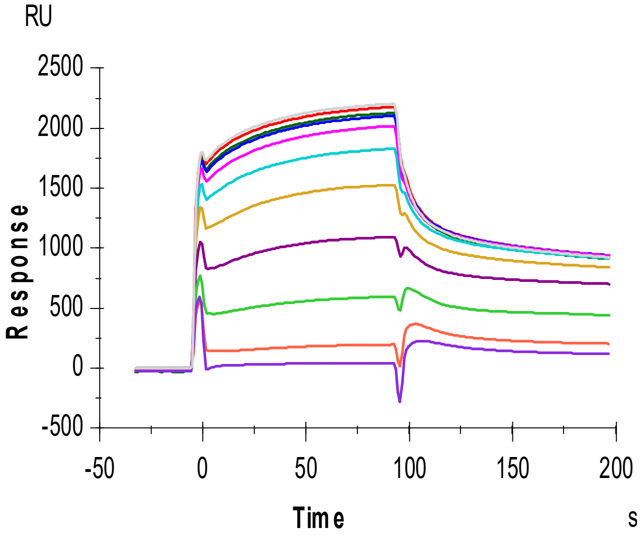

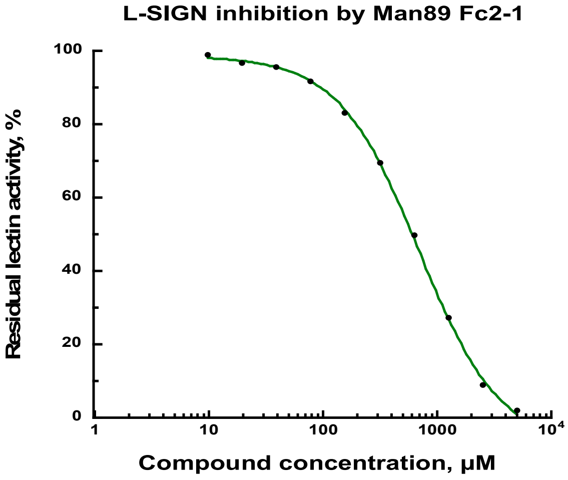

L-SIGN/ 3 (Man98)

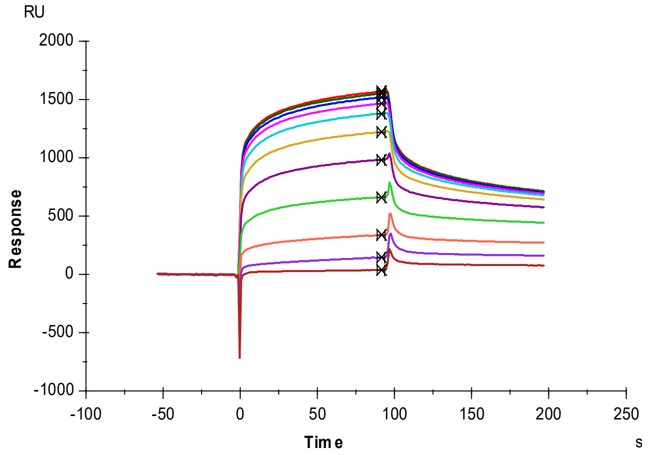

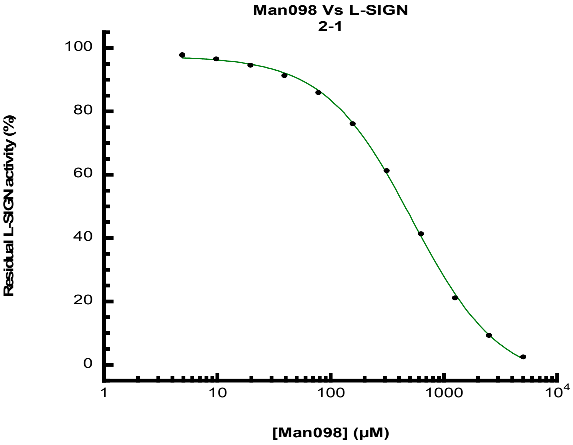

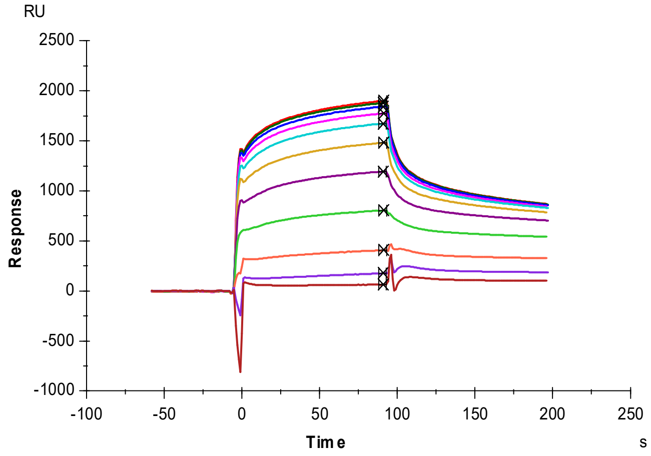

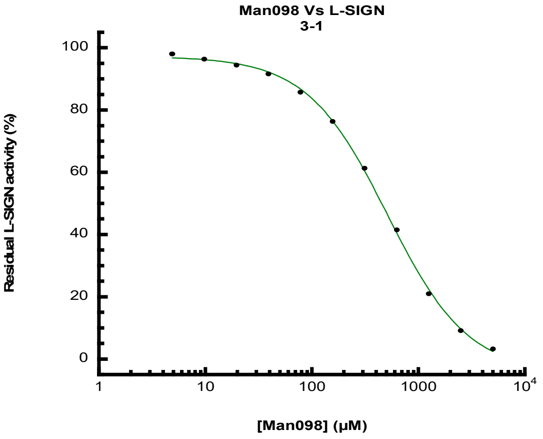

L-SIGN/ 4 (Man96)

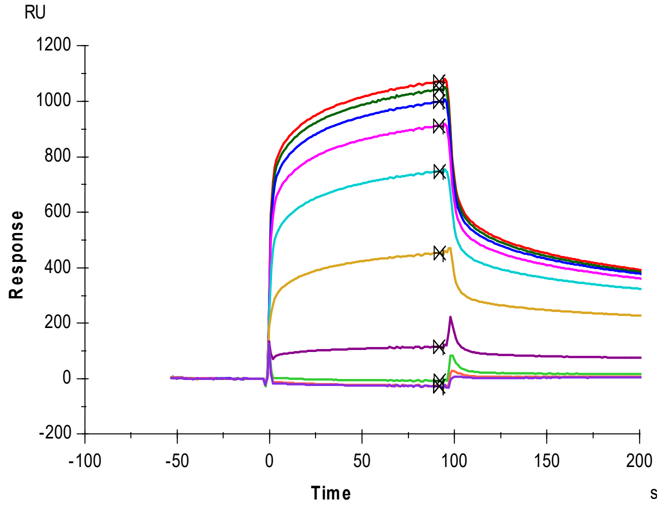

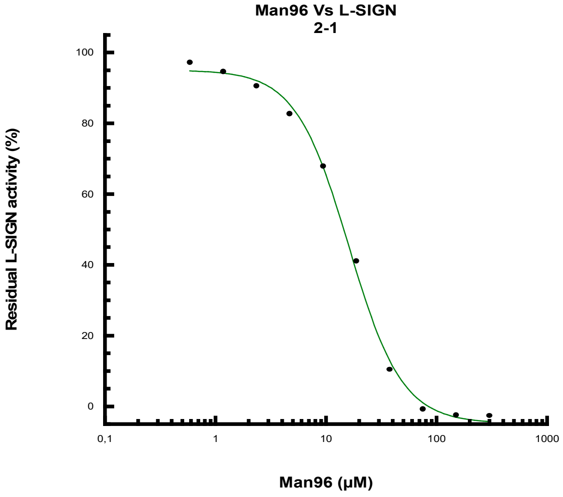

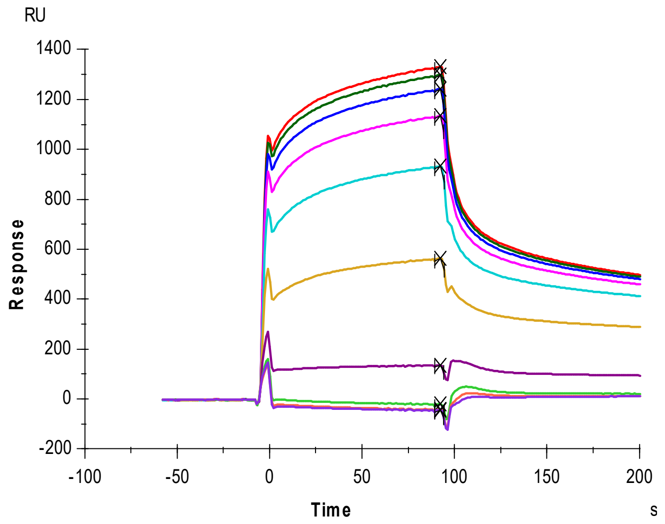

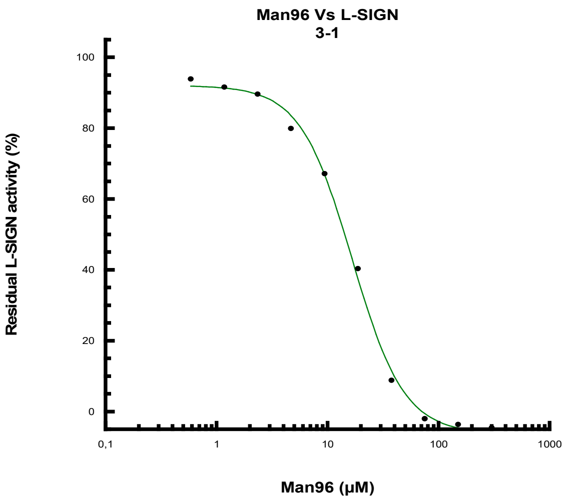

L-SIGN/ 5 (Man102)

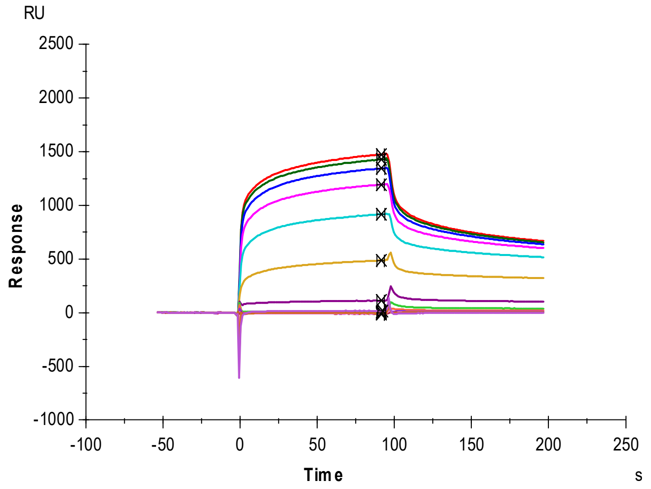

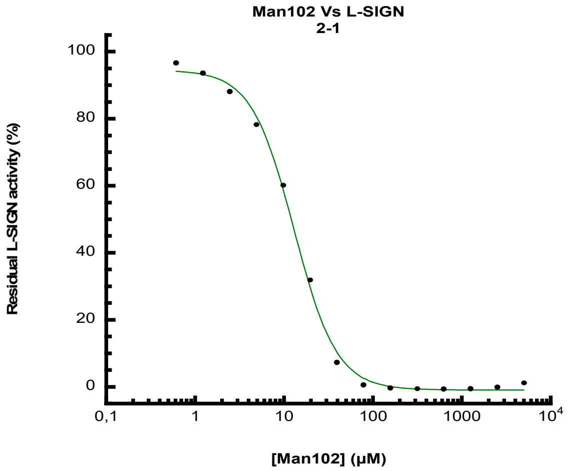

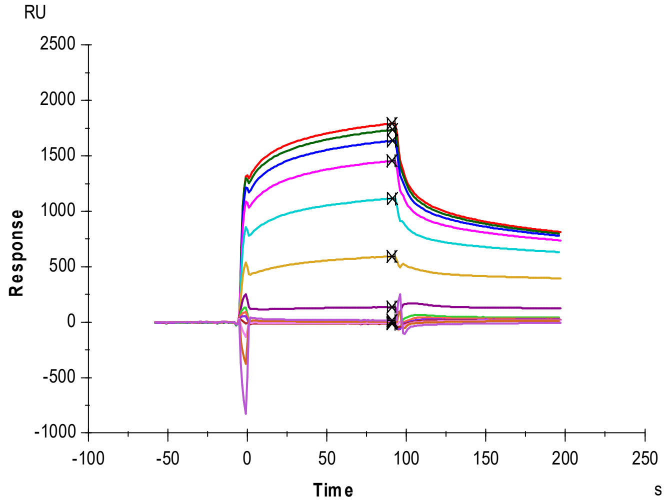

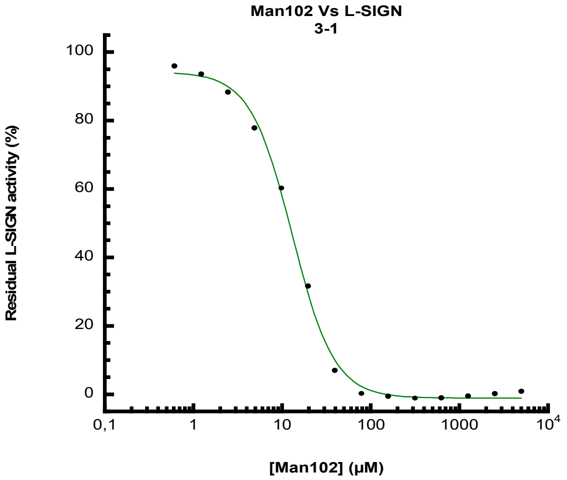

L-SIGN/ 6 (Man100)

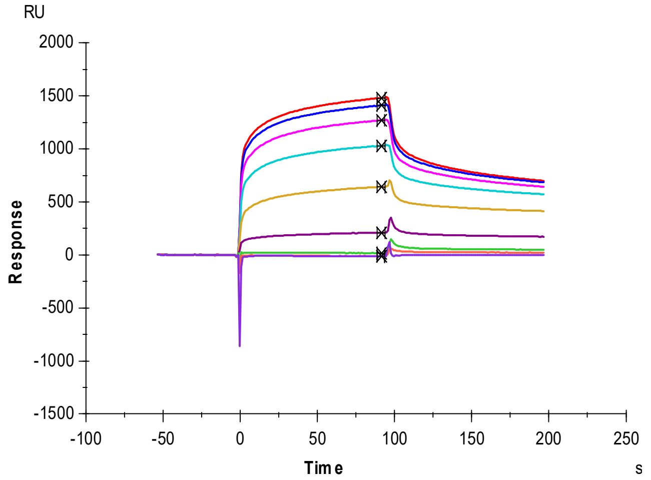

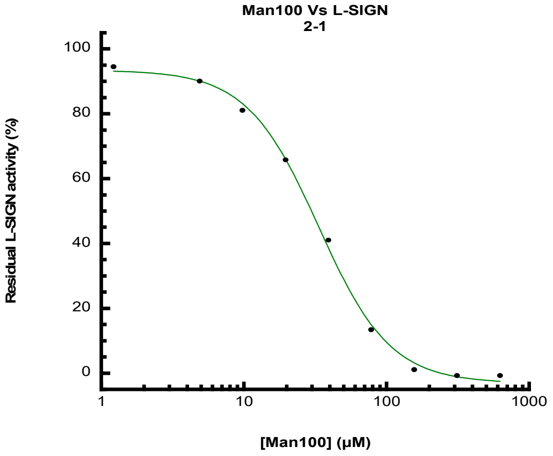

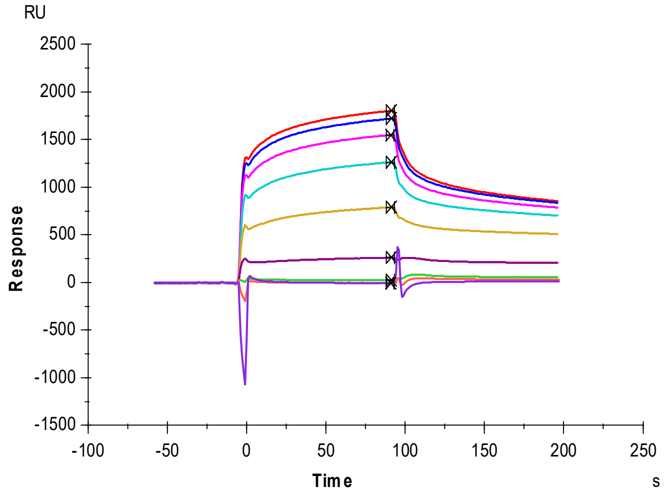

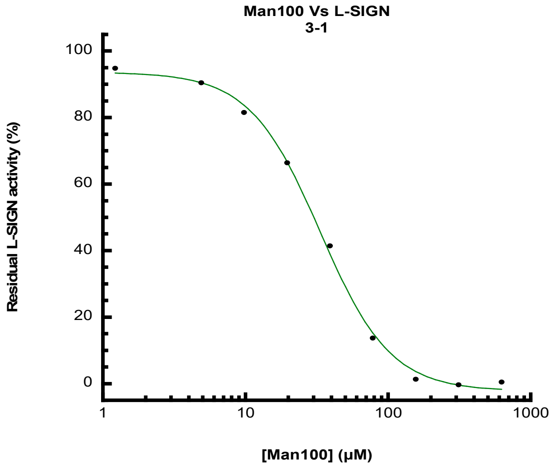

L-SIGN/ 7 (Man101)

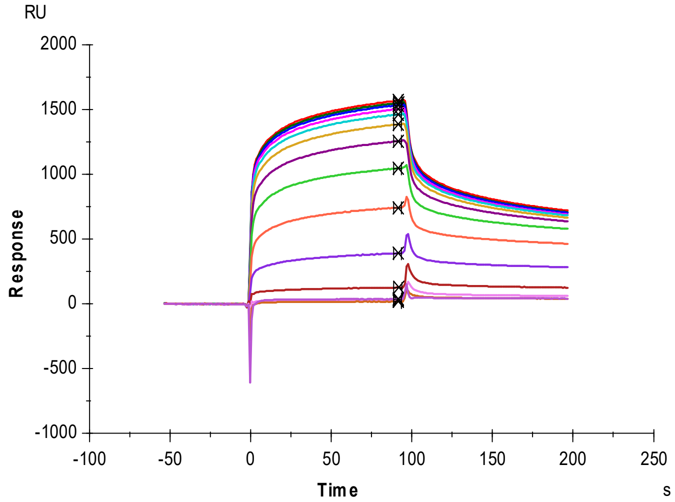

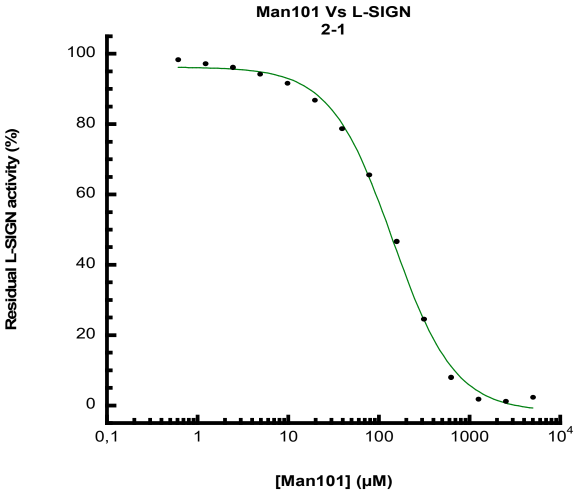

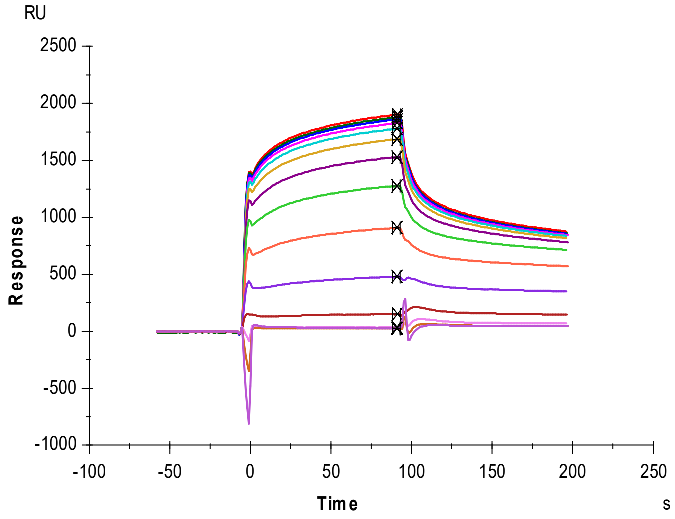

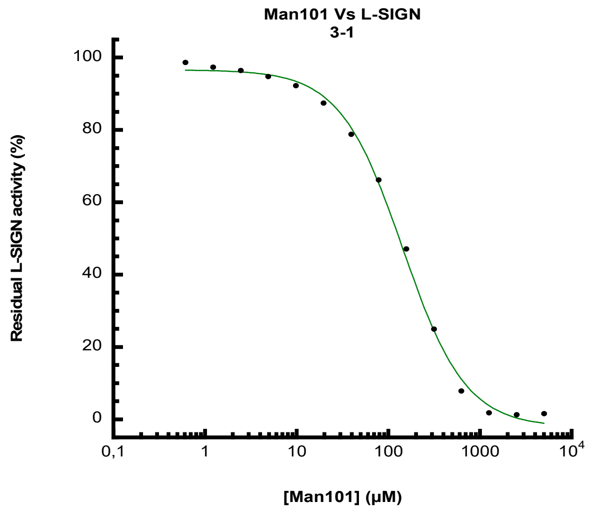

L-SIGN/ 8 (Man99)

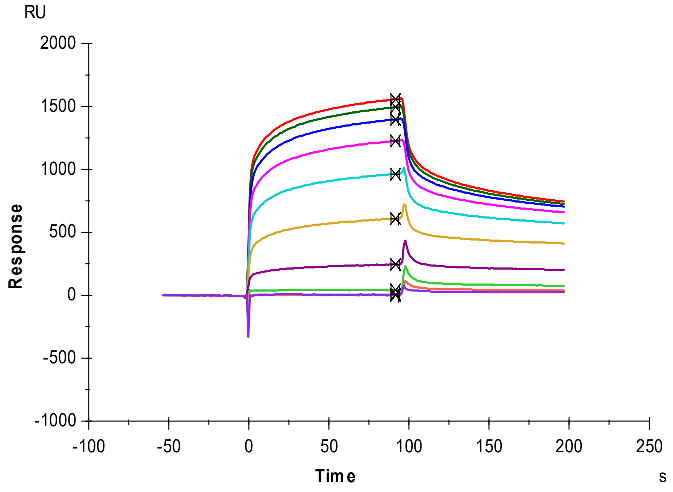

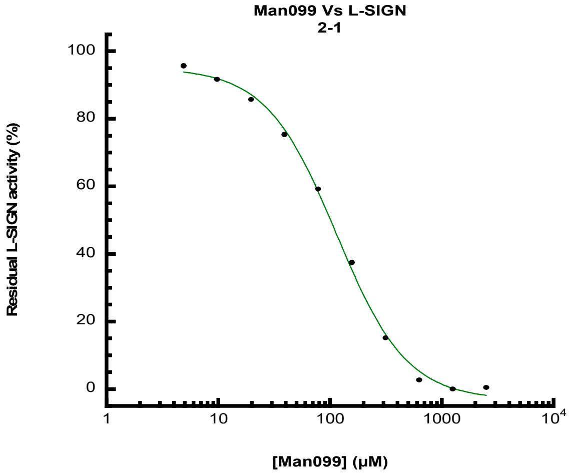

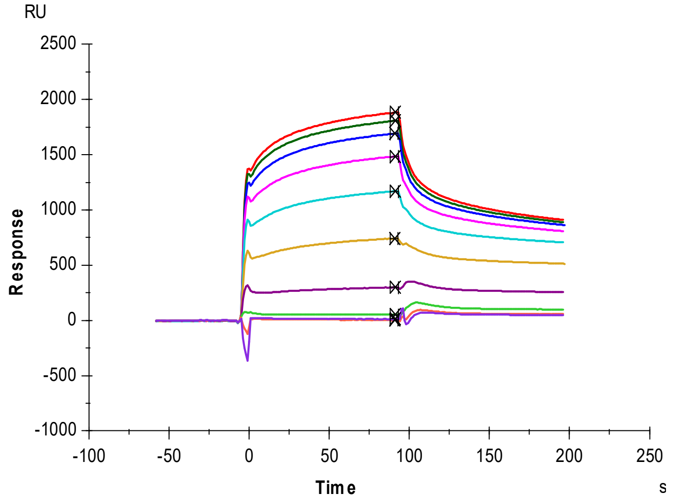

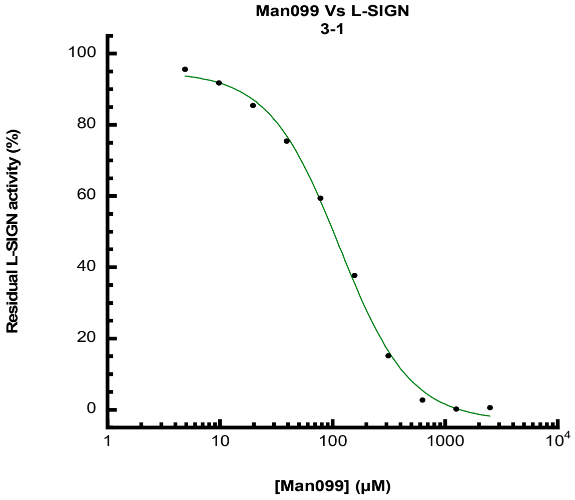

L-SIGN/ 9 (Man97)

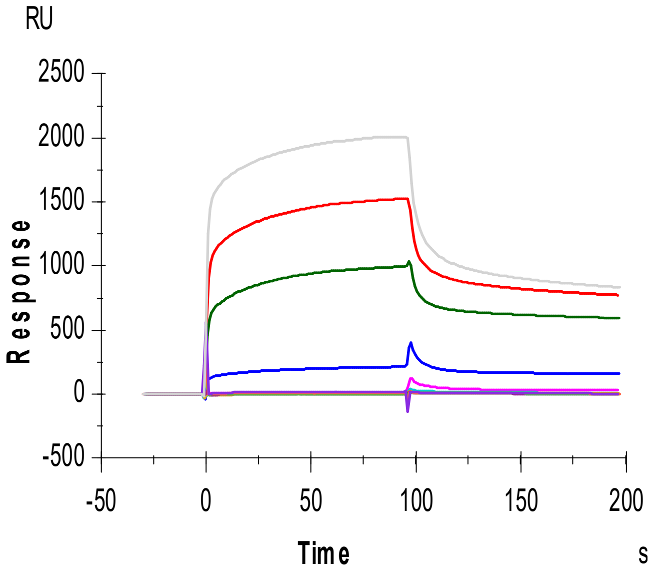

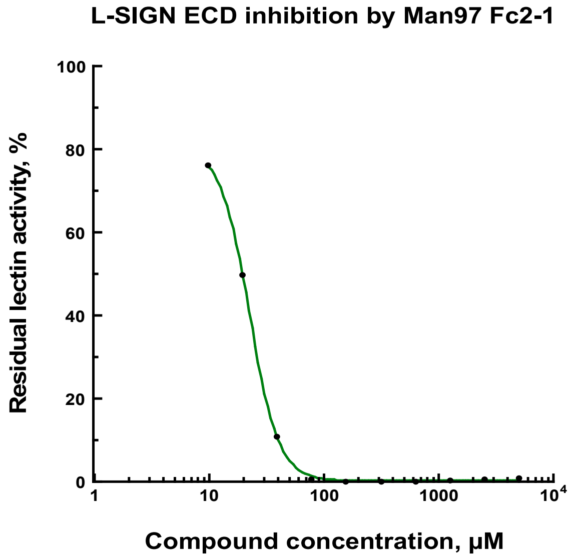

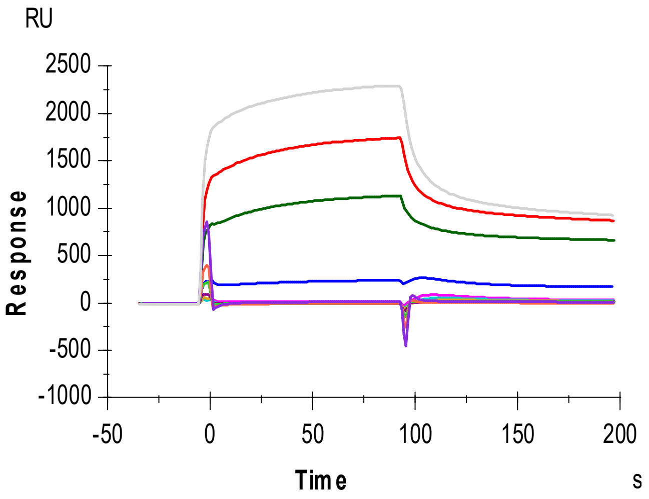

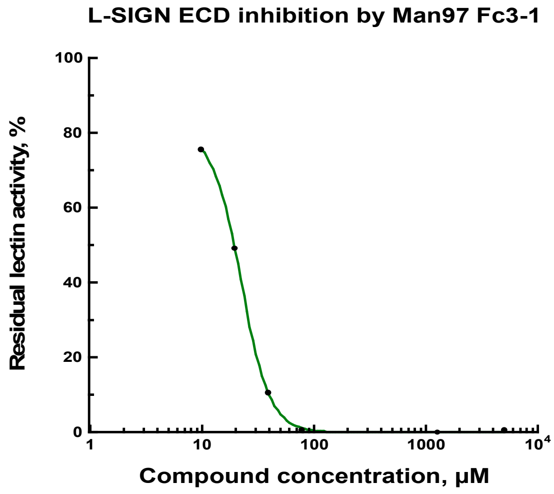

L-SIGN/ 10 (Man94)

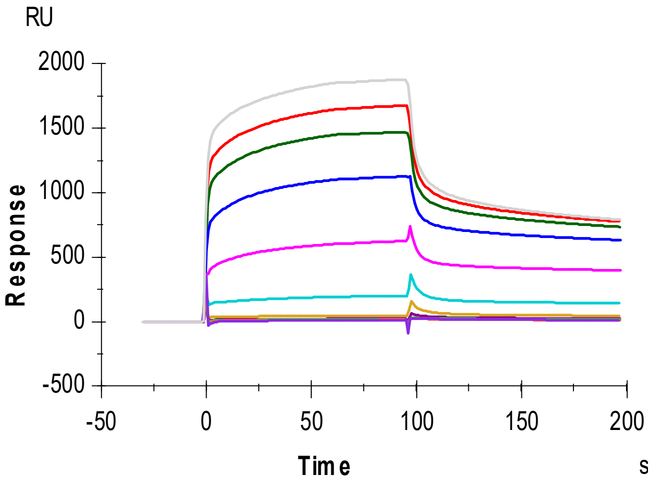

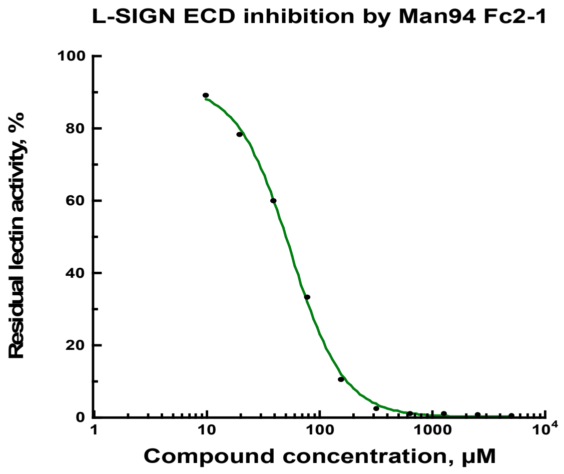

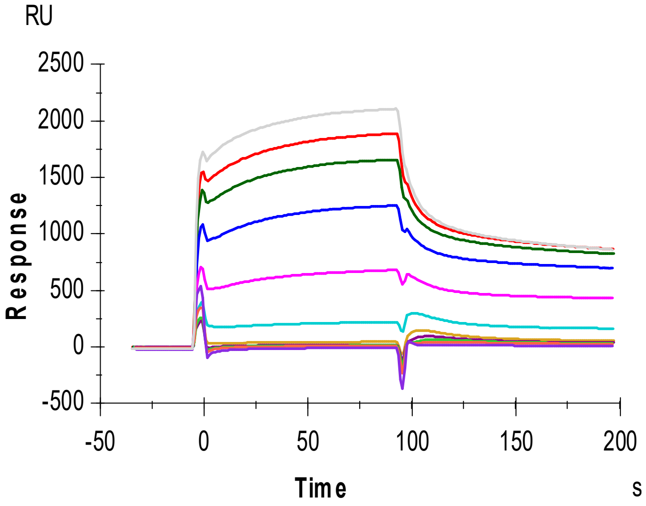

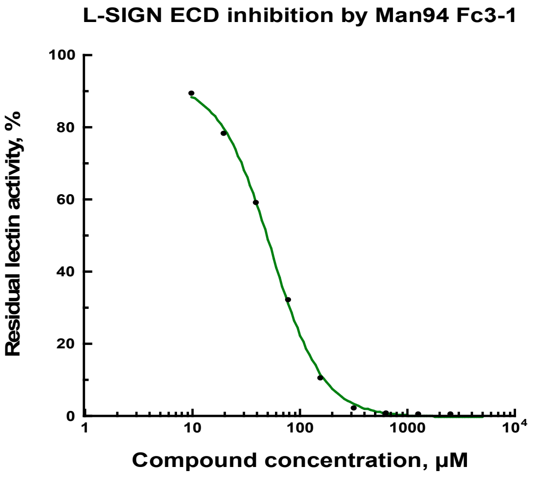

L-SIGN/ 11 (Man103)

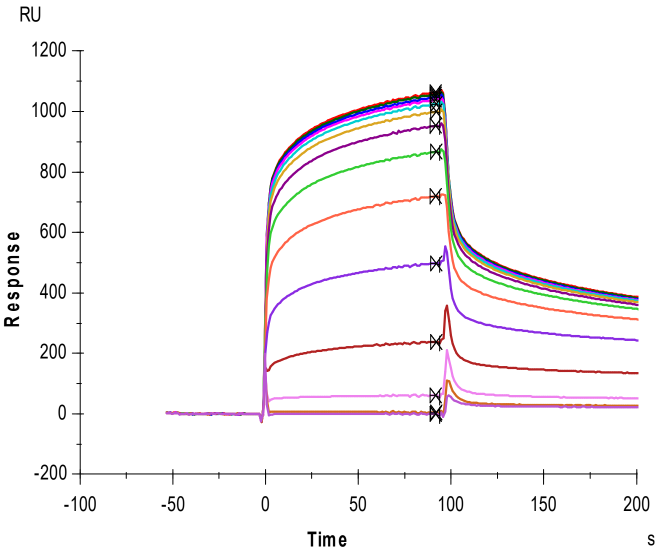

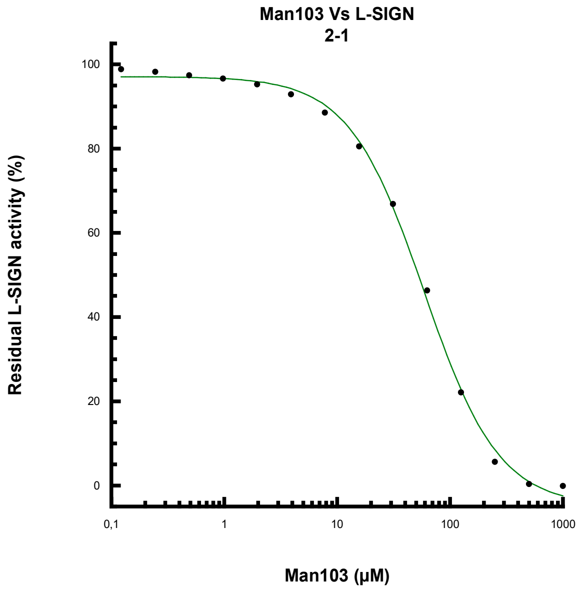

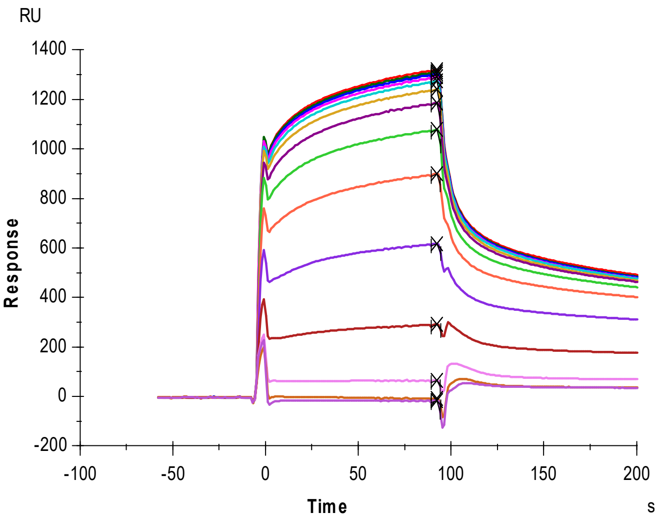

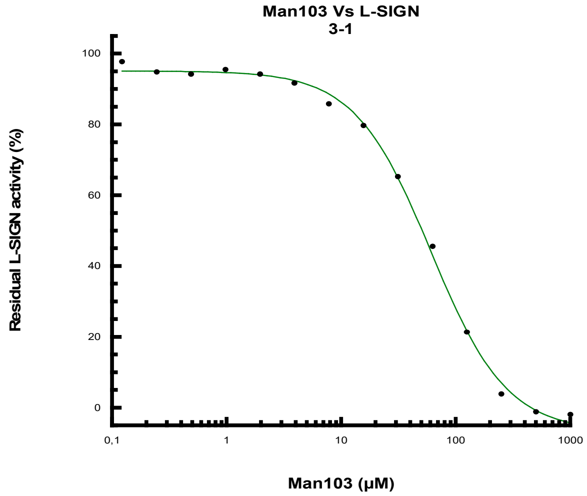

Aggregation studies

As reported in the experimental section of the article, all the ligands were tested at 5 mM as the highest concentration by SPR. In these conditions, it is not unlikely that aggregation phenomena could occur, in particular for ligands **2**-**3**, **8**-**11**, where π-π interactions among the aromatic guanidine isosteric moieties might occur. Thus, following the Reviewer's suggestion, we performed a ^{1}H-NMR analysis of compound **10** by acquiring ^{1}H spectra at different concentrations (5 mM and 1.25 mM in D_2O; NS=320; D1=2.000s; excitation sculpting at 4.700 ppm). We found no evidence of aggregation: no alterations were observed for the chemical shifts or for the line width of the proton signals and no concentration dependency of the integrals appeared to occur. The spectra are reported below.

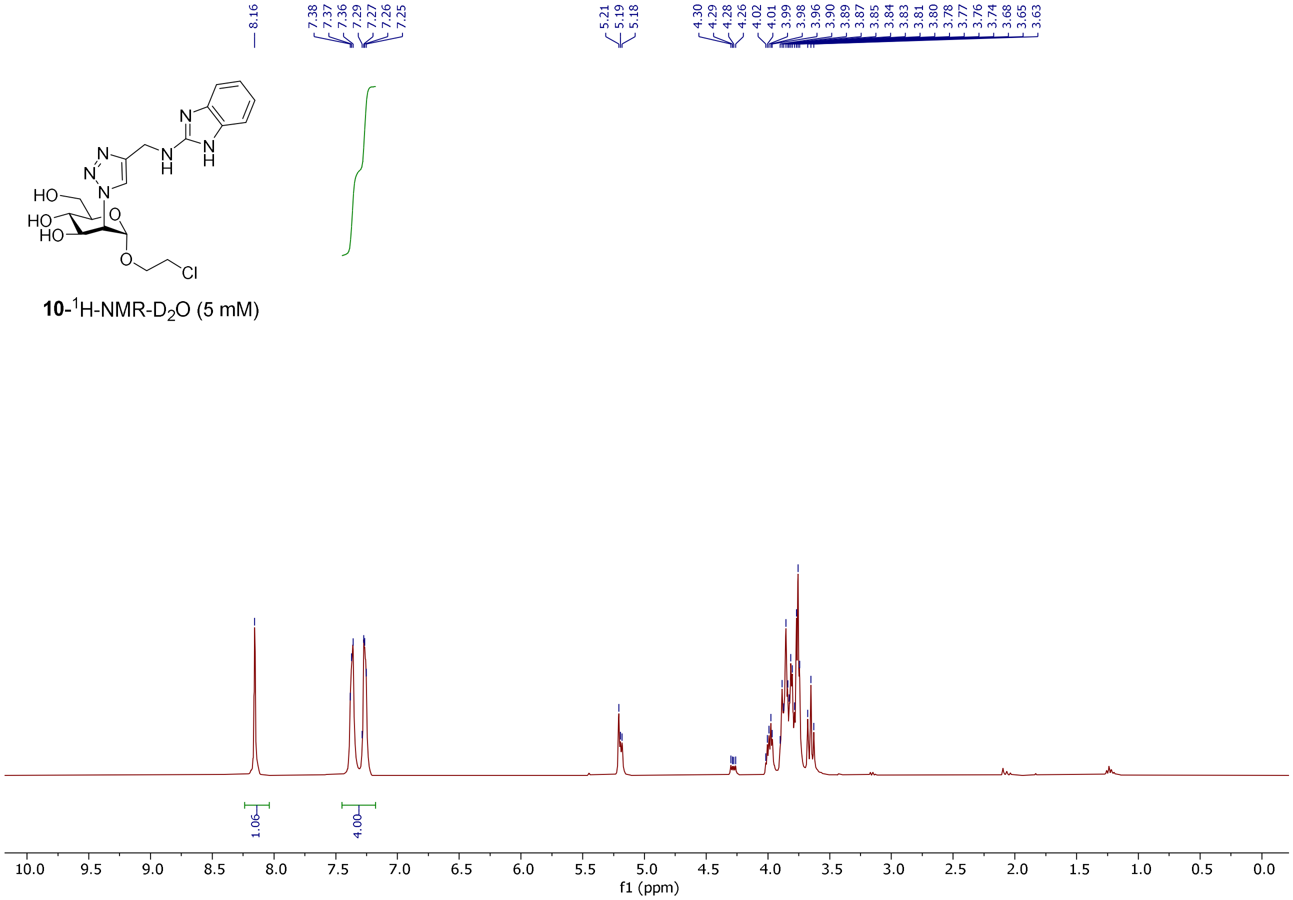

10-^{1}H-NMR-D_2O (5 mM)

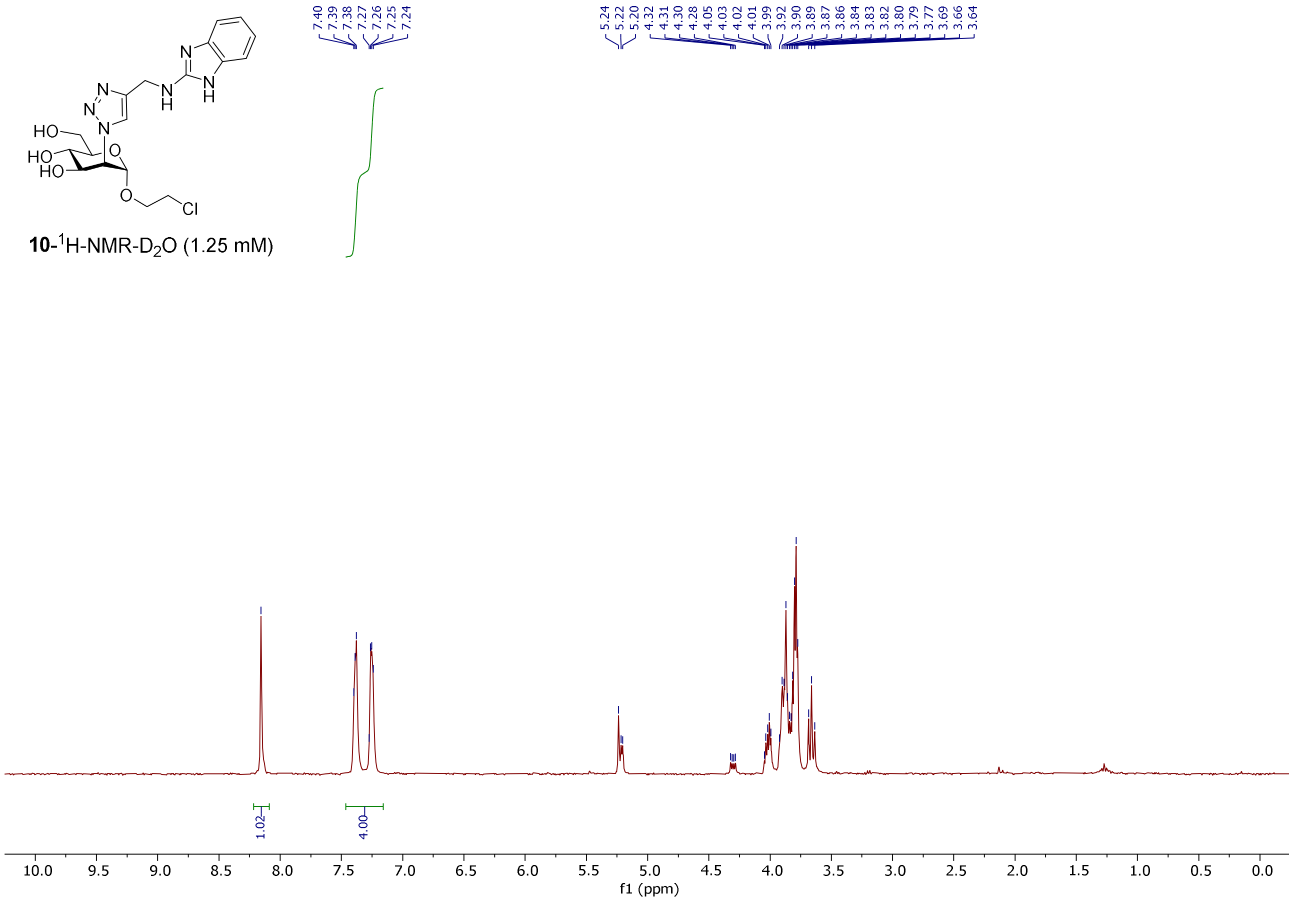
7.40
7.39
7.38
7.27
7.26
7.25
7.24
5.24
5.22
5.20
4.32
4.31
4.30
4.28
4.05
4.03
4.02
4.01
3.99
3.92
3.90
3.89
3.87
3.86
3.84
3.83
3.82
3.80
3.79
3.77
3.69
3.66
3.64
10-1H-NMR-D2O (1.25 mM)
1.02
4.00
10.0 9.5 9.0 8.5 8.0 7.5 7.0 6.5 6.0 5.5 5.0 4.5 4.0 3.5 3.0 2.5 2.0 1.5 1.0 0.5 0.0
f1 (ppm)

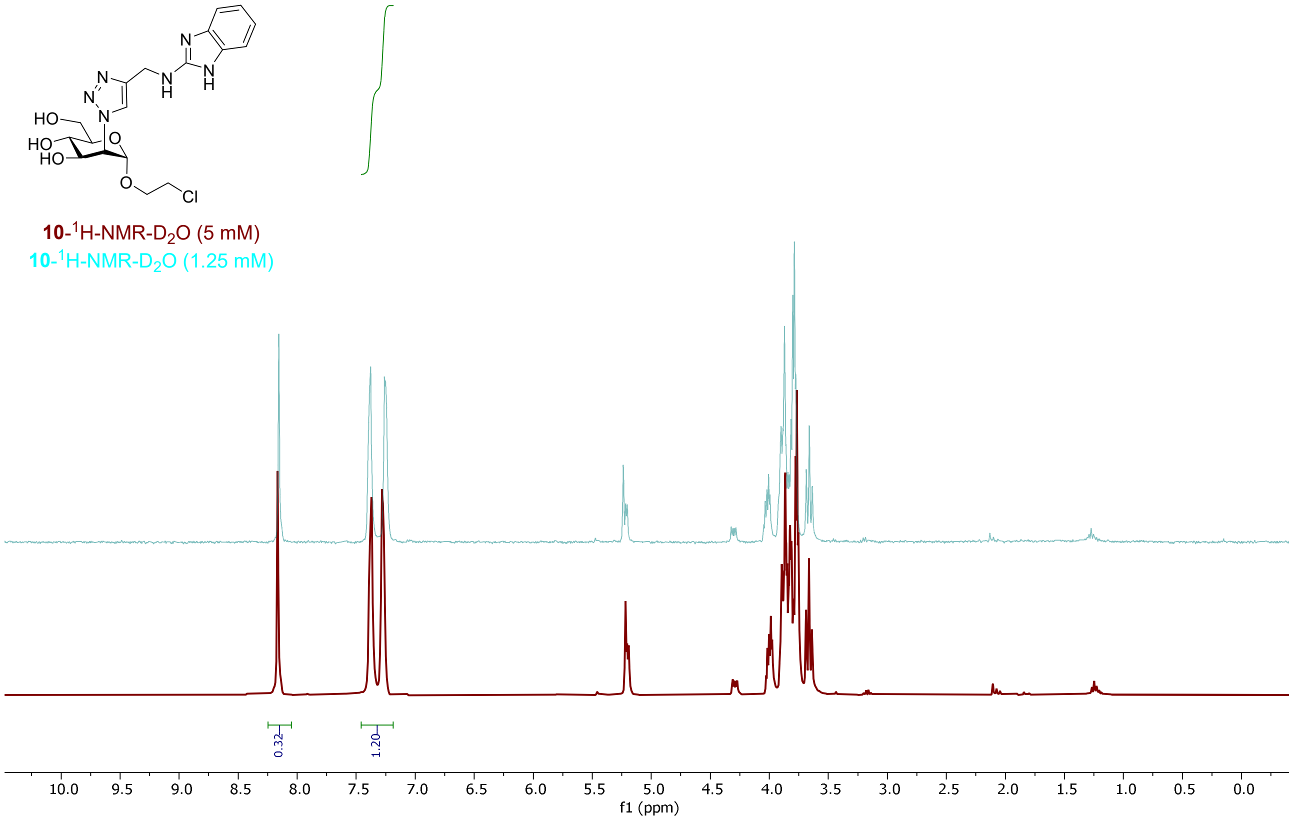
10-1H-NMR-D2O (5 mM)
10-1H-NMR-D2O (1.25 mM)
0.32
1.20
10.0 9.5 9.0 8.5 8.0 7.5 7.0 6.5 6.0 5.5 5.0 4.5 4.0 3.5 3.0 2.5 2.0 1.5 1.0 0.5 0.0
f1 (ppm)

Affinity and selectivity correlation graphs

pKa values were estimated for the conjugated acid of the heterocyclic fragment by Epik, v. 4.3011 (Schrodinger 2018)

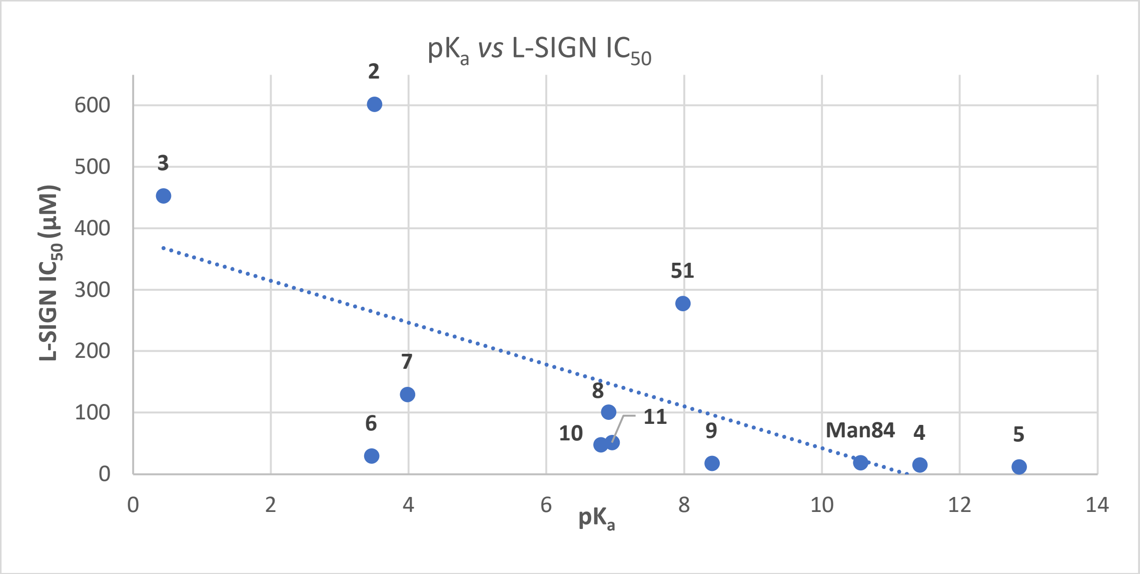

Figure SI-1. Correlation of L-SIGN affinity (IC_{50} in SPR inhibition experiments) with pK_a for all ligands.

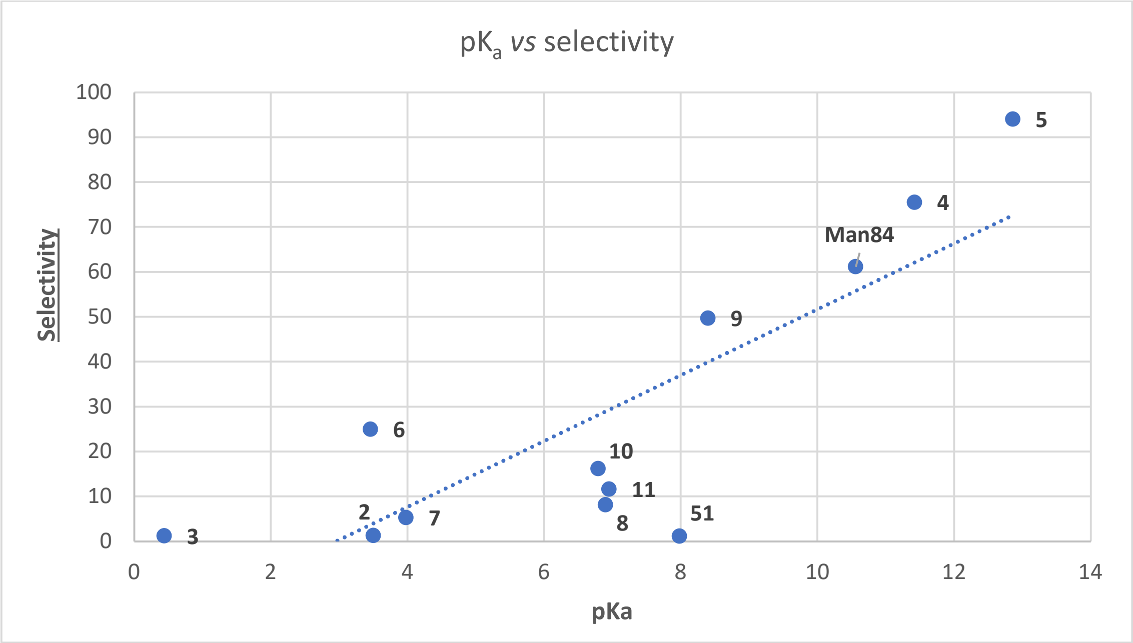

Figure SI-2. Correlation of L-SIGN *vs* DC-SIGN selectivity with pK_a for all ligands.

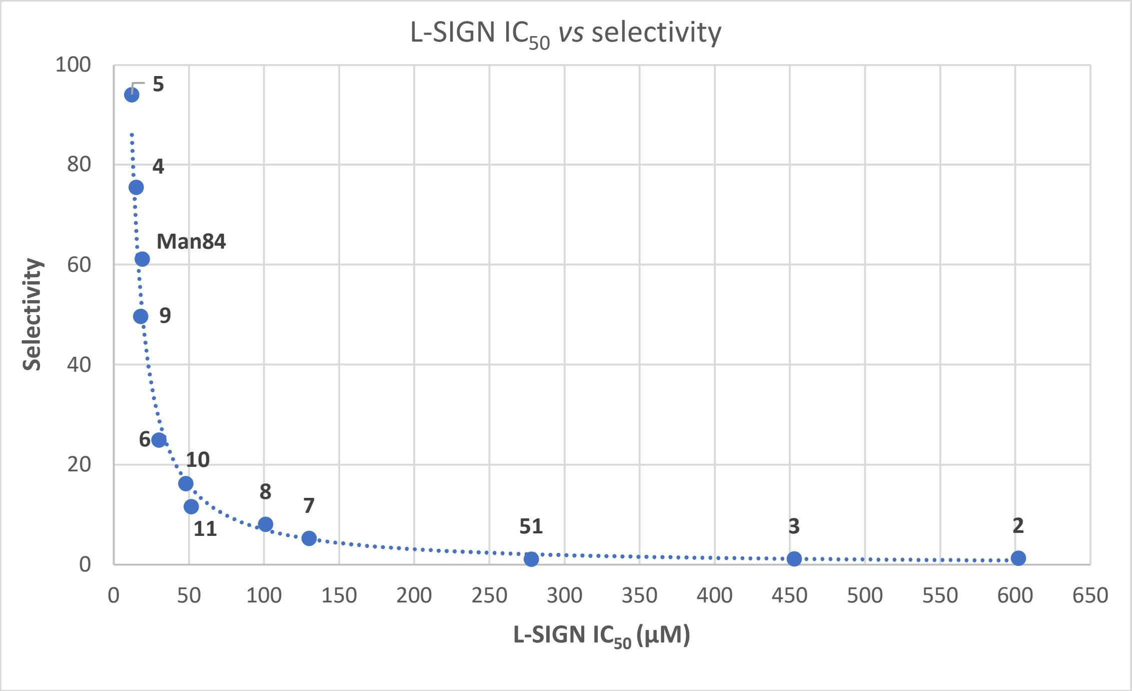

Figure SI-3. Correlation of L-SIGN *vs* DC-SIGN selectivity with L-SIGN IC_{50} for all ligands.

Table SI-2. Crystallographic data and statistics of L-SIGN CRD/4 complex

Table SI-2. Data collection statistics from XSCALE and XDSCONV,[1] refinement statistics from REFMAC 5 and COOT. Values in brackets are for the highest resolution shell.

**Data collection:**	
Space group:	$P6_5$, No.170
Cell a, b, c (Å):	105.72, 105.72, 59.11
Cell α, β, γ (°):	90, 90, 120
Resolution (Å):	49.6-2.0 (2.05-2.00)
$CC_{1/2}$:	99.0 (73.3)
R-factor (%):	12.7 (81.3)
I/σ:	4.66 (1.09)
Completeness (%):	96.9 (99.0)
No of reflections	69433/24815/25602
**Refinement:**	
Resolution (Å):	49.66-2.0
No. of reflections:	23574
R / R_{free}:	0.2275 / 0.2899
Mean B value (Å^2):	41.0
R.m.s.d lengths (Å):	0.0080
R.m.s.d angles (°):	1.4480
Ramachandran favored (%):	90.48
Ramachandran outliers (%):	1.19

Computational studies

Docking calculations were performed using the Schrödinger Suite through Maestro graphical interface (Schrödinger Release 2018-1). Docking protocols were developed using the software Glide and the OPLS3 force field using as a starting structure the complex L-SIGN CRD/ **Man84** (PDB code: 8RCY) and a co-crystal of DC-SIGN CRD (PDB code: 6GHV). The models were applied to rationalize the structural motifs that confer binding affinity and selectivity to the ligands. Particular attention was paid to ligands that may exist in different charge and tautomeric states.

Ligand preparation

The glycomimetic ligands were prepared for docking using the LigPrep tool (version 45011) to create energy minimized 3D structures for the most likely charge and tautomeric states. The protonation states were generated at pH 7±2 and then employed in computational studies. Neutral basic moieties were suggested for ligands **2**, **3**, **6**, **7**; protonated isosteric groups were suggested as the most favorable protonation states for ligands **Man84**, **4**, **5**, **9**. Both neutral and protonated forms were proposed by the tool for ligands **8**, **10** and **11**. The assignment of protonation states was further controlled by calculating the pKa values for the conjugated acid of the heterocyclic fragment by Epik version 4.3011.

Relevant alternative tautomeric states were identified and considered for ligands **6** and **7** (**Figure SI-4**).

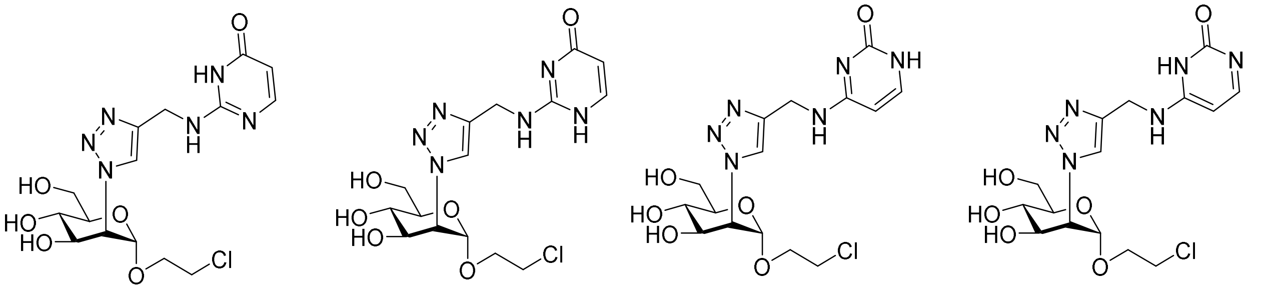

6 (tautomer a) **6** (tautomer b) **7** (tautomer a) **7** (tautomer b)

Figure SI- 4. Tautomeric states considered for ligands **6** and **7**.

Protein preparation

Atomic coordinates from the crystal structure of L-SIGN CRD in complex with **Man84** (PDB code: 8RCY) were taken from the Protein Data Bank. The system (134 residues, residue sequence 266-399) was prepared using the Protein Preparation Wizard of the Maestro graphical user interface. All water molecules were removed. The hydrogen atoms were added and pKa was calculated for protein residues using the PROPKA method[2] at pH 7.4. Then, the protein-ligand complex was subjected to restrained minimization with convergence of heavy atoms to an RMSD of 0.3 Å using the OPLS3 force field.[3] The final structure was used to generate the grid for docking calculations.

Docking calculations

Docking calculations were performed using Glide (Grid-based Ligand Docking with Energetics)[4] version 78011.

The centroid of the ligand **Man84** was used to define a cubic grid inner box with dimensions 10 Å and a cubic grid outer box with dimensions 30 Å. Docking calculations were carried out applying the flexible docking approach and employing the extra precision (XP) scoring function with the OPLS3 force field. Ring and nitrogen-inversion sampling were disabled. No Epik state penalties were added to the final docking scores.

The ligand **Man84** was redocked at the sugar binding site. The program reproduced the co-crystallized binding mode of the ligand with an RMSD value of 0.19 Å (heavy atoms, excluding C1 and C5 side chains, 0.63 Å all heavy atoms), thus validating the docking protocol. The C2-modified mono-mannoside glycomimetics **2-11** and **51** were analyzed employing the same docking protocol and saving at most 10 poses using the XP scoring function. The geometric criteria for displaying protein-ligand H-bonds in **Figure SI-5** and **SI-6a,b** are the following ones: a distance ≤ 2.5 Å between donor H atom and acceptor atoms (D—H···A); a donor angle ≥ 120° between the donor-hydrogen-acceptor atoms (D—H···A); an acceptor angle ≥ 90°between the hydrogen-acceptor-bonded_atom atoms (H···A—X).

Results

The selectivity towards L-SIGN appears to be driven by bulky basic groups, such as guanidine or its isosteres, that unfavourably interact with the bulky positively charged side chain of Lys373 in DC-SIGN, replaced by Asn385 in L-SIGN. Ligands showing the highest affinity and selectivity for L-SIGN present docking poses characterized by a bidentate hydrogen bond interaction between the basic group and the side chain of Glu370 in L-SIGN, as observed in the X-ray structure of **Man84** in complex with L-SIGN. Binding may be further enhanced by electrostatic interactions (and by cation-π interactions with Phe325) if the basic group is protonated under physiological conditions, as in the case of ligands **Man84**, **4**, **5**, **9** and, to a lesser extent, of ligands **8**, **10** and **11**, for which an equilibrium between neutral and charged forms is predicted. Interestingly, only the protonated form of ligand **8** can establish the bidentate hydrogen bond interaction with Glu370. Additional stabilizing interactions are observed in the docking poses of the best L-SIGN ligands, namely H-bonds between the triazole ring (HBA) and Asn379 side chain (HBD) and between the basic moiety (HBD) and Asn385 side chain (HBA).

The key bidentate H-bond interaction and also other stabilizing interactions are not available for ligands **2** and **3** (lacking a second NH in the neutral form) that only show interactions of the mannose core with calcium ion and calcium-coordinating residues. Finally, binding of **6** and **7** towards L-SIGN can be discussed by considering the docking poses of suitable tautomers (**Figure SI-5**). In particular, the steric hindrance exerted by the carbonyl group when ligand **6** adopts the bidentate H-bond interaction with Glu370 can be relieved using tautomer b which provides optimal docking poses in L-SIGN, showing all the key interactions available to a neutral ligand (**Figure SI-5, top**). The best poses of ligand **7** are provided by tautomer b as well, although the bidentate interaction is replaced by simple H-bond interactions with Glu370 and Asn385, and a triazole ring flipping is observed (**Figure SI-5, bottom**).

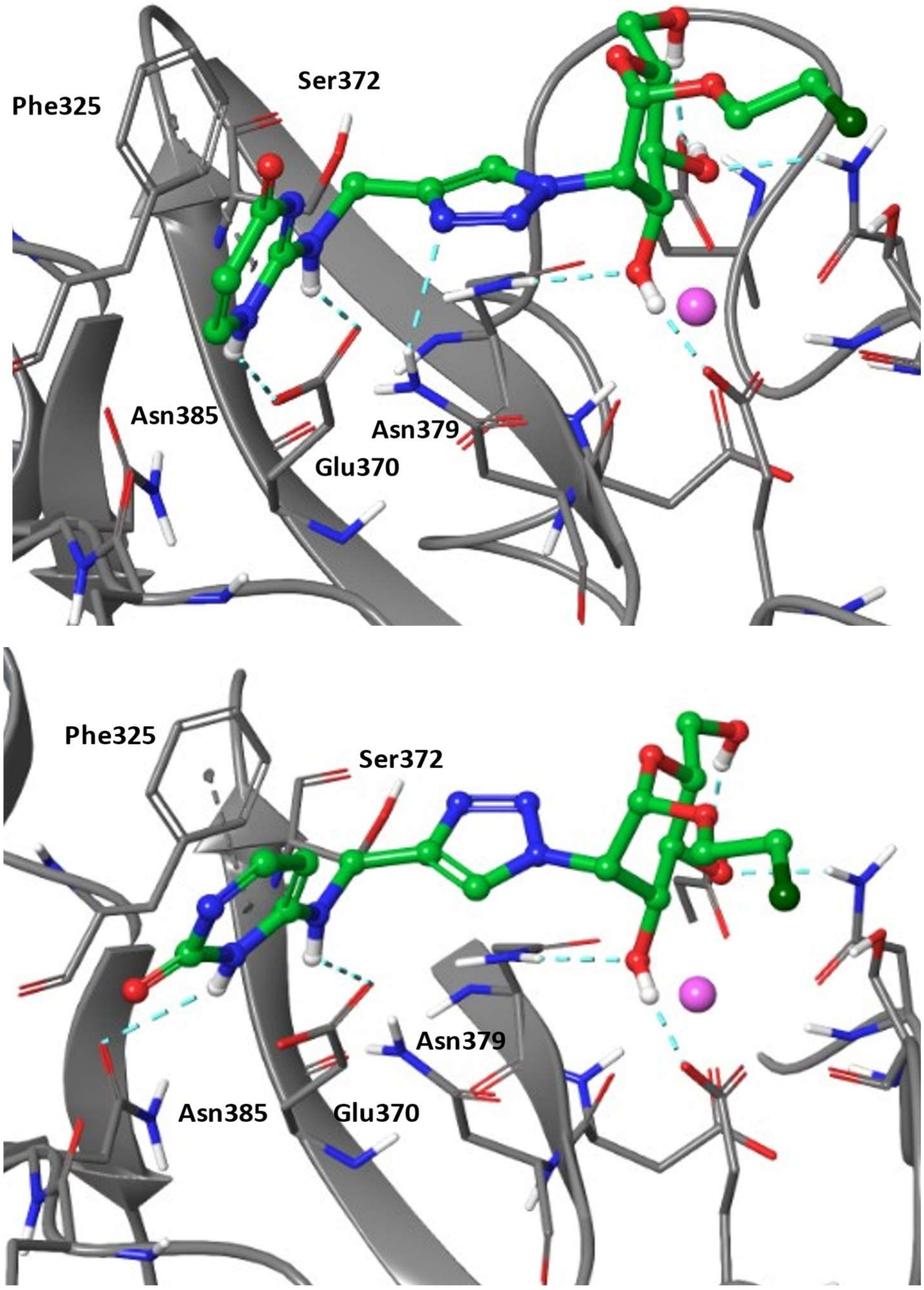

Figure SI-5. Best docking pose of ligand **6** (top, tautomer b) and ligand **7** (bottom, tautomer b) into L-SIGN (8RCY pdb). Only selected lectin residues involved in interactions with the ligand are shown as grey sticks. The calcium ion is represented as a pink CPK sphere. Non-polar hydrogens are hidden for clarity; intermolecular hydrogen bonds are shown as aqua dashed lines.

For compound **51**, which binds unselectively to both lectins, docking studies in L-SIGN (**Figure SI-6a**) suggest a binding mode whereby the protonated amino group binds to the triad E370, F325, S372 (E358, F313, S360 in DC-SIGN, **Figure SI-6b**) forming an ion-pair interaction, a cation-pi interaction and H-bond interactions. This is consistent with the X-ray obtained for the DC-SIGN complex of **Man69** (**Figure SI-6c**), a ligand previously reported by our groups[5] that contains the modified mannose ring of **51** as part of a larger structure. The lack of a bidentate H-bond interaction between the ammonium ion of **51** and the carboxylate of the conserved glutamic acid is the cause of the (relatively) low affinity (ca. 300 µM) of this ligand in both proteins. The

conserved E, F, S triad in DC-SIGN and L-SIGN binding sites results in a very similar mode of binding for **51** in both lectins, explaining the absence of specificity.

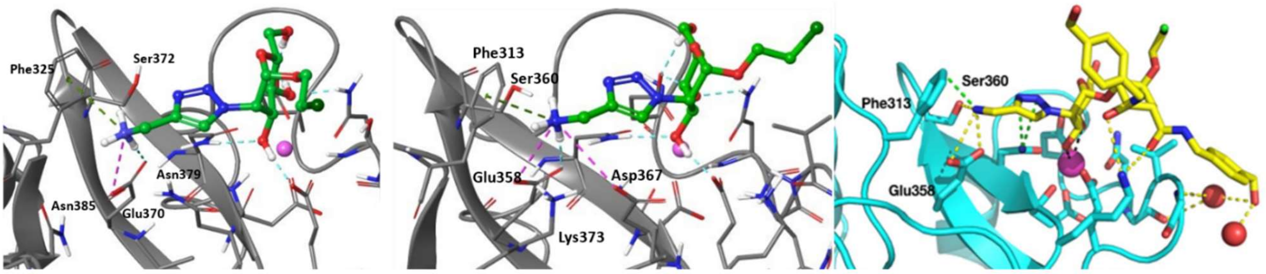

a) b) c)

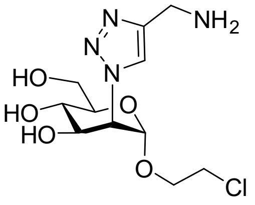

51

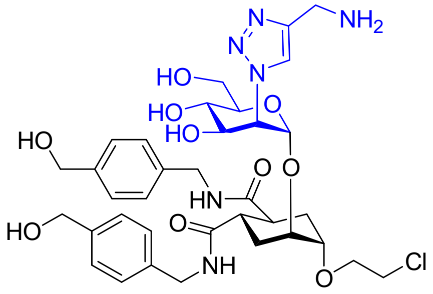

Man69

Figure SI-6. a) Docking of **51**(Man79) in L-SIGN (8RCY); b) Docking of **51**(Man79) in DC-SIGN (6GHV); c) X-ray structure of **Man69** in DC-SIGN (6GHV)[5]

Spectral data for 2-21

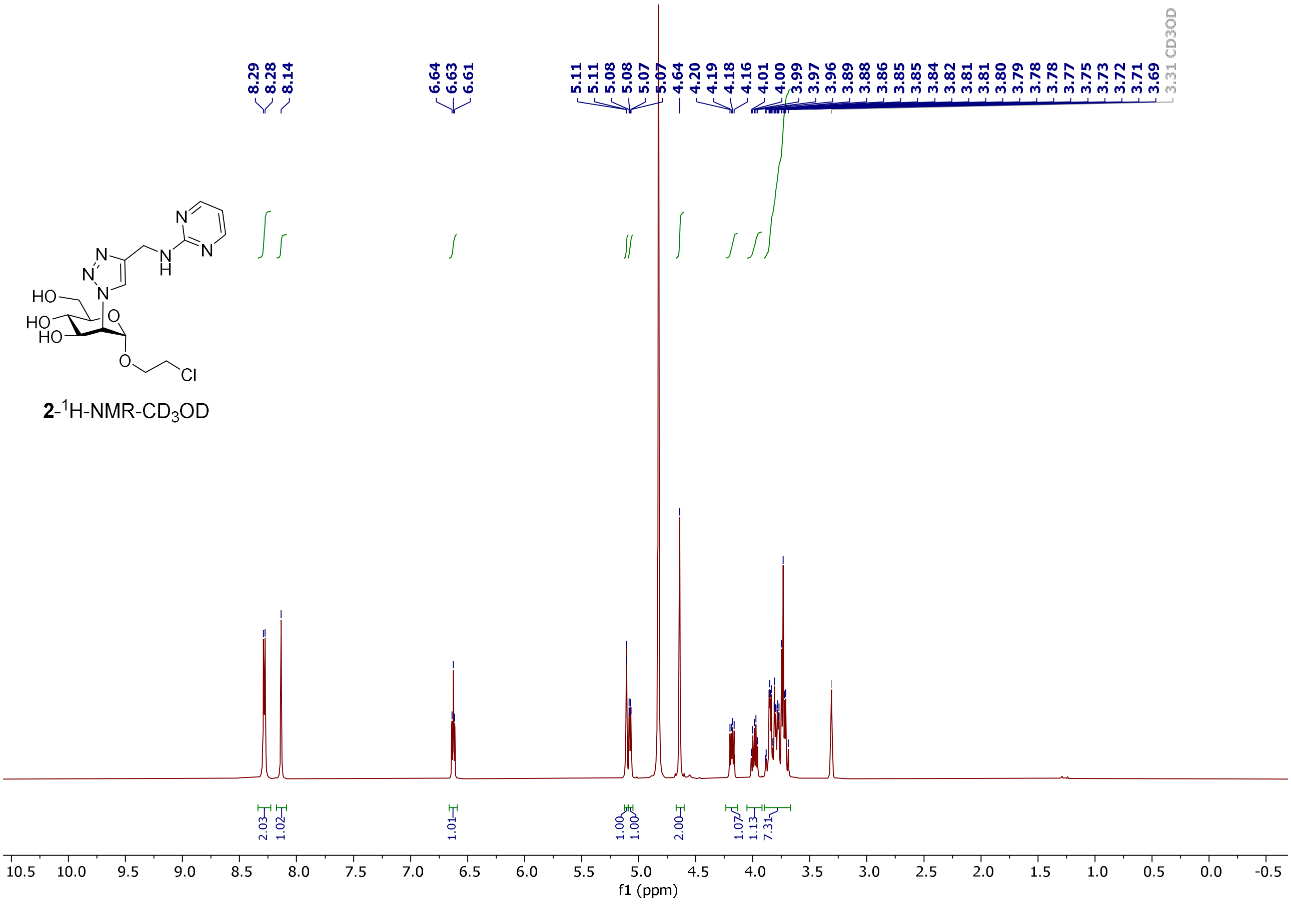

2-^{1}H-NMR-CD_3OD

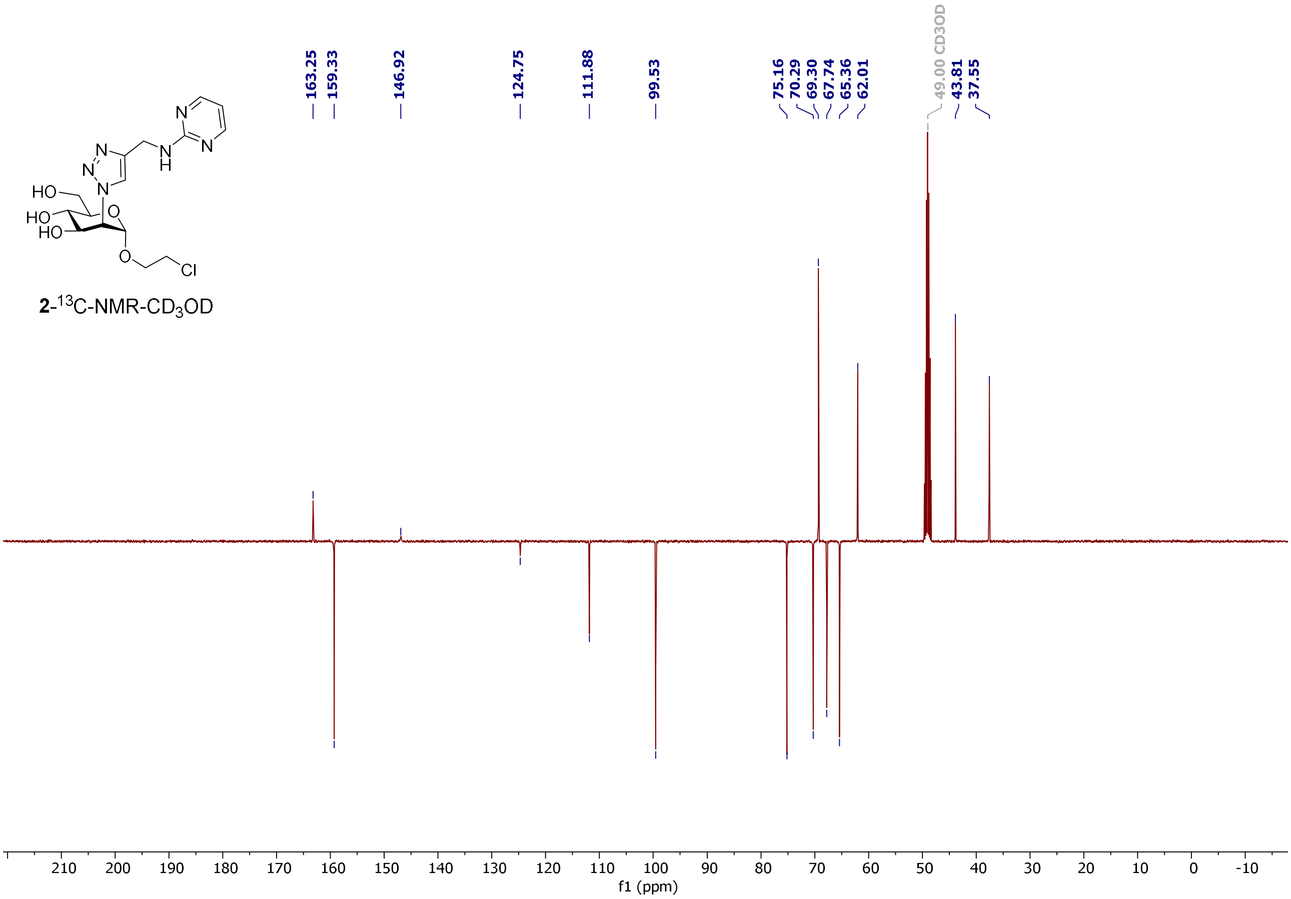

2-^{13}C-NMR-CD_3OD

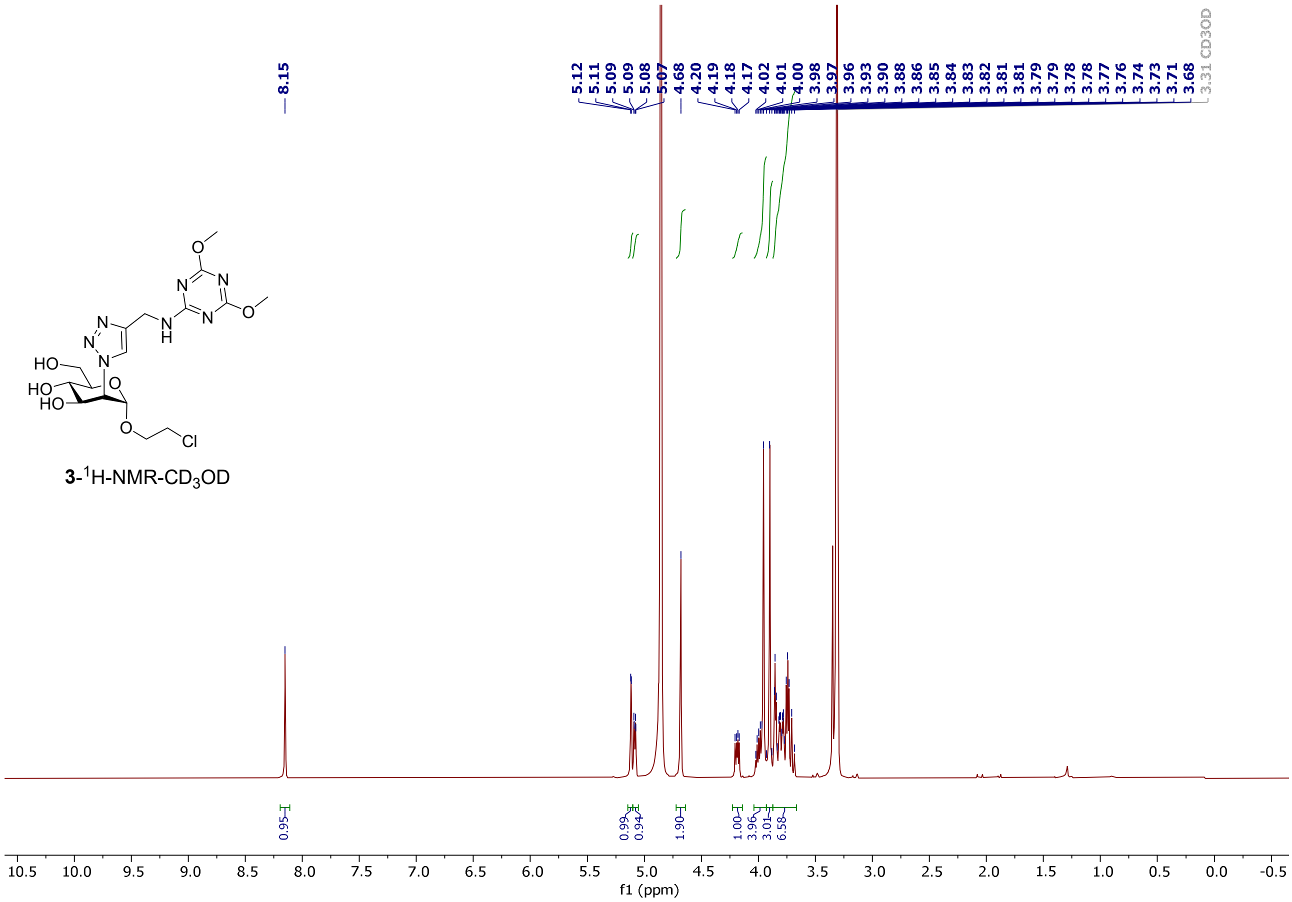

3-^{1}H-NMR-CD_3OD

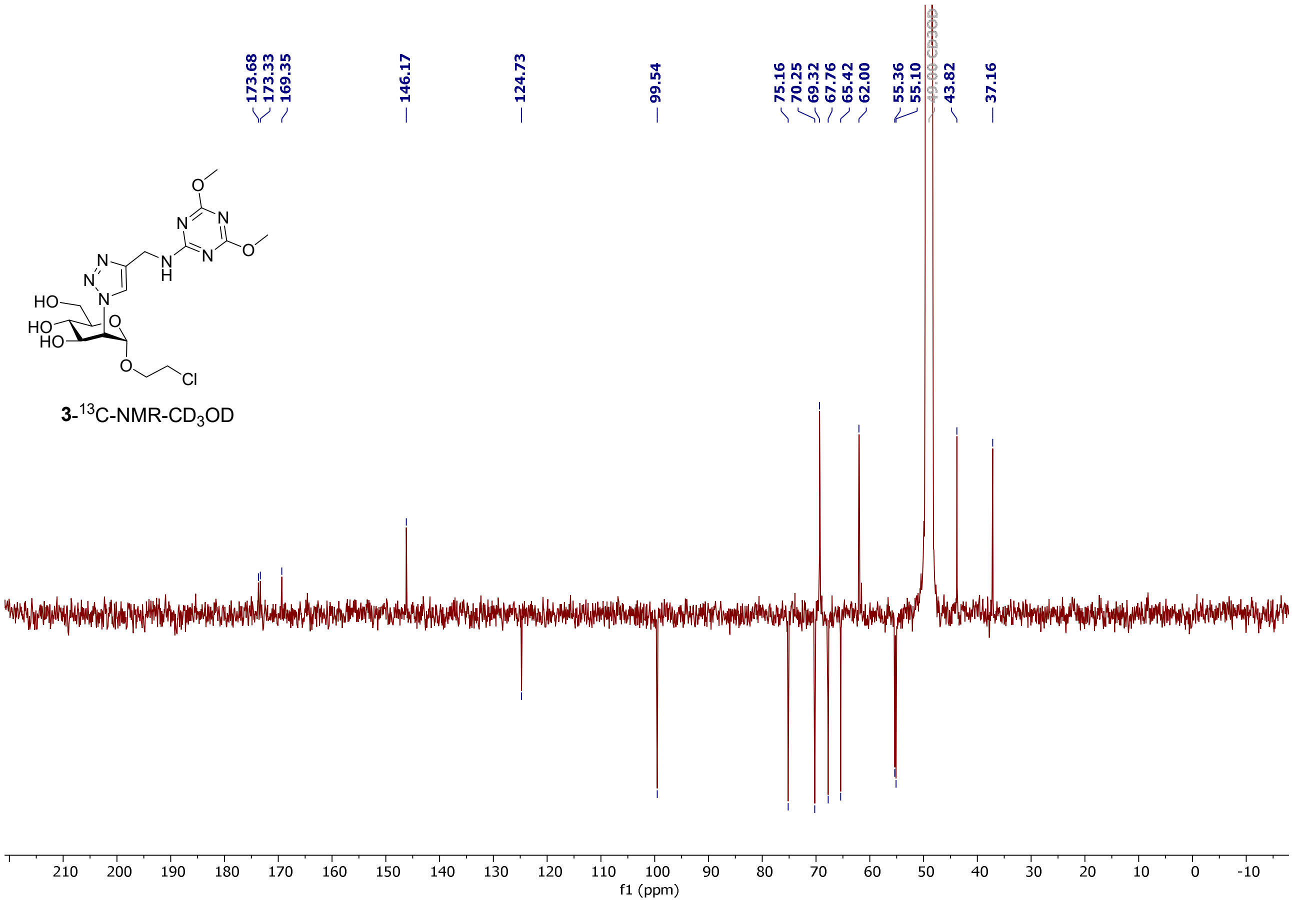

3-^{13}C-NMR-CD_3OD

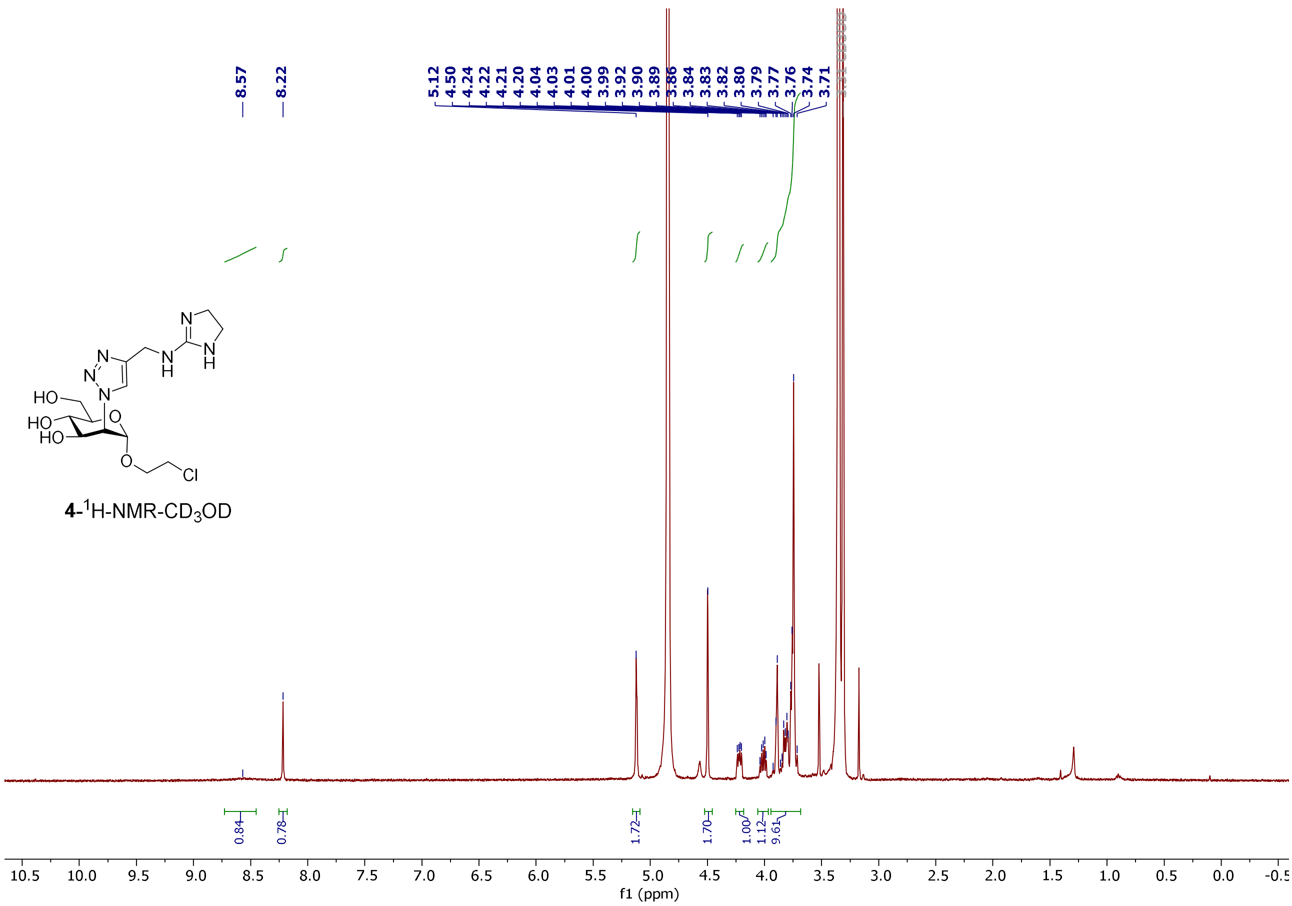
8.57
8.22
5.12
4.50
4.24
4.22
4.21
4.20
4.04
4.03
4.01
4.00
3.99
3.92
3.90
3.89
3.86
3.84
3.83
3.82
3.80
3.79
3.77
3.76
3.74
3.71
0.84
0.78
1.72
1.70
1.00
1.12
9.61
4-1H-NMR-CD3OD
f1 (ppm)

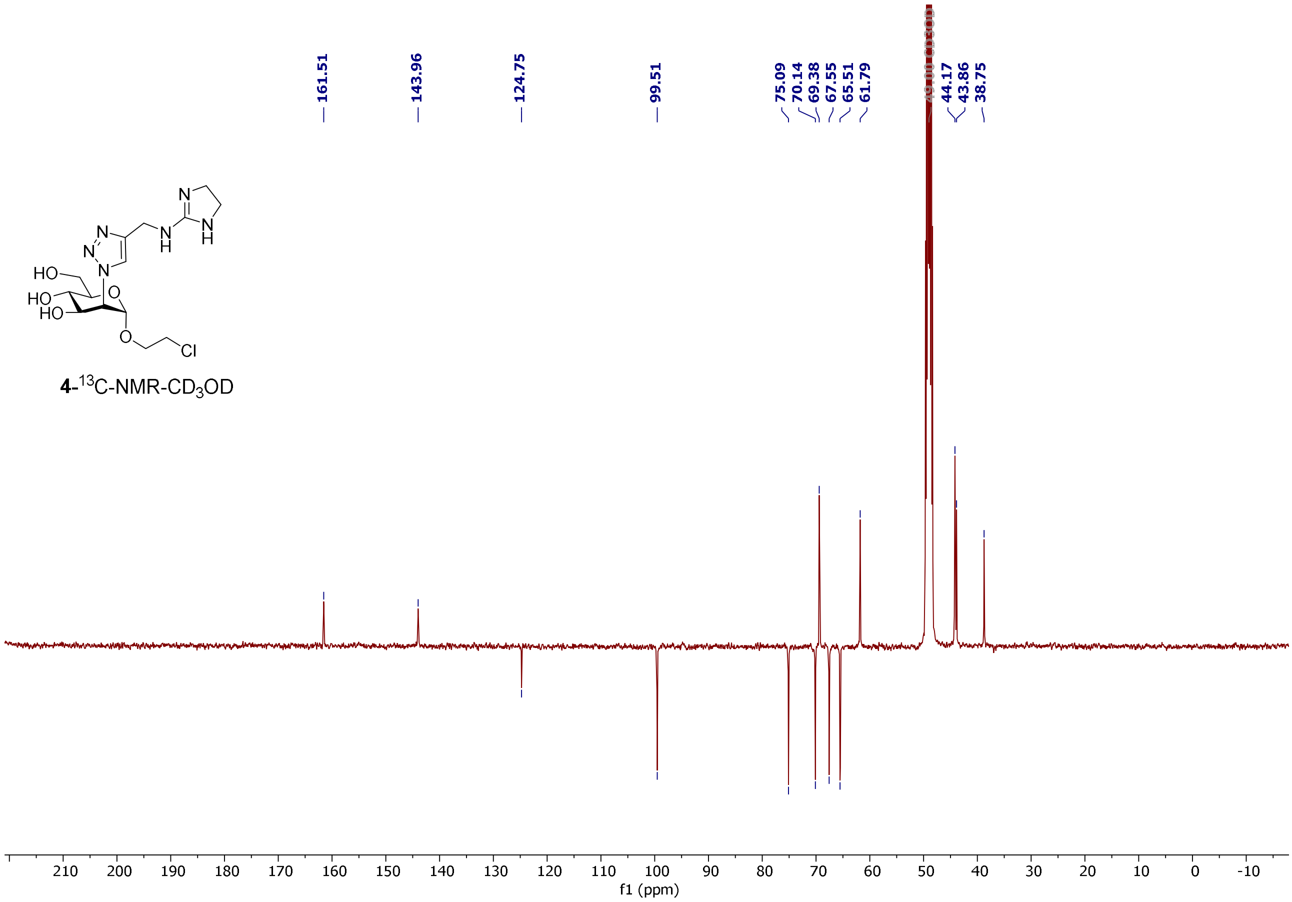
161.51
143.96
124.75
99.51
75.09
70.14
69.38
67.55
65.51
61.79
44.17
43.86
38.75
4-13C-NMR-CD3OD
f1 (ppm)

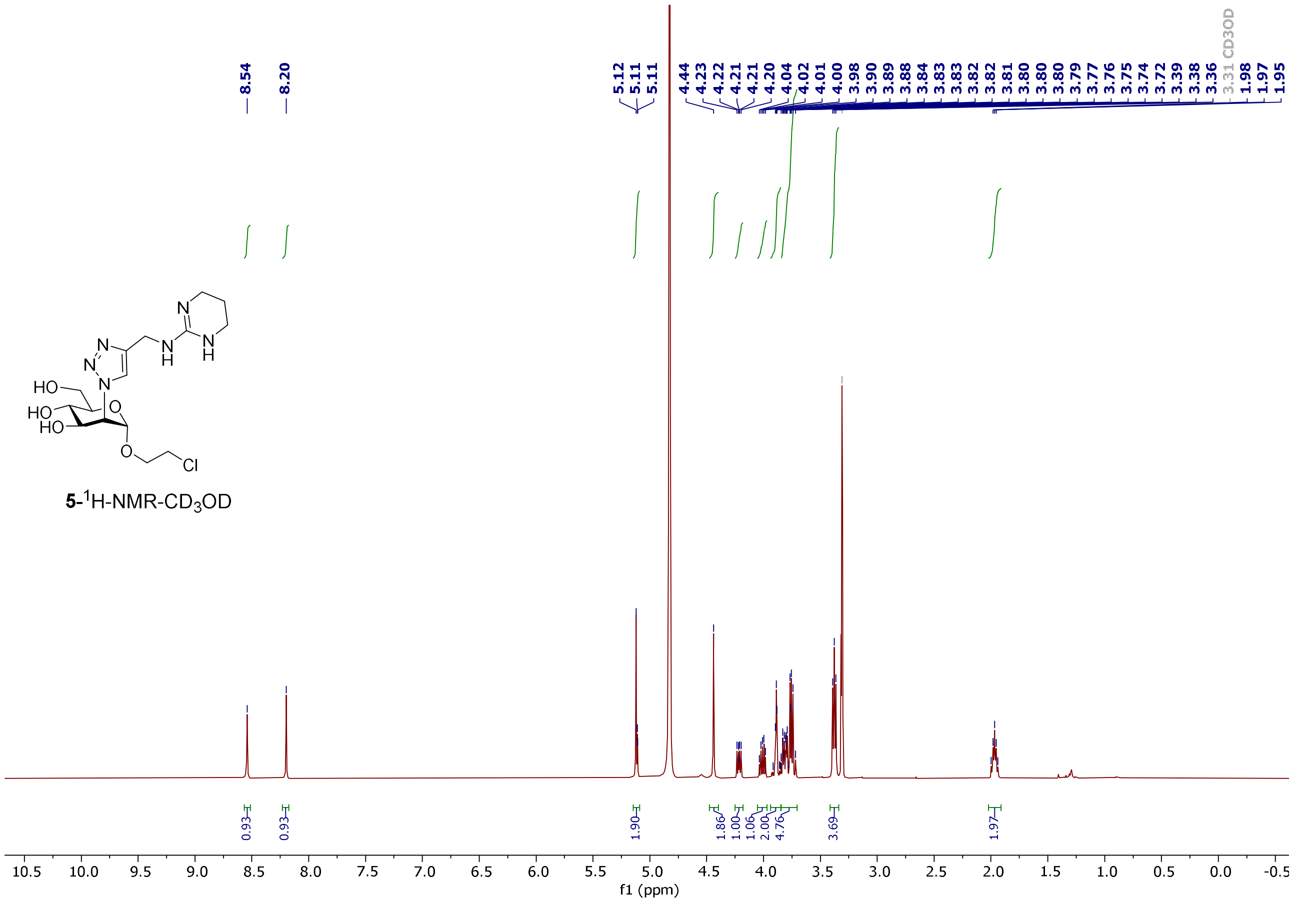
8.54
8.20
5.12
5.11
5.11
4.44
4.23
4.22
4.21
4.21
4.20
4.04
4.02
4.01
4.00
3.98
3.90
3.89
3.88
3.84
3.83
3.83
3.82
3.82
3.81
3.80
3.80
3.79
3.77
3.76
3.75
3.74
3.72
3.39
3.38
3.36
3.31 CD3OD
1.98
1.97
1.95
0.93
0.93
1.90
1.86
1.00
1.06
2.00
4.76
3.69
1.97
5-^{1}H-NMR-CD_3OD
f1 (ppm)

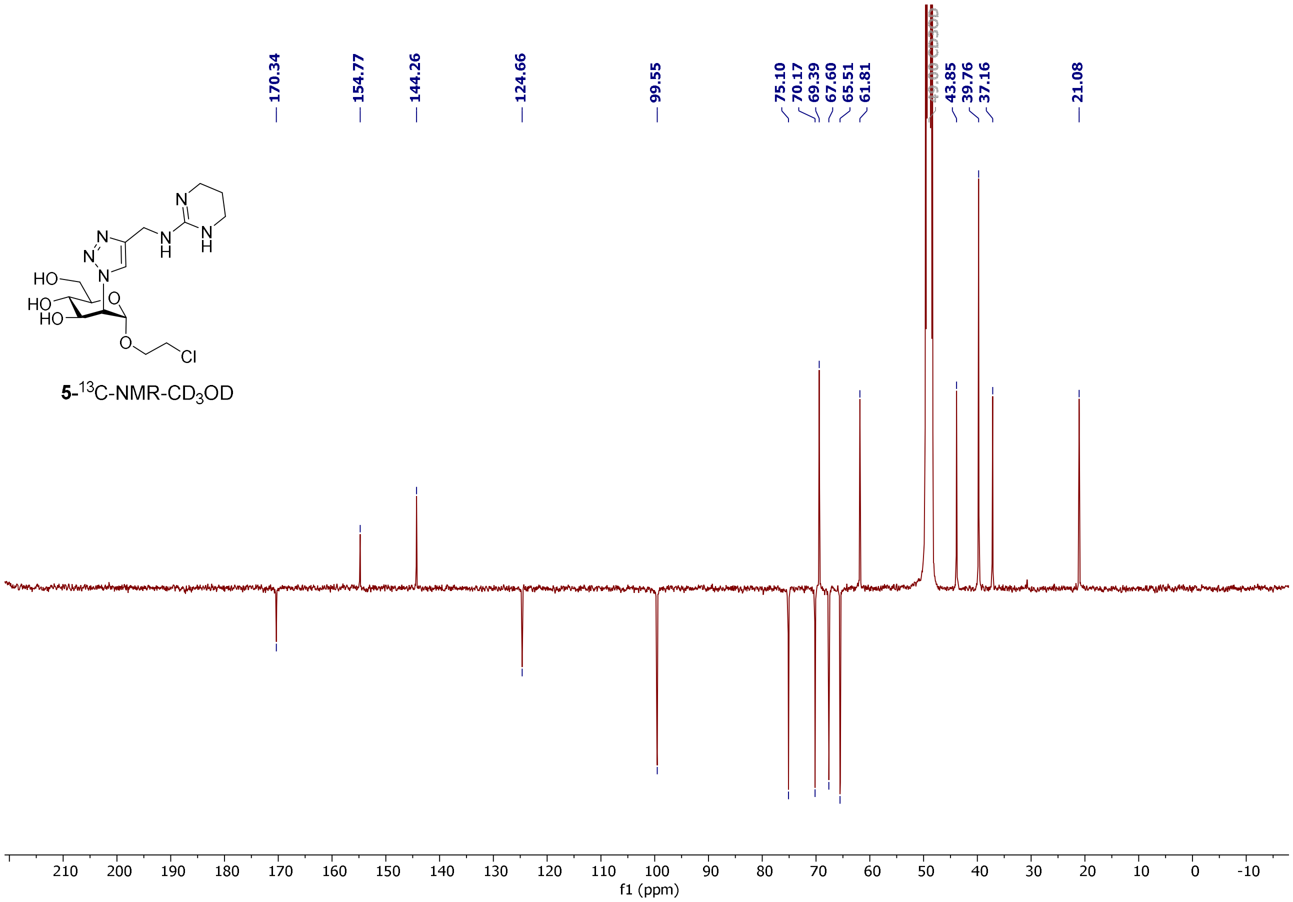
170.34
154.77
144.26
124.66
99.55
75.10
70.17
69.39
67.60
65.51
61.81
CD3OD
43.85
39.76
37.16
21.08
5-^{13}C-NMR-CD_3OD
f1 (ppm)

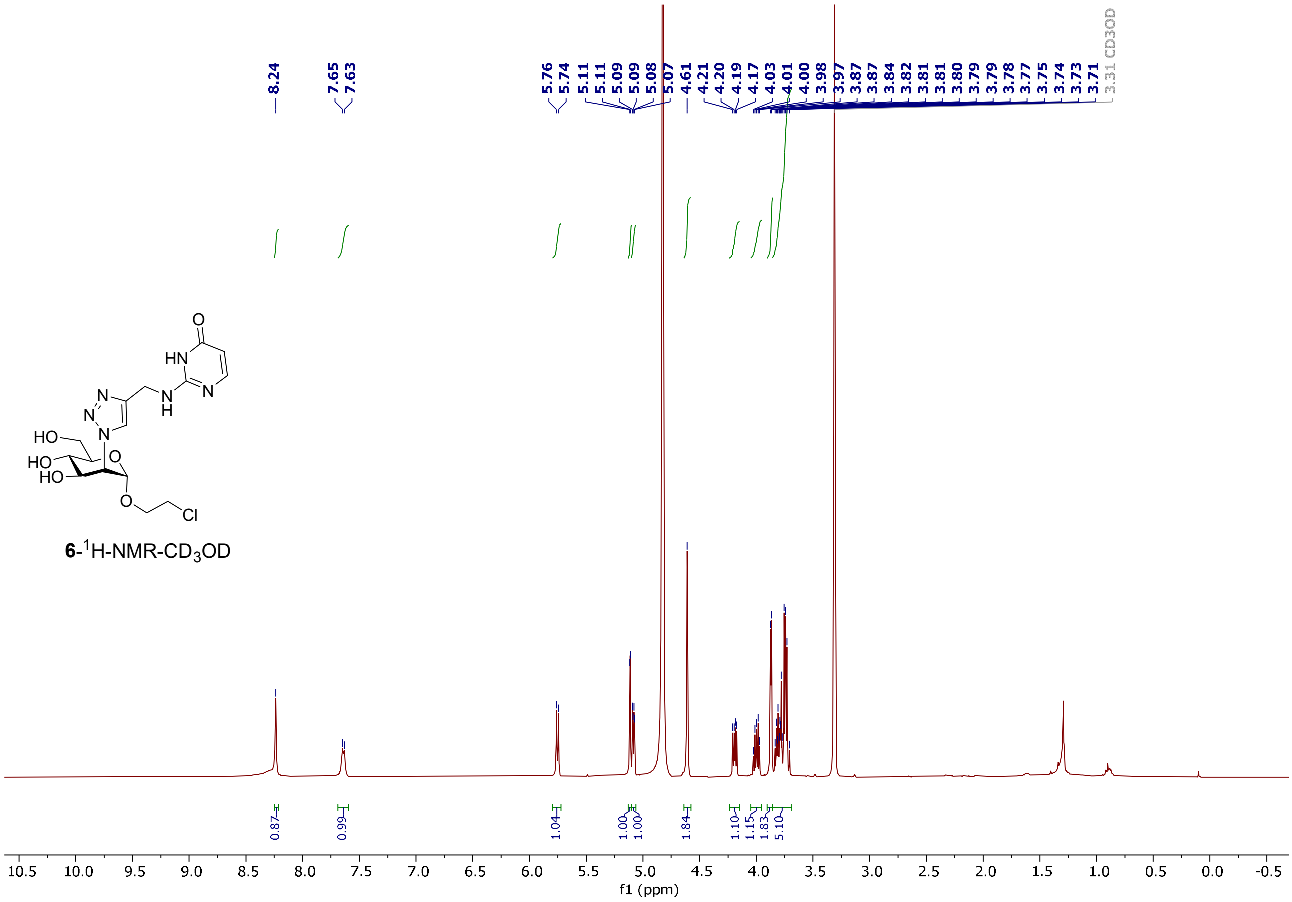

6-¹H-NMR-CD$_3$OD

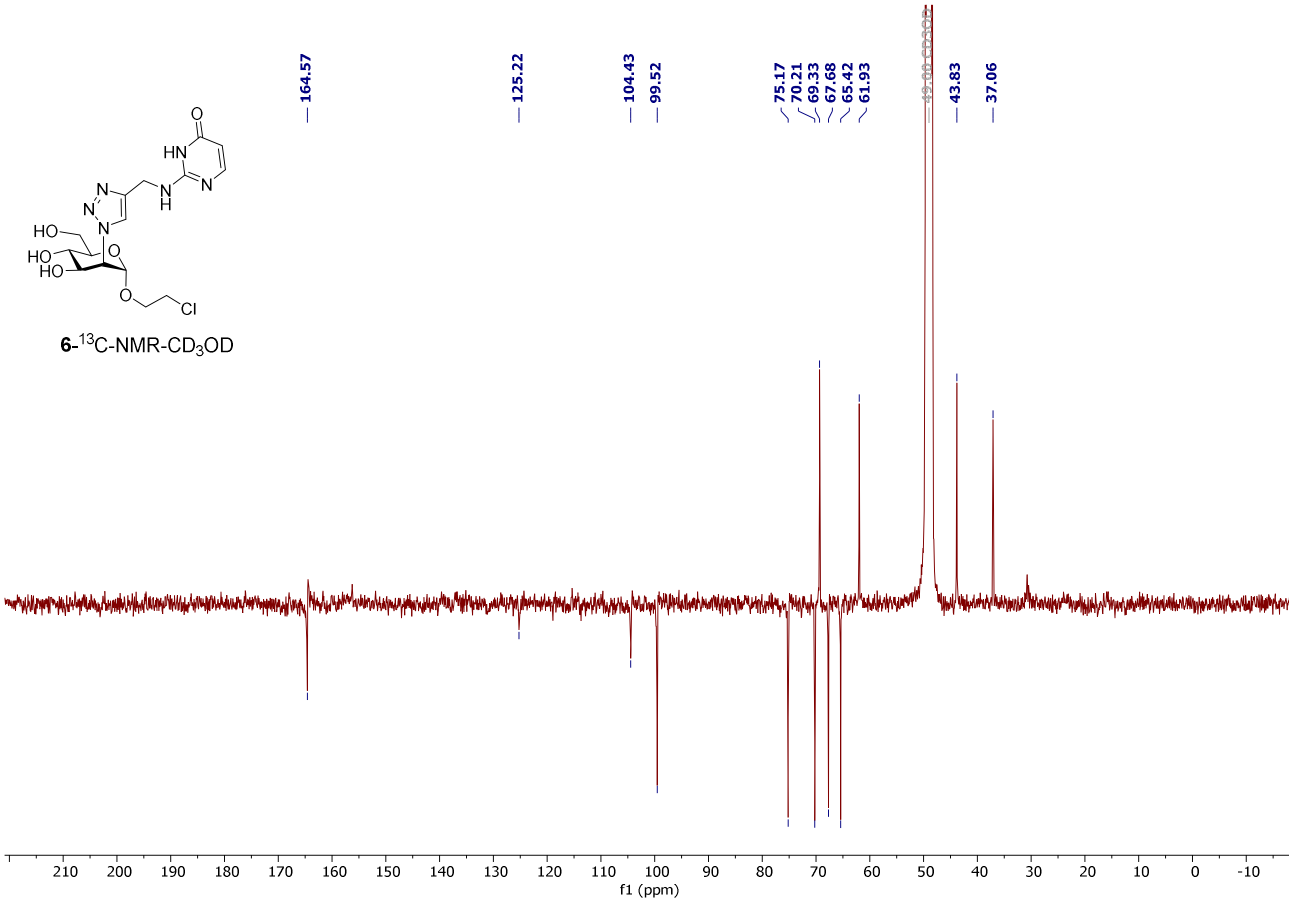

6-¹³C-NMR-CD$_3$OD

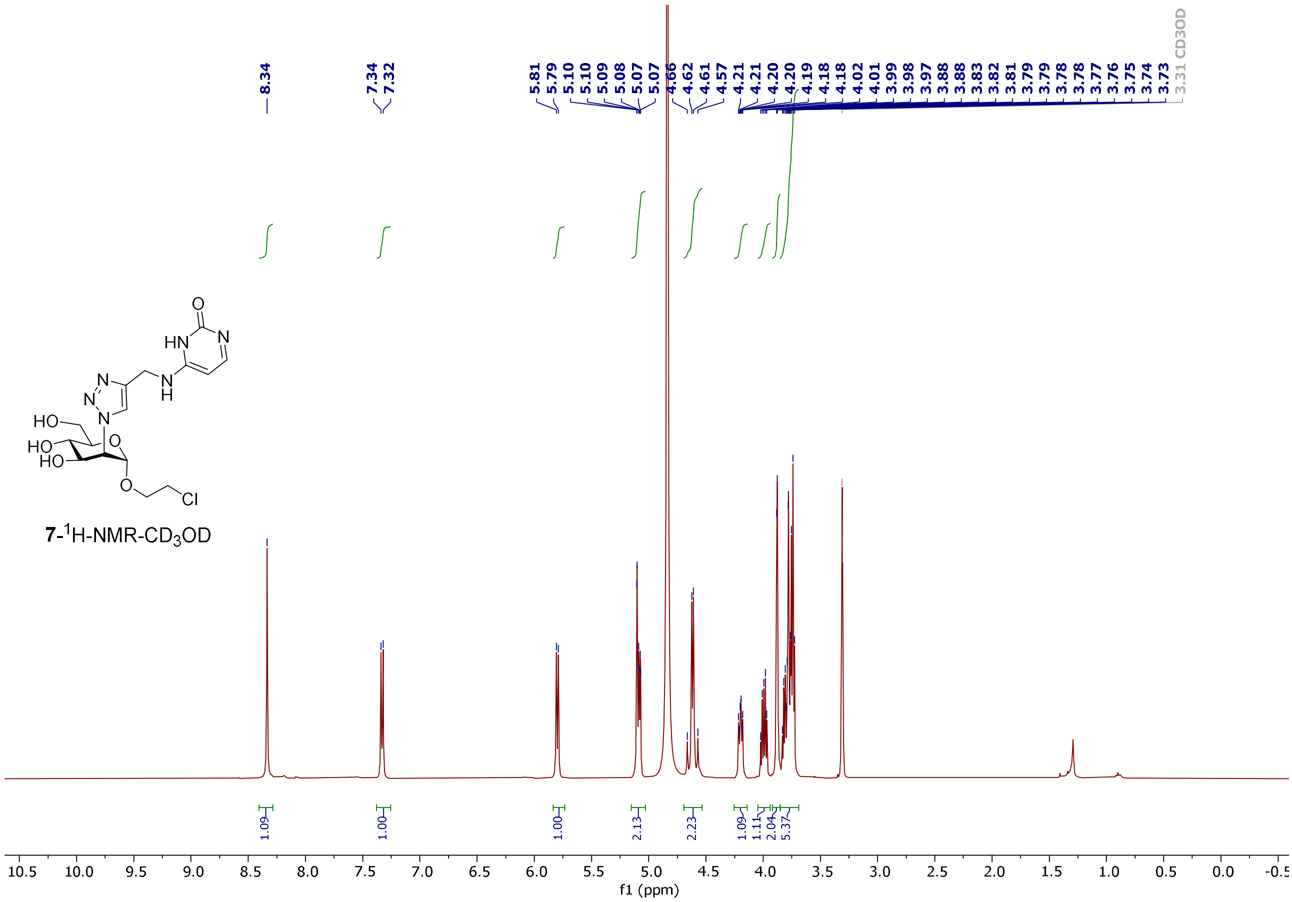

8.34
7.34
7.32
5.81
5.79
5.10
5.10
5.09
5.08
5.07
5.07
4.66
4.62
4.61
4.57
4.21
4.21
4.20
4.20
4.19
4.18
4.18
4.02
4.01
3.99
3.98
3.97
3.88
3.83
3.82
3.81
3.79
3.79
3.78
3.78
3.77
3.76
3.75
3.74
3.73
3.31 CD3OD
O
HN
N
N
N
N
N
H
HO
HO
HO
O
O
Cl
7-^{1}H-NMR-CD$_3$OD
1.09
1.00
1.00
2.13
2.23
1.09
1.11
2.04
5.37
10.5
10.0
9.5
9.0
8.5
8.0
7.5
7.0
6.5
6.0
5.5
5.0
4.5
4.0
3.5
3.0
2.5
2.0
1.5
1.0
0.5
0.0
-0.5
f1 (ppm)

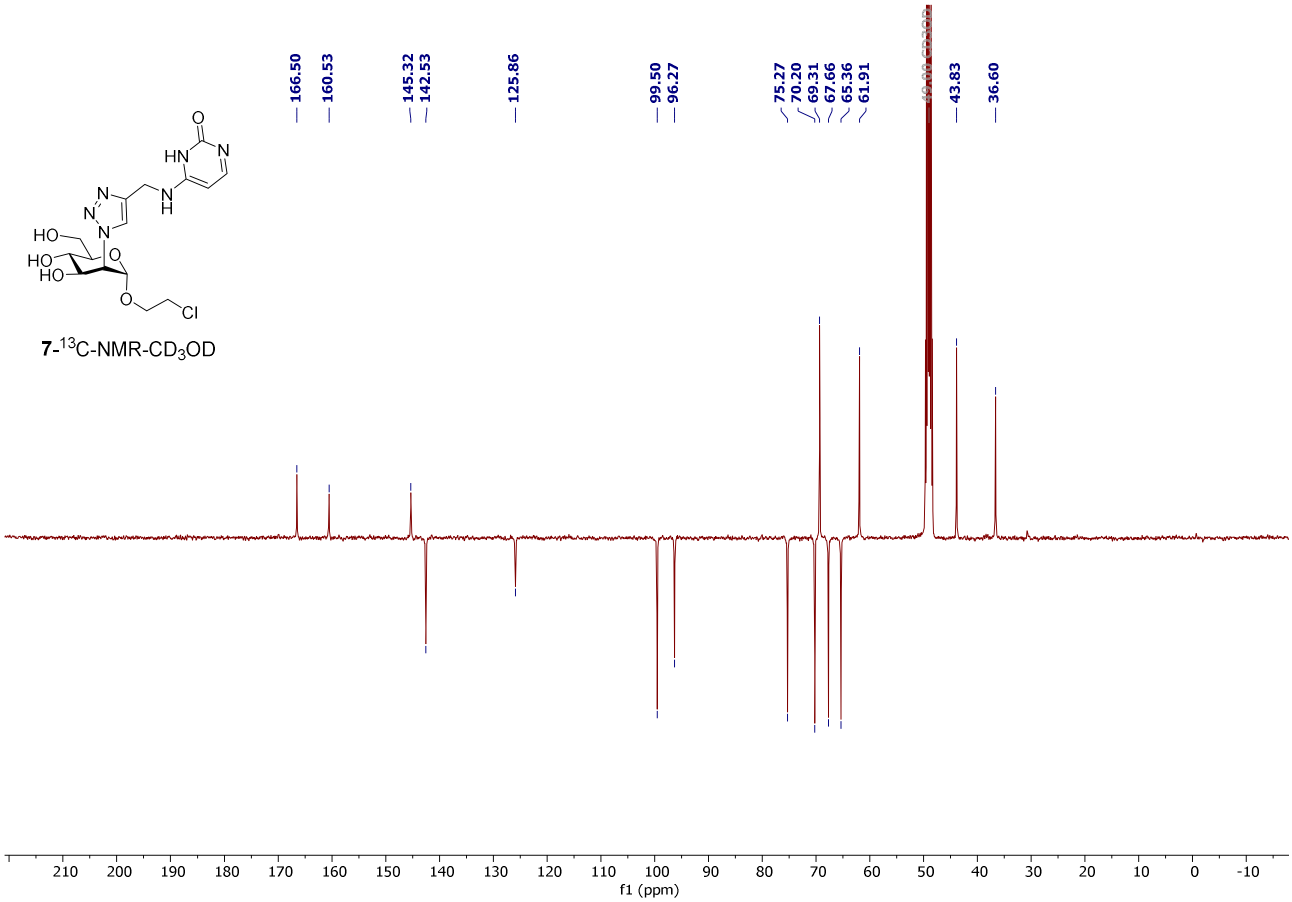

166.50
160.53
145.32
142.53
125.86
99.50
96.27
75.27
70.20
69.31
67.66
65.36
61.91
43.83
36.60
O
HN
N
N
N
N
N
H
HO
HO
HO
O
O
Cl
7-^{13}C-NMR-CD$_3$OD
210
200
190
180
170
160
150
140
130
120
110
100
90
80
70
60
50
40
30
20
10
0
-10
f1 (ppm)

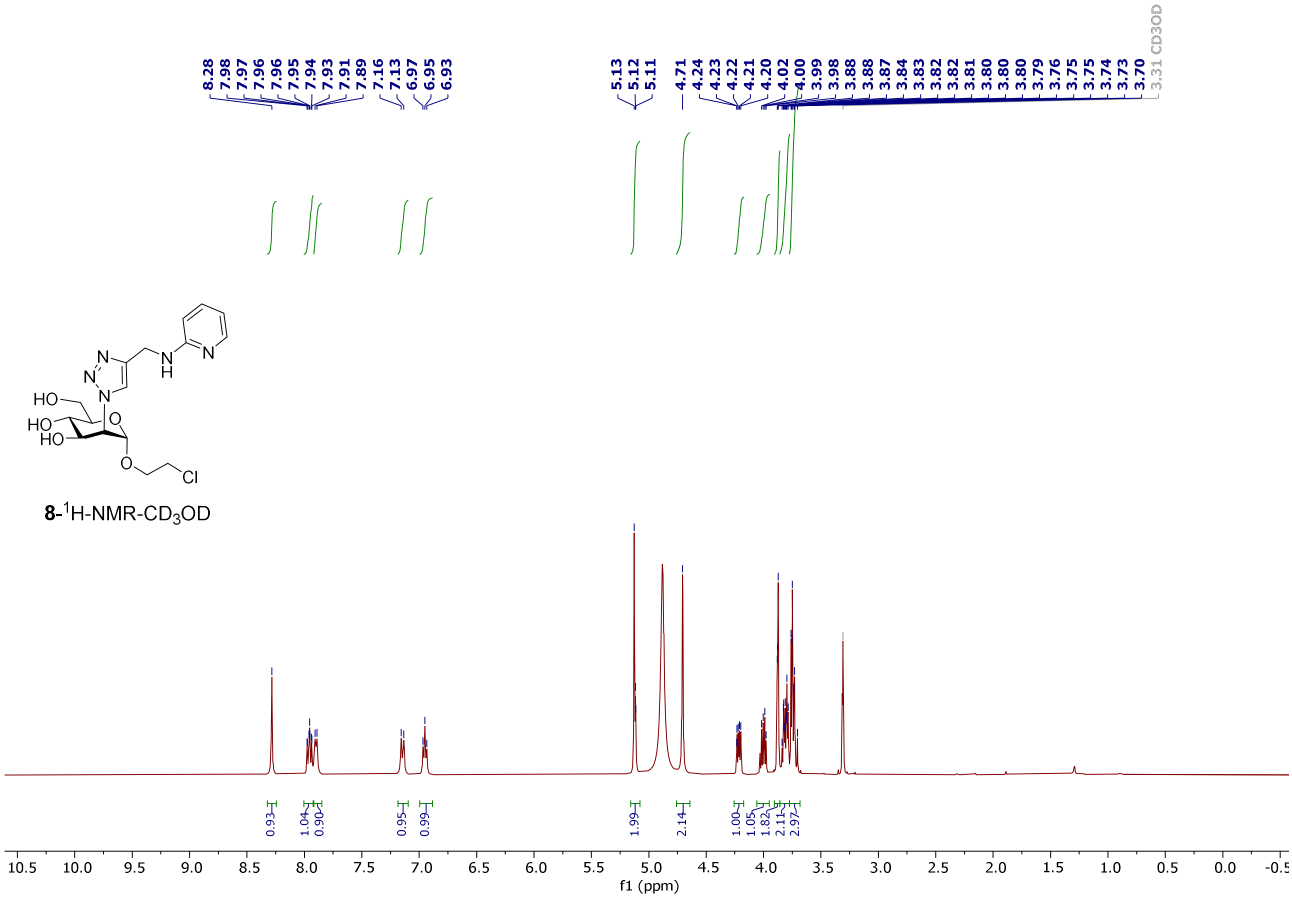

8-^{1}H-NMR-CD_3OD

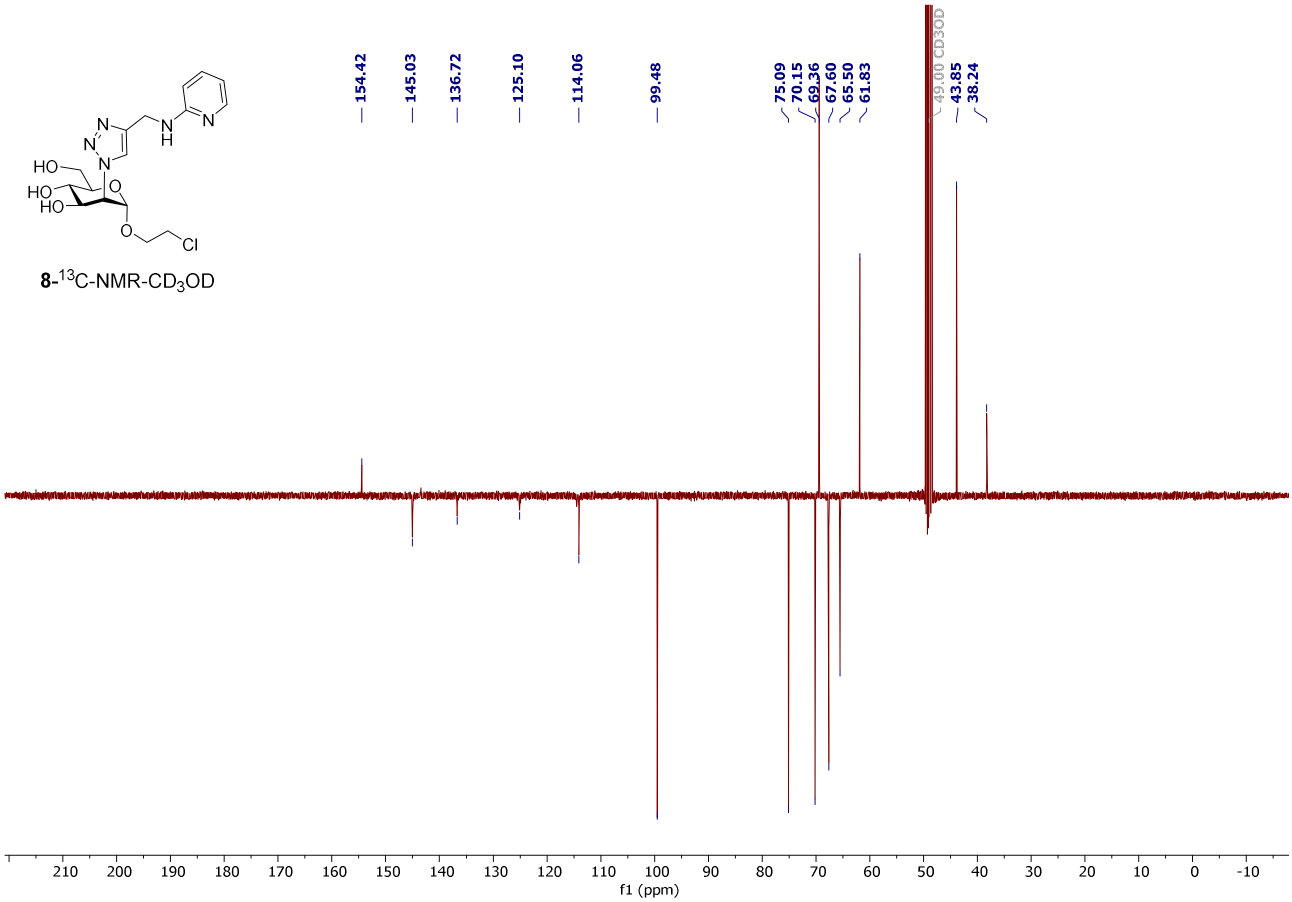

8-^{13}C-NMR-CD_3OD

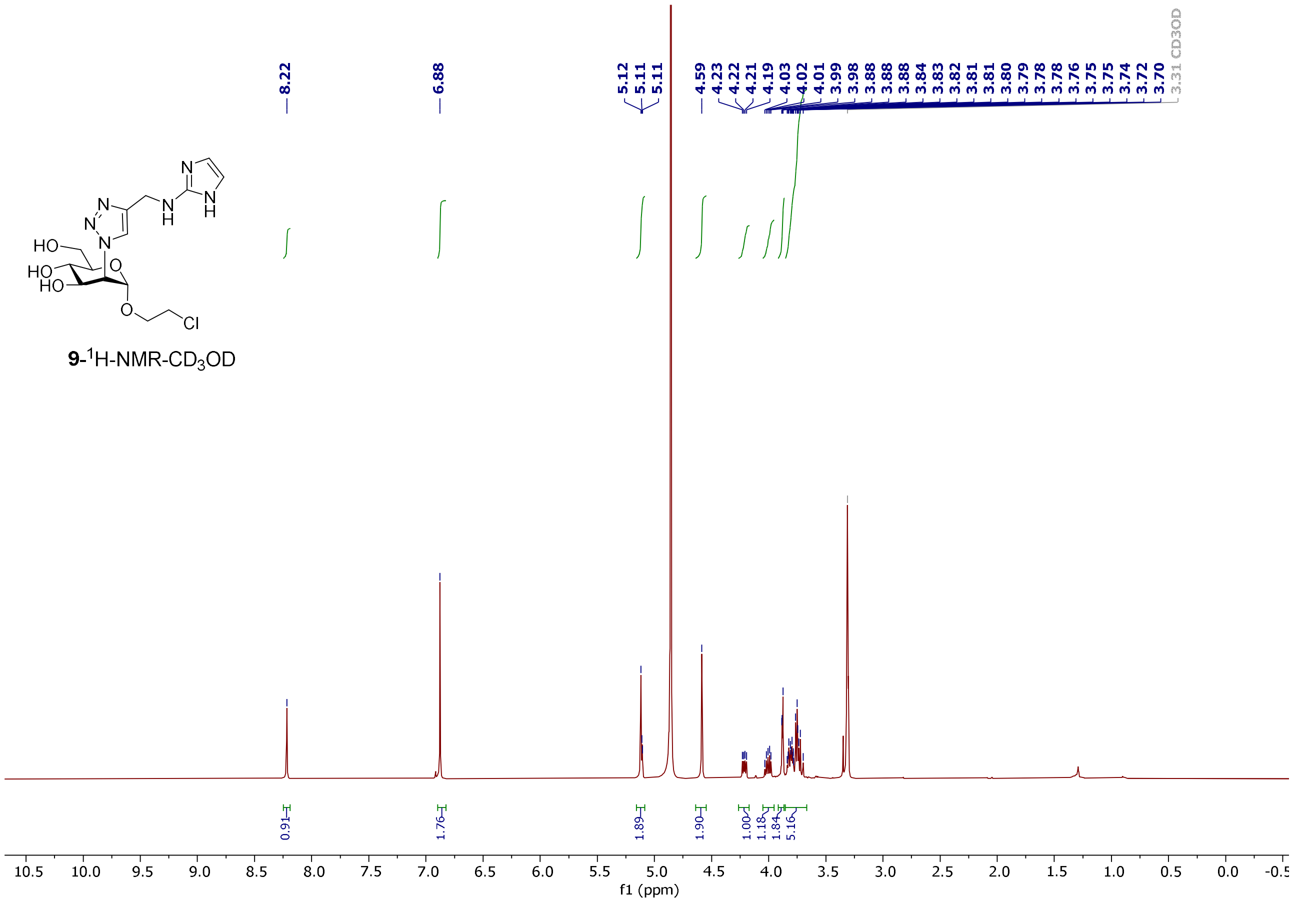

9-¹H-NMR-CD₃OD

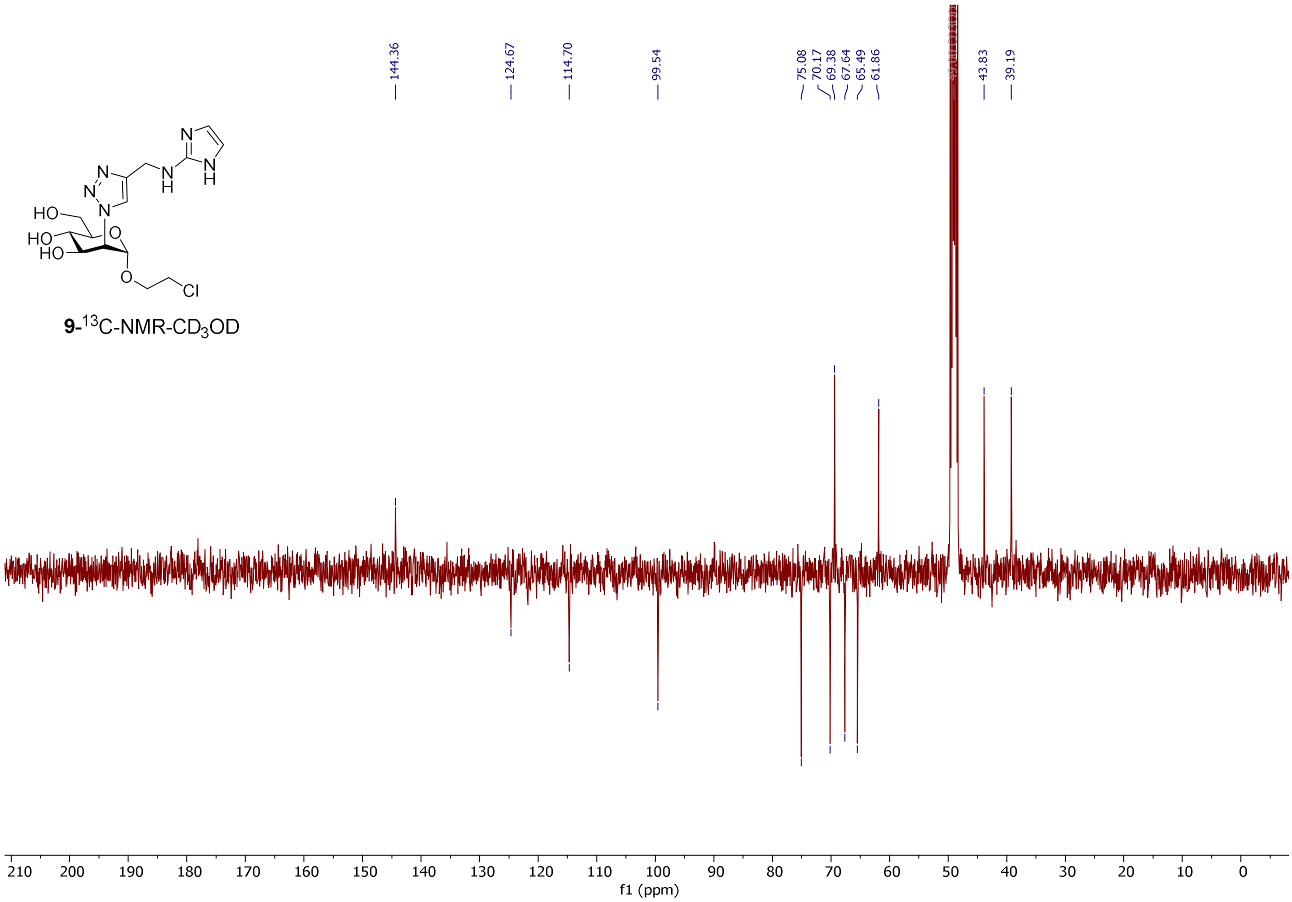

9-¹³C-NMR-CD₃OD

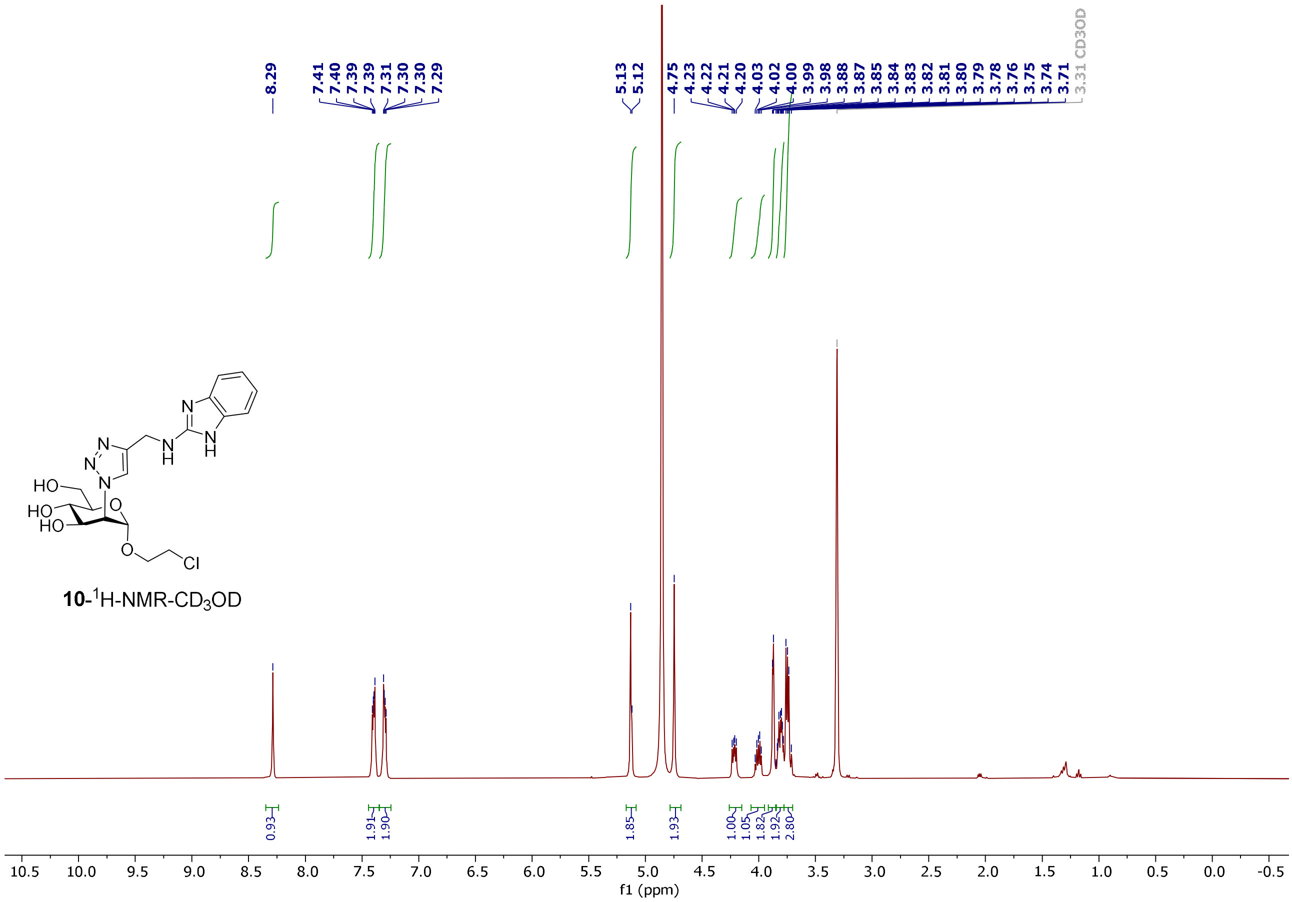

10-^{1}H-NMR-CD_3OD

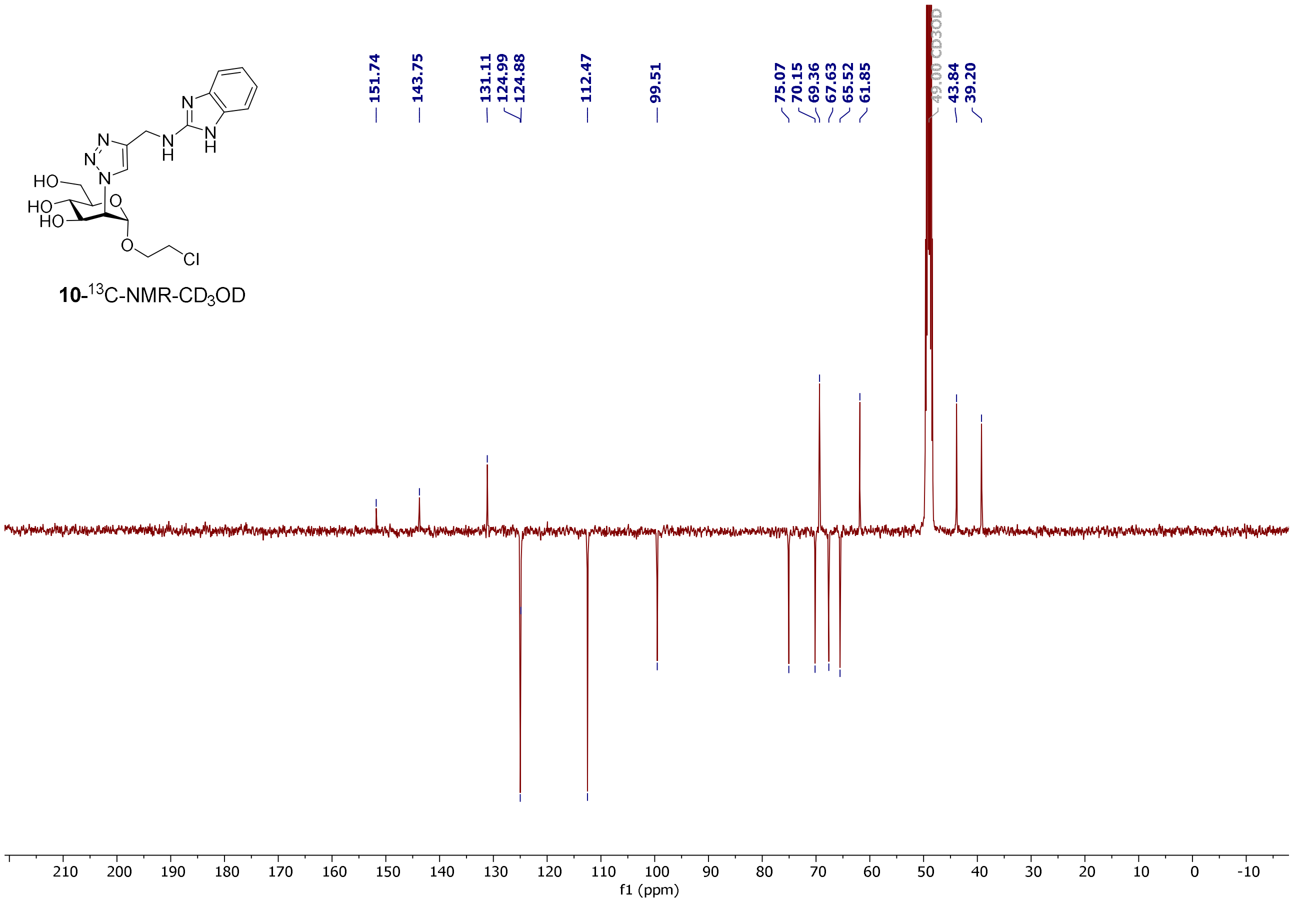

10-^{13}C-NMR-CD_3OD

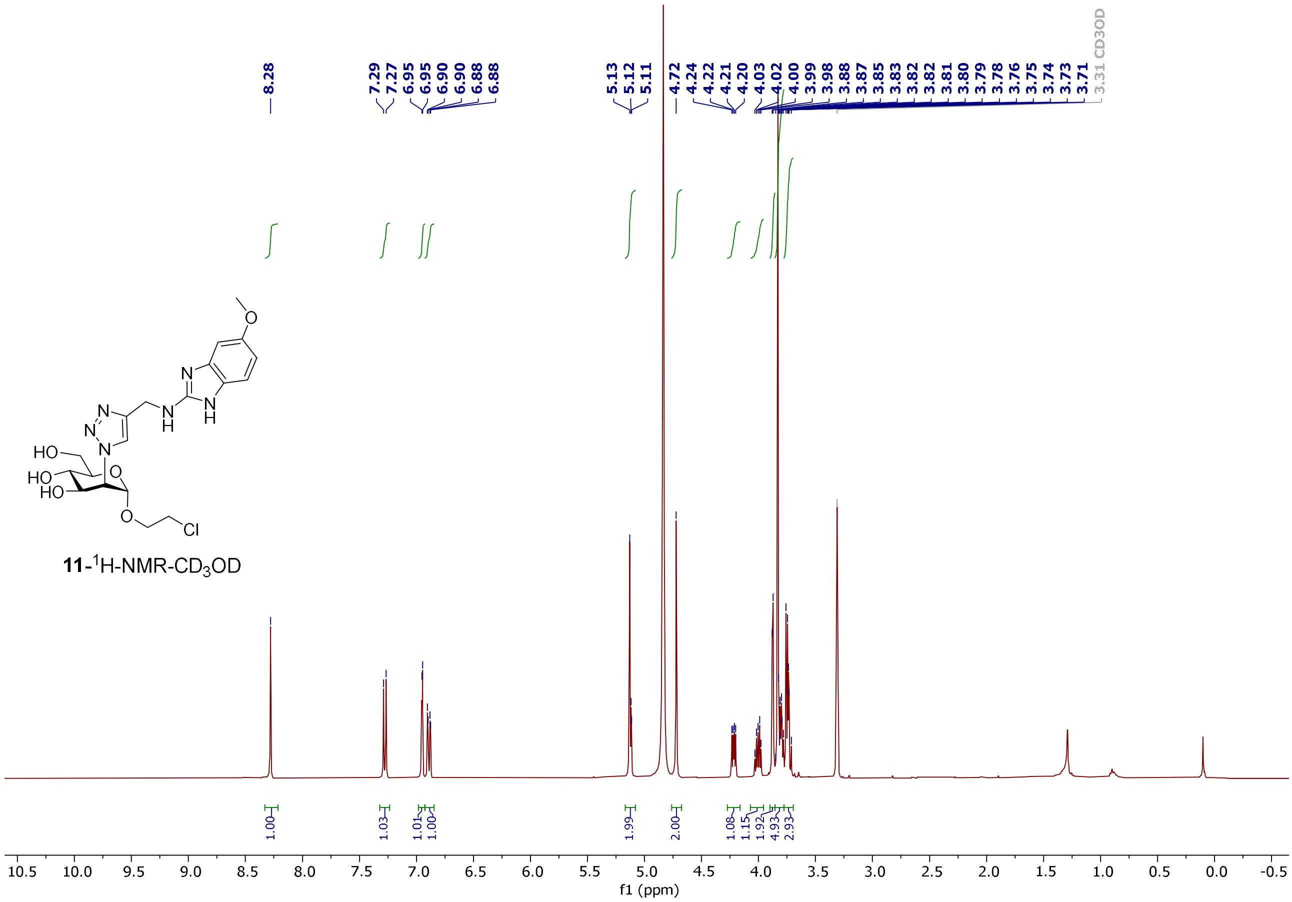

11-^{1}H-NMR-CD$_3$OD

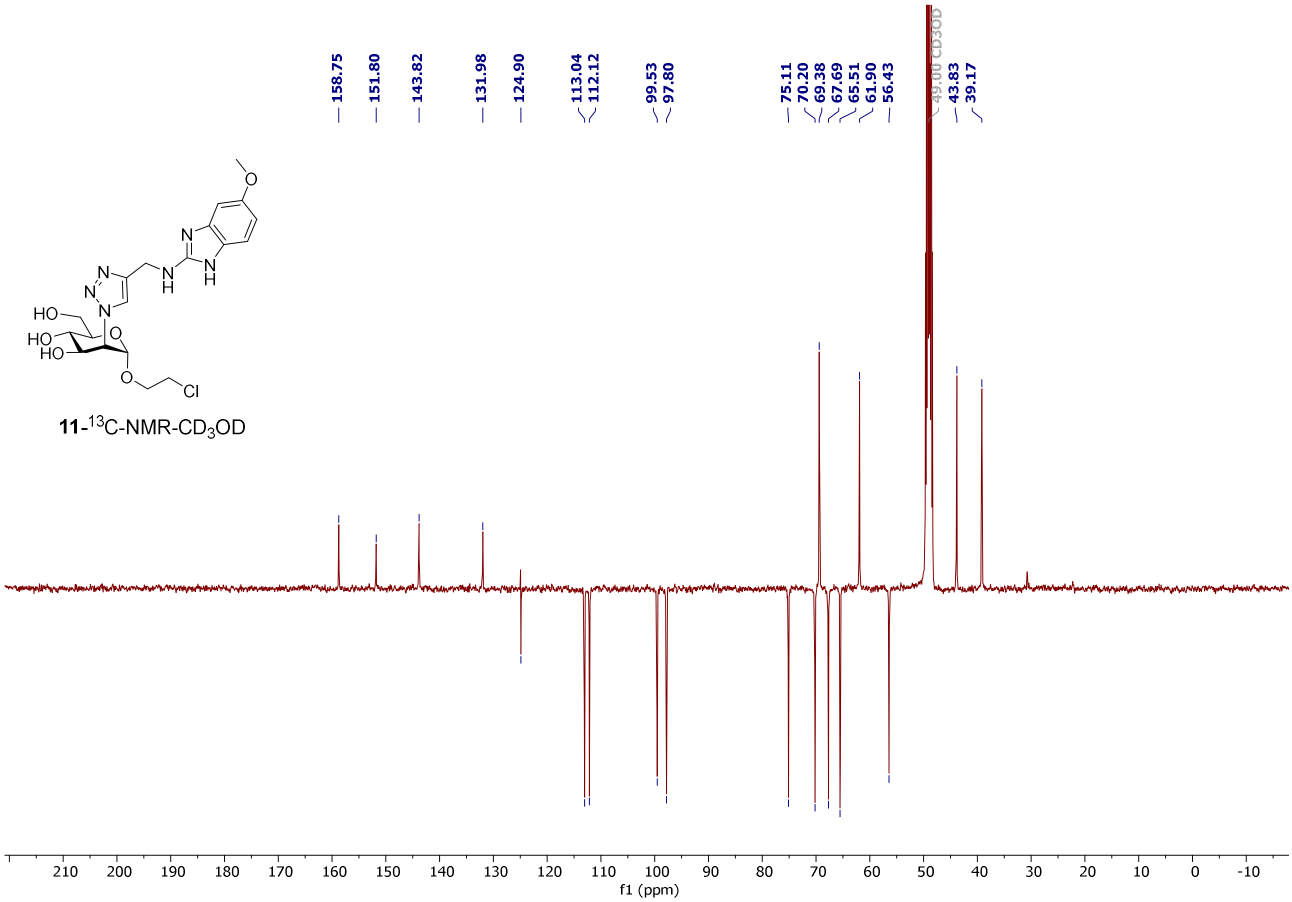

11-^{13}C-NMR-CD$_3$OD

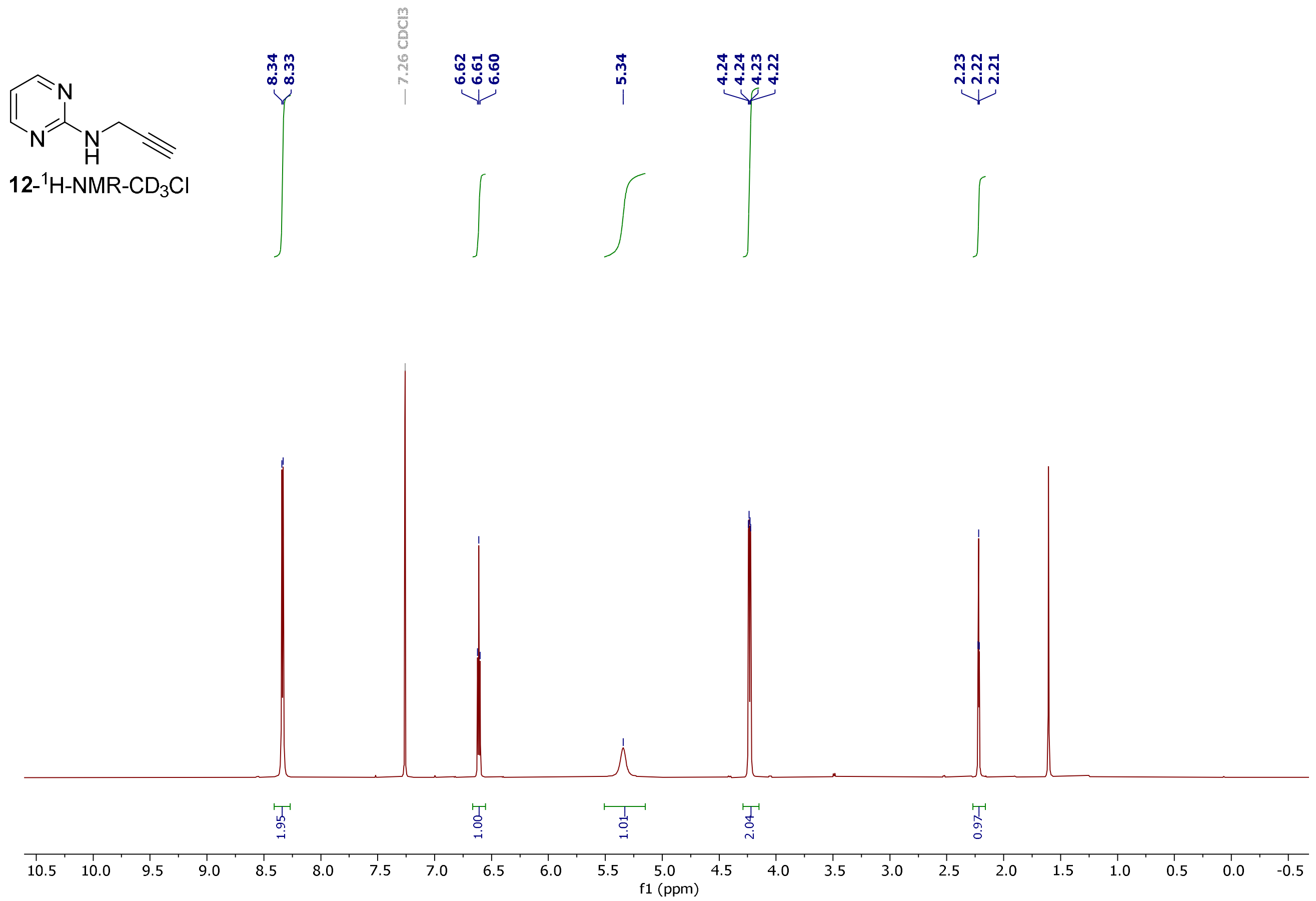
N
N
N
H
12-¹H-NMR-CD₃Cl
8.34
8.33
7.26 CDCl3
6.62
6.61
6.60
5.34
4.24
4.24
4.23
4.22
2.23
2.22
2.21
1.95
1.00
1.01
2.04
0.97
10.5
10.0
9.5
9.0
8.5
8.0
7.5
7.0
6.5
6.0
5.5
5.0
4.5
4.0
3.5
3.0
2.5
2.0
1.5
1.0
0.5
0.0
-0.5
f1 (ppm)

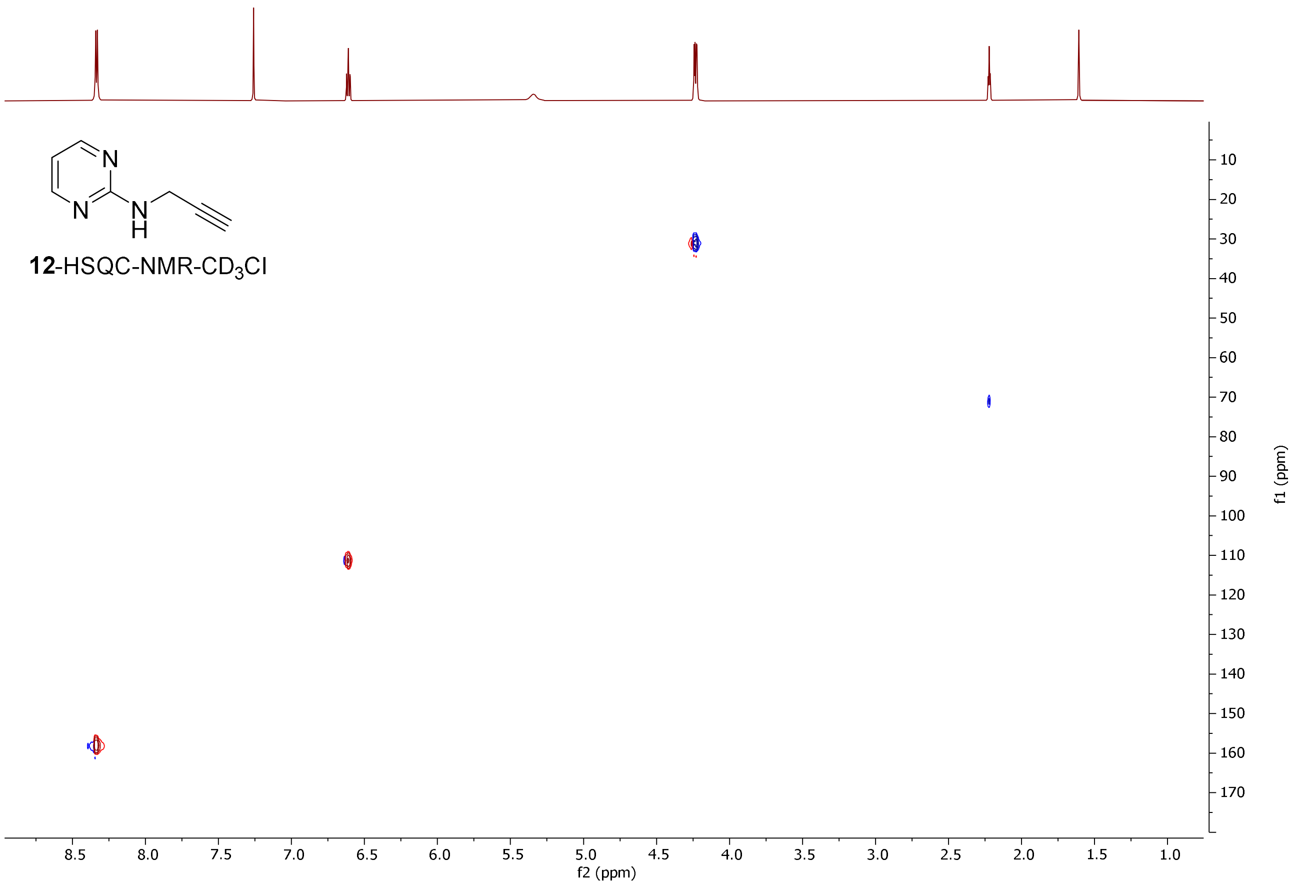
N
N
N
H
12-HSQC-NMR-CD₃Cl
10
20
30
40
50
60
70
80
90
100
110
120
130
140
150
160
170
f1 (ppm)
8.5
8.0
7.5
7.0
6.5
6.0
5.5
5.0
4.5
4.0
3.5
3.0
2.5
2.0
1.5
1.0
f2 (ppm)

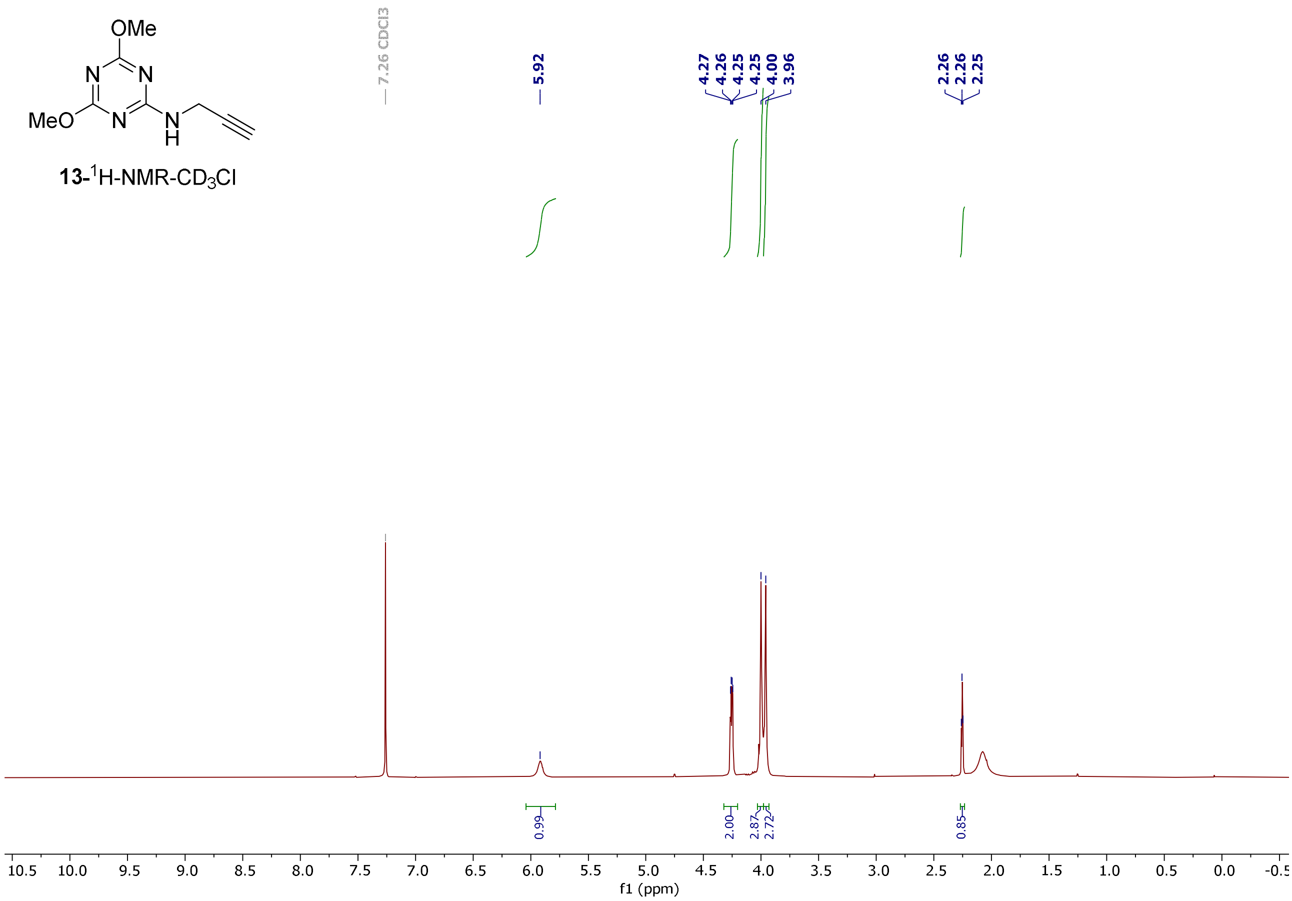

13-^{1}H-NMR-CD_3Cl

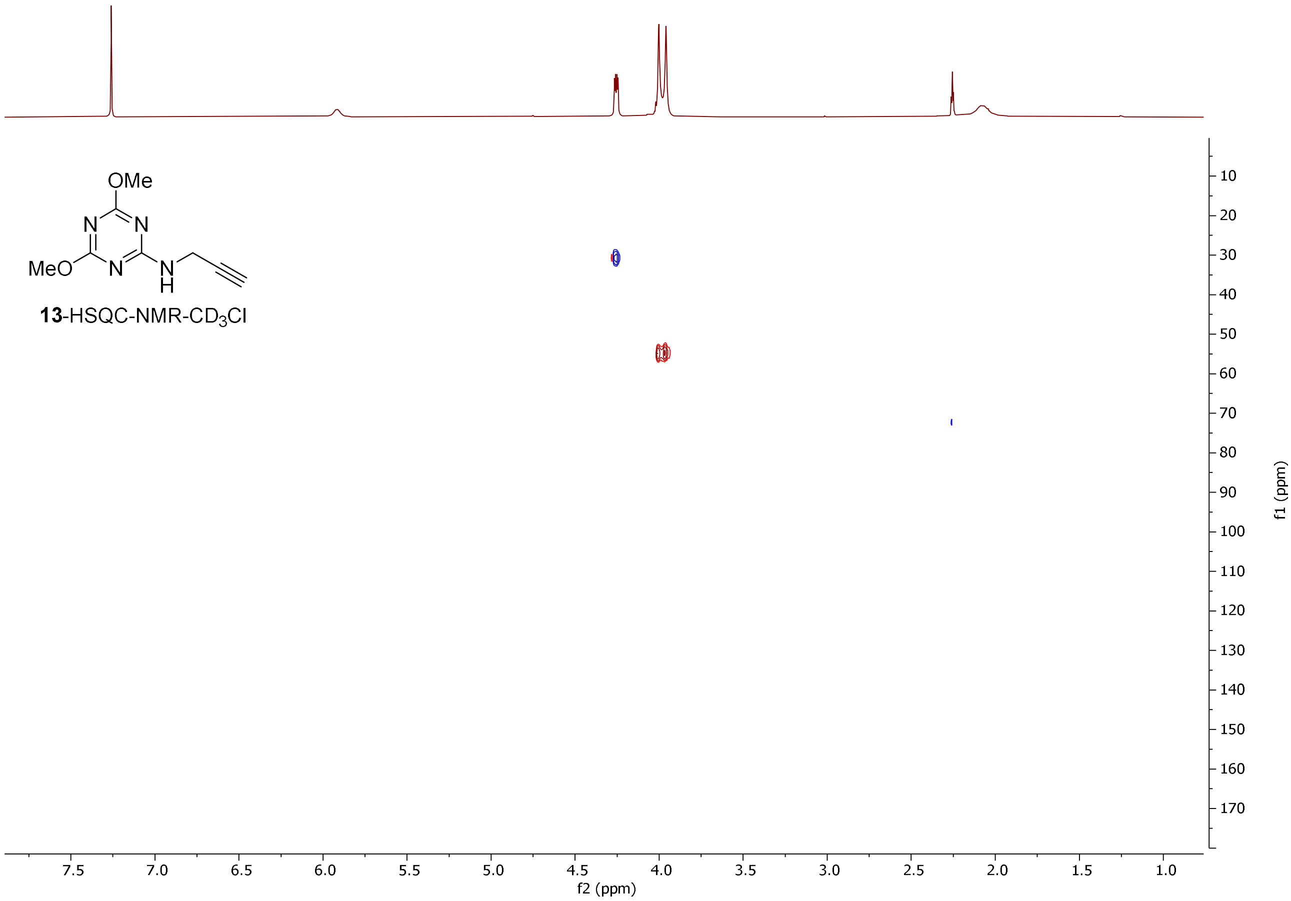

13-HSQC-NMR-CD_3Cl

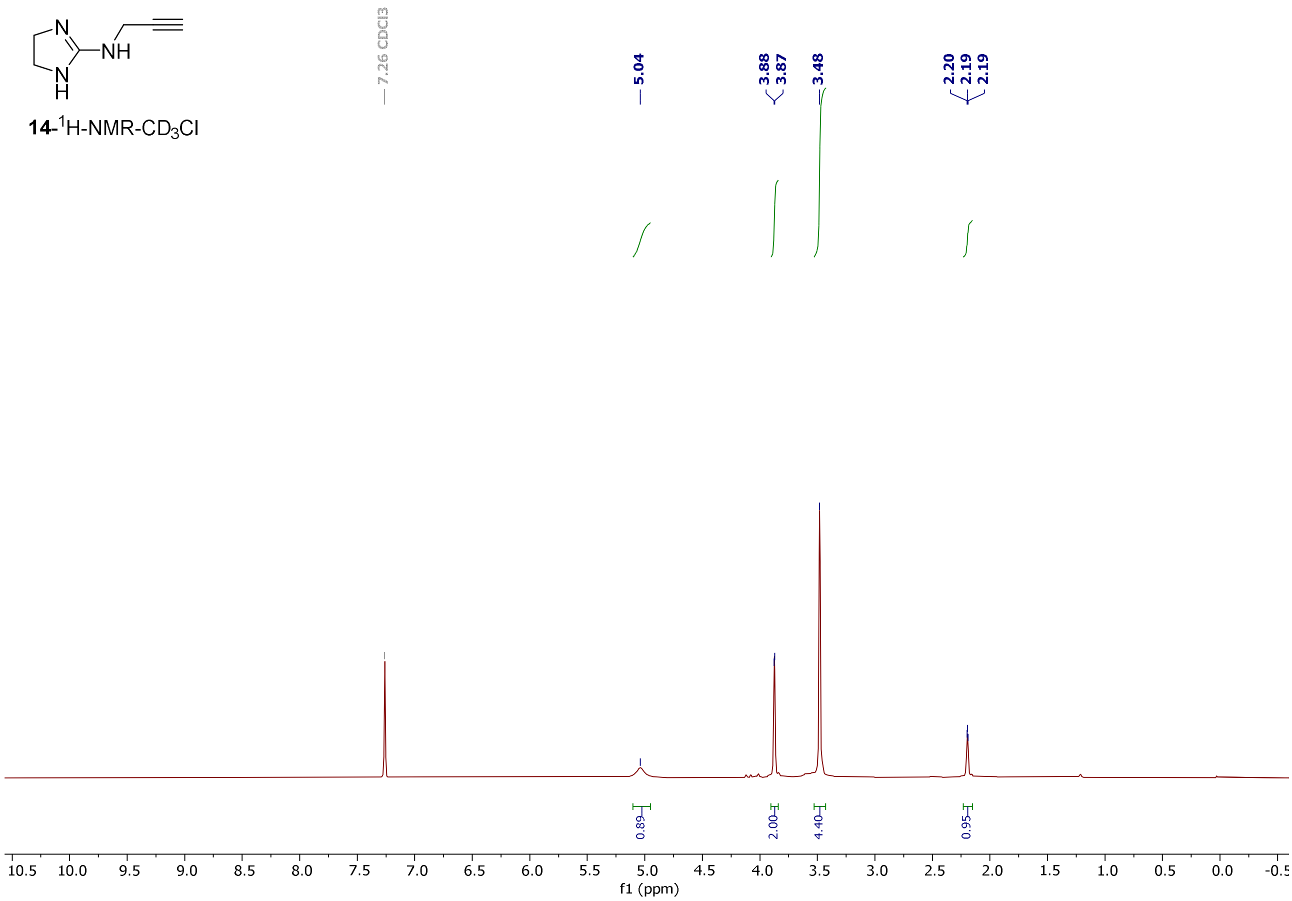

NH
N
H
N
14-1H-NMR-CD3Cl
7.26 CDCl3
5.04
3.88
3.87
3.48
2.20
2.19
2.19
0.89
2.00
4.40
0.95
10.5 10.0 9.5 9.0 8.5 8.0 7.5 7.0 6.5 6.0 5.5 5.0 4.5 4.0 3.5 3.0 2.5 2.0 1.5 1.0 0.5 0.0 -0.5
f1 (ppm)

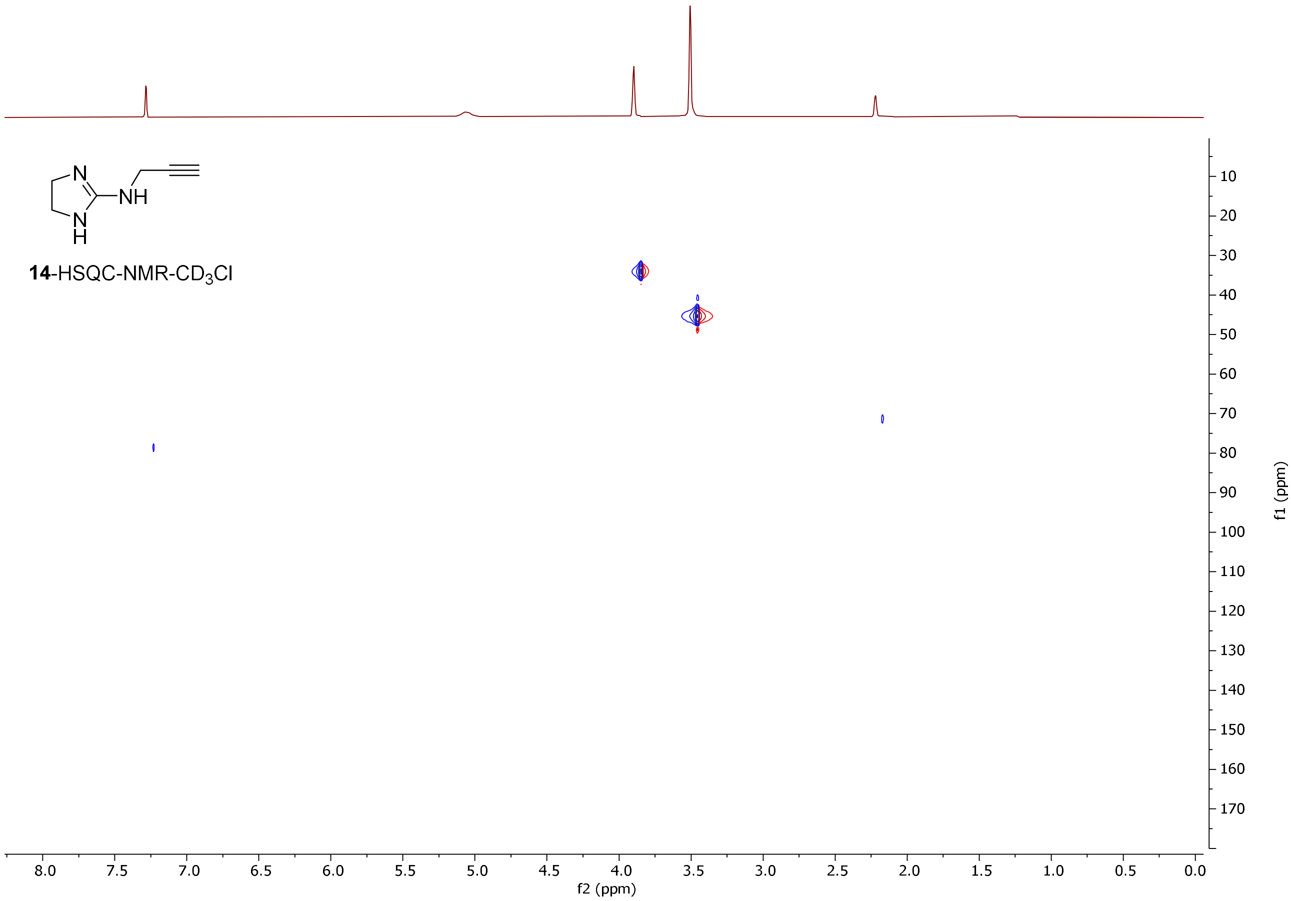

NH
N
H
N
14-HSQC-NMR-CD3Cl
8.0 7.5 7.0 6.5 6.0 5.5 5.0 4.5 4.0 3.5 3.0 2.5 2.0 1.5 1.0 0.5 0.0
f2 (ppm)
10 20 30 40 50 60 70 80 90 100 110 120 130 140 150 160 170
f1 (ppm)

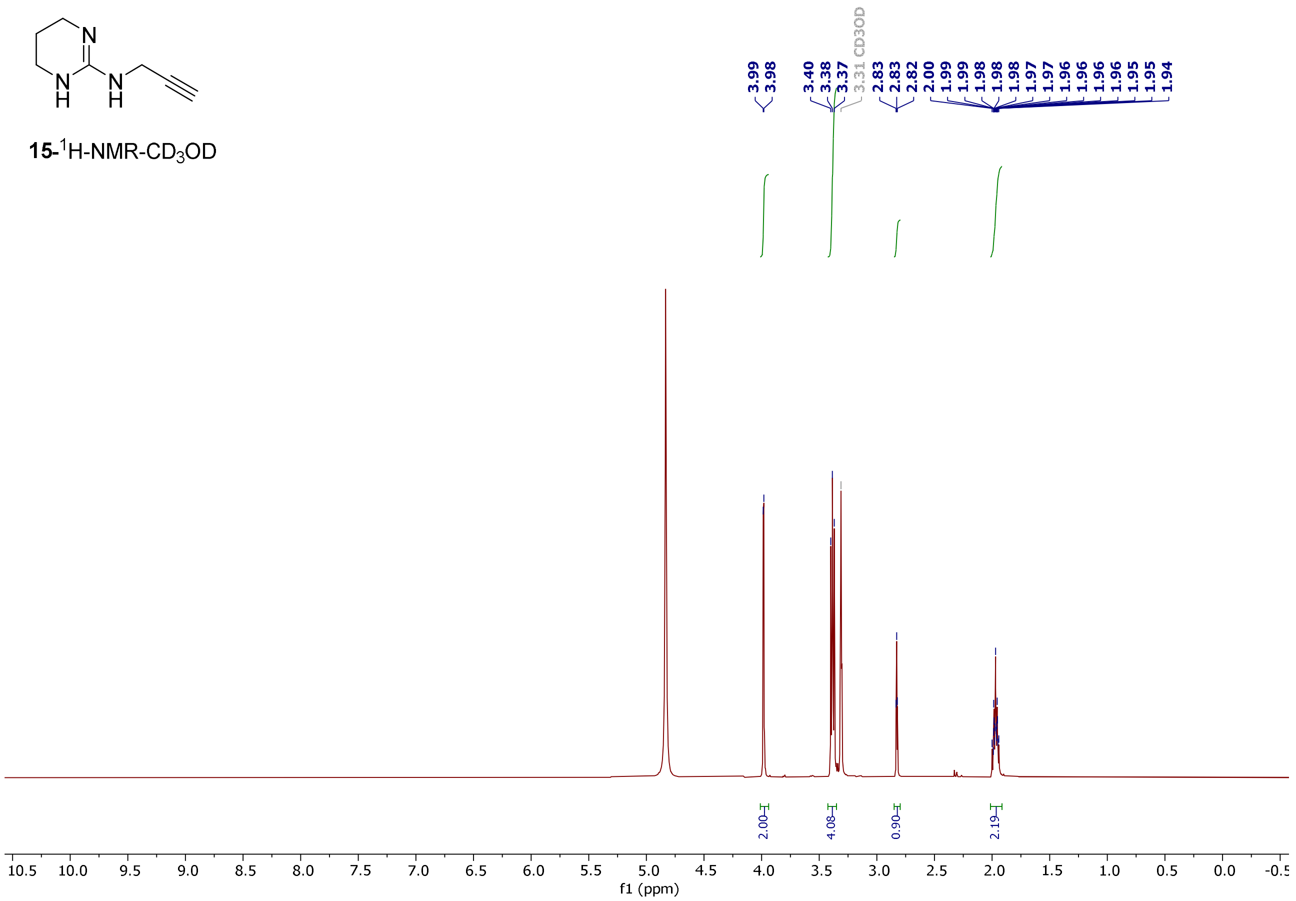

15-^{1}H-NMR-CD_3OD

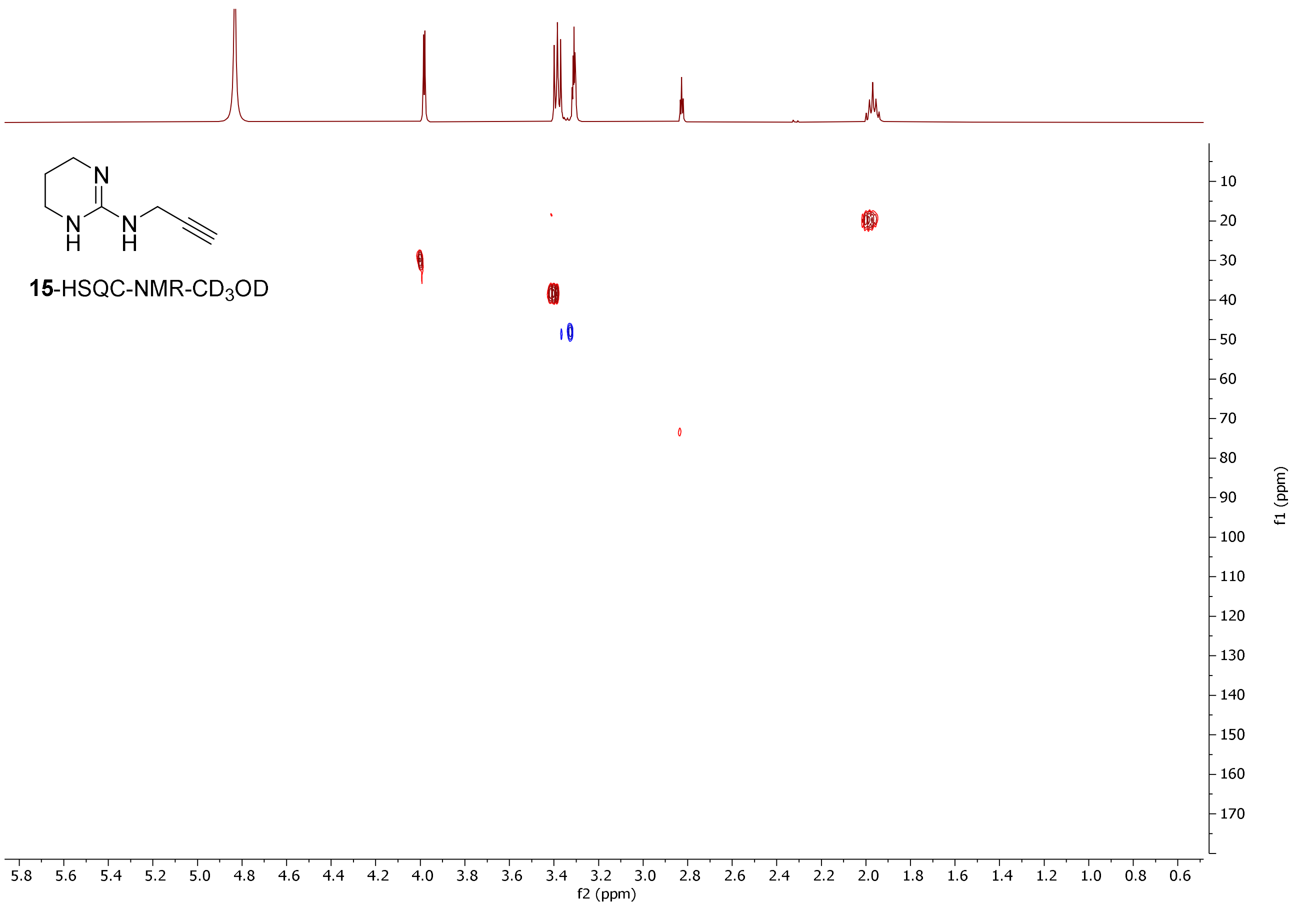

15-HSQC-NMR-CD_3OD

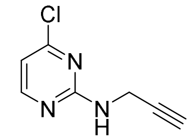

16-^{1}H-NMR-CD_3Cl

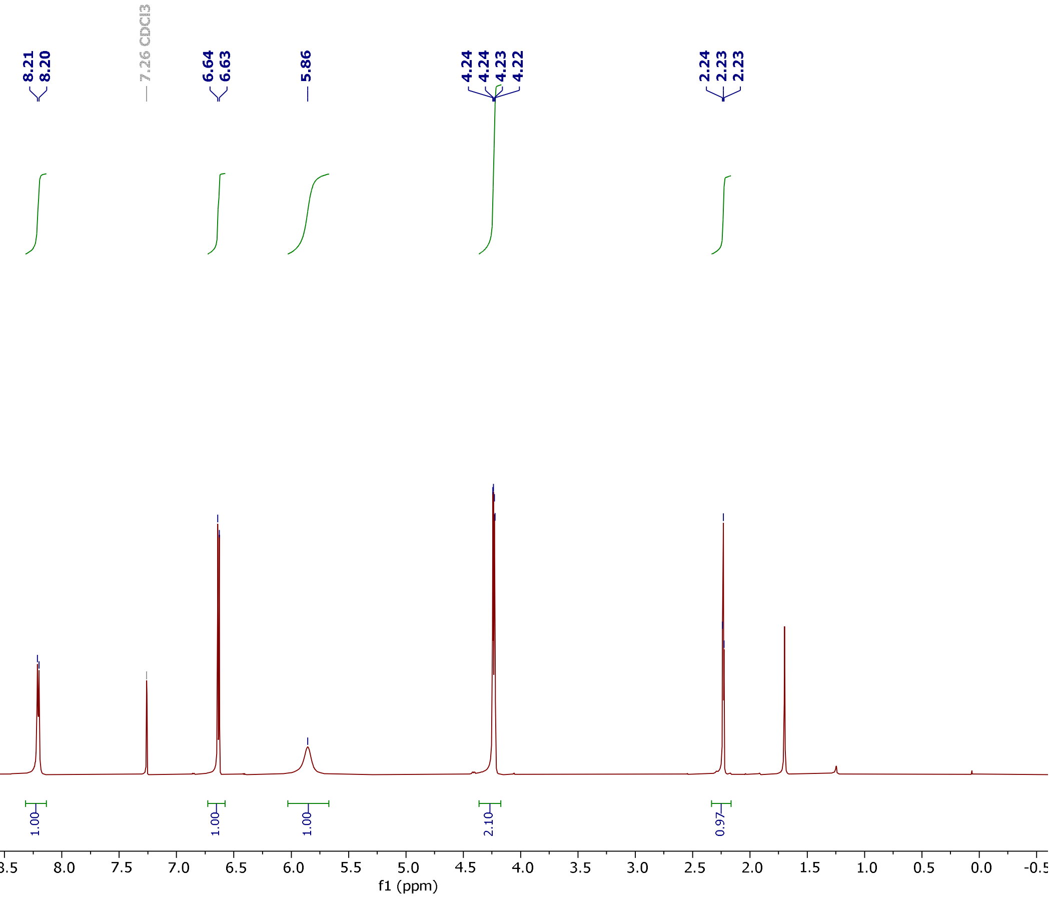

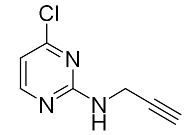

16-HSQC-NMR-CD_3Cl

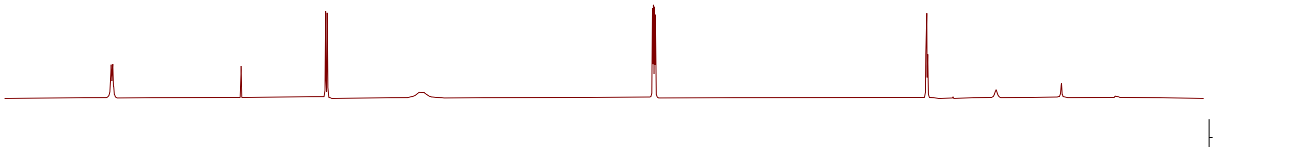

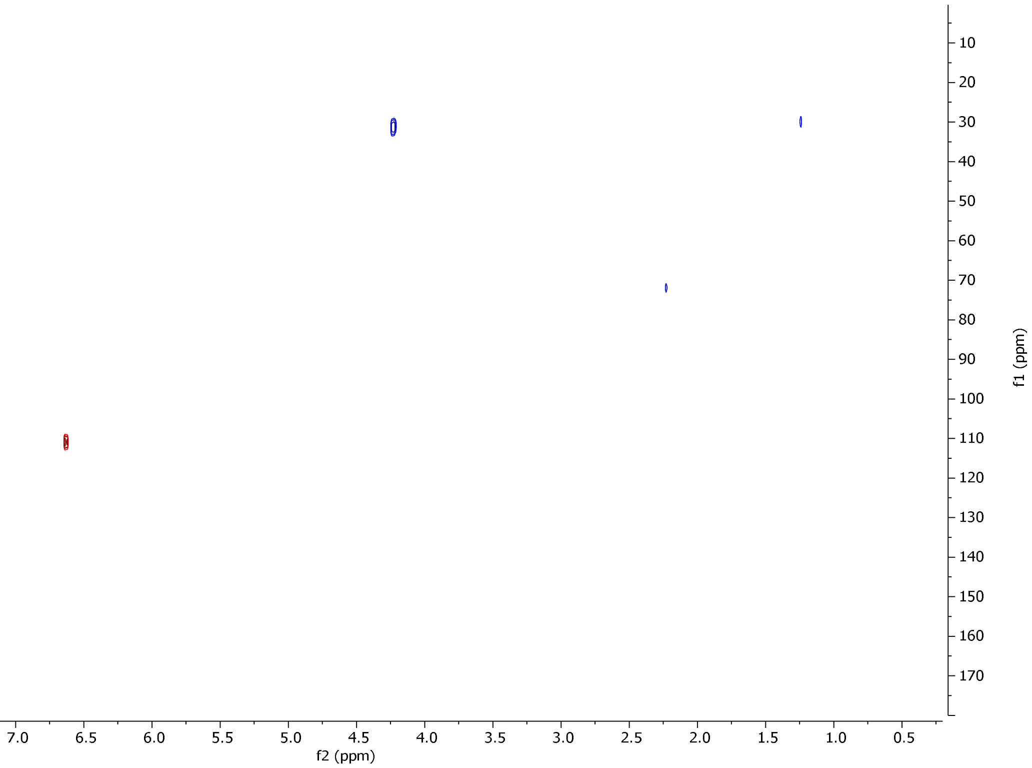

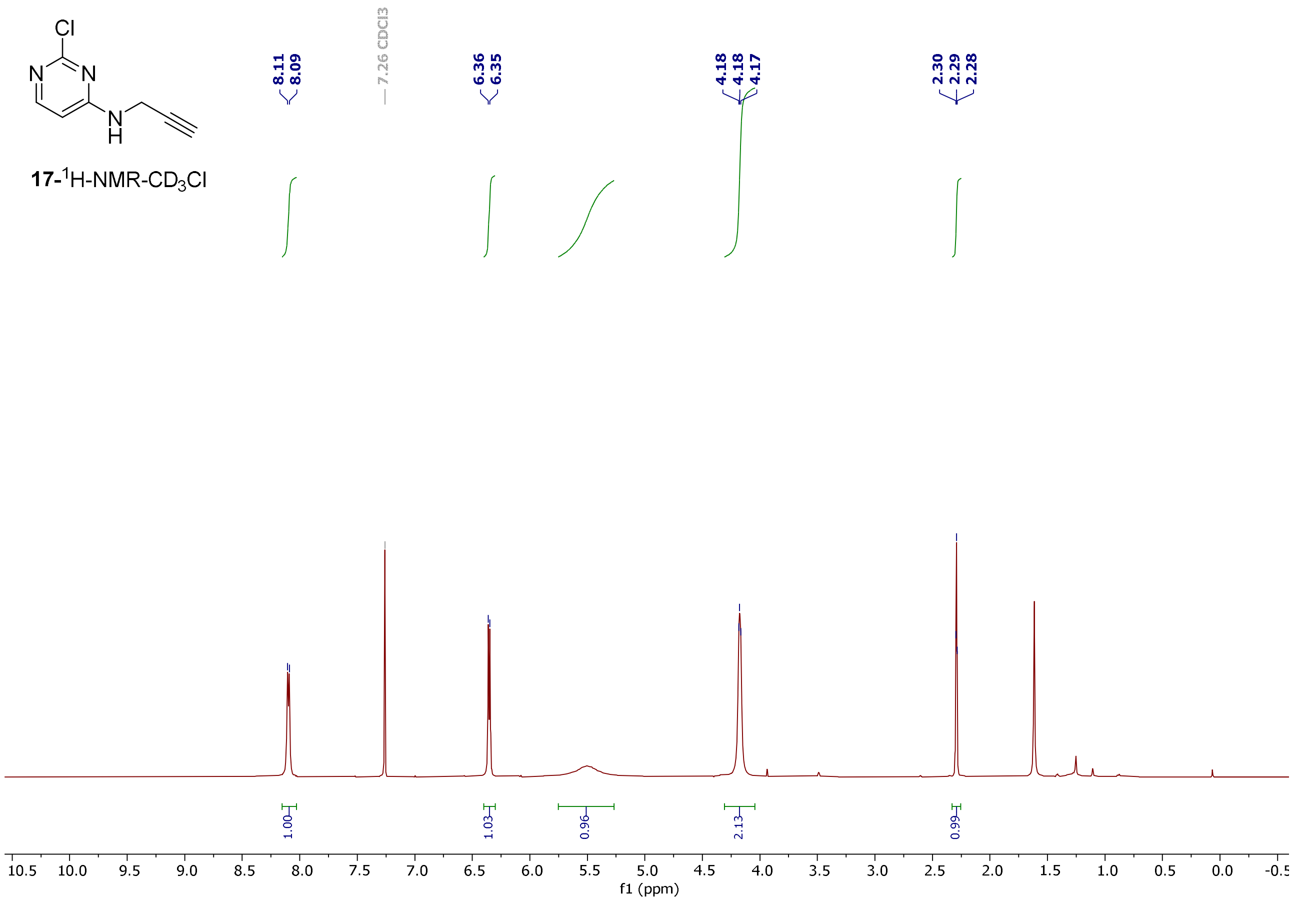
Cl
N
N
N
H
17-^{1}H-NMR-CD$_3$Cl
8.11
8.09
7.26 CDCl3
6.36
6.35
4.18
4.18
4.17
2.30
2.29
2.28
1.00
1.03
0.96
2.13
0.99
f1 (ppm)

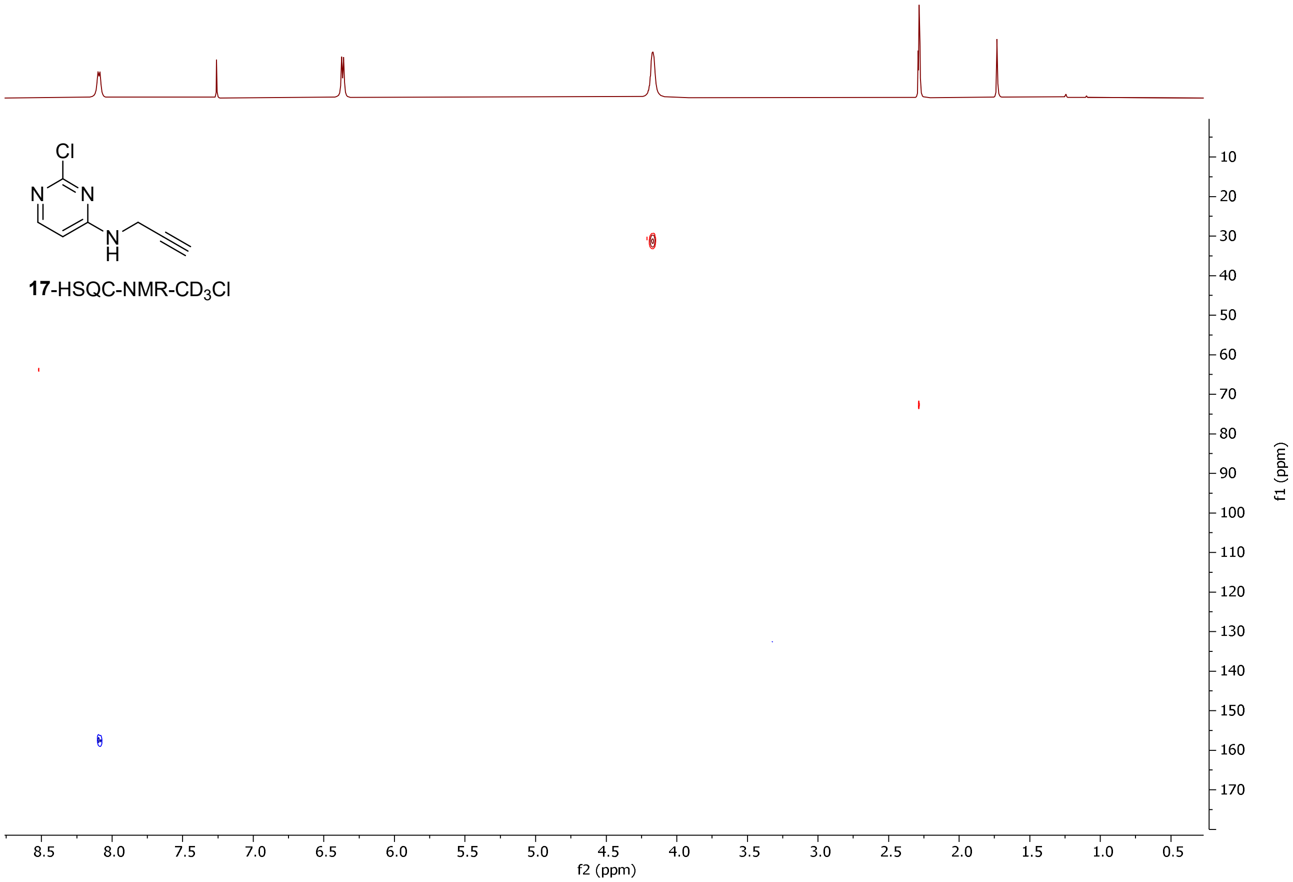
Cl
N
N
N
H
17-HSQC-NMR-CD$_3$Cl
f2 (ppm)
f1 (ppm)

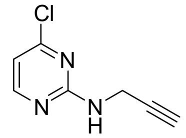

16-NOESY-CD_3Cl

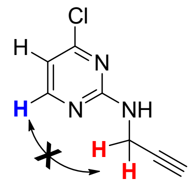

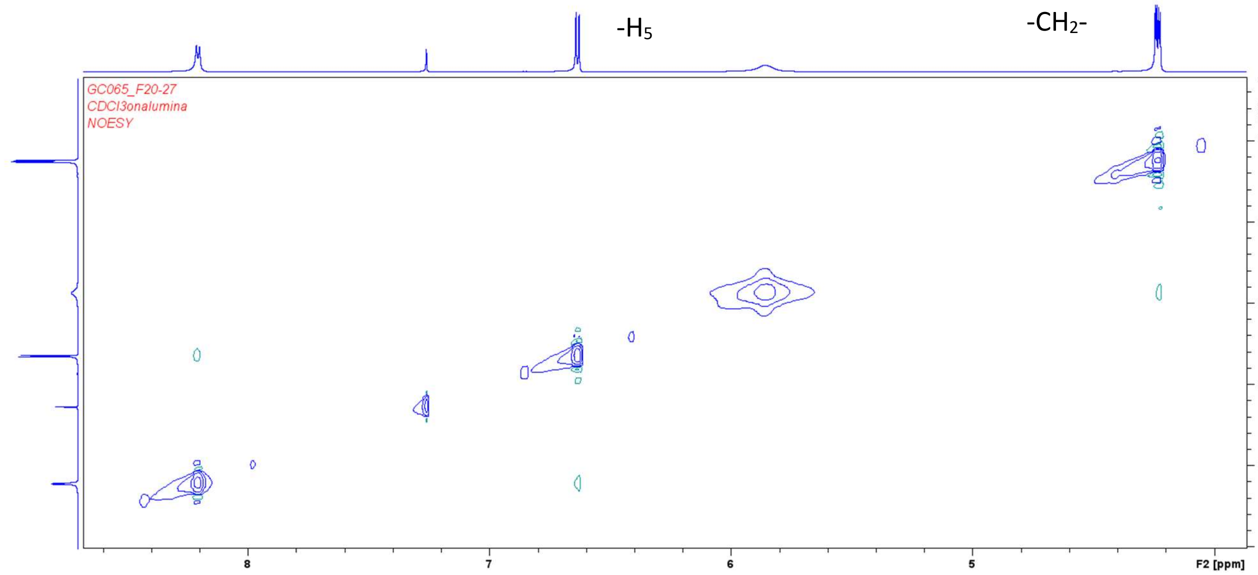

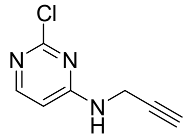

17-NOESY-CD_3Cl

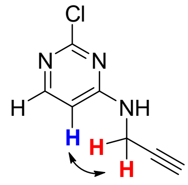

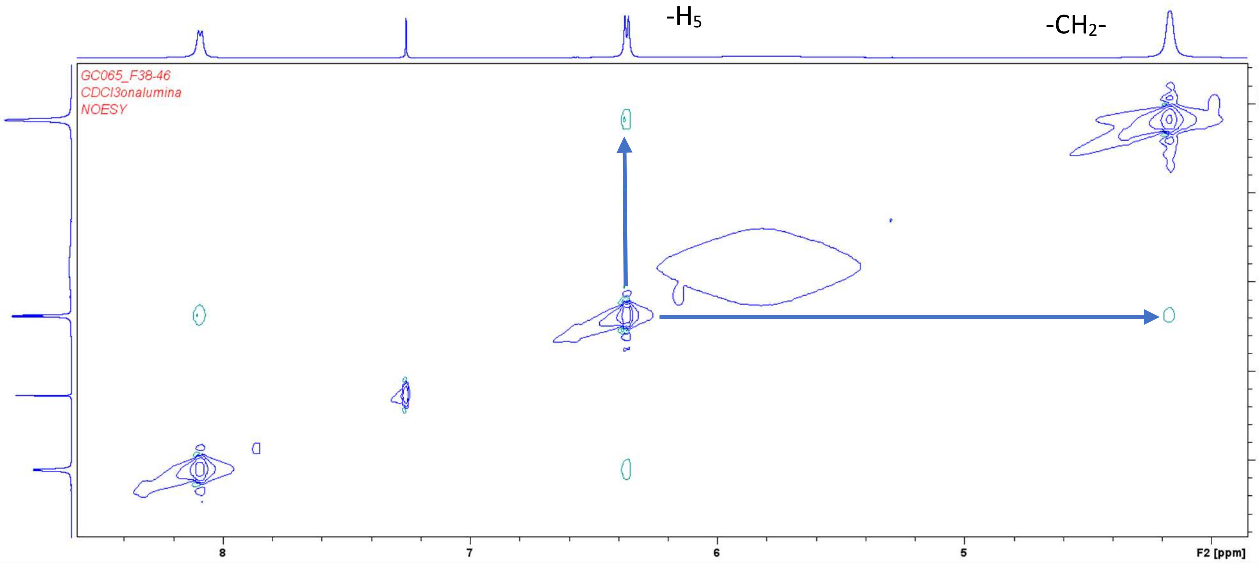

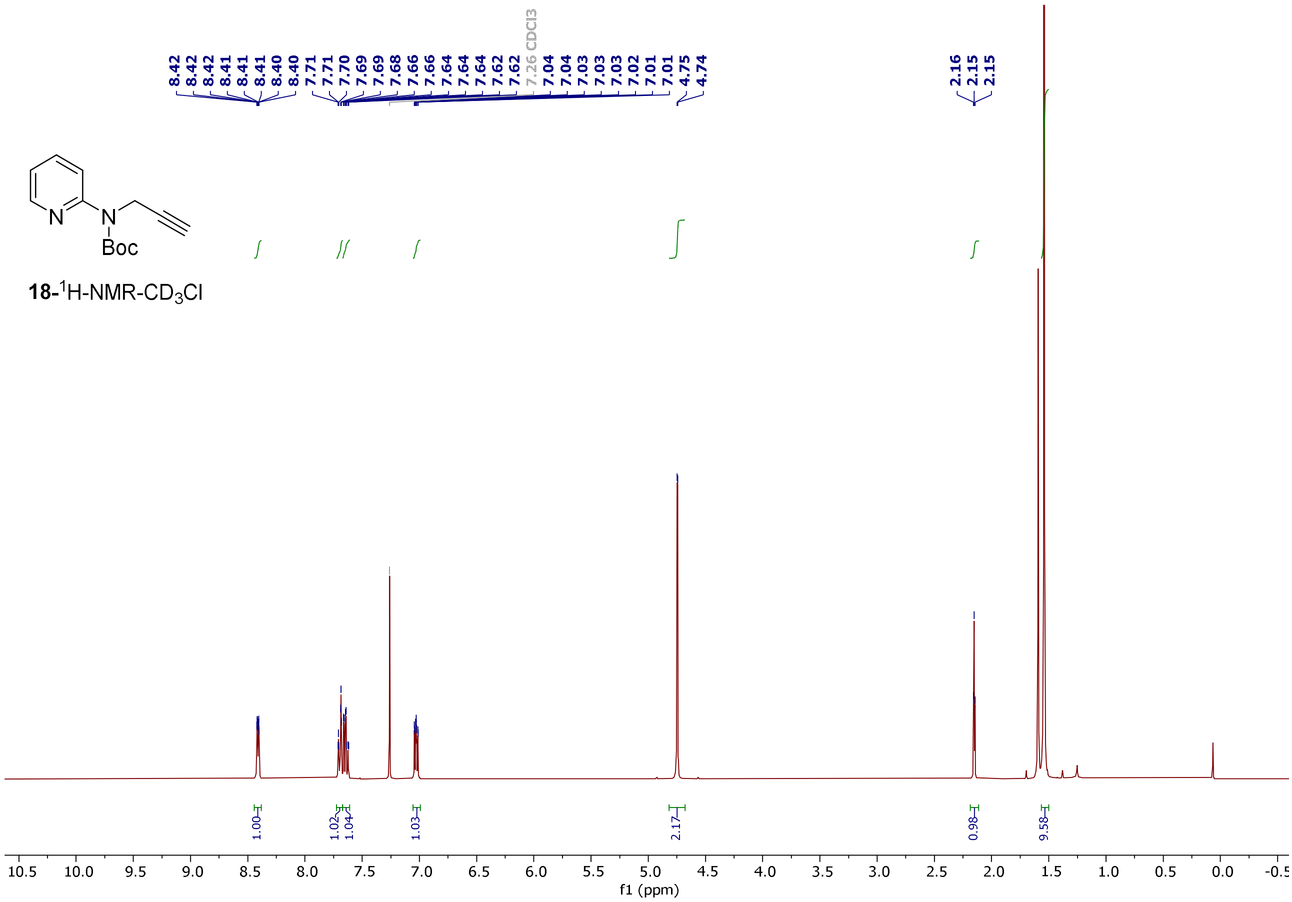

18-^{1}H-NMR-CD$_3$Cl

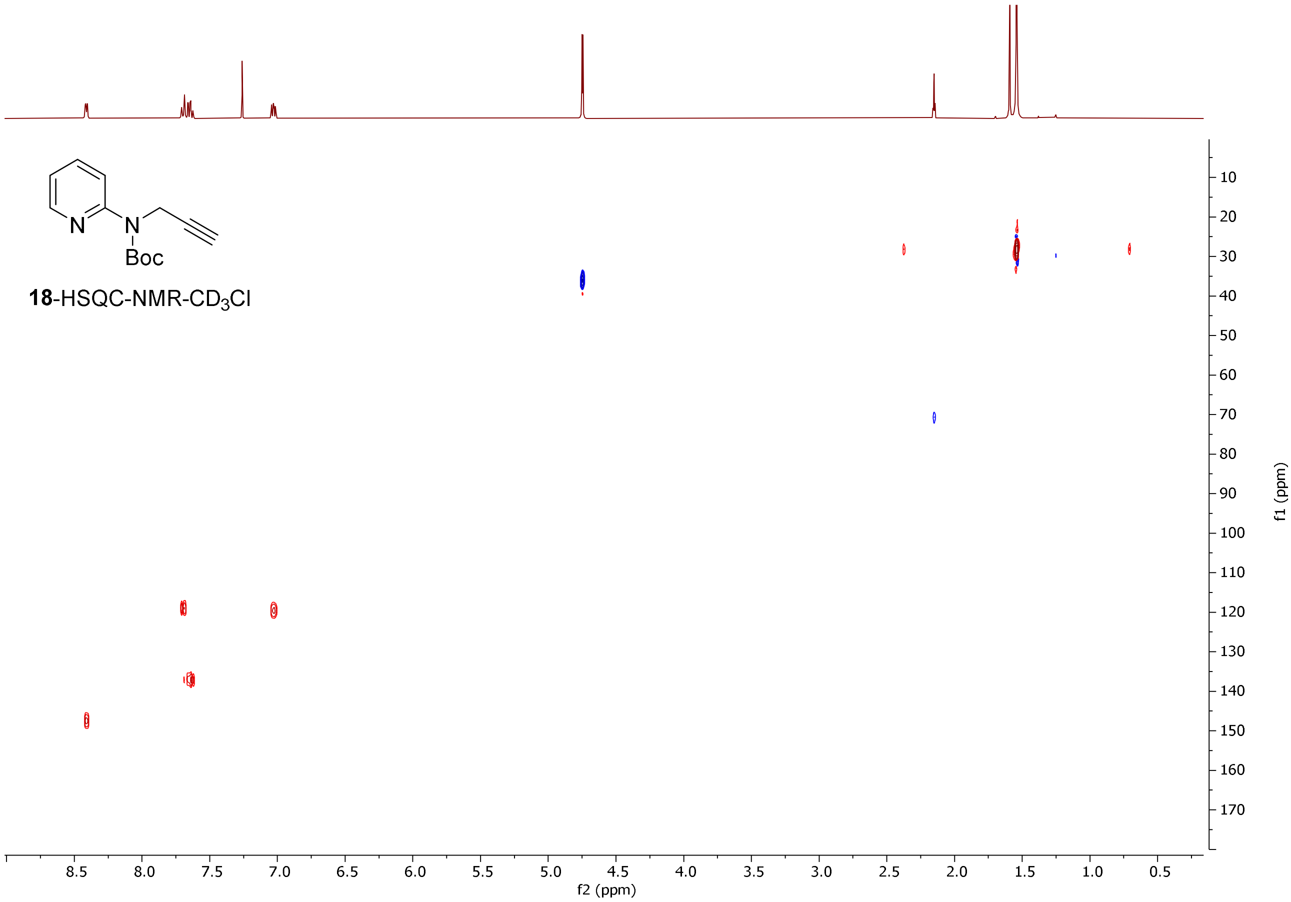

18-HSQC-NMR-CD$_3$Cl

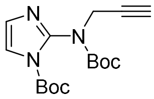

19-¹H-NMR-CD$_3$Cl

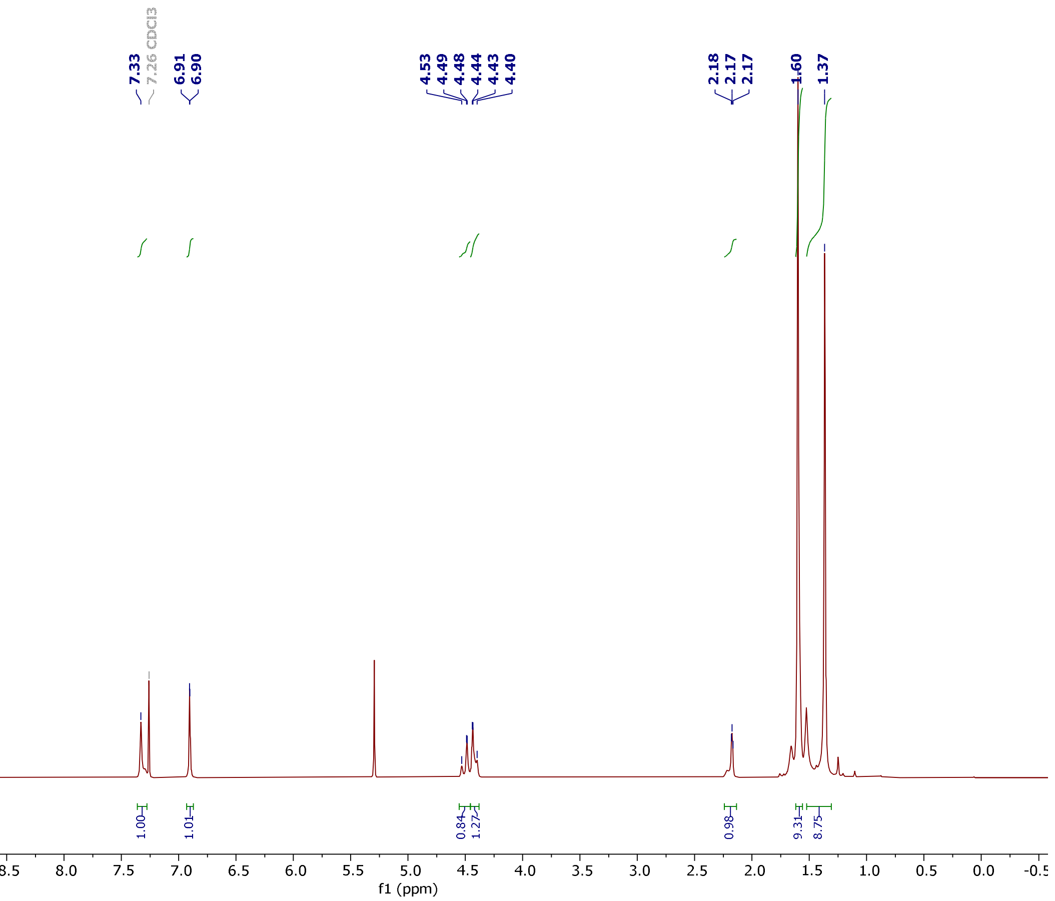

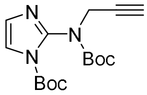

19-HSQC-NMR-CD$_3$Cl

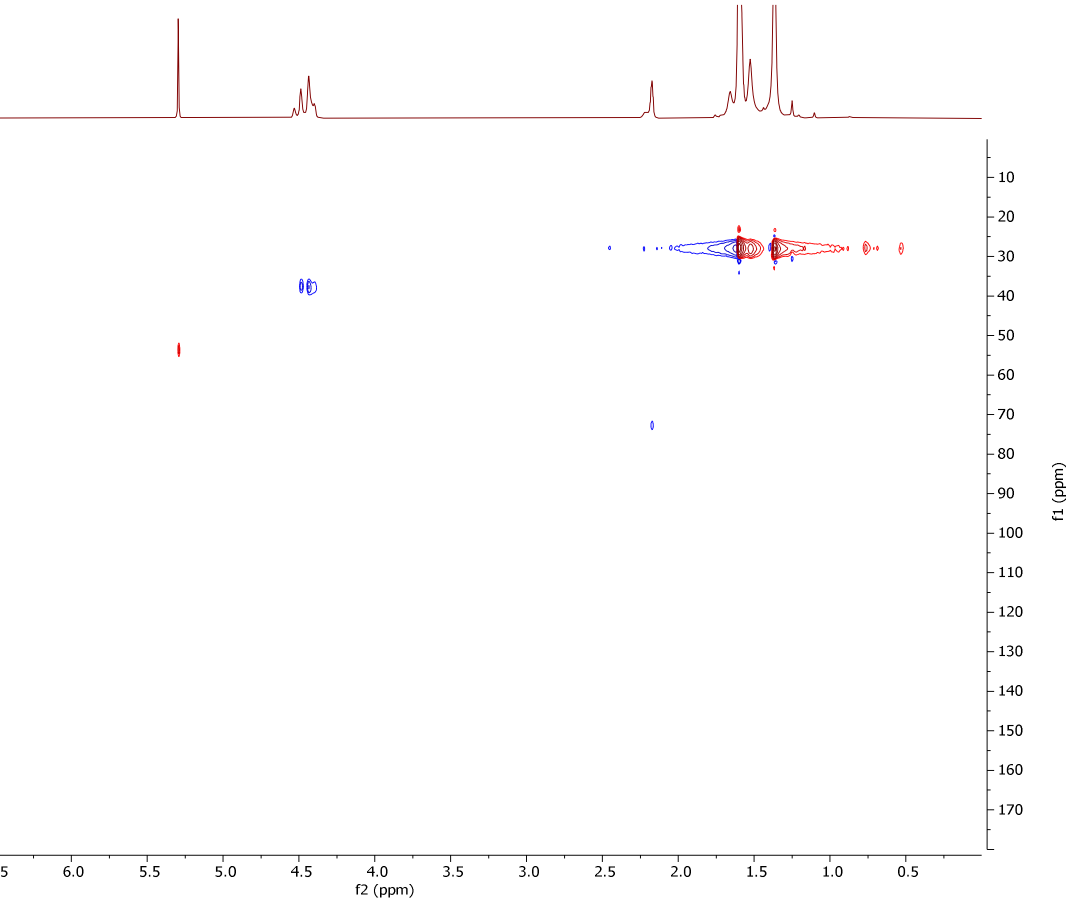

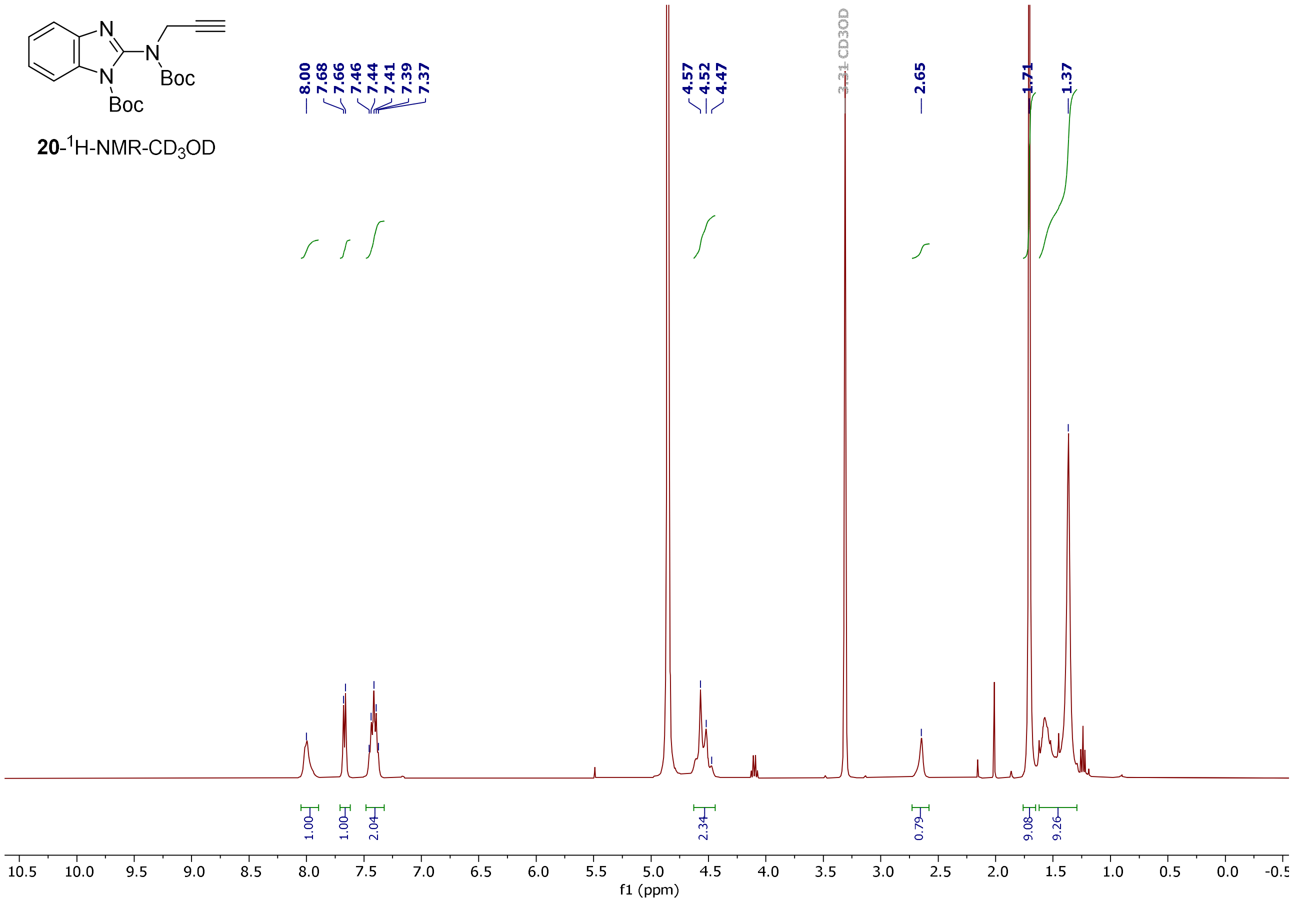

N
N
Boc
N
Boc
20-¹H-NMR-CD3OD
8.00
7.68
7.66
7.46
7.44
7.41
7.39
7.37
4.57
4.52
4.47
3.31 CD3OD
2.65
1.71
1.37
1.00
1.00
2.04
2.34
0.79
9.08
9.26
f1 (ppm)

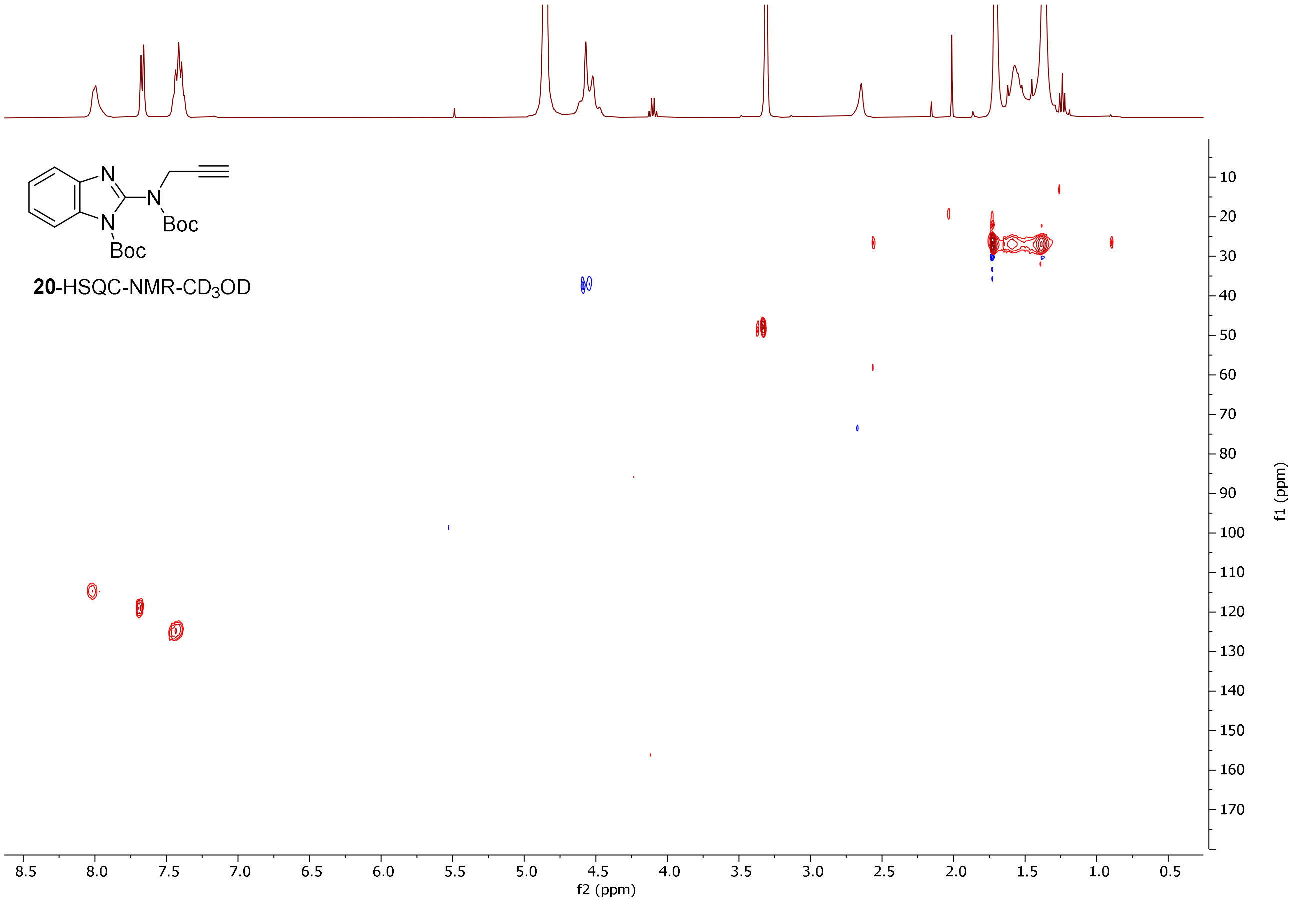

N
N
Boc
N
Boc
20-HSQC-NMR-CD3OD
f2 (ppm)
f1 (ppm)

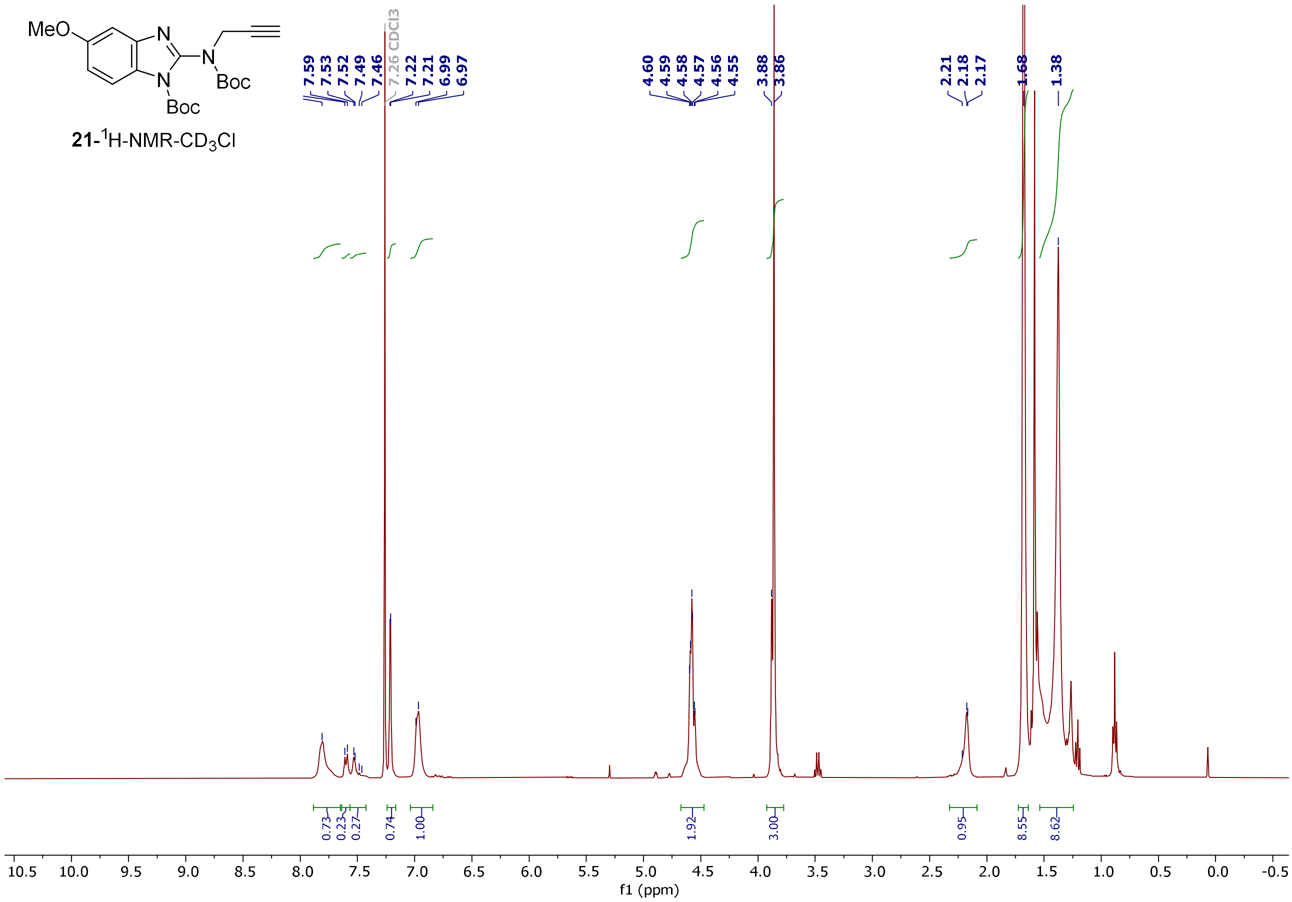

21-^{1}H-NMR-CD$_3$Cl

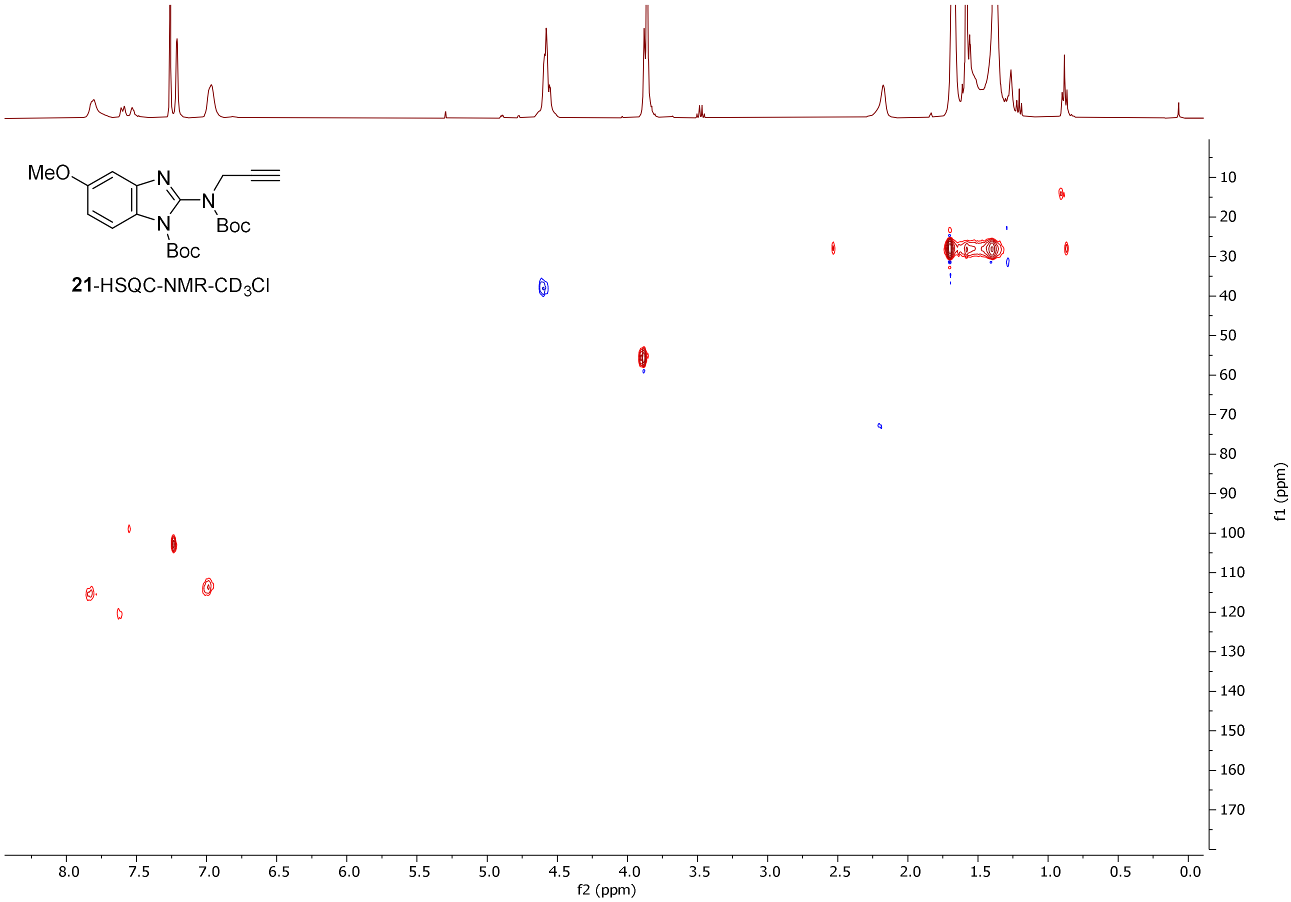

21-HSQC-NMR-CD$_3$Cl

HPLC chromatograms of 2-11

Compounds **2-11** were tested as either salts or neutral structures (as shown in TableSI-1), showing a purity >95 % by HPLC.

For analytical HPLC, the following parameters were used:

- Compounds **2**, **3**, **5**, **6**, **7** & **10**: Jasco LC-4000 equipped with a C18 cartridge (Phenomenex Luna, 100Å, 5 µm, 4.6 mm × 150 mm; flow rate 1 mL/min) and H_2O (+0.1% HCOOH or CF_3COOH) with CH_3CN (+0.1% HCOOH or CF_3COOH) gradient as mobile phase.
- Compounds **4**, **8**, **9** & **11**: Waters HPLC (515 HPLC Pump and 996 PDA) equipped with a C18 cartridge (Atlantis T3, 5 µm, 4.6 mm × 100 mm; flow rate 1 mL/min) and H_2O (+0.1% HCOOH or CF_3COOH) with CH_3CN (+0.1% HCOOH or CF_3COOH) gradient as mobile phase.

The gradient profile was optimized for effective separation of the analytes as described in the main text (see Experimental).

Compound **2**

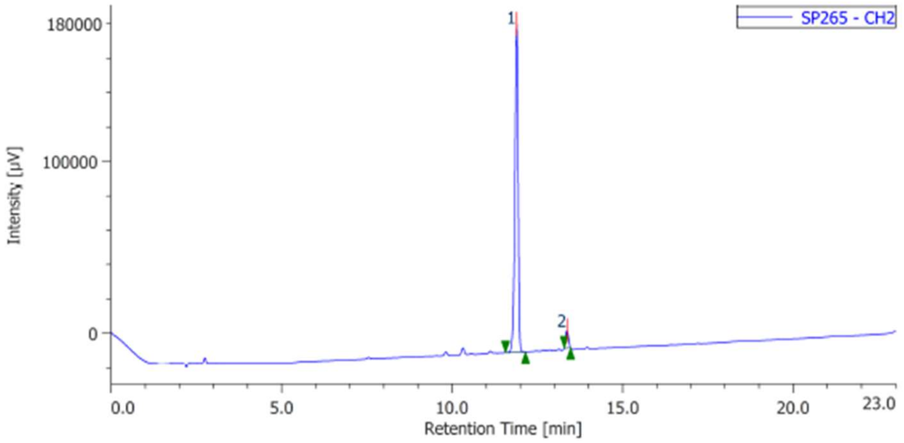

#	Peak Name	CH	tR [min]	Area [µV·sec]	Height [µV]	Area%	Height%	Quantity	NTP	Resolution	Symmetry Factor	Warning
1	Unknown	2	11.892	1239534	190912	95.382	94.847	N/A	80039	8.951	0.844	
2	Unknown	2	13.365	60019	10373	4.618	5.153	N/A	108992	N/A	1.086	

Compound **3**

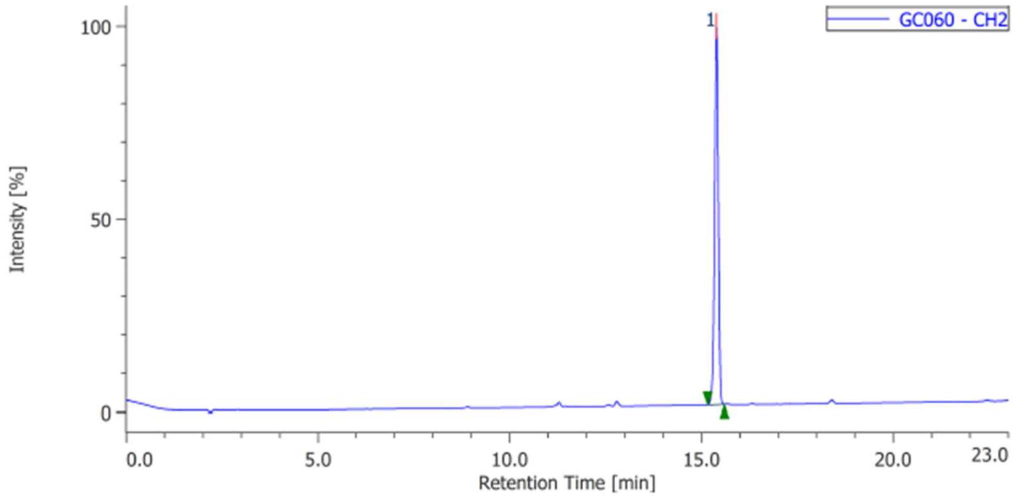

Compound **4**

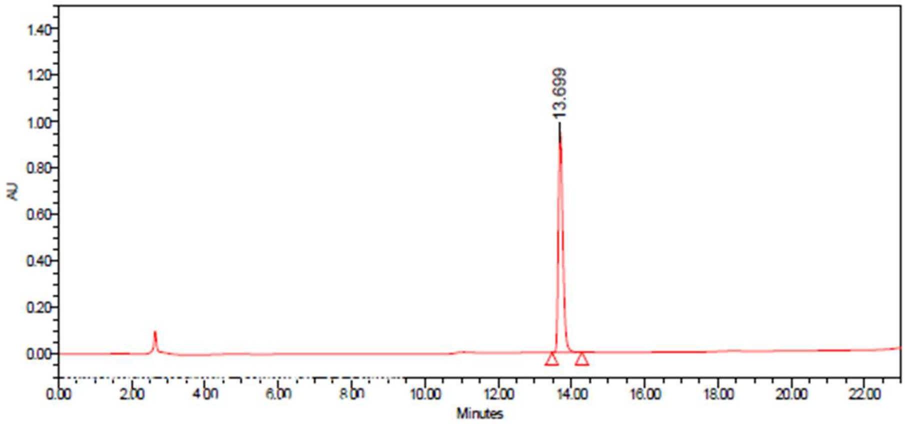

Compound **5**

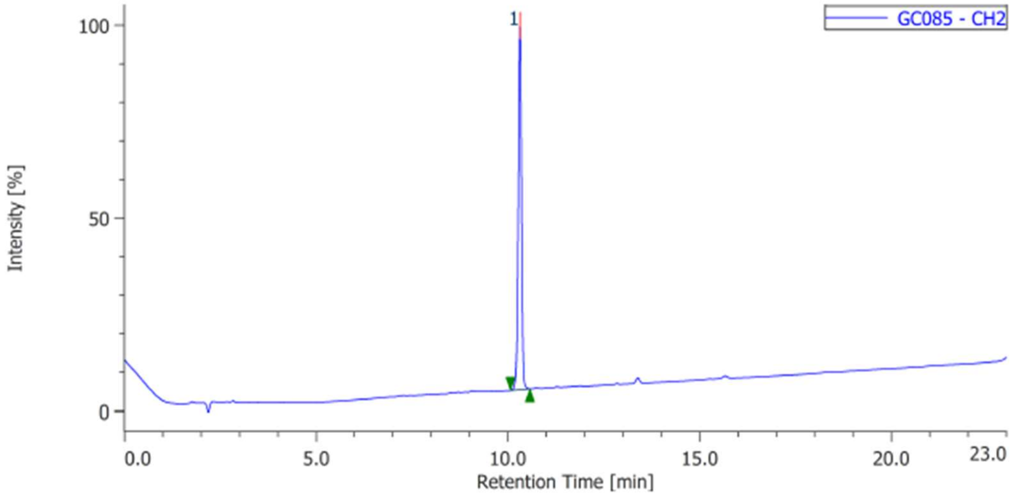

Compound **6**

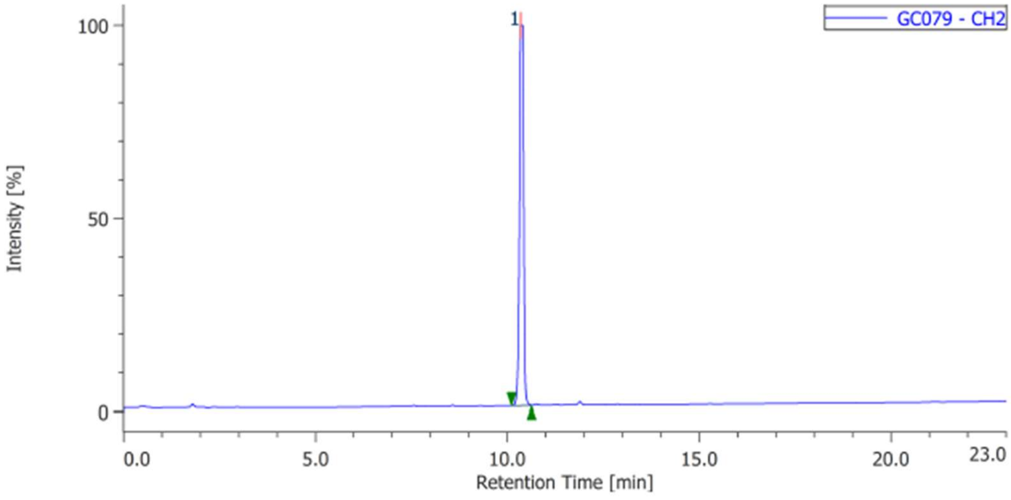

Compound **7**

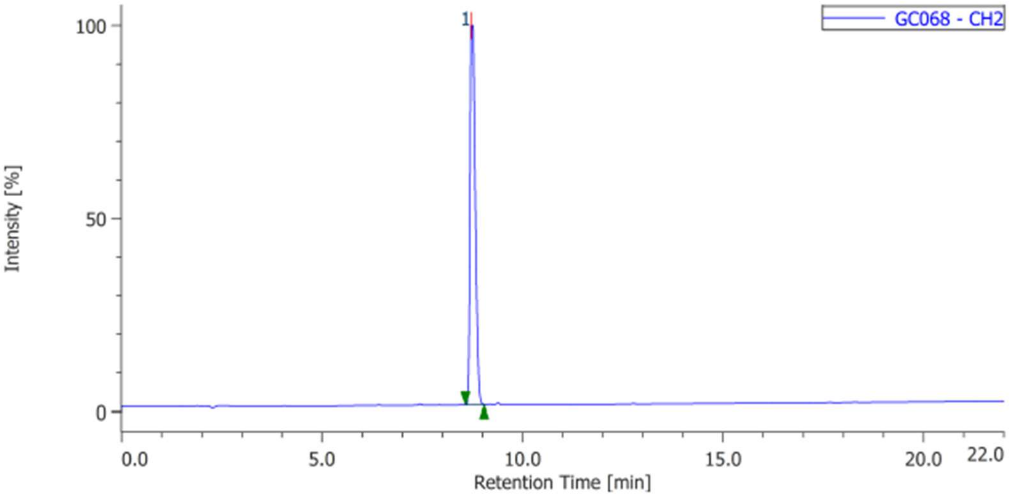

Compound **8**

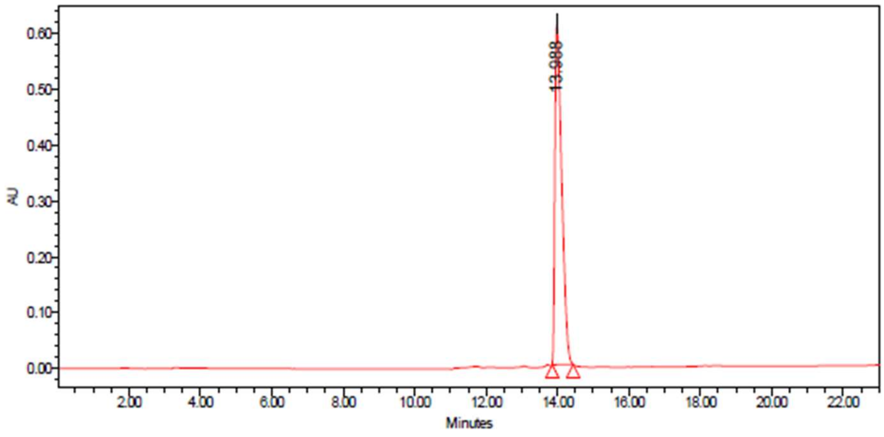

Compound **9**

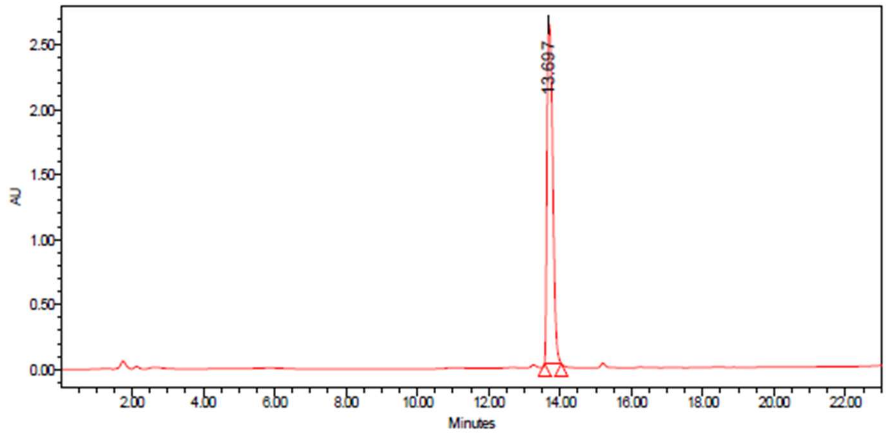

Compound **10**

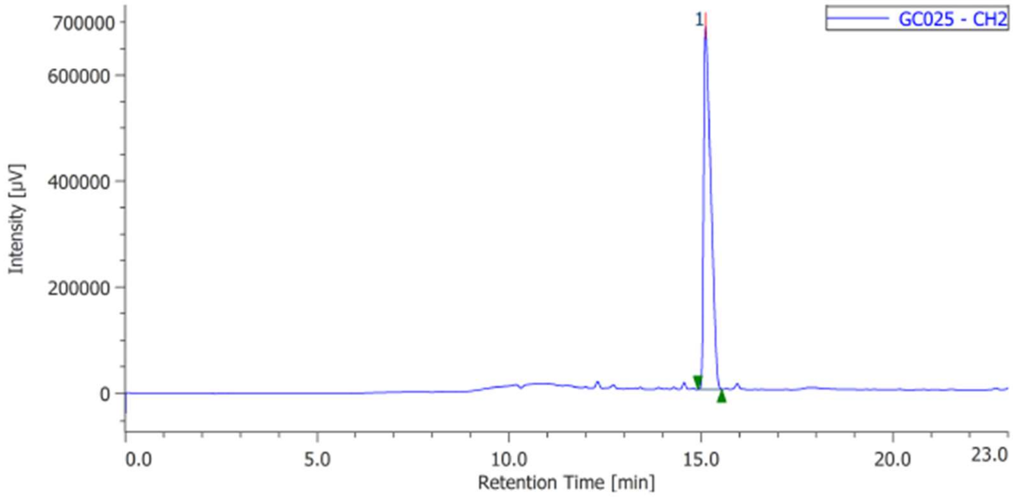

Compound **11**

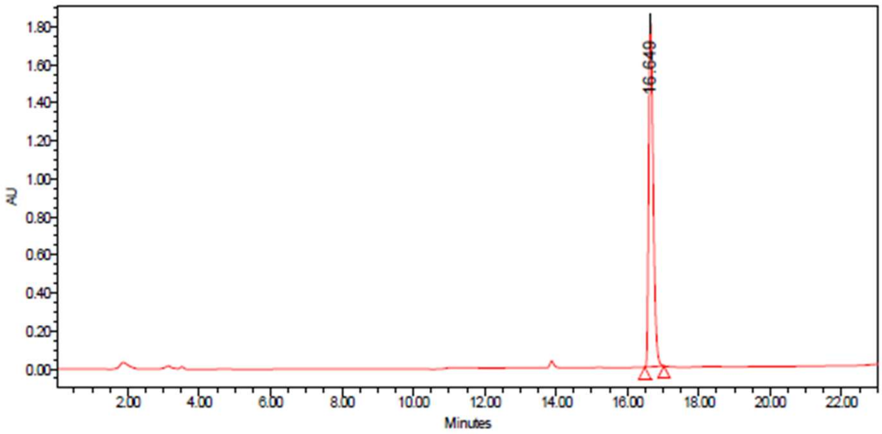

References

(1) Kabsch, W. *XDS*. *Acta Crystallogr. D Biol. Crystallogr.* **2010**, *66* (2), 125–132. https://doi.org/10.1107/s0907444909047337.

(2) Olsson, M. H. M.; Søndergaard, C. R.; Rostkowski, M.; Jensen, J. H. PROPKA3: Consistent Treatment of Internal and Surface Residues in Empirical p K_a Predictions. *J. Chem. Theory Comput.* **2011**, *7* (2), 525–537. https://doi.org/10.1021/ct100578z.

(3) Harder, E.; Damm, W.; Maple, J.; Wu, C.; Reboul, M.; Xiang, J. Y.; Wang, L.; Lupyan, D.; Dahlgren, M. K.; Knight, J. L.; Kaus, J. W.; Cerutti, D. S.; Krilov, G.; Jorgensen, W. L.; Abel, R.; Friesner, R. A. OPLS3: A Force Field Providing Broad Coverage of Drug-like Small Molecules and Proteins. *J. Chem. Theory Comput.* **2016**, *12* (1), 281–296. https://doi.org/10.1021/acs.jctc.5b00864.

(4) Friesner, R. A.; Banks, J. L.; Murphy, R. B.; Halgren, T. A.; Klicic, J. J.; Mainz, D. T.; Repasky, M. P.; Knoll, E. H.; Shelley, M.; Perry, J. K.; Shaw, D. E.; Francis, P.; Shenkin, P. S. Glide: A New Approach for Rapid, Accurate Docking and Scoring. 1. Method and Assessment of Docking Accuracy. *J. Med. Chem.* **2004**, *47* (7), 1739–1749. https://doi.org/10.1021/jm0306430.

(5) Medve, L.; Achilli, S.; Guzman-Caldentey, J.; Thépaut, M.; Senaldi, L.; Le Roy, A.; Sattin, S.; Ebel, C.; Vivès, C.; Martin-Santamaria, S.; Bernardi, A.; Fieschi, F. Enhancing Potency and Selectivity of a DC-SIGN Glycomimetic Ligand by Fragment-Based Design: Structural Basis. *Chem. – Eur. J.* **2019**, *25* (64), 14659–14668. https://doi.org/10.1002/chem.201903391.
